# Issues Relative to the Welding of Nickel and Its Alloys

**DOI:** 10.3390/ma18153433

**Published:** 2025-07-22

**Authors:** Adam Rylski, Krzysztof Siczek

**Affiliations:** 1 Institute of Material Engineering, Lodz University of Technology, 90-924 Lodz, Poland; 2 Department of Vehicles and Fundamentals of Machine Design, Lodz University of Technology, 90-537 Lodz, Poland; ks670907@p.lodz.pl

**Keywords:** nickel, Ni-based alloys, weldability, hot cracking, weld metal solidification cracking, liquation cracking, ductility dip cracking (DDC), strain age cracking (SAC), porosity

## Abstract

Nickel is used in aerospace, military, energy, and chemical sectors. Commercially pure (CP) Ni, and its alloys, including solid-solution strengthened (SSS), precipitation strengthened (PS), and specialty alloys (SA), are widely utilized, typically at elevated temperatures, in corrosive settings and in cryogenic milieu. Ni or Ni-based alloys frequently require welding realized, inter alia, via methods using electric arc and beam power. Tungsten inert gas (TIG) and Electron-beam welding (EBW) have been utilized most often. Friction stir welding (FSW) is the most promising solid-state welding technique for connecting Ni and its alloys. The primary weldability issues related to Ni and its alloys are porosity, as well as hot and warm cracking. CP Ni exhibits superior weldability. It is vulnerable to porosity and cracking during the solidification of the weld metal. Typically, SSS alloys demonstrate superior weldability when compared to PS Ni alloys; however, both types may experience weld metal solidification cracking, liquation cracking in the partially melted and heat-affected zones, as well as ductility-dip cracking (DDC). Furthermore, PS alloys are prone to strain-age cracking (SAC). The weldability of specialty Ni alloys is limited, and brazing might provide a solution. Employing appropriate filler metal, welding settings, and minimal restraint can reduce or avert cracking.

## 1. Introduction

Nowadays, due to its importance in the economy and military, Ni is regarded as a strategic metal, with production consistently rising. The main Ni sources occur in sulfide (39.8%) and laterite (59.5%) deposits [1]. Most of the Ni produced is utilized for alloying stainless steel (62.7%), manufacturing Ni-based alloys (11.9%), plating (9.7%), and other applications [2]. The distinct characteristics of Ni and its alloys categorize them as engineering materials suitable for extremely challenging environments. Such environments frequently feature very high or low temperatures and a corrosive atmosphere, causing many materials to degrade after a relatively brief period. Certain Ni-based superalloys can reliably function from cryogenic temperatures up to 1200 °C, a range unmatched by any other materials [3]. Although Ni and its alloys have several beneficial traits, it is crucial to thoroughly assess their weldability if joining is necessary for product fabrication. While fusion welding is a prevalent joining method, when an alloy exhibits poor weldability, brazing and solid-state welding techniques may be preferred options. The weldability of many Ni and Ni-based alloys is quite good, indicating that a quality welded joint can be achieved fairly easily if the correct welding method is used. Welding certain Ni-based alloys may lead to significant metallurgy-related defects that typically appear as cracking in the weld metal (WM), partially melted zone (PMZ), and heat-affected zone (HAZ). The SSS and PS Ni-based alloys are particularly susceptible to these defects. Various forms of weld joint cracking found in these alloys include solidification and liquation cracking, ductility-dip cracking (DDC), and strain-age cracking (SAC). In terms of weldability, Ni-based SAs are considerably more challenging. Improper welding techniques may also lead to defects associated with the process when bonding Ni and Ni-based alloys. Common instances involve porosity and inclusions in the WM zone, undercuts, insufficient fusion and penetration, and distortion of the workpiece. These flaws can significantly impact the fatigue lifespan and corrosion resistance of constructed materials [4]. Kojundžić et al. [5] examined Ni and its alloys regarding their characteristics, uses, and challenges related to weldability. Basic recommendations to prevent weldability issues are outlined for four specific categories of Ni and its alloys: CP Ni, SSS alloys, PS alloys, and SAs.

This paper aims to review Ni and its alloys regarding their characteristics, uses, and challenges in welding.

## 2. Classification, Features and Applications of Ni and Ni-Based Alloys

Ni forms a face-centered cubic (FCC) lattice structure and does not exhibit any allotropic forms. It dissolves easily with various other metals, facilitating the creation of numerous Ni-centered alloys. It is a ferromagnetic metal displaying a shift from a ferromagnetic to a paramagnetic state. Ni is a dense, heavy metal (density above 5000 kg/m^3^ at 25 °C) renowned for its excellent electrical and thermal conductivity as well as remarkable corrosion resistance. Table 1 and Table 2 illustrate Ni’s fundamental physical and mechanical characteristics. In contrast to metals with a body-centered cubic (BCC) structure, Ni does not exhibit a ductile-to-brittle transition temperature (DBTT).

### 2.1. Classifying Ni and Its Alloys

Literature does not provide a systematic classification framework for Ni and its alloys (similar to that for steel and aluminum alloys). Ni alloys are typically identified by their brand names and the manufacturer’s alloy numbers. Several common trade names include Chromel (Ni-Cr alloys), Nimonic (Ni-Cr-Co alloys), Inconel and Incoloy (Ni-Cr-Fe alloys), Hastelloy (Ni-Mo-Fe alloys), and Monel (Ni-Cu alloys).

Ni and its alloys are often categorized according to their chemical makeup and divided into four separate groups: CP Ni alloys, SSS alloys, PS alloys, and SAs, as shown in Table 3.

#### 2.1.1. CP Ni

CP Ni has a content of over 99 wt.% Ni, with the rest made up of trace elements (C, Co, Cu, Mg, Mn, O) and impurities (S, P, Si) [5]. Sometimes, as impurities therein are considered Mn, Fe, Si, and Cu. Such alloys cannot be heat-treated and can be strengthened through cold work. Certain alloys (301) also include small amounts of Al and Ti as alloying elements. The alloys that have these components can be heat-treated and have the potential to be enhanced through precipitation hardening. They are utilized for components that need a significant degree of mechanical strength, like bolts and springs. Chemical compositions of some CP Ni alloys are presented in Table 4 [12].

CP Ni exhibits strong corrosion resistance in oxidizing environments and outstanding resistance in alkaline solutions, non-oxidizing acids, and halogen gases. CP Ni exhibits excellent thermal and electrical conductivity [12].

The mechanical characteristics of CP Ni are influenced by impurity levels and the history of mechanical and thermal processing, which determine its microstructural traits, especially grain shape and size. Table 5 displays the chosen mechanical properties of CP Ni alloys. Table 6 presents high-temperature strength and physical properties of soft-annealed Nickel 201 (based on [13]). They can be useful during the modeling of the welding processes.

Industrial uses of CP Ni mainly focus on extremely corrosive settings (chemical sector) because of its exceptional resistance to corrosion and tarnishing. Moreover, a variety of CP Ni alloys were specifically formulated for use in high-temperature applications and unique electrical requirements [5].

CP Ni is utilized in the production of equipment for food processing, containers for chemicals, equipment for handling caustics, components for electrical and electronic devices, anodes for electroplating, heat exchangers, fluorescent lighting, and both decorative and protective coatings [12].

CP Ni alloy can be soft-annealed (705–925 °C), stress relieved (480–705 °C), or stress equalized (260–480 °C). Higher temperature choice results in higher ductility. In each case, air cooling may be applied [13].

Postweld heat treatment for caustic applications needs heating the material to 705 °C and maintaining such a temperature for at least 0.5 h per 25 mm of thickness. The heating and cooling rates are affected based on the shape of the part. For complex shapes with nonuniform thicknesses, heating and cooling rates of 111 °C per hour should be used. Shapes with uniform thicknesses can employ faster heating and air cooling [13].

Recommended parameters of heat treatment of CP Ni are as follows [13]:

- Batch soft-annealing in bell-type furnaces: 705–760 °C; 2–6 h; AC.
- Continuous soft-annealing: 815–925 °C; 5 min; AC/WQ.
- Stress relieving: 480–705 °C; 30–120 min; AC.
- Stress equalizing: 260–480 °C; 1–2 h; AC.
- Postweld annealing: 705 °C; AC.

#### 2.1.2. SSS Ni-Based Alloys

SSS Ni-based alloys contain various soluble elements (Cu, Mo, Fe, Cr, W, Co, Ti, Al) that enhance the strength of the Ni matrix by hindering dislocation movement [5].

Mechanical characteristics of SSS Ni-based alloys are presented in Table 7. The tensile strength of solid-solution alloys varies between 550 and 830 MPa, whereas the yield strength spans from 275 to 450 MPa [3]. Alloys 800HT and 800H exhibit excellent strength and resistance to creep at elevated temperatures [14,15,16]. Moreover, these alloys maintain excellent toughness even at cryogenic temperatures.

Industrial uses of SSS Ni alloys are typically designed for corrosive environments and very high operating temperatures (up to 1200 °C) [3]. They are frequently utilized in the chemical, thermal processing, maritime, petrochemical, and aerospace sectors. For instance, Alloy 600 shows significant resistance to reducing environments, whereas Alloy 601 demonstrates outstanding oxidation resistance.

**Ni-Cu based alloys**

Ni-Cu Alloys (branded as Monel) are Ni-based alloys that consist of 29–33% Cu, which serves as the primary alloying element. Cu and Ni create a single-phase solid solution. Ni-Cu Alloys, featuring 3% Al and 0.6% Ti as supplementary alloying elements (Monel K-500), are capable of heat treatment and can be enhanced through precipitation hardening. Ni-Cu Alloys exhibit excellent corrosion resistance in acidic and alkaline environments, possess strong mechanical strength along with good ductility, and have a low thermal expansion coefficient. The alloys have low machinability. Ni-Cu Alloys (Monel) are utilized for producing equipment for chemical processing, valve stems, springs, pumps, shafts, fittings, heat exchangers, screw machine products, and marine apparatus.

Chemical composition of certain Ni-Cu alloys is shown in Table 8 [12]. Mechanical properties of certain Ni-Cu alloys are shown in Table 9 [12]. Table 10 presents high-temperature strength and physical properties of alloys 400 and K-500. They can be useful during the modeling of the welding processes.

Hardness for K-500 alloy varied with temperature, reaching the following values: 311 HBW at 370 °C, 302 HBW at 425 °C, 293 HBW at 480 °C, 255 HBW at 540 °C, and 229 HBW at 595 °C, respectively [18].

Recommended heat treatment parameters for 400 alloy are the following [17]:

- Soft annealing: 700–900 °C;
- Tempering: 550–650 °C.

Recommended heat treatment parameters for K-500 alloy are the following [18]:

Solution annealing of hot-finished products is conducted at 980 °C, and of cold-worked ones at 1040 °C. To avoid excessive grain growth, the time at temperature ought to be kept to below 30 min. Both heating and cooling need to be conducted as fast as possible.

Intermediate annealing may be needed to soften the product during the production of the forming process. It is usually conducted at a temperature ranging from 760 to 870 °C for 1 h.

Age hardening procedures are as follows:

- Soft material (140–180 HBW)—as-forged and quenched or annealed forgings, annealed or hot-rolled rods, large cold-drawn rods, and soft-temper wire and strip:
  o Age hardening: 580–610 °C; 16 h;
  o Furnace cooling: 12 °C/h down to 480 °C;
  o Air cooling.
- Moderately cold-worked material—cold-drawn rods, half-hard strip, cold-upset pieces, and intermediate-temper wire:
  o Age hardening: 580–610 °C; 8 h;
  o Furnace cooling: 12 °C/h down to 480 °C;
  o Air cooling.
- Fully cold-worked material (260–325 HBW, 25–35 HC)—spring-temper strip, spring wire, or heavily cold-worked pieces such as small, cold-formed balls:
  o Age hardening: 520–540 °C; 6 h;
  o Furnace cooling: 12 °C/h down to 480 °C;
  o Air cooling.

**Ni-Mo-based alloys**

As temperatures rise, diffusion-controlled processes start to significantly affect the mechanical properties of Ni-based superalloys subjected to creep load. In a high-temperature and low-stress creep environment, refractory elements with slow diffusion, like Mo, are utilized to enhance creep resistance [19].

The chemical composition of certain Ni-Mo alloys is shown in Table 11. The mechanical properties of certain Ni-Mo alloys are presented in Table 12. Table 13 shows high-temperature strength and physical properties of alloys B2 and B3. They can be useful during the modeling of the welding processes.

Alloy B2 is commonly utilized in harsh reducing environments. It contains considerably less carbon, Si, and Fe than its predecessor, alloy B (UNS N10001). In the as-welded state, alloy B2 is more resistant to weld zone corrosion than alloy B. Managing additional alloying elements like Fe and Cr addressed possible challenges with formation and shaping. Rigorous chemistry management in the manufacture of alloy B2 enables its application in the welded state, and it exhibits reduced vulnerability to stress corrosion cracking in various conditions. It is important to choose the appropriate alloy for the intended use. Alloy B2 must be avoided at temperatures ranging from 1000 °F to 1600 °F because it develops secondary phases that may reduce the material’s ductility. Alloy B-2 is appropriate for application in the chemical processing sector, particularly in regions where HC acid, phosphoric acid, and sulfuric acid are utilized or handled. Alloy B-2 has additionally been utilized in manufacturing pharmaceuticals, acetic acid, ethylene alkylation, and herbicides. Common end use applications encompass pumps, valves, mechanical seals, rupture disks, flanges, fittings, tanks, and vessels [21]. Due to the elevated Mo level, alloy B2 has outstanding resistance to hydrochloric acid across a broad spectrum of concentrations and temperatures. Alloy B2 exhibits strong resistance to HCl, sulfuric acid, and phosphoric acids, and demonstrates outstanding resilience to pitting and stress corrosion cracking in the heat-affected zone. The uniform corrosion rates in different environments are comparable to those of other Ni-Mo alloys like alloy B3. The existence of any oxidizing substances, even in minimal quantities, will substantially enhance corrosion. Alloy B2 must not be utilized in oxidizing environments, as these alloys exhibit minimal to no resistance in those conditions [21].

Alloy B3 exhibits outstanding resistance to hydrochloric acid across all concentrations and temperatures. It can also resist sulfuric, acetic, formic, and phosphoric acids, along with other non-oxidizing substances. The B3 alloy can reach a level of thermal stability significantly better than that of its predecessors, such as B2 alloy. It shows remarkable resistance to pitting corrosion, stress-corrosion cracking, and attacks from knife-line and heat-affected zones. The enhanced thermal stability of alloy B3 leads to a lower likelihood of forming harmful intermetallic phases in alloy B3, thus granting it superior ductility compared to B-2 alloy under different thermal cycling conditions and afterward. B3 exhibits excellent overall characteristics for forming and welding. It can be forged or otherwise shaped while hot, as long as it is maintained at 1230 °C for a duration adequate to heat the whole piece. Due to being a low-carbon alloy, utilizing lower hot finishing temperatures may be essential for controlling grain size. B3 can additionally be created through cold working. While it does work-harden fairly quickly, B3 parts can be produced using all standard cold forming methods. The uniform corrosion resistance of B3 alloy to chloric acid remains unchanged by cold reductions of up to 50% when compared to the alloy in its solution heat-treated state. B-3^®^ can be utilized as a replacement for B2 alloy. Similarly to B2 alloy, B3 should not be employed in environments containing ferric or cupric salts, as these salts can lead to quick corrosion failure. The top benefit of alloy B3 compared to B2 alloy comes from its capacity to retain outstanding ductility when subjected to short-term exposures at intermediate temperatures. Such exposures are commonly encountered during heat treatments linked to manufacturing. Although brief exposures at temperatures like 700 °C can greatly embrittle B2 alloy, B-3^®^ shows significant resistance at that temperature. B3 shows a significant tolerance to this kind of embrittlement for several hours. This allows for much easier fabrication of the alloy into intricate parts like shaped heads [21].

Recommended heat treatment parameters for B2 alloy are the following:

Soft annealing must occur at temperatures between 1095 and 1185 °C for either 5–10 min (continuous annealing) or 60 min (batch annealing). The item must be submerged in water or quickly cooled in the air. Alloy B2 forms detrimental intermetallic compounds Ni_3_Mo and Ni_4_Mo when exposed to temperatures between 540 and 815 °C, regardless of whether the exposure lasts from 10 to 15 min. Thus, both heating and cooling ought to occur as swiftly as feasible. Place the treated component into the furnace that has reached the annealing temperature and utilize furnaces with high temperature capabilities [22].

Recommended heat treatment parameters for B3 alloy are the following:

Solutioning: 1060 °C; water or rapid air cooling [23].

The NiMo master alloy provided by KBM Affilips is utilized in manufacturing stainless steels, special steels, and superalloys for solid solution strengthening, precipitation hardening, deoxidation, desulfurization, and more. NiMo50 Master Alloy is composed of 50% Mo balanced by the remainder Ni. The Ni-Mo 50% master alloy (NiMo50) provides a simple and dependable method for incorporating Mo into Ni alloys, such as super alloys. It possesses a lower density and a lower melting point compared to Mo, which has a melting point of 2610 °C and a density of about 10.2 g/cm^3^. NiMo is employed for precipitation hardening and solid solution strengthening. NiMo is also utilized in creating intricate carbides in Ni-containing super alloys [24].

Mehta et al. [25] examined the microstructure, texture, and flow behavior dependent on orientation of Ni-16Mo alloys in hot rolled and annealed states. The elevated and reduced stacking fault energy levels connected to Ni-16Mo lead to partial recrystallization post-recovery and the formation of twins in the recrystallized grains, respectively. The alloy exhibits two gradients in the actual plastic stress–strain curve and adheres to the Ludwigson relation. The low-strain regime of the Ni-16Mo alloy is associated with the existence of uniformly distributed and closely spaced slip lines, along with minor amounts of both deformation twins and strain localization. The primary characteristics of the high-strain regime in the alloy Ni-16Mo show significant volume fractions of deformation twins and strain localization, accompanied by a limited number of coarse penetrating slip lines crossing the grain boundaries.

Tawancy [26] examined the vulnerability of certain commercially available SSS Ni-base alloys to the formation of intermetallic compounds during aging at high temperatures and the associated impacts on mechanical properties. Based on the precise chemical makeup and aging temperature, along with the morphology of the precipitate, certain alloys may be prone to forming harmful intermetallics, especially Ni_3_Mo, Ni_4_Mo, µ, σ, δ Ni_3_Nb, and the Laves phase. Nevertheless, it is observed that specific intermetallics can yield an excellent combination of strength and ductility, such as Ni2(Mo, Cr) with a Pt_2_Mo-type superlattice, along with the γ″ phase of Ni_3_Nb featuring a DO22-type superlattice. Furthermore, it has been shown that in certain situations, a minor inclusion of an alloying element like Fe to a specific alloy may slow down the kinetics of creating harmful intermetallic compounds; nonetheless, a comparable addition to a different alloy might result in a contrary effect.

Interestingly, Yang et al. [27] created a novel category of materials that utilize the dispersion strengthening of SiC particles along with precipitation strengthening through nano-precipitates for use in molten salt nuclear reactors. A series of SiC (0.5–2.5 wt.%) reinforced dispersion and precipitation strengthened (DPS) NiMo-based alloys was produced using a mechanical alloying (MA) process, followed by spark plasma sintering (SPS), rapid cooling, high-temperature annealing, and water quenching. The NiMo matrix in these alloys is significantly bolstered by the distribution of SiC from the original powder blend and nano-Ni3Si particles, which formed during the sintering/annealing process. In addition, the matrix is enhanced by a solid solution of Mo within Ni. These NiMo alloys benefit from dispersion, precipitation, and solid solution strengthening, resulting in their exceptional mechanical properties.

**Ni-Fe-based alloys**

Ni-Fe (NiFe) alloys for temperatures reaching 600 °C comprise Nifethal^®^ 70 and Nifethal^®^ 52 low-resistivity alloys possessing a high temperature coefficient of resistance. The positive temperature coefficient enables heating elements to decrease power as the temperature rises. Common uses include low-temperature tubular components with self-regulating characteristics [28]. Table 14 presents the components of selected Ni-Fe-based alloys. Table 15 shows the physical and mechanical properties of selected Ni-Fe-based alloys.

Sun [30] created a wrought Ni-Fe-based alloy that exhibits outstanding creep rupture life for use in 700 °C-class advanced ultra-supercritical (A-USC) steam turbine rotor applications. In tensile tests, the Ni-Fe-based alloy shows outstanding yield strength at 700 °C, surpassing that of several other Ni-based/Ni-Fe-based alloys. Trans-granular and intergranular fracture modes were noted at room temperature, 700 °C, and 750 °C. Nevertheless, the intergranular fracture mode became prevalent above 700 °C. Dynamic recrystallization took place at both 700 °C and 750 °C, accompanied by rising average misorientation angles. The volume fraction of the γ′ precipitate was approximately 20%, and the average size of the γ′ precipitates was about 30 μm, showing no significant alteration following the tensile tests. The main deformation processes were planar slip at ambient temperature, the Orowan mechanism for the bypass of γ′ precipitates, and dislocation shearing at 700 °C and 750 °C. The tensile properties, fracture behaviors, and deformation mechanisms are strongly correlated.

Ni-Fe alloys are utilized as soft magnetic materials, for glass-to-metal bonding, and as materials with specified thermal expansion characteristics. Invar (UNS K93600), consisting of 36% Ni and the rest Fe, is distinctive for possessing an almost negligible coefficient of thermal expansion near room temperature. This renders it important where high dimensional stability is necessary, like in precision measuring devices and thermostat rods. It is additionally utilized at cryogenic temperatures due to its extremely low thermal expansion rates. Alloys with 72–83% Ni possess optimal soft magnetic characteristics and are utilized in transformers, inductors, magnetic amplifiers, magnetic shielding, and memory storage devices [29].

Alloy 36 (Invar K93600) is a Ni-Fe alloy with low expansion also in low temperatures of 250 °C. It exhibits good strength and toughness at very low temperatures. Such an alloy can be used for tanks for liquid natural gas, measuring and thermostatic instruments [20].

The recommended heat treatment parameters for Alloy 36 are the following [31]:

The annealing must take place at temperatures ranging from 820 to 900 °C, followed by cooling in air. In contrast to air cooling, water cooling after annealing yields a reduced heat expansion coefficient. Nevertheless, the obtained microstructure lacks stability. After cold forming of under 10%, the annealing temperature must not go beyond 860 °C. Stress relief annealing is conducted at temperatures around. 700 degrees Celsius. The minimum thermal expansion values at 100 °C are obtained through a three-step heat treatment process: About. 30 min of heat treatment at 830 °C followed by rapid cooling in water. Heating to 300 °C; holding the temperature for 1 h; cooling in air. Heating again to 100 °C; holding the temperature for 30 min; cooling in the furnace to room temperature for 48 h. The material needs to be inserted into a furnace that has reached the highest annealing temperature prior to any heat treatment. For strips and wires as the product shape, the heat treatment may be conducted in a continuous furnace at a speed and temperature suited to the geometry.

**Ni-Cr-Fe alloys**

Ni-Cr-Fe (Ni-Cr-Fe) alloys belong to two categories [29]:

- Ni-Cr-Fe alloys exhibit remarkable strength at elevated temperatures and possess the capability to withstand oxidation, carburization, and various forms of high-temperature corrosion. The most recognized is alloy 800 (UNS N08800), along with its variants 800H (UNS N08810) and 800HT (UNS N08811). (Recently, such alloys have been categorized as stainless steels due to their elevated Fe content accompanied mainly by Ni and Cr). Stainless steel 800H exhibits exceptional high-temperature strength and high resistance to high-temperature oxidation.
- Ni-Cr-Fe (along with Mo and Cu) alloys that offer outstanding corrosion resistance in certain applications. Arguably, the most recognized is alloy 825 (UNS N08825), known for its high resistance to sulphuric acid. Alloy G3 (UNS N06985) provides high corrosion resistance against commercial phosphoric acids and various complex solutions with strong oxidizing acids.

Table 16 presents the chemical composition of certain Ni-Cr-Fe alloys.

Various amounts of Ni are present in certain stainless steels. The latter belong into three major classes [32]:

1. Cr (11.5–17%)-Fe alloys with carefully controlled carbon content. Can be heat-treated to a magnetic martensite structure and are therefore known as martensitic stainless steels.
2. Cr (17–27%)-Fe alloys with low carbon content. They are non-hardenable by heat treatment. Their crystal structure is magnetic ferrite and therefore are known as ferritic stainless steels.
3. Cr (16–26%)-Ni (6–22%)-Fe alloys with low carbon content. They are non-hardenable by heat-treatment. They exhibit crystal structure of nonmagnetic austenite and are therefore called austenitic stainless steels.

Table 17 presents the composition and some basic properties of various types of stainless steel [32]. Since no standard has been found for specifying samples for testing various Ni-Based alloys, samples related to stainless steel can be used.

Inconel (tm) is a specialized alloy that incorporates greater amounts of Ni and Cr compared to stainless steel, along with several other elements in minimal amounts. It is, in fact, a trademark of Inco Alloys International and belongs to a category of metals referred to as Ni-based super alloys. These minor additions of different elements constitute solid-solution hardening. It is rather costly and is typically reserved for situations where some form of stainless steel is inadequate. A notable feature of high-Ni alloys, such as Inconel (tm), is their excellent resistance to numerous corrosive substances. Except for a few cases, high-Ni alloys generally perform much better than martensitic, ferritic, and austenitic stainless steels in corrosive conditions. Table 18, Table 19 and Table 20 show components and the properties of Inconels (tm) [32]. Table 21 presents high-temperature strength and physical properties of certain Inconel alloys. They can be useful during the modeling of the welding processes.

Ni-Cr-Fe alloys demonstrate excellent strength at elevated temperatures and are resistant to oxidation, carburization, and various forms of corrosion [29].

The inclusion of Cr in Ni-Cr-Fe alloys forms a surface layer that enhances resistance to corrosion and oxidation [40].

Symons [41] studied the effect of Cr on the hydrogen embrittlement of Ni-Cr-Fe alloys with Cr levels between 6% and 35% wt. In the uncharged state, ductility, assessed by the percentage of elongation or reduction in area, rose with an increase in Cr content of the alloy. H_2_ exerted only a slight influence on the mechanical characteristics of the low-Cr alloys. The inclusion of hydrogen significantly influenced the ductility of the alloys with higher Cr content. In the 26% Cr alloy, the elongation at failure decreased from 53% to 14%, accompanied by a shift in fracture mode from a combination of ductile dimple and ductile intergranular failure to a brittle-like intergranular failure. The highest level of embrittlement was noted in the 26% Cr alloy. The peak in embrittlement coincided with the lowest point in stacking-fault energy. The heightened hydrogen embrittlement in the high-Cr alloys resulted from enhanced slip planarity attributed to decreased stacking-fault energy. Slip planarity did not influence the breakage of the uncharged samples

Ni-Cr-Fe alloys are used in bearings, castings, ballast, step soldering, radiation protection, household appliances, and general heating devices [29].

The physical and mechanical properties do not vary significantly between alloy 800, alloy 800H, and alloy 800HT, especially at temperatures below 650 °C [38].

Recommended heat treatment parameters for alloy 600 are the following [34]:

- Annealing—softens the material. Proper treatment takes 15 min in about 1010 °C.
- Solutioning—dissolves the carbides and increases grain size, which is good for creep resistance and rupture strength. Proper treatment takes 1 to 2 h at 1090–1150 °C.

Recommended heat treatment parameters for alloy 601 are the following [35]:

- Solution-treatment: 1100–1200 °C.
- Annealing: 1000–1100 °C.

During solution treatment or annealing, the piece needs to be held in the furnace for 30–60 min and then be water quenched or rapid air cooled (continuous annealing) or held for 60–180 min and air-cooled (batch annealing).

Recommended heat treatment parameters for Alloy 625 are the following [36]:

- Soft annealing: 980–1150 °C; 30–60 min.
- Solutioning: 1150 °C; 2 h.

There are multiple heat treatments for alloy 718 [37].

The first one described in AMS 5662 allows the treated piece to achieve the optimum stress-rupture life, notch-rupture life, rupture ductility, and tensile strength. The procedure comprises:

- Annealing: 925–1010 °C; 1 h.
- Air cooling.
- Age hardening: 620 °C; 8 h.
- Furnace cool to 650 °C.
- Hold at 650 °C until furnace time for the entire age-hardening cycle equals 18 h.
- Air cooling.

The second one described in AMS 5664 is preferred in tensile-limited applications because it promotes the best transverse ductility in heavy sections, impact strength, and low-temperature notch tensile strength, at the cost of notch brittleness in stress rupture. The procedure comprises:

- Annealing: 1035–1065 °C; 1 h.
- Air cooling.
- Age hardening: 760 °C; 10 h.
- Furnace cool to 650 °C.
- Hold at 650 °C until furnace time for the entire age-hardening cycle equals 20 h.
- Air cooling.

The third one, given in NACE MR0175/ISO 15156-3, describe the following heat treatment for the best sour gas applications (oil/gas drilling equipment):

- Annealing: 1010 °C; 2 h.
- Water Quenching.
- Age hardening: 780–800 °C; 6–8 h.
- Air cooling.

Annealing of HASTELLOY(r) G-3 alloy can be conducted at 1177 °C followed by rapid cooling of air and water quenching [42].

**Ni-Cr-Mo-W alloys**

Composition of certain Ni-Cr-Mo-W alloys is presented in Table 22. Physical and mechanical properties of such alloys are shown in Table 23. Table 24 presents high-temperature strength and physical properties of certain Ni-Cr-Mo-W-based alloys. They can be useful during the modeling of the welding processes.

Incoloy 825 demonstrates outstanding resistance to phosphoric and sulfuric acids. The alloy is resistant to a broad spectrum of corrosive conditions and intergranular sensitization. It is resistant to oxidizing and reducing acids, stress corrosion cracking, pitting, and intergranular corrosion, making it suitable for chemical and petrochemical processing, oil and gas extraction, pollution management, waste treatment, and pickling uses. Alloy 825 is utilized in phosphoric acid evaporators, pickling machinery, vessels and piping for chemical processing, equipment for recovering spent nuclear fuel, propeller shafts, and tank trucks [43].

Incoloy 925 (N0925) is a corrosion-resistant, high-strength Ni-Cr-Fe alloy that is PS, featuring additions of Ti, Al, Mo, and Cu. It can be considered an enhanced variant of alloy 825 that has similar corrosion resistance but offers superior strength characteristics due to precipitation hardening. Alloy 925 is mainly utilized in the oil and gas sector for drilling and surface gas well parts, such as piping, valves, and fasteners. It is additionally utilized in the maritime sector [44].

Incoloy 926 (UNS N08926/W.Nr. 1.4529) is an austenitic stainless-steel alloy that shares a comparable chemical composition with the 904 L alloy and exhibits enhanced corrosion resistance against various aggressive environments. Its N concentration is 0.2%, while its Mo concentration is 6.5%. The resistance to pitting and crevice corrosion in halide media was significantly enhanced by the presence of Mo and N. Ni and N not only guarantee the stability of metallographic structures but also more effectively reduce the tendency for intergranular separation during thermal or welding processes compared to the N levels in Ni alloys. Cu enhances tolerance to sulfuric acid, while nitrogen improves yield and tensile strength. It is relevant in multiple systems such as fire protection, water treatment, marine engineering, and hydraulic piping flow, as well as in the elements utilized in the acidic gas and phosphate manufacturing. It can also be utilized in power generation facilities for cooling sewage water in condensation and piping systems, as well as for producing acidic organic catalyst chlorinated derivatives, cellulose pulp, polished bars in corrosive oil wells, hose systems in ocean engineering, components of flue gas desulfurization systems, sulfuric acid condensation and separation systems, transportation of corrosive chemical containers, and reverse osmosis desalination plants [45].

Nimonic 86 is a Ni-Cr-Mo alloy that forms a solid solution and includes magnesium and cerium as alloying elements. It exhibits outstanding resistance to cyclic oxidation at 1050 °C, is ductile, suitable for welding, and resistant to creep. Nimonic 86 is utilized in aerospace (gas turbine combustion chambers, afterburner components) and thermal processing (heat-treatment furnace machinery) [46].

Nimonic 90 (UNS N07090) is a wrought alloy of Ni, Cr, and cobalt that has been age-hardened, incorporating Ti and Al as alloying elements. It can withstand heat and creep up to 920 °C. It is utilized for gas turbine parts (blades, disks, forgings, rings), within the automotive sector, for hot-working tool parts, and for springs that operate at high temperatures [47].

Alloy C276, commonly referred to as N10276 or Hastelloy C276, is a Ni-Cr-Mo alloy with Ni as its main component. It possesses remarkable resistance to heat and corrosion. Alloy C276 is utilized in chemical processing, pollution management, pulp and paper manufacturing, industrial and municipal waste treatment, as well as the retrieval of sour natural gas. Its uses encompass stack liners, ducts, dampers, scrubbers, stack-gas reheaters, fans, fan enclosures, heat exchangers, reaction vessels, evaporators, and transfer pipes [48].

Hasteloy C4 is a ternary alloy composed of Ni, Cr, and Mo that is low-carbon, low-Si, and free of W, with Ti added to stabilize leftover carbon. It exhibits remarkably high-temperature stability, exceptional resistance to harsh aqueous conditions, and satisfactory weldability. It can be viewed as a more stable and superior weldable version of Alloy C276. Alloy C4 exhibits favorable ductility and corrosion resistance following extensive aging at temperatures ranging from 650 to 1040 °C. It shows great resistance to stress-corrosion cracking and oxidizing environments up to 1040 °C [49].

Alloy 825 is usually subjected to heat treatment via annealing, commonly at temperatures ranging from 920 to 980 °C, with an optimal temperature of 940 ± 10 °C. This method aids in obtaining a soft and stable configuration, while quick water quenching can enhance corrosion resistance [52,53].

Recommended heat treatment parameters for Alloy 925 are the following [44]:

▪ Solution-treatment: 980–1040 °C 0.5–4 h; for sizes up to 25 mm WQ or AC, for sizes above 25 mm WQ.
▪ Age-hardening: 730–750 °C for 8 h; Furnace cooling: 55 °C/h for 2 h; Age-hardening: 610–630 °C for 8 h; AC or WQ.

Heat treatment for Alloy 800H and Alloy 800HT include [38]:

▪ Precipitation temperature: 760 °C.
▪ Batch annealing in bell-type furnaces: Cool rapidly through the range between 760 and 540 °C to ensure freedom from sensitization. Alloys 800H and 800HT are not susceptible to thermal cracking.

Heat treatment for sheets made of Nimonic 86 can be realized in two variants [46]:

- The first: 1150 °C for 10 min, air cooling.
- The second: 1150 °C for 2–4 h, air cooling.

Nimonic 86 undergoes precipitation hardening followed by recrystallization when exposed to 500–600 °C for long time periods. Cold working accelerates the process and extends the temperature range to 450–700 °C. Contrary, rapid cooling after annealing enhances thermal stability by prolonging the time needed for hardening to occur.

Recommended heat treatment parameters for C-276 alloy are the following [48]:

- Solution annealing: 1107–1135 °C.

Recommended working and heat treatment parameters for Hastelloy C4 are the following [49]:

- Soft annealing: 1065 ± 14 °C; 30 min; WQ.

Annealing involves heating to 1066 °C and water quenching. A hold time at the annealing temperature of 10 to 30 min is recommended, with thicker structures requiring the full 30 min.

**Ni-Fe-Cr-Mo alloys**

Composition of certain Ni-Fe-Cr-W alloys is presented in Table 25. Physical and mechanical properties of such alloys are shown in Table 26. Table 27 presents high-temperature strength and physical properties of certain Fi-Cr-Fe-Mo-based alloys. They can be useful during the modeling of the welding processes.

Alloy HX/2.4665 exhibits an excellent combination of oxidation resistance, fabricability and high-temperatures strength. This alloy can be used for components for gas turbines and industrial furnaces.

Alloy C22 (N06022) is a Ni-Cr-Mo alloy created in 1985 to provide improved thermal stability compared to Alloy C276 and enhanced resistance to chloride-induced localized corrosion and stress-corrosion cracking over Alloy C4. Recognizing that Mo and W play opposing roles compared to Cr in reducing versus oxidizing acidic settings, Alloy C22 was created with the right balance of these elements to ensure versatility across various corrosive conditions. Alloy C22 is utilized in the production of acetic acid, manufacturing of cellophane, systems for chlorination, intricate acid mixtures, electro-galvanizing rollers, expansion bellows, flue gas scrubbing systems, systems for HF scrubbing, geothermal wells, incineration scrubber systems, reprocessing of nuclear fuel, production of pesticides, creation of phosphoric acid, systems for pickling, plate heat exchangers, selective leaching systems, cooling towers for SO_2_, sulfonation systems, tubular heat exchangers, and valve weld overlays [54].

Alloy 59 (UNS N08031) is a corrosion-resistant Ni-Cr-Mo alloy characterized by extremely low levels of carbon and silica. It can be regarded as a modification of alloys C-2000 and C-4, created explicitly for harsh environments in flue gas desulfurization systems. It exhibits superior thermal stability compared to C2000 and C4, and it achieved NACE MR 0175/ISO 15156 Level VII resistance, making this alloy quite appealing for the Oil & Gas sector. It is approved for the transportation of dangerous materials. It is primarily utilized in chemical processing and pollution management. Common uses include scrubbers, heat exchangers, ventilators, and agitators for flue gas desulfurization in fossil fuel power stations and waste combustion facilities, SO_2_-washers for marine diesel engines, reactors for acetic acid, acetic anhydrides, hydrofluoric acid, coolers for sulfuric acid, and pipes in geothermal energy plants [55].

Alloy 2000 (N06200, Hastelloy C2000) is a Ni-Cr-Mo alloy that includes Cu as an additional alloying element. It can be regarded as an enhanced variant of C-22, exhibiting overall superior resistance to both reducing and oxidizing conditions. Ductile, simple to weld, resistant to pitting, crevice attack, and stress corrosion cracking, it stands out as one of the most adaptable alloys utilized in chemical processing. The blend of approximately 16% Mo and around 1.6% Cu offers protection against reducing agents (such as dilute hydrochloric or sulfuric acids), while about 23% Cr content ensures strong resistance to oxidizing acids. It is used in the components of chemical processing plants—such as reactors, heat exchangers, valves, and pumps—for environments that are both oxidizing and reducing [56].

Alloy 686 (UNS N06686) is a single-phase, austenitic Ni-Cr-Mo superalloy enhanced with W. It withstands very aggressive oxidizing (due to high Cr) and reducing (Ni and Mo) conditions. Due to its minimal Fe and C levels, along with the combination of Mo and W, it provides excellent resistance to localized corrosion, like pitting and crevice. Low C preserves corrosion resistance in the heat-affected areas of welded joints. It is utilized in chemical processes, pulp production, paper production, pollution management, and waste disposal applications [57].

Recommended heat treatment parameters for Hastelloy C-22 are as follows [54]:

- Solution-treatment: 1120 ± 14 °C, 30 min, WQ.

Annealing involves heating to 1120 °C and water quenching (thin pieces can be rapid air cooled). A hold time at the annealing temperature of 10 to 30 min is recommended, with thicker structures requiring the full 30 min.

Recommended heat treatment parameters for Haynes 59 are the following [55]:

- Solution-treatment: 1100–1180 °C; rapid air cool/water quench.

For best corrosion resistance, the component should be rapidly cooled (>150 °C/min) from solutioning temperature down to 500 °C.

Recommended heat treatment parameters for Hastelloy C2000 are as follows [56]:

- Solution-treatment: 1135–1163 °C; 30 min; water quench.

Solution heat treatment involves holding in ~1149 °C and water quenching (<10 mm thin pieces can be rapid air cooled). A hold time at the annealing temperature of 10 to 30 min is recommended, with thicker structures requiring the full 30 min.

Recommended heat treatment parameters for Alloy 686 are the following [57]:

- Annealing: 1180–1200 °C; water quench.

**Ni-Cr-Co-Mo alloys**

The composition of certain Ni-Cr-Co-Mo alloys is presented in Table 28. Physical and mechanical properties of such alloys are shown in Table 29. Table 30 presents the physical and mechanical properties of Inconel 617 alloy. They can be useful during the modeling of the welding processes.

Alloy 617 (UNS N06617/W.Nr. 2.4663a) is a SSS Ni-Cr-Co-Mo alloy known for its strength at elevated temperatures, excellent creep resistance, and oxidation durability. It also possesses strong resistance to environments with high-temperature carburizing and nitriding. It is utilized for gas turbines, petrochemical and thermal processing, as well as for the production of nitric acid [58].

Recommended heat treatment parameters for Inconel 617 are as follows [58]:

- Continuous soft-annealing: 1120–1175 °C; 30–60 min; air cool/water quench
- Batch soft-annealing in bell-type furnaces: 1120–1175 °C; 1–3 h; air cool
- Intermediate annealing: 1040 °C
- Solution-treatment according to ASME SB 176: 1140–1232 °C—to achieve an austenitic matrix without carbide precipitates.

Dong et al. [59] investigated the microstructure analysis and creep enhancement characteristics of a non-γ′ phase Ni-based high-temperature alloy C-HRA-2. M_23_C_6_ carbides, measuring approximately 10–50 nm in diameter, precipitate and disperse throughout the grains following creep. The creep-rupture strength of alloy C-HRA-2 at 650 °C after 10,000 h is approximately 222 MPa, while after 100,000 h, it is anticipated to be around 170 MPa. The creep-rupture of alloy C-HRA-2 results from the formation and expansion of voids at the trigeminal and transverse grain boundaries. The formation of voids results from the movement of grain boundaries and the deposition of large M_23_C_6_ particles at the grain boundaries. The contribution of M_23_C_6_ to creep strength is increased when the γ′ phase is absent. The M_23_C_6_ phase enhances creep strength by obstructing dislocation movement and sliding along slip planes.

Chen et al. [60] examined the solidification process and precipitation mechanism of the C-HRA-3 Ni-Cr-Co-Mo-based heat-resistant alloy. The findings verify that the main solidification precipitates in the core of the C-HRA-3 alloy VAR ingot are made up of Ti(C,N), M6C-M_23_C_6_ symbiotic phases, Ti(C,N)-M6C-M_23_C_6_ symbiotic phases, along with surrounding dispersed M6C and M_23_C_6_ carbides. Effectively decreasing the cooling rate during the phase above the precipitation temperature and raising the cooling rate below that temperature can greatly diminish the size and volume fraction of the symbiotic phases.

#### 2.1.3. PS Ni-Based Alloys

While precipitation strengthening is not exclusive to these alloys, it is noteworthy that a considerable amount of this strength is preserved at elevated temperatures. The precipitation process relies on the restricted solubility of alloying elements (typically Ti, Al, Nb) within the Ni matrix. The intermetallic compounds, referred to as precipitates, arise from supersaturated solid-solution during thermal processing. They prevent the movement of dislocations, thereby enhancing matrix strength. The PS alloys may also include elements that enhance solid-solution strengthening (Cr, Co, Fe, Mo, W, and Ta), improve oxidation and hot-corrosion resistance (Cr, Ta, La, Th), and provide creep resistance (B, Zr). The tensile strength of PS alloys varies between 830 MPa and 1590 MPa, whereas their yield strength spans from 585 MPa to 1249 MPa. The key mechanical property for this category of alloys is frequently a 1000 h rupture stress value. Table 31 displays the fundamental mechanical characteristics of the chosen PS alloys. The industrial uses of PS Ni-based alloys are mainly designed for high-temperature applications, like jet engine turbine blades. Alloys K-500 and 80A have been utilized in the chemical and oil and gas sectors because of their outstanding resistance to corrosion and oxidation [14,15,16].

Table 32 shows the chemical composition of certain PS Ni-based alloys.

**Ni-Cu-Al-Ti alloys**

K-500 Alloy K-500, often called “K-MONEL”, is a Ni-Cu alloy that is hardenable through precipitation. Its corrosion resistance is comparable to that of alloy 400, with the added benefit of increased strength and hardness. The incorporation of Al and Ti into the Ni-Cu alloy enables a subsequent heat treatment that enhances the mechanical properties. Alloy K-500 exhibits low magnetic permeability and robust non-magnetic properties across a broad temperature spectrum, even at subzero levels.

Alloy K-500 is commonly used in the marine, chemical processing, oil and gas, pulp and paper, pharmaceutical, food processing, and electronics sectors. Applications for the end use of alloy K-500 encompass fasteners, springs, chains, components for pumps and valves, drill collars, doctor blades, scrapers, mixing shafts, impellers, sensors, electrical parts, and various other highly corrosive uses where both strength and hardness matter.

The corrosion resistance of alloy K-500 is comparable to that of alloy 400. Nonetheless, in its age-hardened state, alloy K-500 may undergo stress corrosion cracking in specific environments. The resistance of alloy K-500 to H_2_S renders it valuable in sour gas settings, making it a perfect option for use in the oil industry. The minimal corrosion rates in seawater render alloy K-500 ideal for applications in the marine sector. Pitting can happen in stagnant or slow-moving seawaters, but the pitting rate eventually diminishes after the initial onset [61]. Table 33 shows high-temperature strength and physical properties of K500 [18].

For this alloy, solution annealing of hot-finished products is performed at 980 °C, and of cold-worked ones at 1040 °C. To avoid excessive grain growth, the time at temperature should be kept under 30 min. Both heating and cooling should be conducted as fast as possible [18].

Intermediate annealing may be required to soften the product during the production of the forming process. It is usually performed at temperatures between 760 and 870 °C for 1 h [18].

The age hardening procedure is the following for [18]:

▪ Soft material (140–180 HBW)—as-forged and quenched or annealed forgings, annealed or hot-rolled rods, large cold-drawn rods, and soft-temper wire and strip:
  o Age hardening: 580–610 °C; 16 h.
  o Furnace cooling: 12 °C/h down to 480 °C.
  o Air cooling.
▪ Moderately cold-worked material—cold-drawn rods, half-hard strip, cold-upset pieces and intermediate-temper wire:
  o Age hardening: 580–610 °C; 8 h.
  o Furnace cooling: 12 °C/h down to 480 °C.
  o Air cooling.
▪ Fully cold-worked material (260–325 HBW, 25–35 HC)—spring-temper strip, spring wire or heavily cold-worked pieces such as small, cold-formed balls:
  o Age hardening: 520–540 °C; 6 h.
  o Furnace cooling: 12 °C/h down to 480 °C.
  o Air cooling.

**Ni-Cr-Al-Ti**

Alloy 80A (UNS N07080) is a Ni-Cr alloy containing Ti and Al, which is age-hardened, heat-resistant, creep-resistant, and provides resistance to S in combustion gases. It operates at temperatures up to 815 °C. It finds application in the automotive industry for exhaust valves, in aerospace for fasteners, gas turbine blades, rings, and disks, and in nuclear power facilities for boiler tube supports [62].

Recommended heat treatment parameters for Alloy 80A to achieve high stress-rupture strength are the following:

Heat treatment for extruded bars:

Solution-treatment: 1080 °C 8 h; AC.

Age-hardening: 700 °C 16 h; AC.

Heat treatment for extruded bars, subsequently cold stretched:

Solution-treatment: 1080 °C 8 h; AC.

Stabilizing anneal: 850 °C 24 h; AC.

Age-hardening: 700 °C 16 h; AC.

Heat treatment for cold-rolled sheet:

Solution-treatment: 1150 °C 2–3 min; fluidized bed quench

Solution-treatment: 1040 °C 20 min; AC.

Age-hardening: 750 °C 4 h; AC.

Heat treatment for welded sheet:

Solution-treatment: 1150 °C 2–3 min; AC.

Weld.

Post-weld heat treatment: 925 °C 1 h; AC.

Age-hardening: 750 °C 4 h; AC.

Alloy 90 (UNS N07090) is a Ni-Cr-Co alloy that is wrought and age-hardened, with additions of Ti and Al as alloying elements. It is resistant to heat and creep up to 920 °C. It is utilized for components in gas turbines (blades, disks, forgings, rings), in the automotive sector, for hot-working tool parts, and for high-temperature springs [47].

Recommended procedure for heat treatment of rods and profiles made of Alloy 90 after cold and/or hot working are the following:

Annealing: 1150 °C/8 h/air cooling.

Precipitation hardening: 700 °C/16 h/air cooling.

Recommended procedure for cold plastic working and heat treatment of sheets:

Annealing: 1150 °C/2–3 min/bath cooling.

Cold working.

(Welding).

Recrystallization annealing: 1040 °C/20 min/air cooling.

Precipitation hardening: 750 °C/4 h/air cooling.

Alloy C263 (UNS N07263) is a Ni-Cr-Co-Mo alloy that is resistant to creep, PS, and designed for high temperatures. It is provided in the annealed state. It is suggested for service temperatures reaching 850 °C, though it can withstand oxidation up to 1000 °C. It welds easily (showing no tendency for cracking after weld heat treatment), possesses favorable fabrication properties, offers excellent wear resistance, and does not demonstrate tensile ductility at intermediate temperatures. It is utilized in aerospace and industrial gas turbines for combustion chambers at low temperatures, transition liners, and exhaust cones and rings [63].

Recommended heat treatment parameters for Alloy C263 are the following:

Solution-treatment: 1140–1160 °C; rapid air cool/water quench.

Age-hardening: 785–815 °C; 8 h; air cool.

Intermediate annealing: 1070–1090 °C; 4–6 min; air cool.

Homogenizing (treatment): 1150 °C; 1 h; air cool.

**Ni-Fe-Cr-Nb-Al-Ti**

Haynes 282 is an alloy designed for structural uses at elevated temperatures, demonstrating superior creep strength within the range of 650–930 °C, outperforming both Waspalloy and Rene 41. Haynes 282 features a distinctive blend of creep strength, thermal stability, weldability, and fabricability that is absent in presently available commercial alloys. Its characteristics render it appropriate for essential gas turbine uses, including combustors, turbine and exhaust parts, and nozzle elements [64].

Inconel 713 is a Ni alloy that excels in high-temperature environments and under severe mechanical stress. A key benefit is its capacity to sustain stability at high temperatures while providing outstanding creep resistance. It retains mechanical strength up to 980 °C. Inconel 713 displays excellent resistance to distortion at elevated temperatures. It demonstrates outstanding resistance to oxidation. It is perfect for gas turbines and engine parts that function at elevated temperatures. It is not as appropriate for marine settings as Inconel 625 but provides better thermal stability. It is commonly favored for parts subjected to elevated temperatures, like in aerospace, gas turbine, and energy industries. Its appropriateness for casting applications provides a major benefit in manufacturing intricate engine components [72].

Waspaloy is a Ni alloy that hardens with age, designed for use in gas turbine applications. Resistant to creep and heat, it is utilized in combustion environments up to 870 °C. Nevertheless, in crucial and demanding applications, the highest operating temperature must not surpass 650 °C. This is used for gas turbine parts in the aerospace sector, encompassing compressor disks, rotor disks, shafts, spacers, seals, rings, casings, fasteners, and more [66].

Recommended heat-treatment parameters for Waspalloy are as follows:

Solution-treatment: 1066–1093 °C; rapid air cool/water quench.

Age hardening I: 996 °C; 2 h; air cool.

Age hardening II: 843 °C; 4 h; air cool.

Age hardening III: 760 °C; 16 h; air cool.

René 41 (UNS N07041) is a superalloy consisting of Ni-Cr-Co-Mo, known for its age-hardening and creep resistance, with Al and Ti as alloying elements. It is suitable for components in jet engines and high-speed airframes, including fasteners, wheels, turbine casings, and afterburner parts [67].

Recommended heat treatment parameters for René 41 are the following:

Solution-treatment: 1040–1050 °C; 4 h; AC (for max tensile ductility).

Solution-treatment: 1080–1090 °C; 1 h; AC (for max creep-resistance).

Age-hardening: 760 °C; 16 h; AC.

For cast René 41, heat treatment comprises:

Solution-treatment: 1065 °C; 3 h; AC.

Solution-treatment: 1120 °C; 30 min; AC.

Age-hardening: 900 °C; 4 h; AC.

Inconel 725 Alloy 725 (N07725) is a PS Ni-Cr-Fe-Mo alloy that contains Nb as an alloying element. It can be viewed as an age-hardenable version of alloy 625, offering similar corrosion resistance but greater strength. It withstands ordinary corrosion, pitting, sulfide stress cracking, hydrogen embrittlement, and stress-corrosion cracking in sour gas wells. It can be utilized for hangers, landing nipples, side pocket mandrels, polished bore receptacles in sour gas operations, high-strength fasteners in marine use, and polymer extrusion dies [68].

For the Inconel 725 solution heat-treatment involves holding at 1040 °C and air cooling. A higher temperature of 1066 °C can be chosen as post-welding heat treatment for the highest impact strength. Overall, however, 1040 °C is preferable.

Solutioned parts should be age-hardened. There are multiple age hardening procedures, but for sour gas applications, the recommended procedure comprises:

Age hardening: 730 °C; 8 h.

Furnace cooling: 56 °C/h.

Age hardening: 620 °C 8 h.

Air cooling.

Inconel 706 (UNS 09706) is a PS alloy, offering excellent mechanical strength along with good workability. The properties of the alloy resemble those of Inconel alloy 718, except that alloy 706 is easier to fabricate, especially through machining. The significant amounts of Ni and Cr offer excellent protection against oxidation and corrosion. The main components responsible for precipitation hardening in the alloy are Nb and Ti. The Al levels additionally play a role in the hardening reaction. The precipitation-hardening mechanism in Inconel alloy 706 offers the advantageous feature of a postponed hardening reaction when subjected to precipitation temperatures. This trait provides the alloy with outstanding resilience against postweld strain-age cracking. Inconel alloy 706 is utilized in numerous applications that demand high strength along with easy fabrication. In the aerospace sector, the alloy is utilized for turbine disks, shafts, and housings; diffuser housings; compressor disks and shafts; engine supports; and connectors. Besides aerospace uses, the alloy is utilized for turbine disks in major industrial gas turbines [69].

Incoloy alloy 909 (UNS N19909) is an alloy composed of Ni, Fe, and cobalt, known for its notable features, including a consistently low coefficient of thermal expansion, a stable modulus of elasticity, and impressive strength. The alloy is reinforced through a precipitation-hardening heat treatment enabled by the inclusion of niobium and Ti. The merging of low expansion with high strength renders Incoloy alloy 909 particularly advantageous for gas turbines. The reduced expansion allows for tighter regulation of clearances and tolerances, resulting in improved power output and fuel efficiency. The increased strength enhances strength-to-weight ratios, resulting in lighter aircraft engines. Due to these factors, Incoloy alloy 909 is utilized for vanes, casings, shafts, and shrouds in gas turbines. The characteristics of Incoloy alloy 909 are appealing for use in rocket-engine thrust chambers, ordnance components, springs, steam-turbine bolts, gauge blocks, instrumentation, and glass-sealing applications [70].

Incoloy alloy 945 (UNS N09945) is a robust, corrosion-resistant material intended for rigorous applications in the oil and gas sector. The characteristics of this age-hardenable Ni-Fe-Cr alloy are improved by the presence of Mo, Cu, Ni, Ti, and Al. The Ni content of the alloy leads to its resistance against stress corrosion cracking induced by chlorides. Ni, along with Mo and Cu, also provides alloy 945 with exceptional resistance to general corrosion in reducing environments. Mo likewise provides resistance to localized damage (such as pitting and crevice corrosion). The Cr present in the alloy offers protection in oxidizing conditions. Nb, Ti, and Al take part in the age hardening process that happens during heat treatment. Their interaction creates gamma prime and gamma double prime precipitates, which enhance the alloy’s strength. Incoloy alloy 945 is ideal for downhole oil and gas applications that demand high strength and resistance to corrosion in harsh sour wells with elevated levels of H_2_S and HCl. Due to its resistance to stress cracking in H_2_S-rich environments, the alloy is appropriate for gas-well components both downhole and on the surface, such as tubular products, hangers, valves, landing nipples, tool joints, and packers. The alloy can additionally be utilized for fasteners, pump shafts, and robust piping systems. Traditional annealed and aged alloy 945 materials demonstrate a minimum yield strength of 125 ksi. A high-strength grade of the alloy, known as Incoloy alloy 945X, shows a minimum yield strength of 140 ksi. Alloy 945X also possesses greater strength compared to alloys 718, 725, and 925. Cold-worked and specifically aged products are offered with even greater strength for uses like shafting [71].

Zhang et al. [73] indicated that the shape and volume ratio of precipitates are essential for the mechanical characteristics of PS high-entropy alloys (HEAs). They investigated how Ni and Al influence the precipitation behavior and mechanical characteristics of (FeCoCr) 98-x-y NixTi2Aly (x = 25, 30, 35; y = 4, 6, 8) HEAs. It was discovered that a rise in Ni and Al can significantly enhance the development of coherent γ′ precipitates, but an incoherent Heusler phase (Ni2AlTi) will also unavoidably emerge once the Al concentration surpasses 6%. Both γ′ and the Heusler phases have the potential to enhance the materials, but due to their common constituent elements, the two phases created synergistic effects. For example, an overload of the Heusler phase not only hinders the development of γ′ but also diminishes ductility. The Ni to Al ratio significantly influenced the formation of the Heusler phase, and it could be entirely removed when the Ni/Al ratio exceeds 5. In this scenario, the highest volume fraction of γ′ in (FeCoCr)57Ni35Ti2Al6 attains 33%, and the alloy displays a tensile strength exceeding 1 GPa along with a reasonable ductility of approximately 35%.

To summarize, the application of various CP Ni, its SSS alloys and its PS alloys is presented in Table 34.

#### 2.1.4. The Specialty Ni-Based Alloys

The specialized Ni-based alloys can be categorized into two distinct subgroups—those reinforced by dispersed oxide (Oxide Dispersion Strengthened—ODS) and those reinforced by Ni aluminides. The production of ODS alloys relies on an advanced mechanical alloying technique that combines various metal and oxide powders, whereas Ni aluminides are derived from Ni_3_Al or NiAl intermetallic compounds. The specialized Ni-based alloys typically exhibit excellent corrosion and creep resistance when subjected to high temperatures. These alloys possess greater specific strength than other Ni alloys due to their reduced density. The primary disadvantage of specialty Ni-based alloys is their limited ductility and toughness. Common uses are found in the aerospace sector for components utilized in turbojet engine manufacturing [3].

**Ni-aluminides**

Ni Al (Ni–Al) intermetallics featuring different lattice structures are versatile materials that serve as next-generation superalloys, catalysts, and even coating substances. The Ni_10_Al_90_, Ni_20_Al_80_, and Ni_35_Al_65_ alloys, associated with the formation of the quasi-crystalline phase (Q-phase) linked to Al_3_Ni, Al_3_Ni_2_, and NiAl intermetallics, exhibit fewer icosahedral-like clusters in the liquids when compared to the other two face-centered cubic (FCC) forming alloys (Ni_2_Al_98_ and Ni_74.5_Al_24.5_). A robust interconnection of central atoms within the icosahedral-like clusters was noted in the three liquids related to Q-phases, fostering the formation of intermetallics. The pre-peaks observed in the total X-ray structure factors are closely connected to the Al_3_Ni, Al_3_Ni_2_, and NiAl intermetallics regarding the bond lengths. FCC forming liquids, whether on the high Al side or the high Ni side, did not exhibit any pre-peak phenomenon [74].

Jóźwik et al. [75] examined existing and potential uses of Ni_3_Al-based intermetallic alloys that may be beneficial for structural and functional applications. The primary parts produced from these materials are designed chiefly for forging dies, furnace assemblies, turbocharger parts, valves, and the piston heads of internal combustion engines. The Ni_3_Al-based alloys created through directional solidification are also regarded as a material for making turbine blades in jet engines. Additionally, the creation of composite materials utilizing Ni_3_Al-based alloys as a matrix reinforced by TiC, ZrO_2_, WC, SiC, and graphene is also documented. Owing to their unique physical and chemical characteristics, it is anticipated that these materials, when shaped into thin foils and strips, will greatly enhance the production of advanced technology devices such as Micro Electro-Mechanical Systems (MEMS) or Microtechnology-based Energy and Chemical Systems (MECS), along with heat exchangers, microreactors, micro-actuators, parts of combustion chambers, and gaskets in rocket and jet engines, as well as elements of high specific strength systems. Moreover, their catalytic characteristics might be utilized in catalytic converters, air purification systems to eliminate chemical and biological toxins, or in the production of H_2_ through HC decomposition.

Meng et al. [76] introduced an innovative technique to address the challenge faced in wire arc additive manufacturing of Ni-Al intermetallic compounds (IMCs), specifically the absence of welding wire due to the brittle fracture occurring during wire drawing. A hybrid manufacturing process combining synchronous wire and powder was utilized, using Ni wire as the forming material and Al powder as auxiliary filler. Utilizing an optimized feeding rate of Al powder (VAl) at 0.77 g/min, the resultant microstructure featured a γ-Ni matrix alongside a grid-like formation of γ′-Ni_3_Al/γ-Ni, leading to an increase in ultimate tensile strength by 8% and retaining an elongation of 90% compared to the deposited thin-wall lacking Al powder. As the VAl rose to 1.39 g/min, the deposited microstructure stayed the same; however, the distribution of precipitated γ′-Ni_3_Al/γ-Ni changed to a block-like shape because its average content increased from 18.22% to 50.48%. This subsequently led to fractures at the bead surface once the deposition height attained 11 mm. This research primarily examined how varying the quantity of Al powder impacts the microstructure changes in deposited Ni-Al IMCs, along with its mechanisms affecting mechanical properties and susceptibility to cracking.

Raising the VAl from 0.1 to 1.03 g/min enhanced the ultimate tensile strength (UTS) of the deposited thin-wall but reduced the elongation and heightened the anisotropy degree of UTS. Clear cracks showing typical intergranular fracture were visible on the end surface of deposited parts when the VAl was at least 1.39 g/min.

In comparison to the deposited thin-wall lacking Al powder, the horizontal and vertical ultimate tensile strength (495 MPa and 480.5 MPa) at the optimal VAl of 0.77 g/min improved by 12% and 8%, while the horizontal and vertical elongations (36.3% and 35%) decreased by 3% and 10%. Additionally, the anisotropy degree of UTS reduced from a peak of 5.18% at VAl of 1.03 g/min to 3.02%, showing a decline of 42%.

The changes in mechanical properties and the propensity for cracking of deposited Ni-Al IMCs were closely linked to the transformation of microstructures. The microstructure featuring a γ-Ni matrix and precipitated γ′-Ni_3_Al/γ-Ni arranged in a grid-like pattern was achieved at a VAl of 0.5–1.03 g/min, enhancing the UTS through a combination of precipitation strengthening and grain refinement. By raising the VAl to 1.39 g/min, the distribution of precipitated γ′-Ni_3_Al/γ-Ni shifted to a block-like morphology due to the higher average content of 50.48%, which directly caused cracks on the deposited surface because of the heightened brittleness.

Asirvatham et al. [77] examined how the Ni-plating technique and its thickness affect the laser weldability of Al busbars used in interconnects for lithium-ion battery cells. Three Ni plating techniques (electroless medium P, electrolytic bright, and sulfamate) were used at thicknesses of 3, 9, and 15 μm. It was discovered that Ni plating greatly affects weldability in laser-welded joints, as weld strength rises from 1 kN to 1.5 kN with greater plating thickness. Bright and electroless Ni plating showed better weld strength and hardness than sulfamate plating. The hardness of the weld varies from 60 to 75 HV with a plating thickness of 3 μm, goes up to 100–120 HV at 9 μm, and reaches 150–200 HV at 15 μm for both bright and electroless Ni plating. In comparison, sulfamate plating at 15 μm shows a marginally reduced hardness of 100–150 HV. The weld fracture surface showed a shift in failure mode from ductile to a combination of ductile and brittle, suggesting an increase in hard phases. Likewise, thicker plating typically raised porosity, while sulfamate plating showed low porosity (1.5%) even at greater thicknesses, unlike bright (10%) and electroless (3.1%). It was developed an Al–Ni eutectic structure featuring hard Al_3_Ni intermetallics distributed within a more ductile α-Al matrix, which enhances the noted mechanical properties.

Li et al. [78] researched a novel welding technique for Al/Ni dissimilar metals through molecular dynamics. The strong connection between Al and Ni was accomplished through Al-Ni reactive nano-multilayers (RNMLs). The tensile strength of the bonded element was 57% greater than that of diffusion-bonded Al/Ni nanowires. The Young’s modulus of the bonded material was 48.88 GPa greater than that of single-crystal Al and 68.09 GPa less than that of single-crystal Ni. An abundance of stair-rod dislocations and nano-coherent twins were produced during tensile loading, enhancing the mechanical properties of the bonded component. With the rising atomic ratio of Al-Ni RNMLs, the tensile strength initially dropped to 6.22 GPa before steadily rising to 7.48 GPa, while the Young’s modulus increased from 113.73 to 141.73 GPa and remained constant. When the atomic ratio was 1.98, Shockley dislocations and stair-rod dislocations were produced more readily. In the same way, an ignition temperature of 830 K produced the greatest tensile strength of the bonded components among all RNML ignition temperatures.

An alloy called IC-221M, composed of Ni_3_Al, integrates Ni with various other metals such as Cr, Mo, Zr, and B. Incorporating B enhances the alloy’s ductility by favorably modifying the grain boundary chemistry and encouraging grain refinement. The Hall-Petch parameters for this substance were σo = 163 MPa and ky = 8.2 MPa·cm1/2 [79]. B enhances the hardness of bulk Ni_3_Al through a comparable mechanism. This alloy boasts exceptional strength relative to its weight, being five times more robust than typical SAE 304 stainless steel. In contrast to the majority of alloys, IC-221M exhibits enhanced strength from room temperature to 800 °C.

The alloy demonstrates high resistance to heat and corrosion, making it suitable for heat-treating furnaces and various applications where its extended durability and lower corrosion provide an edge over stainless steel [80]. Research has shown that the microstructure of this alloy contains a Ni_5_Zr eutectic phase, making solution treatment effective for hot working without fracturing [81].

Walter and Cline [82,83,84] introduced a NiAl-Cr-Mo eutectic alloy with Mo replacing Cr. It led to a transformation in phase morphology from Cr rods within the NiAl matrix to varying lamellar plates when the Mo concentration surpassed 0.6 at.%. Additionally, the growth direction shifted from <100> to <111> following the addition of Mo. This phase system allows for an enhancement of both the fracture toughness and the creep strength at high temperatures. Johnson et al. [85] explored the mechanical properties of NiAl-Cr-Mo alloys. A containerless method of directional solidification was employed via the electromagnetically levitated zone process in an ultrapure He environment for the production of alloys. The fracture toughness measured for directionally solidified NiAl-28Cr-6Mo alloys was 22 MPa√*m*, in contrast to 6 MPa√*m* [86] found for polycrystalline NiAl. NiAl-Cr-Mo alloys are distinguished by a favorable blend of properties at both room and elevated temperatures. Nonetheless, the mechanical characteristics still require enhancement to rival the Ni-based superalloys.

The alternative eutectic composition of Ni-33Al-33Cr-1Mo was studied by Yang et al. [87] and subsequently by Whittenberger et al. [88]. In both studies, the fracture toughness of alloys produced through directional solidification was comparable (17 and 16 MPa√*m*, respectively). Whittenberger determined that at increased withdrawal rates (over 127 mm/h), the fracture toughness reduced notably (from 16 to 7 MPa√*m*), which was linked to the change in microstructure from lamellar forms to Cr(Mo) fibers/grains within the NiAl matrix. In the scenario of Mo-rich composite (Ni-33Al-31Cr-3Mo), the lamellar structure maintained the quicker growth rates. Additionally, it was noted that the levels of interstitial elements (like C, O, N) did not seem to be the primary reason for reducing the toughness of Mo-modified NiAl composites.

NiAl-Cr-Mo eutectic alloys continue to capture the interest of researchers because of the most favorable properties achieved for NiAl alloys to date. Cui et al. [89,90,91] and Guo et al. [92,93] suggested incorporating hafnium into NiAl-Cr-Mo alloys. Two alloys were chosen: 0.2 and 0.5 (at.%) of Hf (Ni-32Al-28Cr-(6−x)Mo-xHf, where x is 0.2 and 0.5 at.%, respectively) produced through directional solidification. In the initial composition (0.2Hf), a dendritic structure was noted, whereas in the 0.5Hf alloy, an intercellular structure developed. The inclusion of hafnium led to improved high-temperature strength and an increased brittle-to-ductile transition temperature (BDTT) because of a distinct strengthening mechanism. The ductility and creep resistance of NiAl-Cr-Mo alloys (Ni–33Al–28Cr–6Mo–0.2Hf (at.%) alloys subjected to various temperatures of strong magnetic field treatments: HT—no SMF treatment, MT1—1 h at 1346 °C, MT2—1 h at 1446 °C, and MT3—1 h at 1546 °C) have diminished due to the disruption of the cellular structure induced by hafnium. Following heat treatment in a strong magnetic field, a notable enhancement in creep properties at room temperature was recorded [94]. This enhancement was due to the conversion of hafnium from the Heusler phase (Ni2AlHf) into Hf solid solution within the NiAl-Cr(Mo)-0.2Hf alloy.

Guo et al. [95] suggested a more advanced composition of the NiAl alloy. Building on earlier research [96], the authors suggested incorporating a minor quantity of Ho into the NiAl-Cr-Mo-Hf alloy. The alloys were generated through arc-melting in an Ar environment utilizing a non-consumable W electrode. Alloys were melted again over five times to achieve a uniform chemical composition. The alloy buttons were subjected to homogenization at 1250 °C for 24 h in evacuated silica capsules, then cooled to room temperature in a furnace. The greatest enhancement of compressive properties was noted when the Ho concentration reached 0.1%.

The addition of Ho led to a lamellar enhancement in eutectic structures within the NiAl-28Cr-6Mo-0.15Hf alloy. As the Ho content increased, the eutectic cells were refined, but the intercellular areas became coarser. Liang et al. [97] suggested the production of a comparable NiAl alloy [NiAl–28Cr–5.94Mo–0.05Hf–0.01Ho (at.%)] utilizing the liquid metal cooling method employed by Johnson [85], testing two growth rates (3 and 10 mm/min). The aligned microstructure exhibited a finer and more disrupted state at a growth rate of 10 mm/min compared to 3 mm/min. The yield strength at 1100 °C was comparable for both alloys produced at growth rates of 10 mm/min and 3 mm/min, and it was greater than that of an alloy made using the Bridgman method. This enhancement effect resulted from a more organized microstructure, improved solid solution strengthening, and the beneficial impact of Ho. The fracture toughness of the alloy produced at 3 mm/min was slightly greater than that at 10 mm/min due to the improved alignment of the microstructure.

A hypereutectic alloy Ni-31Al-32Cr-6Mo was recently introduced in [98,99]. Based on the foundational research concept, an increase in the volume fraction of the toughening phase in a hypereutectic alloy may lead to a higher melting point for the alloy, along with improved mechanical properties. Furthermore, to enhance fracture toughness, the authors utilized findings from prior studies, where a fully eutectic microstructure was achieved in an off-eutectic alloy under conditions of increased temperature gradient [100,101]. These two characteristics, namely the hypereutectic composition and elevated temperature gradient, might greatly improve the fracture toughness at room temperature. The fracture toughness of the Ni-31Al-32Cr-6Mo alloy attained 23.74 MPa√*m* when solidified at a rate of 10 μms^−1^ and exhibited a planar eutectic microstructure. In this alloy, a quasi-cleavage fracture pattern was seen on the surfaces of the fracture.

The greatest fracture toughness recorded to date (26.15 MPa√*m*) was achieved in a NiAl-36Cr-6Mo hypereutectic alloy that was directionally solidified using the zone melted liquid metal cooling method (ZMLMC) at a withdrawal speed of 10 μm/s and a temperature gradient of 600 K/cm [102]. The improvement in fracture toughness was due to the increased volume of the strengthening phase and the achievement of a well-oriented microstructure featuring a planar eutectic microstructure in the hypereutectic alloy.

Azarmi et al. [103] produced NiAl composites strengthened with SiC particles using a hot-pressing method. It was discovered that the greater stiffness of SiC particles in contrast to the NiAl matrix, along with robust matrix/reinforcement interface characteristics, might enhance the creep properties of the reinforced composites. Nevertheless, the composite manufacturing method requires enhancement because of the low relative density and inadequate strengthening effect of the produced composites.

It is widely recognized that the combination of a nanocrystalline intermetallic matrix with a dispersed strengthening phase can enhance strength at elevated temperatures and improve material ductility [104,105]. For instance, Zhou et al. [106] synthesized nanocrystalline NiAl-TiC powders, during which two distinct reactions for compound formation happened concurrently. An extensive investigation into the solid-state reaction that takes place during this mechanical alloying process was conducted by Krivorutcho et al. [107]. Whittenberger et al. [108] also made attempts to enhance the mechanical characteristics of NiAl, exploring two distinct strengthening mechanisms at elevated temperatures. They determined that ODS (oxide dispersion strengthened) NiAl produced through cryomilling or roasting in nitrogen was not as robust as cryomilled NiAl with similar levels of AlN.

Mechanical alloying (MA) was employed to produce NiAl composites with the inclusion of refractory metals Fe, Ga, and Mo [109]. The powders were ultimately consolidated using the hot pressing method. The maximum mechanical properties were achieved with a mix of transition elements (NiAl + 6%Fe + 2%Mo), reaching a strain of 19.2%. Samples were sintered at 1200 °C for 30 min, achieving a relative density of 96.3% for the sintered material.

Sheng et al. [110] created NiAl alloys incorporating Au in amounts up to 1 atomic %. The findings from their research indicated that (i) incorporating Au into the NiAl alloy refines the NiAl grains, (ii) an increased amount of Au leads to the development of the α-AlAu2 phase, and (iii) the addition of Au causes moderate solid solution strengthening at low temperatures and significant solid solution strengthening at high temperatures.

In a recent study, Liu et al. [111] suggested enhancing the high-temperature tribological and mechanical characteristics of NiAl through the incorporation of Mo2C. The hot-pressing method was chosen for consolidation. The hardness and density rose as the Mo2C content increased, and the mechanical properties were superior to those of the NiAl alloy. The friction coefficient at 700 °C attained 0.35 with 8% vol. content of Mo2C, while it was 0.48 for NiAl. A common fracture morphology of NiAl-Mo2C composite (with 8 wt.% Mo addition) features a transgranular cleavage facet crack fracture mode combined with a dimple fracture.

There was considerable attention given to doping the NiAl structure with Hf alongside other elements. In 1994, Darolia [112] secured a patent for a NiAl single-crystal intermetallic alloy that demonstrated enhanced high-temperature strength, achieving optimal results with a chemical composition of 0.05Ga, 0.5Hf, and 5.0Ti (at.%).

Within the ductile phase toughening mechanisms of Ni aluminides, two additional mechanisms deserve mention: (a) toughening through martensitic transformation, and (b) heat treatment, which can also be utilized after the ductile phase toughening.

**(a) Transformation of martensite**

The impact of the martensitic transformation of beta-NiAl was examined in [113,114,115]. Martensitic transformation can take place in Ni-rich NiAl through swift cooling (B2 to L10).

The findings in [115] indicate that the martensite phase undergoes deformation at lower stresses compared to B2-NiAl. In [103], the authors examined the effect of martensitic transformation (both thermal and stress-induced) on the fracture toughness of NiAl. The martensitic transformation affected the fracture toughness solely in the scenario of an as-cast inhomogeneous polycrystalline NiAl, where the martensitic phase irreversibly develops at crack tips without reverting. Kainuma et al. [116] and Thompson et al. [117] examined the effects of ternary elements on the martensitic transformation of β-NiAl. The impact of ternary elements on the martensitic transformation in relation to the fracture toughness of NiAl has not been documented so far. Numerous studies detailed the transformation in NiAl through molecular dynamics simulations [118,119,120]. In terms of high-temperature use of NiAl, the deviation from the stoichiometric ratio, essential for martensitic transformation in NiAl, will lead to a decline in physical and mechanical properties at elevated temperatures (like thermal stability). Nonetheless, this kind of toughening might be appealing in various areas of NiAl usage.

**(b) heat treatment**

More than a dozen papers discuss the impact of heat treatment on NiAl alloys, but only a handful address its effect on fracture toughness. Significant efforts were made to enhance the NiAl-Cr-Mo alloys infused with Hf. As noted in the previous section, the formation of the Heusler (Ni2AlHf) phase in the NiAl-Cr-Mo-Hf alloy decreases the mechanical properties. As the proportion of the Heusler phase rises in the Hf-doped alloy NiAl/Cr(Mo), the interface weakens compared to the constituent phase. Cracks can readily form at interfaces where the Ni2AlHf phase is present [90]. An initial investigation by Cui et al. [121] indicated that following HIPing and aging treatment (HIP at 1300 °C/100 MPa for 2 h, followed by aging at 1050 °C for 40 h), the density of the Heusler phase at the NiAl/Cr(Mo) phase boundary is considerably reduced, whereas the mechanical properties improved. Recently, Wang and colleagues [122] showcased the progression of fracture toughness throughout the heat treatment of NiAl-Cr(Mo)-(Hf, Dy)-Fe alloy. Following the heat treatment at 1250 °C for 48 h and aging at 1050 °C for 24 h, the fracture toughness of the NiAl alloy increased to 18.4 MPa√*m*, compared to the as-cast material, which had 13.7 MPa√*m*. Conversely, the alteration in microstructure due to high temperature exposure, grain growth, and variations in mechanical properties is undesirable from an application perspective.

Summarizing, the enhancement of ductility in NiAl intermetallic compounds arises from the industry’s increasing demand for innovative materials that can boost engine performance while lowering fuel consumption and maintenance needs. Given that NiAl-based materials could serve as a potential alternative to Ni-based superalloys, primarily as structural components in aero engines, several criteria need to be met. Bewlay et al. [123,124] indicated that to substitute the Ni-based superalloys, (i) the creep strength must exceed 170 MPa at a creep rate of 2 × 10^−8^ s^−1^ at 1200 °C (assuming a material density of 7 g/cm^3^), and (ii) the minimum fracture toughness should surpass 20 MPa√*m*.

NiAl alloys and composites can be used as structural materials for high temperatures. Following over 40 years of investigation into NiAl, a rise in fracture toughness greater than 400% has been attained (from 6 MPa√*m*, [86] to 26.15 MPa√*m*, [102]). These research findings indicate that the chemical makeup of high-performance NiAl-based materials should, in addition to Ni aluminide, include Cr and Mo in appropriate ratios, and possibly one or more additions of rare earth metals or other compounds to compete with Ni-based superalloys utilized in aircraft engines.

An evaluation of the processing methods for NiAl-base materials indicates that directional solidification offers the most favorable combination of mechanical properties, particularly when paired with the zone melted liquid metal cooling technique (ZMLMC) [100,101,102]. Moreover, the arc-melting methods appear to be promising [95,96].

Powder metallurgy methods could also be intriguing [109,110,111], but based on the results shown, this approach appears to be less efficient compared to directional solidification. The NiAl alloys produced through micro-alloying exhibit highly encouraging mechanical characteristics; however, the grain coarsening that occurs during high-temperature cycles needs to be considered since it could lead to material degradation.

The brittle nature of polycrystalline NiAl arises from a limited number of independent slip systems [125,126]. The primary reason for enabling extra slip systems in the B2-ordered structure of NiAl to enhance its ductility is the incorporation of an additional element into the intermetallic framework. The research findings presented support this assertion and demonstrate that this method can produce beneficial outcomes for the NiAl application in aircraft engines.

Even if NiAl alloys or NiAl-based composites can be utilized in turbine blades, they cannot compete with the next generation of Ni-based superalloys. At present, the 6th generation TMS-238 Ni-superalloy demonstrates usability up to 1120 °C for 1000 h of creep life at 137 MPa [127,128] and displays a favorable combination of mechanical and environmental characteristics. Conversely, NiAl turbine blades, although they have poorer mechanical properties compared to Ni-superalloys, could be more appealing if they demonstrate enhanced temperature resistance, corrosion resistance, or an extended creep lifespan. [129].

**Oxygen dispersion strengthened Ni alloys**

Ni-based Oxide Dispersion Strengthened (ODS) alloys are a category of high-temperature alloys characterized by the distribution of small oxide particles within the Ni matrix, which improves their strength, creep resistance, and various mechanical properties. These particles, typically alumina (Al_2_O_3_) or yttria (Y_2_O_3_), serve as barriers to dislocation movement, hindering material deformation and enhancing resistance to deformation at elevated temperatures [130,131,132]. The tiny oxide particles, usually sized between 5 and 50 nanometers, are evenly distributed within the Ni matrix. This dispersion generates a pinning effect on dislocations, which are flaws in the crystal structure that permit the material to deform when under stress. By impeding the movement of these dislocations, the oxide particles substantially enhance the material’s strength and resistance to creep at elevated temperatures [130,131]. ODS Ni alloys are especially useful in situations that demand high-temperature resistance and longevity, including aerospace parts and nuclear reactors. Their capacity to uphold mechanical stability at high temperatures renders them an appropriate selection for challenging conditions applications [130,132].

ODS Ni alloys are frequently manufactured through powder metallurgy methods, including spark plasma sintering (SPS) or hot isostatic pressing (HIP). These techniques enable accurate regulation of the oxide particle dimensions and dispersion in the Ni matrix [130]. Besides their strength at elevated temperatures and resistance to creep, ODS Ni alloys also demonstrate excellent corrosion resistance and ability to withstand radiation, which makes them appropriate for various [130,131,132]. ODS Ni alloys are commonly employed in manufacturing gas turbine engines, nuclear reactors, and various high-performance applications where durability and resistance to harsh conditions are essential [130,132].

ODS Ni alloys were fabricated through mechanical milling and spark plasma sintering (SPS) of Ni–20Cr powder, with an added dispersion of 1.2 wt.% Y_2_O_3_ powder. Additionally, 5 wt.% Al_2_O_3_ was incorporated into Ni–20Cr–1.2Y_2_O_3_ to enhance composite strengthening in the ODS alloy. The influence of milling duration, sintering temperature, and sintering hold time was examined concerning both mechanical characteristics and microstructural development. A substantial quantity of annealing twins was noted in the sintered microstructure for every milling duration. Nonetheless, extended milling duration led to enhanced hardness and reduced twin width in the consolidated alloys. An increased sintering temperature resulted in a greater proportion of recrystallized grains, along with enhanced density and hardness. Incorporating 1.2 wt.% Y_2_O_3_ into the Ni–20Cr matrix notably diminished the grain size due to the dispersion strengthening effect of Y_2_O_3_ particles in regulating grain boundary mobility and recrystallization processes. The mechanisms of strengthening at room temperature were measured using both experimental and analytical methods, showing good consistency. A significant yield stress from high compression achieved at 800 °C for the Ni–20Cr–1.2Y_2_O_3_–5Al_2_O_3_ alloy was ascribed to the synergistic influence of dispersion and composite reinforcement [133].

ODS superalloys find extensive application in aerospace and nuclear reactors because of their remarkable creep strength and resistance to fatigue cracking at high temperatures. In this study, a laser powder bed fusion (L-PBF) technique was used to create an ODS NiCrFeY alloy suitable for high-temperature applications. The microstructure and mechanical characteristics at ambient and high temperatures of the L-PBF processed ODS NiCrFeY alloy were examined. It was shown that equiaxed grains featuring a significant proportion of low-angle grain boundaries and cellular structures with a high density of dislocations were produced in the as-built alloy. Y_2_O_3_ nanoparticles were generated in situ within the ODS NiCrFeY alloy. The majority of the Y_2_O_3_ nanoparticles were situated at the cell membranes and were trapped with numerous dislocations. The ODS NiCrFeY alloy demonstrated elevated ultimate tensile strengths of 810, 560, and 250 MPa at room temperature, 600 °C, and 800 °C, respectively, because of the enhancing effects of dispersed Y_2_O_3_ nanoparticles and fine cellular structures [130].

Yalcin et al. [134] developed an Inconel 718 alloy (IN718) enhanced with nano-oxides. The composition of the alloy is 0.3 wt.% Y_2_O_3_—IN718 has been established using a thermochemical modeling approach based on CALPHAD. The ODS-IN718 alloy, engineered for this purpose, is manufactured using the Selective Laser Melting (SLM) technique with different power and speed settings to identify the optimal processing conditions for this system. The SLM parameters are refined for the manufacturing of ODS-IN718 alloy. To eliminate the non-equilibrium phases that negatively affect the mechanical properties and enhance the number densities of nano-oxide and other strengthening phases of γ′/γ″, several heat treatment processes are utilized according to thermochemical calculations. The samples that are treated at 1050 °C for 1 h and then aged at 650 °C for 5 h show the most favorable microstructural and mechanical characteristics. Tensile tests indicate that the strength and ductility of the solutionized and aged samples show significant enhancement over the standard solutionized and double-aged samples, owing to the fine and uniform microstructure, particularly at high temperatures.

## 3. Welding Characteristics of Ni and Ni-Based Alloys

**Weldability**

Weldability is a technological characteristic used to evaluate the ability and potential of different metals to be effectively joined during a specific welding procedure. Weldability has been described in various ways, but the most commonly recognized definition is: “Weldability is the capacity of a material to be welded under the imposed fabrication conditions into a specific, suitably designed structure performing satisfactorily in the intended service” [9]. The welded joint must closely resemble the base metal characteristics to ensure safe and dependable component performance in its operational setting. Despite having some weldability challenges, Ni and its alloys can be effectively welded when proper welding and fabrication methods are employed.

Welding methods employed for Ni and its alloys share numerous similarities with those utilized for stainless steel. Due to the relatively high viscosity of molten Ni and its alloys, they necessitate heat source oscillations (weaving) and greater precision when managing the weld pool and introducing filler metal. Caution must be exercised when selecting base metal alloy, appropriate filler metal, weld joint design, welding technique, and related welding parameters. Post-weld heat treatment may be needed to alleviate residual stresses from welding and attain the desired properties of the joint. [4,135].

**Weldability tests**

Numerous tests have been created to evaluate the weldability of materials, yet only a few have been standardized. Weldability assessments can be classified into four main types: mechanical, non-destructive, service performance, and specialty evaluations. All tests, except nondestructive ones, necessitate weld sectioning for metallographic assessment. A primary aim of the weldability test is to assess the alloy’s vulnerability to different kinds of cracks that may arise during the welding process. Thus, weldability assessments remain a crucial method for assessing the cracking susceptibility of Ni-based alloys [5].

The most prevalent hot cracking weldability assessment is the Varestraint test, created in the 1960s. Today, numerous Varestraint tests have been created based on the original. The main concept of this test is to impose controlled strain on the specimen (greater than 3 mm) while welding to encourage crack formation. Consequently, several distinct metrics can be utilized to measure an alloy’s susceptibility to cracking, including total crack length, maximum crack length, or the threshold strain required to trigger cracking. The Varestraint test has been utilized to evaluate the susceptibility of various Ni-based alloys to liquation and solidification cracking [3,136].

The Sigmajig Test is utilized to assess the susceptibility to solidification cracking in thinner sheets (<3 mm) where the Varestraint test is less suitable. The testing apparatus is intended to regulate the specimen’s level of restraint, allowing for the assessment of the correlation between applied stress and the emergence of cracks. The stress range at which solidification cracking takes place serves as a valuable indicator of an alloy’s resistance to cracking—a more limited range signifies greater resistance to solidification cracking. [4].

The Hot-Ductility test is intended to assess the ductility loss of an alloy that occurs during the thermal cycle of welding. This is a crucial step since weld cracking is often a result of reduced ductility. This test has been employed to assess liquation cracking and ductility dip cracking in different Ni-based alloys. The metric known as liquation cracking temperature range (LCTR) is frequently utilized to measure an alloy’s susceptibility to liquation cracking. It is described as the temperature variation between the point at which the alloy’s strength falls to zero and the point where ductility is regained to an observable degree (a narrower LCTR signifies improved cracking resistance) [4].

The Strain-to-Fracture-Test was created to assess ductility-dip cracking, a phenomenon observed in Ni-based and various austenitic alloys. The test sample is cut from base metal and reduced (narrowed) in the central area where autogenous spot GTAW is performed. It is subsequently placed in the Gleeble thermo-mechanical simulator, heated to a critical temperature (in vacuum or inert gas) where the ductility dip occurs, and ultimately subjected to the specified strain level during welding. Conducting various tests at different temperatures allows for the identification of the alloy’s ductility-dip temperature range—an alloy with a broader ductility-dip temperature range and a lower temperature threshold for crack formation is deemed more prone to crack development [3].

According to [137], welding Ni-based superalloys has consistently posed a significant challenge for researchers. The elevated dynamic shear strength and increased strain hardening tendency complicate the welding process. Inadequate penetration, microfissures in the heat-affected zone (HAZ), weakened strength in HAZ, and compromised mechanical properties in the weld fusion zone (FZ) are several challenges faced in various welding methods when welding Ni-based super alloys. The authors reviewed the welding studies of Ni-based superalloys using various welding techniques such as gas tungsten arc welding (GTAW), gas metal arc welding (GMAW), electron beam welding (EBW), laser beam welding (LBW), and friction stir welding (FSW). The majority of welding methods are deemed appropriate for welding Ni-based alloys, whereas EBW and LBW methods provide benefits such as low heat input, a high weld depth-to-width ratio, a narrow HAZ, minimized distortion, and superior mechanical properties in comparison to arc welding of Ni-based alloys.

Organizations like the American Society for Testing and Materials (ASTM) and the International Organization for Standardization (ISO) developed several standards and tests for welds, which can be of a destructive or non-destructive nature.

Destructive ASTM standards for welding joint evaluations mainly aim to measure the strength and functionality of welds through physical failure testing. Typical examples consist of tensile tests, bending tests, impact assessments, and macro etching evaluations, which assist in assessing attributes such as strength, ductility, toughness, and weld integrity. Relevant ASTM standards pertinent to these evaluations comprise ASTM E8 (Standard Test Methods for Tension Testing of Metallic Materials), ASTM E290 (Standard Test Methods for Bend Testing of Metallic Materials), and ASTM E208 (Standard Test Method for Charpy Impact Testing of Metallic Materials) [138,139,140,141,142,143,144,145,146,147,148].

Destructive testing of welded joints is commonly utilized for welding procedure specification approval according to standards as EN ISO 15614-1 for steels or EN ISO 15614-2 for Al and its alloys. Hardness tests for the specification and qualification of welding procedure, by EN ISO 15614-1, are realized for:

- Butt joint with full penetration,
- T-joint with full penetration,
- Branch connection with full penetration,
- Fillet welds [149].

Example set of destructive tests for specification and qualification of welding procedure for butt weld with full penetration for level 2, in accordance with ISO 15614-1, comprises:

- transverse tensile test (2 specimens),
- transverse bend test (4 specimens),
- impact test (2 set of 3 specimens),
- hardness test (1 specimen),
- macroscopic examination (2 specimens).

Tensile tests for metallic materials are commonly realized according to standards ASTM E8/E8M and ISO 6892-1. It is also possible to carry out tensile tests at elevated (ISO 6892-2) or low temperature (ISO 6892-3).

Usually (when qualifying welding procedure in accordance with ISO 15614-1) specimens are made to meet requirements of ISO 4136.

For welded joints most common bending tests are realized according to standard ISO 5817, which is required by ISO 15614-1.

In welding, as per ISO 15614-1 requirements, for Charpy Impact Tests V V-notch is used. The test should be conducted according to the ISO 9016 standard [150].

It should be emphasized, however, that the quality of the results of individual tests is strongly dependent on the type of welding, joined and connecting materials. The use of these tests, and especially the recognition of factors influencing their course, is most advanced in relation to steel and Al and its alloys.

ASTM standards provide numerous non-destructive testing (NDT) techniques for evaluating weld joints, confirming their integrity without harming the material. Several important ASTM standards comprise:

- Radiographic Testing:
  1. ASTM E165: Standard Practice for Radiographic Examination of Welds.
  2. ASTM E1032: Standard Practice for Radiographic Examination of Weldments Using Industrial X-ray Film.
- Ultrasonic Testing:
  1. ASTM E709: Standard Practice for Ultrasonic Inspection of Weldments.
- Other NDT Methods:
  1. ASTM E1312: Standard Practice for Electromagnetic (Eddy-Current) Examination of Ferromagnetic.
  2. ASTM E2261: Standard Practice for Examination of Welds Using the Alternating Current Field.
  3. ASTM E2984/E2984M: Standard Practice for Acoustic Emission Examination of High Pressure, Low Carbon, Forged Piping [151,152,153,154,155,156].

**Welding techniques employed for connecting Ni and Ni-based alloys**

Hinshaw [157] reported that Ni alloys can be effectively joined using all welding techniques or methods, except for forge welding and oxyacetylene welding. The author examined the heat treatment of Ni alloys and gave nominal compositions for specific weldable wrought Ni and Ni alloys. It offered details about gas-tungsten arc welding, gas-metal arc welding, plasma arc welding, shielded metal arc welding, and submerged arc welding for welding Ni alloys. It was also examined the issues faced in the arc welding of Ni alloys, such as porosity, cracking, and stress-corrosion cracking. The author additionally assessed the elements that affect the selection of filler metal and welding technique for Ni alloys.

**Figure 1:**
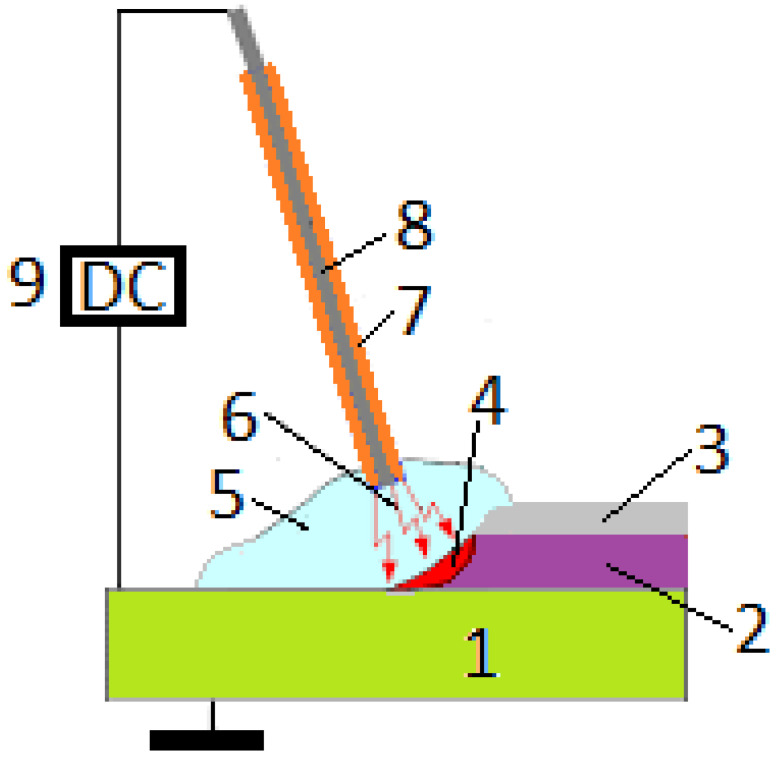
Scheme of SMAW process. 1—workspiece, 2—shielded weld, 3—slag layer, 4—weld pool, 5—processing gas, 6—arc, 7—coating, 8—electrode, 9—DC power supply.

**Figure 2:**
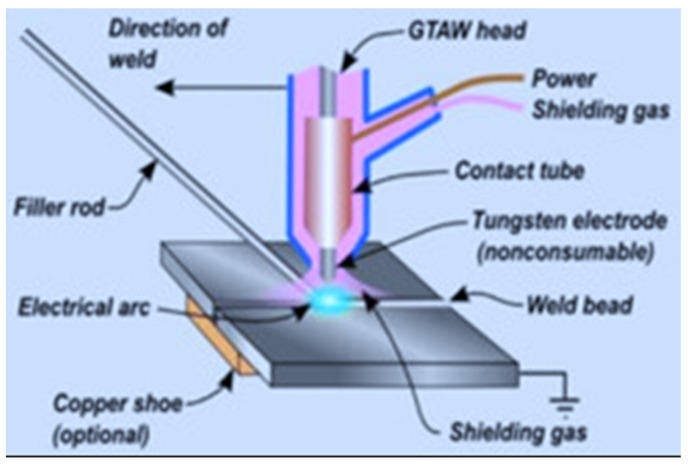
Scheme of GTAW process [164].

**Figure 3:**
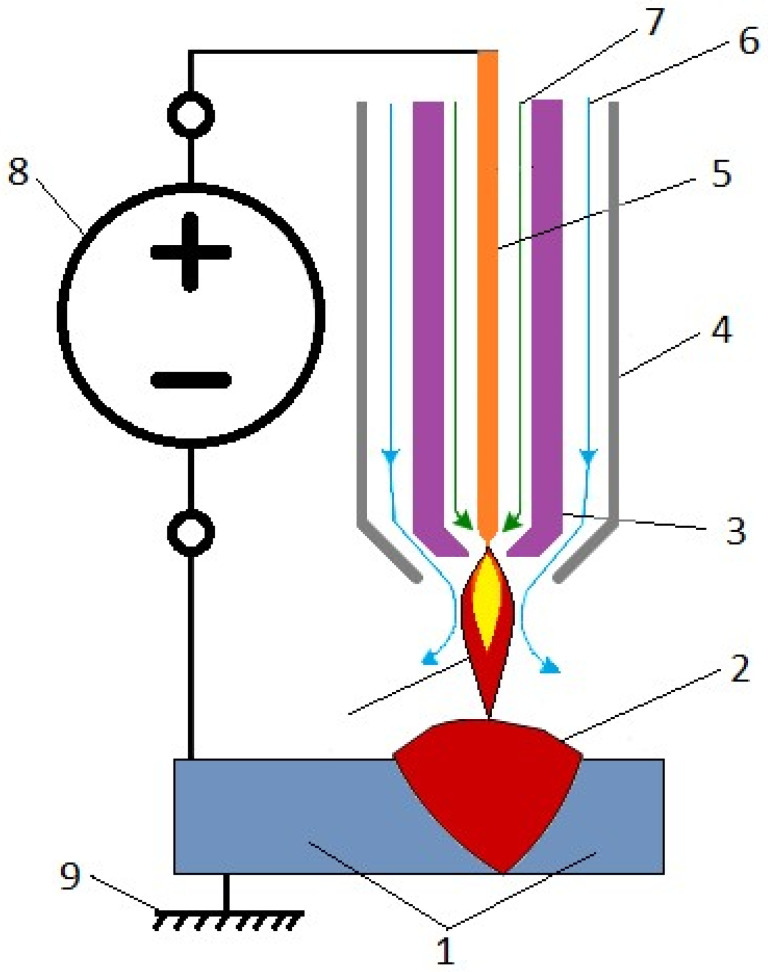
Scheme of PAW process. 1—workpieces, 2—weld pool, 3—constricting cooled nozzle, 4—shielding gas nozzle, 5—non-consumable W electrode, 6—shielding gas, 7—plasma gas, 8—DC power supply, 9—ground.

**Figure 4:**
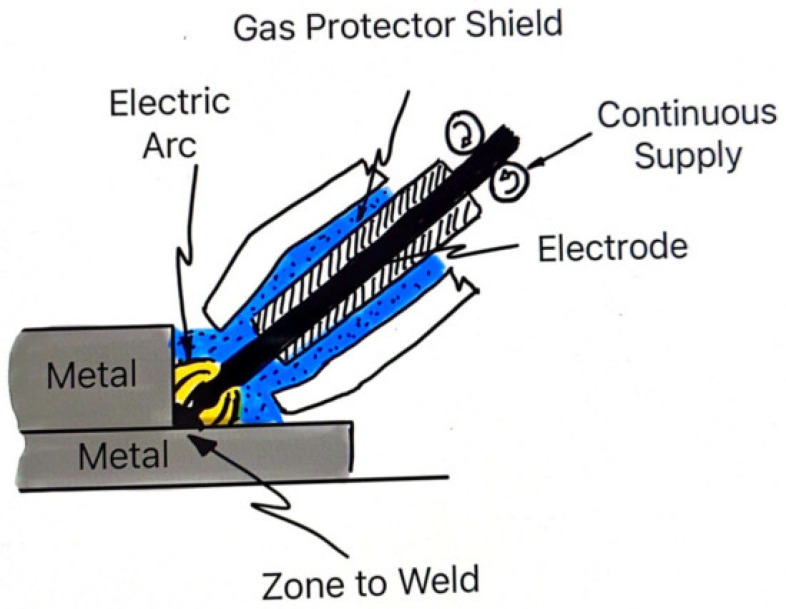
Scheme of GMAW process [165].

**Figure 5:**
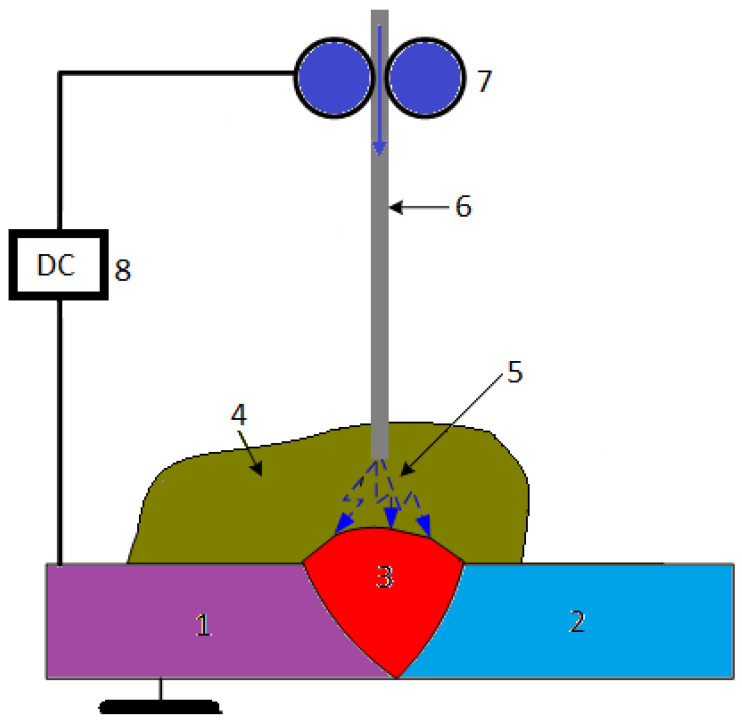
Scheme of SAW process. 1, 2—joined elements, 3—weld pool, 4—fused covering flux, 5—submerged arc, 6—electrode wire, 7—drive rolls, 8—DC power supply.

**Figure 6:**
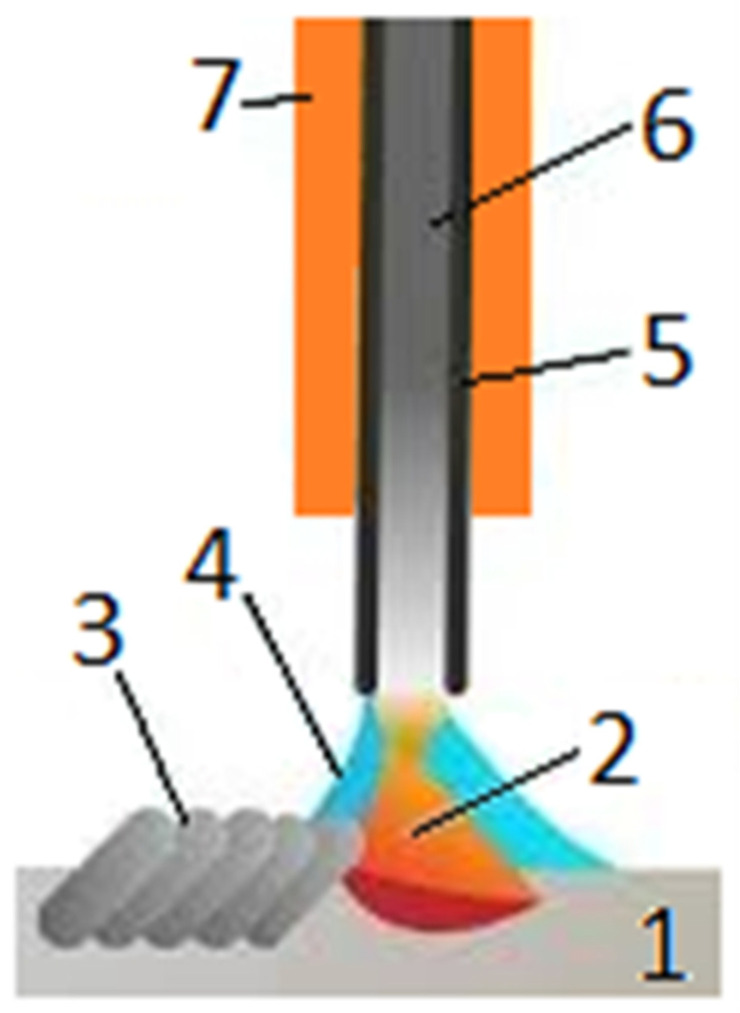
Scheme of FCAW process. 1—base material, 2—arc, 3—slag, 4—shielding gas, 5—tubular electrode, 6—flux core, 7—gas nozzle.

Commonly used arc welding techniques such as shielded metal arc welding (SMAW) shown in Figure 1, gas tungsten arc welding (GTAW) presented in Figure 2, plasma arc welding (PAW) shown in Figure 3, gas metal arc welding (GMAW) presented in Figure 4, and submerged arc welding (SAW) shown in Figure 5 can effectively join CP Ni and SSS Ni alloys. Flux-cored arc welding (FCAW), presented in Figure 6, is utilized less frequently. Even though oxyacetylene welding (OAW) can be performed, arc welding processes should always be favored. This is particularly crucial when welding the low-carbon Ni alloy, as it may take in unwanted carbon from the oxyacetylene flame. PS alloys are typically welded through the GTAW process due to their ability to offer outstanding protection from oxidation and minimize the loss of hardening elements, as well as decreasing the likelihood of hot cracking, [4,158,159,160]. The traditional GTAW method exhibits relatively low productivity; however, various process variants have been created and implemented to enhance welding productivity and penetration while minimizing heat input. Among them are pulsed GTAW (P-GTAW), activated GTAW (A-GTAW), and keyhole GTAW (K-GTAW), [161]. The direct current electrode negative (DCEN-GTAW) setup is typically utilized for welding Ni and its alloys. To ensure proper weld joint protection and achieve a high-quality weld, it is advisable to utilize a broad gas cup fitted with a gas diffuser (gas lens) and perform back-purging at the root side of the joint. The GMAW process offers greater productivity, although the control of the weld pool is not as good as in GTAW. An affordable and straightforward SMAW method can also be employed to bond Ni and Ni-based alloys. The mechanical characteristics of weld joints created through arc welding of Ni and Ni-based alloys are greatly influenced by the type of process used and the associated welding parameters. Noticeable variations in the mechanical properties of weld joints can be seen even among various types of the same process, including conventional GTAW and P-GTAW, and A-GTAW. In general, both SSS and PS alloys can achieve high weld joint efficiency; however, it is anticipated that joint ductility and toughness will be reduced compared to the base metal, particularly when welding PS alloys [159,161,162,163].

**Figure 7:**
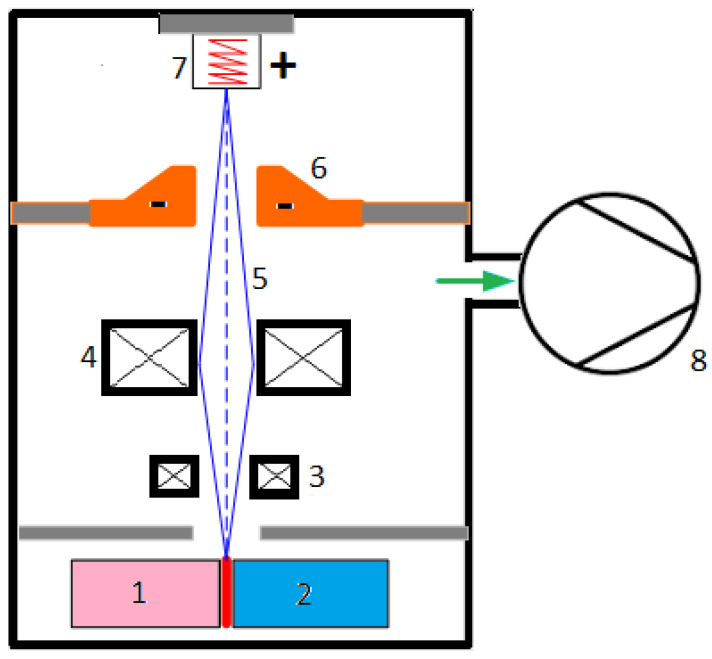
Scheme of EBW process. 1, 2—joined elements. 3—deflection coil, 4—focusing coil, 5—electron beam, 6—anode, 7—cathode, 8—vacuum pump.

**Figure 8:**
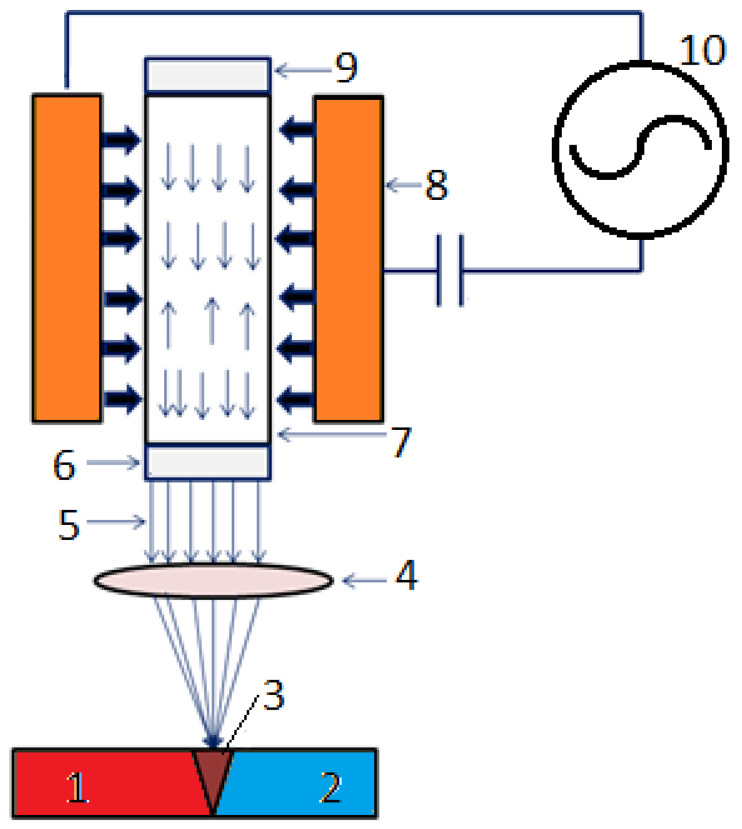
Scheme of LBW process. 1, 2—joined elements, 3—weld, 4—lens, 5—laser beam, 6—partially reflecting mirror, 7—Rb crystal, 8—flash lamp, 9—fully reflecting mirror, 10—power supply.

In addition to electric arc welding methods, Ni and its alloys are also joined using electron beam welding (EBW), as shown in Figure 7, and laser beam welding (LBW), as presented in Figure 8. These methods offer equivalent or even superior joint characteristics in comparison to GTAW. Their primary benefits include outstanding defense against atmospheric contamination, a high ratio of weld depth to width, minimal heat input, a narrow heat-affected zone (HAZ), and reduced distortion. For these reasons, the EBW and LBW methods can facilitate the joining of certain Ni-based alloys that are deemed challenging to weld with arc welding. Nonetheless, hot-cracking and porosity may frequently occur in instances of deep penetration welds, [4,158,166,167,168,169]. The mechanical characteristics of weld joints created through laser or electron beam welding of Ni and its alloys resemble the base metal in alloys that exhibit strong weldability. Nevertheless, for PS alloys, the properties of weld joints are frequently less than those of the base metal, although they can be greatly enhanced by suitable heat treatment, which is often impractical in numerous situations [166,167,168].

While most weldability problems with Ni and Ni-based alloys are linked to metallurgy, process-related issues can also lead to significant defects in weld joints like incomplete fusion or penetration, undercut, and overlap. Most of them can be avoided if the correct fusion zone geometry is obtained. Moreover, correct fusion zone shape can help diminish welding residual stresses and the likelihood of cracking. The geometry of the fusion zone is mainly influenced by the parameters of the welding process, which is why their frequently intricate relationship is of major concern.

Friction welding (FRW) and friction stir welding (FSW) are solid-state welding techniques that have been effectively employed to join Ni-based alloys. Their benefits consist of minor welding distortion, weld joint characteristics that surpass those of fusion welds, comparatively high welding speed, and the removal of solidification-related flaws like cracks and porosity. However, these methods necessitate robust clamping tools and are more rigid than arc welding [170,171,172,173].

**Figure 9:**
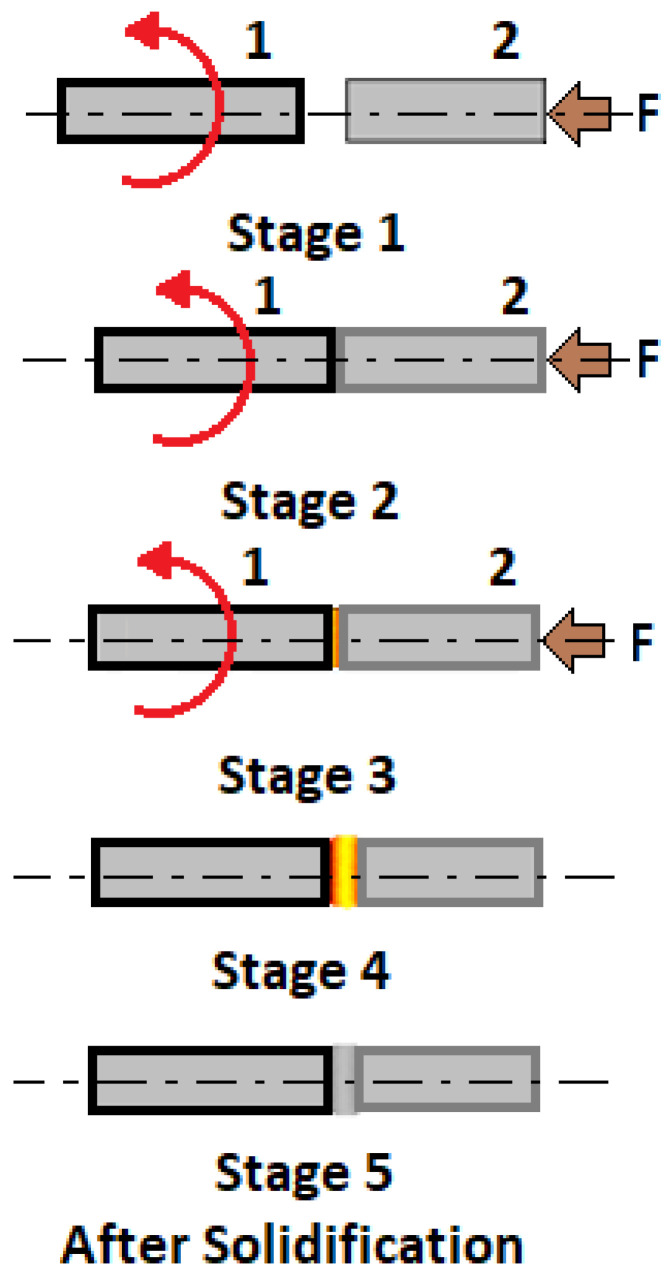
Scheme of FRW process. 1—rotating element, 2—non-rotating element, F—force.

**Figure 10:**
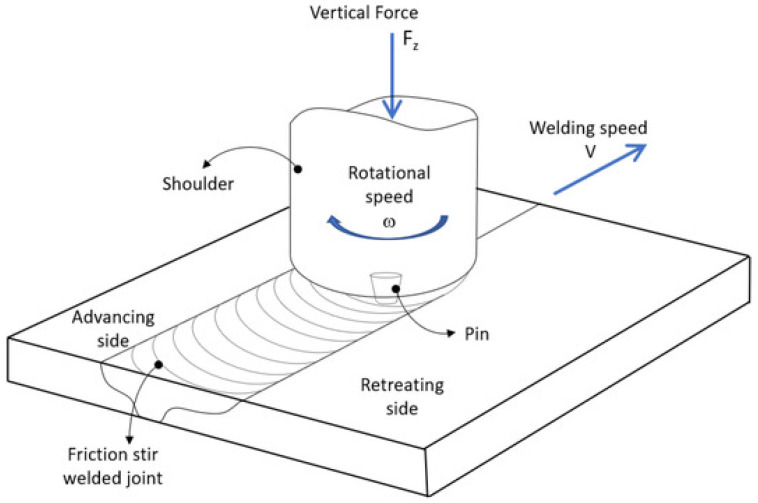
Scheme of FSW process [174].

Processes like friction welding (Figure 9) or friction stir welding (Figure 10) in solid-state welding produce weld joints with superior mechanical properties than those created by fusion welding. The strength of these welded joints frequently surpasses that of the base metal at the point of failure. This enhancement in weld joint strength can be linked to the grain refinement that occurs in the weld joint area. Nevertheless, mechanical characteristics like fatigue and fracture toughness still require comprehensive assessment to completely grasp the effects of solid-state welding methods on Ni and Ni-based alloys [171,172].

According to [172], recently, there has been a growing interest in the use of Ni-based alloys within the oil industry. In subsea engineering, the ability to weld high-strength materials efficiently is crucial. FSW is a method to join various materials while maintaining or even enhancing their characteristics. This information is important for Corrosion-Resistant Alloys (CRA) utilized in the deep-sea extraction of hydrocarbons. Until now, studies have concentrated on FSW of Inconel alloys 600, 625, and 718. FSW shows promise for Ni alloys; however, it relies on future research concerning tool technology and corrosion studies.

Smith and Grant [175] reported that Ni-based superalloys are difficult to join with other processes. Fusion-based processes often lead to liquation cracking of such alloys. Fusion processes require a high level of skill and/or a high level of process control.

Limited joining processes are used for Ni-nased alloys:

- Diffusion bonding
- Plasma arc welding
- GTAW

FSW/P of high melting point alloys is costly; however nickel-based superalloys can be joined using FSW/P due to:

- Existing processes are costly and time-consuming.
- Nickel-based super alloy products are high value.

There are some limitations in FSW/P of Ni-based super alloys:

- Tool wear and failure are noted as a common issue.
- During tensile tests, achieving failure outside FSW/P stir zone is possible.
- Lack of commonality in FSW/P efforts.

Using FSW/P, the joint of wrought Haynes 282 was obtained for sheets of 5 mm and 9.5 mm, being in a solution annealed condition. Weld Parameters with w/argon shielding comprised:

▪ Travel Speed: 0.42 mm/s.
▪ Temperature Control: 800 to 850 °C.
▪ Axial Force: 62 kN (based on visual quality).
▪ Rotational speed: 50–100 RPM (output variable from temperature control).
▪ Tool: MegaStir PCBN Q60 FSW Tools w/4 and 6 mm pin length.

Characterization before and after the standard 2-step heat treatment includes:

▪ Full solution anneal.
▪ Step 1: 1010 °C for 2 h then air cooled.
▪ Step 2: 788 °C for 8 h then air cooled.

The temperature control algorithm for successful FSW/P of steel alloys resulted in oscillatory behavior. It was found that controller tuning is required, although it defaulted to manual temperature control for initial efforts. Oscillation led to the premature failure of FS tools in the form of pin failure and shoulder cracking.

Acceptable welds were created at 850 °C, with small internal voids present at 800 °C.

Evaluation of microstructure after heat treatment revealed weld consistency from base metal to FSW/P stir zone in all but small areas.

Microhardness testing before heat treatment and after heat treatment revealed that:

▪ The FSP nugget was harder than the base material after FSP.
▪ After heat treatment, microhardness slightly varied from the base material to the nugget.
▪ Both post-heat treatment thermal exposure methods used exhibited similar observations.
▪ Upon 760 °C exposure, hardness increase was observed.
▪ Upon 871 °C exposure, a reduction in hardness was noted due mainly to coarsening of the γ′ precipitates (~200 ± 150 nm).
▪ All these observations were similar for both the base metal and the processed region.

Precipitate analysis revealed that:

▪ Mo-rich phases should be prevalent in 871 °C but not in 760 °C.
▪ M6C occurs from 780 °C to 1080 °C.

The tensile testing revealed that:

▪ Processed region tensile properties were similar to the base metal, resulting in a joint efficiency of 100%.
▪ All the cross-weld FSP samples failed in the base metal.
▪ The lowest local strain was observed in the processed region.
▪ The highest local strain was noted in the base metal.

Creep testing in 760 C revealed that:

▪ Grain boundary fracture and sample failure were primarily in the banded region.
▪ As FSP (before creep testing), mostly, MC and M_23_C_6_ precipitates in the banded region.
▪ Differences appear after creep testing, including:
  - Presence of Mo-rich phases along grain boundary (GB) and grain
  - interior.
  - Platelet like phase could be µ phase ((Ni,Co)7Mo6) or σ (FeCrMo.CrCo).

The authors summarized that:

- Ni-based superalloys can be successfully friction stir welded and processed
- Low rotation speeds and processing temperatures are important to successful FSW/P of nickel-based superalloys.
- FSW/P of solution annealed Haynes 282 significantly increases hardness in the stir zone. However, the standard two-step heat treatment yields similar hardness from the base material through the FSW/P regions.
- FSW/P of Haynes 282 plus post-solution anneal + FSW/P two-step heat treatment (standard fabrication process) yields FSW/P mechanical properties (creep and tensile) that can meet base material properties.
- FSW/P can heal defects in cast material.
- Inconel 617 is similarly processable by FSW/P but appears to require higher processing temperatures.

Sharma [176] studied FSW of a gamma prime (γ′) reinforced Haynes 282 nickel-based superalloy with a unique hemispherical tool. An efficiency of approximately 96% was attained in the as-welded state, which was then enhanced to nearly 100% following a two-step post-weld aging heat treatment. A very fine (~2 µm) grained microstructure was seen in the FSWed area compared to the coarse (~48 µm) grain base metal area. The carbides (MC and M_23_C_6_) and γ′ precipitates are recognized as the significant microstructural phases found in the welded area. The aging heat treatment led to the formation of the γ′ strengthening phase and changed the structure of the M_23_C_6_ carbide phase in the welded area from a continuous film to separate particles at the boundaries of the grains. No measurable tool wear was observed at least up to a welding distance of 200 mm.

Kippold et al. [177] performed FSW processing on both solid-solution strengthened and precipitation-hardened nickel-based alloys. All operations were performed using a W-25Re tool material featuring a narrow cone angle. Processing depths measured approximately 2 mm. Processing parameters for Alloys 625, 718, and Hastelloy X were defined in relation to spindle rotation and processing speed. Microstructure analysis showed a significant reduction in grain size due to processing. Additive friction stir processing involved depositing a precipitation hardenable Alloy 282 filler on an Alloy 600 substrate, followed by friction stir processing to alter the microstructure of the as-welded material. Gamma-prime precipitates existed in the processed layer, and significant enhancements in hardness (strength) could be obtained through an aging heat treatment compared to the welded and aged state

Several publications focus on comprehending the connection between weld bead geometry (penetration depth, width, and area) and welding parameters, particularly in P-GTAW and A-GTAW processes. The models that outline the connection between process parameters and fusion zone shape are created to enhance welding process parameters and achieve preferred bead geometry [163,173,178].

**Differences between various welding processes and their correlations with the weldability of Ni-based alloys.**

Shielded Metal Arc Welding (SMAW), Gas Tungsten Arc Welding (GTAW), Plasma Arc Welding (PAW), Gas Metal Arc Welding (GMAW), Submerged Arc Welding (SAW), Flux-Cored Arc Welding (FCAW), Electron Beam Welding (EBW), Laser Beam Welding (LBW), Friction Welding (FRW), and Friction Stir Welding (FSW) all have unique properties and applications for welding Ni-based alloys. SMAW, GTAW, PAW, GMAW, FCAW, and SAW are arc welding methods, while EBW, LBW, FRW, and FSW use different energy sources [179,180,181,182,183,184,185,186,187,188,189,190,191,192,193,194]. Ni-based alloys can be joined through different techniques, yet certain methods are preferable over others because of the alloys’ natural characteristics and susceptibility to cracking. Typically, gas-shielded techniques such as TIG (GTAW) and MIG (GMAW) are favored for welding the majority of Ni-based alloys. Processes like Friction Stir Welding (FSW) in solid-state welding are also effective for connecting these alloys [5,195,196].

Arc Welding Processes include:

- SMAW—uses a consumable, flux-coated electrode to create an arc, shielding the weld pool with the flux. It is suitable for various materials and positions, but has slower welding speeds and less precise control than other arc methods [183,184,186,187,188,189,190,191,192,193,194,197,198,199,200,201,202,203,204]. It can be utilized for welding Ni-based alloys, but precise choice of electrodes and settings is essential, particularly for precipitation-hardened alloys. Ni alloys typically melt at lower temperatures than steel, and require less heat input when welding compared to steel. Excessive heat application may result in problems such as the loss of alloying elements and heightened distortion. Choosing the appropriate electrode is essential for attaining the desired characteristics of the weld. Ni-based electrodes are frequently employed, and their formulation must be selected to complement the base metal and the intended welding properties. The SMAW process can affect the weld microstructure, with elements such as heat input and cooling rate impacting grain size and phase development. In certain situations, post-weld heat treatment might be required to enhance characteristics such as strength and toughness. In dissimilar welding of Ni-based alloys to different materials, selecting the appropriate electrode and welding parameters is even more essential. Particular attention must be paid to the risk of cracking or other problems at the junction. The SMAW process for nickel alloys may be susceptible to specific defects, including porosity or cracking. Effective welding methods, such as controlling parameters and preparing joints, are crucial to reduce these problems [196,205,206,207,208,209,210,211,212,213,214,215,216,217,218,219,220,221,222,223,224,225,226,227,228]
- GTAW—uses a non-consumable tungsten electrode and inert shielding gas to create a precise, controlled arc, often requiring a skilled operator. It is excellent for joining thin sections of Ni-based alloys and other materials [185,199,229,230]. The arc formed between the tungsten and the workpiece generates the necessary heat for melting the base materials. A filler metal can be added to the weld pool as needed. The process is shielded by an inert gas, typically argon or helium, to protect the molten weld from contamination. GTAW/TIG is known for its precision and ability to create high-quality welds, making it suitable for a variety of materials and applications. Commonly employed for welding thin and non-ferrous materials, such as nickel alloys, because of its accuracy and capacity to regulate heat input [196,198,229,231,232]. GTAW (Gas Tungsten Arc Welding) factors such as current, travel speed, and shielding gas greatly influence the microstructure and weldability of nickel (Ni) alloys. These factors affect grain size, phase development, and the occurrence of defects like cracks or porosity, which in turn influence mechanical characteristics such as strength, hardness, and ductility [233,234,235,236].
- PAW—is similar to GTAW but uses a more concentrated arc and a plasma gas to create a higher energy, more focused weld. This process can be automated and is used for thicker sections of Ni-based alloys [184,185,237]. It is suitable for welding nickel alloys, offering high energy concentration and deep infiltration [195,238,239,240]. The PAW process parameters strongly influence the microstructure and weldability of Ni-based alloys. These consist of welding current, welding speed, plasma gas flow rate, and the composition of the shielding gas. Tuning these parameters is essential for producing a quality weld that exhibits the required mechanical attributes and microstructural properties [195,196,239,241,242,243,244,245,246].
- GMAW—uses a continuous, consumable wire electrode and shielding gas to create the arc. It is a faster process than SMAW and GTAW, making it suitable for high-volume production [197,198,200]. Such an alternative, appropriate gas-shielded method is frequently employed for the thicker parts of nickel alloys [196,205,247]. GMAW (Gas Metal Arc Welding) of nickel alloys is affected by various process parameters that impact both the microstructure and weldability. Essential parameters consist of welding current, voltage, travel speed, shielding gas flow rate, and wire feed rate. Adjusting these parameters is essential for attaining the intended weld characteristics, including strength, ductility, and crack resistance [248,249,250,251,252,253,254,255].
- FCAW—utilizes a continuous, flux-cored wire electrode that provides shielding gas and filler metal. It is versatile, suitable for outdoor welding, and can be used on dirty or contaminated surfaces [183,184,185,202,203,220,256,257,258,259]. The weldability and microstructure of nickel-based alloys during FCAW are greatly affected by the composition of the filler metal and welding parameters. Factors such as current, voltage, travel speed, and shielding gas composition, along with the type and proportion of alloying elements in the filler wire, significantly influence the microstructure, mechanical properties, and crack resistance of the weld [250,260,261,262].
- SAW—uses a submerged arc, shielded by a blanket of flux. It is highly productive and suitable for thick plates of Ni-based alloys [186,187,188,189,190,191,192,193,194,256,263,264]. It is typically limited to solid-solution strengthened nickel alloys because of the risk of cracking in precipitation-hardened alloys [196,205,245]. The microstructure and weldability of Ni-based alloys in submerged arc welding (SAW) are affected by various essential factors, such as chemical composition, heat input, and welding conditions. These elements influence the solidification characteristics, grain patterns, phase development, and ultimately, the mechanical attributes and corrosion resistance of the weld [217,245,265,266,267].

Non-Arc Welding Processes include:

- EBW—uses a focused beam of high-energy electrons to melt and fuse metals, offering high precision and deep penetration, especially for Ni-based alloys [268,269,270]. It is very effective for welding nickel-based superalloys, creating deep, narrow welds with little distortion [5,195,271,272,273,274]. According to [245], EBW results in a joint that features deep penetration, a narrow fusion zone and heat-affected zone, along with minimal residual stresses thanks to a more uniform heat distribution, making EBW joints potentially better than conventional welded joints. In IN825, EBW joints are generally superior to GTAW welds, as the increased heat input resulted in a coarser microstructure and crack formation. However, when process parameters are not fine-tuned, achieving welds without cracks becomes challenging. This is especially difficult in single-crystal alloys, since GTA welds are typically free of cracks, whereas EBW welds contain stray grains that cause cracking. The unique aspects of the EBW process, including the need for a vacuum chamber, result in superior joint quality, even when contrasted with LBW. A comparison of laser and electron beam welding on thick joints composed of precipitation-strengthened Waspaloy and Udimet 720Li showed that the EBW joints exhibited lower porosity, though greater distortion was observed in the joints. The fusion zone also varies in shape and size. The factors influencing the process are categorized into two categories: beam characteristics (such as accelerating voltage and beam current) and joint properties (including welding speed, welding width, vacuum pressure, and preheating temperature). Statistical methods can be employed to merge the parameters efficiently. The combination of various values for beam current, accelerating voltage, welding speed, and beam oscillation showed that the accelerating voltage, along with beam current, significantly influences both the penetration and the width of the beam. The beam oscillation was not as significant; however, it influenced the crack susceptibility for Inconel 718 welded by EBW in the solution-treated state. The oscillation of the elliptical beam enhanced tensile strength and ductility at room temperature regardless of the heat treatment.
- LBW—utilizes a laser beam to melt and fuse metals, providing high precision and controlled heat input [200,268,269,275,276,277]. Like EBW, LBW provides high power density and accuracy for welding nickel alloys and other materials, especially in aerospace and various high-performance uses [195,278,279,280]. According to [245], the CO_2_ gas laser formerly used for welding tasks has been replaced by solid-state lasers today, like the Nb:YAG laser. Lately, fiber lasers have become popular due to their ability to enable rapid welding and their notable stability and precision. The fiber laser possessed enhanced melting efficiency and reduced the minimal heat input needed for complete penetration welds of Inconel 617 butt joints relative to the CO_2_ laser. Gas lasers have been effectively utilized for welding various superalloy joints, including Inconel 625, Inconel 718, and Nimonic. Examples of Nd:YAG laser applications include Inconel 718, Inconel 600, and NiTi shape memory alloy. The Nd:YAG laser demonstrated the ability to produce superior quality joints compared to various other laser types. A higher uniformity of the beads was noted in Inconel 718 joints welded with the Nb:YAG laser compared to the gas laser. This involves reduced residual stresses and the absence of microfissuring, which is a significant concern in laser welding. The Nd:YAG laser offers several benefits, including a high-energy absorption rate due to its low reflectivity, faster welding speeds, and reduced residual stress compared to CO_2_ lasers. These benefits render the Nd:YAG laser more practical and appropriate for on-site use and factory automation in high-volume manufacturing across various industrial applications. Obtaining smooth welds depends on the selection of laser parameters, the conditions before welding, and the heat treatments after welding.
  An appropriate adjustment of the laser welding settings ensures the quality of the welds. ANOVA analysis or DOE techniques enable the identification of optimal parameter combinations. The key factors include laser power and welding speed; however, additional elements are also important, including shielding gas flow rate, shielding gas pressure, focal position, and pulsation frequency.
  - FRW—creates a weld by applying pressure and rotating two pieces of metal together, producing a solid-state weld without melting. It is good for joining Ni-based alloys and other materials [200,281,282]. It can be realized as the Inertia Friction Welding (IFW) [1] or the Linear Friction Welding (LFW) [283]. The main factors influencing the friction welding of Ni alloys include friction pressure, upset pressure, burn-off length, rotational speed, friction duration, and forge pressure. These factors directly impact the heat input, plastic deformation, and ultimately, the microstructure and mechanical characteristics of the weld [284,285,286,287].
- FSW—uses a rotating tool to stir and consolidate the material being joined, producing a strong, solid-state weld without melting. It is suitable for joining Ni-based alloys and other materials [200,282,288,289]. It generates high-quality welds with little distortion, presenting a favorable alternative for connecting nickel alloys [5,290,291,292]. Friction stir welding (FSW) of Ni-based alloys and others is affected by various essential parameters that impact the microstructure and weldability. These factors encompass rotational speed, welding speed, tool shape, and axial force. Adjusting these parameters is essential for producing high-quality welds with preferred mechanical characteristics [293,294,295,296].

Various aspects affecting the choice of the welding type for Ni-based alloys include:

Ni-based Alloys and Welding—Ni-based alloys are known for their excellent corrosion resistance, high-temperature strength, and oxidation resistance. Welding these alloys can be challenging due to their tendency to crack or deform under high heat. The specific welding process chosen depends on the alloy composition, thickness, required weld quality, and application. For example, GTAW and EBW are often preferred for thin-walled Ni-based components requiring high precision, while FCAW and SAW are suitable for thicker sections [186,187,188,189,190,191,192,193,194,197,198,269,297,298,299,300].

Process selection needs to consider:

- Material thickness—thicker materials may require SAW or FCAW, while thinner sections may be better suited for GTAW or LBW [186,187,188,189,190,191,192,193,194,301].
- Weld quality requirements—high-quality welds for critical applications may require processes like GTAW or EBW, which offer more control and precision [199,269,302].
- Cost and production speed—GMAW and FCAW are often faster and more cost-effective for high-volume production [198,200,303].
- Operator skill and experience—some processes, like GTAW and EBW, require highly skilled welders [199,269].
- Application requirements—certain applications may demand the unique properties of a specific process, such as the deep penetration of EBW or the precision of LBW [269].

Difficulties and factors to take into account relative to weldability:

- Cracking—Ni-based alloys, particularly those that are precipitation-hardened, tend to experience cracking during welding, which can include solidification cracking, liquation cracking, and ductility-dip cracking [5,265,304].
- Segregation—alloying elements may separate during solidification, influencing the microstructure and characteristics of the weld [265,305].
- Heat input—its high rate can worsen cracking and microstructural problems, making it essential to regulate heat input during welding [306,307,308,309,310].
- Weld metal composition—must be thoughtfully selected to equal or surpass the characteristics of the base metal [306,311,312].
- Microstructure—The microstructure of nickel alloys, especially the existence of intermetallic phases and carbides, may affect weldability [195].
- Welding different metals—welding nickel alloys with different metals can pose extra difficulties concerning thermal expansion and phase changes [313,314,315].
- Particular Alloy Factors:
  o SSS alloys—typically demonstrate superior weldability compared to precipitation-hardened alloys [5,245].
  o Precipitation-hardened Alloys—demand meticulous choice of welding settings and materials to reduce the risk of cracking [245,304,316].
  o Cast Alloys—might be more prone to cracking because of larger grain sizes and uneven distribution [196].

Through thoughtful evaluation of the welding technique, alloy makeup, and risk of cracking, effective welds can be created in nickel-based alloys for differing uses [4,195,196,205,305].

**The effect of welding processes on the microstructure of Ni-based alloys**

Different welding processes, such as SMAW, GTAW, PAW, GMAW, SAW, FCAW, EBW, LBW, FRW, and FSW, can significantly impact the microstructure, primary phase, and secondary phase of joined parts made from Ni-based alloys. The specific effects depend on factors like welding parameters, alloy composition, and post-weld heat treatment, as well as in the case of the other alloys [194,317,318,319,320,321,322,323].

1. Welding Parameters and Their Impact [194,250,317,318,319,320,324,325,326,327,328,329,330,331,332,333]:

▪ Heat Input:—welding processes introduce significant heat into the weld zone, affecting the microstructure. Higher heat input can lead to coarser grain size and potentially the formation of undesirable phases.
• Cooling rate—the cooling rate after welding also influences the microstructure. Slow cooling can promote the formation of larger grains and phases, while rapid cooling can lead to finer microstructures and the formation of martensite.
• Filler metal—the type and composition of the filler metal used in welding can significantly impact the weld microstructure and phase composition.

2. Microstructure (not only for Ni-based alloys but also for the other) [194,237,317,318,319,320,334,335,336,337,338,339,340,341,342,343,344,345]:

- F-SMAW, GMAW, GTAW, PAW, FCAW—these processes can result in a microstructure consisting of the primary phase, such as the Ni-based alloy’s base matrix, and potentially secondary phases like carbides or intermetallic compounds. The specific microstructure depends on the alloy’s composition and welding parameters.
- EBW, LBW—these methods can create very fine-grained microstructures due to rapid cooling and high energy density.
- Friction Welding (FRW) and Friction Stir Welding (FSW)—these solid-state welding processes can produce microstructures characterized by deformation bands and grain refinement in the heat-affected zone.

Yu et al. [217] investigated the microstructure and hardness of weld metals used in liquid hydrogen storage tanks, with a focus on the effects of three welding methods: GTAW, SAW, and SMAW. They found that while GTAW and SMAW produced weld metals with similar microstructures, SAW generated significantly larger grains with a pronounced preferential orientation. The use of weaving techniques played a key role in shaping the solidification microstructures. Additionally, the hardness of the weld metal was comparable to that of the base material, with a slight reduction corresponding to increased grain size.

GTAW (Gas Tungsten Arc Welding) can affect the microstructure of nickel (Ni) alloys by [233,234,235,236]:

- Elevated current, which may cause excessive heat input, leading to a coarser grain structure in the weld zone (WZ) and heat-affected zone (HAZ), which might decrease strength and ductility. It may also elevate the likelihood of solidification cracking, particularly in alloys prone to cracking.
- Low voltage, which might not deliver adequate fusion, resulting in insufficient penetration and possible welding flaws. It may lead to a reduced grain size, yet it might not be enough to obtain the required mechanical properties.
- High travel speed, which lowers heat input, potentially resulting in a finer grain structure in the WZ and HAZ. Nonetheless, it could also decrease penetration and result in insufficient fusion.
- Leisurely pace of travel augments heat input, which may result in a rougher grain structure and heightened likelihood of solidification cracking. It also enables improved integration and infiltration.
- Protective gas in the form of Ar, frequently utilized for GTAW of Ni alloys, offering effective protection against environmental contamination. Alternatively, He provides a greater heat input than argon, which can be beneficial for thicker materials or challenging alloys to weld. Nevertheless, it may also elevate the risk of porosity if not adequately managed. Gas mixtures can also be customized to enhance welding characteristics for specific alloys and uses.
- The kind of tungsten electrode employed (e.g., thoriated, ceriated) can influence arc stability and the qualities of the weld pool.
- The electrode’s angle in relation to the workpiece can affect arc direction, penetration depth, and the shape of the bead.
- Heating Ni alloys beforehand can decrease thermal stresses and enhance weldability, particularly for thicker materials.
- Post-welding heat processing may be essential to enhance the microstructure and boost mechanical properties.

The PAW parameters can affect the microstructure of Ni-based alloys by [195,196,239,241,242,243,244,245,246]:

- Elevated current typically results in more heat input, which may create broader weld beads, greater penetration, and coarser grain structures in the Heat Affected Zone (HAZ).
- Increased welding speeds, which may lead to shallower penetration and finer grain structures because of lower heat input and quicker solidification periods.
- The plasma gas flow rate impacts the characteristics of the plasma jet, altering the shape of the weld pool, the penetration depth, and the overall quality of the weld. Increased flow rates may result in greater penetration but could also heighten the chance of porosity.
- Selecting a shielding gas (such as Argon, Helium, or combinations) affects the stability of the weld pool, its oxidation resistance, and the development of particular microstructural phases. It also influences the heat exchange and cooling speed of the weld.
- In powder-fed PAW, the rate of powder feed influences the dilution of the base metal and the makeup of the weld deposit. Accurate regulation of the powder feed rate is crucial for obtaining the targeted chemical composition and mechanical characteristics.
- The gap between the welding torch and the workpiece affects the heat spread and the properties of the weld pool. Keeping a steady torch standoff is crucial for even welding.
- In pulsed PAW, parameters such as frequency and current, which can be modified to regulate heat input, weld pool dimensions, and solidification speed, affect the microstructure and reduce the risk of cracking.

GMAW parameters can affect the microstructure of Ni-based alloys by [248,249,250,251,252,253,254,255]:

- A higher welding current typically results in deeper weld penetration, but may also raise the risk of cracking, particularly in nickel-based alloys, because of their sensitivity to thermal input.
- Welding voltage influences arc stability and the shape of the weld bead. Increased voltage may produce a broader weld bead, whereas decreased voltage can yield a thinner, more concentrated weld.
- Increased travel speeds lead to reduced heat input and can enhance the microstructure of the weld, though they may also cause incomplete fusion and defects if not managed correctly.
- Shielding gas prevents atmospheric pollutants from affecting the weld pool. The correct flow rate is vital for avoiding porosity and guaranteeing a clean weld.
- The speed of the wire feed directly regulates the quantity of filler metal introduced to the weld, affecting both the size of the weld and the chemical makeup of the weld metal.
- Pre-heating the workpiece, which can lower thermal stresses and avoid cracking, while keeping proper interpass temperatures, aids in controlling heat input during multi-pass welding.

The microstructure of GMAW welds in nickel-containing alloys is affected by elements such as cooling rate, solidification characteristics, and the incorporation of alloying components. Typically, fine-grained microstructures are preferred for enhanced mechanical characteristics.

FCAW parameters can affect the microstructure of Ni-based alloys by [250,260,261,262]:

- Increased current and voltage, which typically boost heat input, resulting in a larger weld pool and greater penetration. High current may result in the loss of alloying components and increased porosity. Higher voltage can enhance arc stability and encourage grain development.
- Higher travel speeds decrease heat input, leading to faster cooling and possibly increased hardness. Inadequate travel speed can result in excessive heat input, causing warping and fracturing.
- The selection of shielding gas, which can impact weld penetration, bead formation, and the development of oxides and nitrides, affects the microstructure and mechanical characteristics.
- The makeup of the flux-cored wire, especially the inclusion of elements such as Ni, Cr, Mo, and Nb, can considerably influence the phase changes during cooling, thereby impacting the weld’s strength, hardness, and resistance to corrosion.
- Heat input, depending on current, voltage, and travel speed, is essential in influencing the microstructure and characteristics of the weld. Maximizing heat application is crucial for attaining the preferred penetration, grain structure, and phase arrangement.
- Preheating, which helps decrease thermal stresses and enhances microstructure while keeping the right interpass temperature, is vital to prevent excessive cooling rates or overheating.

The microstructure of Ni-based alloys during SAW can be affected by [217,245,265,266,267]:

- Alloying elements such as Nb, C, and others are vital in influencing the solidification process, phase changes, and the emergence of secondary phases like NbC and Laves phases. These stages can greatly influence the ductility and overall effectiveness of the weld.
- Carbon concentration substantially impacts weldability, influencing phase development and microstructure.
- Ni concentration enhances strength and low-temperature impact toughness by encouraging the development of acicular ferrite.
- The heat supplied during SAW influences the grain size and distribution, phase changes, and the development of acicular ferrite. Increased heat input can result in larger grains and a greater proportion of acicular ferrite, which might enhance hardness and tensile strength, yet could reduce impact toughness and corrosion resistance.
- The rate of welding impacts the cooling rate and solidification duration, which, in turn, affect grain size and microstructure.
- Welding voltage and current influence the energy input and melt pool properties, thereby impacting the weld bead dimensions and depth.
- The makeup of the shielding gas, especially the levels of oxygen and carbon dioxide, can impact oxide formation and alter the microstructure of the welded metal.

The mechanical properties are greatly influenced by the grain size and structure. Microstructures with fine grains typically enhance tensile characteristics, whereas structures with coarse grains can improve creep and fatigue resistance at higher temperatures. The process of solidification and the cooling that follows can result in different phase changes, such as the development of austenite, ferrite, and martensite. The nature and arrangement of these phases affect the mechanical characteristics of the weld. The weld’s corrosion resistance can be influenced by its microstructure, grain boundaries, and the existence of specific phases. For instance, certain inclusions or precipitates can serve as locations for the onset of corrosion.

According to [245], Hastalloy X joints made with a pulsed Nd:YAG laser resulted in much lower porosity compared to those made with a continuous fiber laser. Continuous waves (CW) mode results in lower seam quality compared to pulse waves (PW), leading to adverse effects on the mechanical properties of the welded parts. Decreased porosity occurs in the PW joints, where the local CO micro-voids in the melting pools are efficiently eliminated by the following pulse. Raising the frequency of the laser beam while simultaneously enhancing the stirring in the melting pool allows for even reduced porosity levels. To minimize the problem of welding fractures in Ni-based superalloys. A usable strategy when facing high crack susceptibility is to implement an appropriate heat treatment before welding. This results in a more uniform distribution of precipitates, minimizes thermal gradients, and changes the hardness of the base material. Along the fusion line connecting the FZ and HAZ of the laser welded polycrystalline GH909 superalloy, it was observed the formation of γ/Laves eutectic constituents within the laser welds: the solidification process and the mechanisms for developing Laves eutectic constituents and grain boundary liquation in the FZ and HAZ, which are regarded as the main factors contributing to liquation cracking. The liquation of Nb-rich MC carbides in welded Ni-based superalloys is identified as a major cause of HAZ microfissuring. The connection of HAZ cracking with the liquation of grain boundary constituents like γ–γ′ eutectics, carbides, Cr–Mo borides, and Ni–Zr intermetallics also occurred. The toughness of the base material and the dissolution of the aforementioned grain boundary elements significantly influence the crack vulnerability of the joints. Grain boundary liquation is said to arise from the heat introduced during welding and the resulting grain coarsening. In the GH909 specimens welded with a fiber laser, the joint microstructure showed that larger grains are more susceptible to precipitation and segregation, increasing liquation of lower melting components at elevated temperatures. Lowering the laser power and, to a lesser extent, the welding speed helps prevent liquation cracking. Formation of a liquid film on the grain structure in the HAZ was similarly noted for IN738 cast superalloy. Notably, the occurrence of this harmful phenomenon was not seen in the Haynes282 autogenously welded by a single pass CO_2_ laser beam: the lack of the relevant γ–γ′ eutectic transformation components was attributed to the altered primary solidification route due to C addition, while Ti and Mo rich MC-type carbide are present in the FZ. For Inconel 800, it was found that increasing the scanning speed allows for grain refinement and slight precipitation of the Laves phase, as this reduces heat input and improves the cooling rate. Concerning the solidification cracks, they typically occur at grain boundaries and are thus influenced by the grain boundary energy (γgb). The latter is influenced by the misorientation angle (θ) and alloying elements, affecting the crack susceptibility. Several strategies were suggested to prevent solidification cracking, including grain refinement and laser oscillation, which can induce more turbulence in the molten pools, promoting fine-grain nucleation. Significant applications of LBW include turbine blades. To enhance the creep resistance of Ni-based superalloy turbine blades for high-temperature uses, single-crystal or directionally solidified microstructures are frequently assessed alongside the impact of crystallographic orientation on the alloy’s susceptibility. Fusion welding of single-crystal alloys presents challenges concerning the potential presence of stray grains in the fusion zone, which are frequently linked to cracking. For instance, several stray grains were noted in the center of the FZ of a PWA 1480 single crystal weld produced by pulsed laser, and a crack along the high-angle boundaries of stray grains can be seen. The formation mechanism of stray grains is indicated by the constitutional supercooling occurring in front of the advancing solidification front. The formation of stray grains depends on the laser parameters, as this issue can be mitigated by lowering the laser power and the welding speed.

According to [245], during EBW, the grain size influencing crack susceptibility is primarily impacted by welding speed and heat input. The usual microstructure seen in EBW Ni-based alloy joints features equiaxed dendrites in the center of the FZ and a shift from cellular to columnar dendrites close to the HAZ. Typically, the microstructure is consistently finer than that achieved with alternative welding methods, which theoretically results in greater hardness and tensile strength. However, other elements could significantly influence the outcome, like the quantity of δ-precipitates resulting from thermal treatments. The grain refinement occurs due to the reduced heat input that defines the EBW process compared to alternative methods. The detailed microstructure was also noted in single-crystal Ni-based superalloy joints, where the microstructure changes to polycrystal following EBW. Cracks due to solidification and liquation in the FZ and HAZ are primarily linked to the formation of phases like γ–γ′ eutectic, Cr-Mo boride, Ni-Zr intermetallic, MC carbide, and γ′ phase in IN738. Liquation cracking in solutionized K465 nickel-based superalloy is additionally promoted by the segregation of elements like Ti, Nb, and Al, along with the previously mentioned phases. B segregations are regarded as contributing factors to the susceptibility of cracks in Ni superalloy 718. Raising the beam current is advantageous because the increased heat input helps reduce residual stresses associated with the cooling of the welds. Additionally, raising the beam current enhances the backfilling process, which involves the filling of a crack caused by the molten material in the fusion zone that retreats due to capillarity. Crack vulnerability is influenced not just by the heat input, but it also increases when the welding speed decreases. The quantity and length of transverse cracks rise as welding speed decreases due to (i) the accumulation of certain alloying elements at grain boundaries, resulting in a larger volume of liquid film, and (ii) the conversion of low-angle grain boundaries (LAGB) into high-angle grain boundaries (HAGB). This leads to increased vulnerability to cracking. Additionally, the average grain size of the joint reduces as the welding speed increases, which alters the mechanical properties, including the microhardness of the fusion zone. In addition to optimizing process parameters, various strategies are suggested to minimize cracking susceptibility by (i) managing the chemical composition in the weld, (ii) adding a deposited layer, and (iii) utilizing pre-heating or post-welding heat treatments.

The FRW parameters can affect the microstructure of Ni-based alloys by [284,285,286,287]:

- Greater rotational speeds result in elevated friction and heat production at the weld interface. Excessive speed can lead to grain coarsening and diminish weld strength.
- The length of friction directly affects the level of heat generated. Extended friction durations can result in excessive heat and possibly unfavorable microstructural alterations.
- Friction pressure governs the contact force between the moving and fixed components. It affects the speed of heat production and plastic deformation. According to a study published by Springer, greater friction pressure typically results in improved bonding and enhanced tensile strength. Used following the friction stage, this pressure solidifies the weld and guarantees a strong joint. The strain rate during the welding process is also linked to forge pressure.
- Length of burn-off characterizing the quantity of material that is “removed” or displaced during the friction process. It quantifies plastic deformation and material loss, and is affected by friction duration, pressure, and rotational speed.

Employing Inertia Friction Welding (IFW), Tong et al. [346] joined large-diameter hollow bars composed of Inconel 690 and 316LN successfully. A significant mechanical mixing area was discovered at the welding interface. The elemental diffusion layer was discovered within the “wrinkles” of the mechanical mixing area. A small amount of C elements gathered on the friction and secondary friction surfaces. The joints’ tensile strength and impact toughness were enhanced as the total welding energy input increased. Elevating the friction pressure may result in a uniform refinement of the grain across all sections of the joint, thereby improving the mechanical properties of welded connections. The Kirkendall effect in the welded joint resulted in enhanced metallurgical bonding at the welding interface near the Inconel 690 side in comparison to the 316LN side.

Geng et al. [283] examined the microstructural changes and thermo-mechanical field distribution during the Linear Friction Welding (LFW) of GH4169 and FGH4096 superalloys. They discovered that the acoustically distinct joint of FGH4096 and GH4196 created through linear friction welding was distinguished by separate curled flash extending from all four sides of the interface and was free of defects in the weld zone. The amount of deformation in GH4169 superalloy exceeded that of FGH4096, leading to a greater flash size post-welding. The welded area included the friction interface zone (FIZ), thermally and mechanically affected zone (TMAZ), and base metal (BM). Complete dynamic recrystallization took place in the FIZ, resulting in a considerably refined grain size, along with total dissolution of strengthening precipitates on both sides of the weld line due to elevated temperature and increased plastic deformation. An incomplete dynamic recrystallization (DRX) with partially dissolved strengthening precipitates was detected in TMAZ, leading to the formation of a softened area as indicated by the micro-hardness changes. The weld’s maximum temperature attained was 1237 °C, with a more significant temperature gradient observed on the GH4169 superalloy side. The predicted plasticized zone width from the interface measured 1.65 mm on the FGH4096 side and 2.05 mm on the GH4169 superalloy side, aligning well with the microstructure observations.

FSW parameters can affect the microstructure of Ni-based alloys by [293,294,295,296]:

- Rotation speed impacts the thermal input and material movement during FSW. Elevated speeds can result in greater heat input, which may enhance grain refinement in the stir zone (SZ), but could also lead to issues such as voids or cracking if the speed exceeds certain limits. Reduced speeds can cause inadequate heat generation, resulting in poor mixing and weak connections.
- Welding speed establishes the tool’s dwell time in the weld area, consequently affecting the heat input and material blending. Increased welding speeds may lower heat input, which could result in incomplete mixing and flaws, whereas decreased speeds can raise heat input and potentially cause excess material flow and warping.
- Tool configuration, including the length of the pin, the diameter of the pin, and the diameter of the shoulder, all influencing the flow of the material and the generation of heat. The design of tools is essential for ensuring adequate material blending and reducing flaws. For instance, a wider shoulder diameter can enhance heat input and material flow, whereas a longer pin can influence the depth of the stirred region.
- Axial load regulates the force exerted by the tool on the workpiece, affecting material flow and the occurrence of defects. Increased axial force may enhance material mixing and consolidation; however, too much force can result in tool wear or material ejection.

3. Primary Phase [194,317,318,319,320,336]:

- Ni-based Alloys—the primary phase in Ni-based alloys is typically the Ni-rich matrix, which can be either a face-centered cubic (FCC) phase or a combination of FCC and other phases depending on the alloy’s composition.
- Welding effects—welding can alter the primary phase by promoting phase transformations or precipitating secondary phases within the matrix.

4. Secondary Phase [194,317,318,319,320,347,348,349,350,351]:

- Carbides—such as MC or MX, can precipitate in the weld zone, especially in alloys with high carbon content.
- Intermetallic Compounds—such as sigma phase (σ) or chi phase (χ), can also form depending on the alloy composition and welding conditions.
- Oxides—depending on the welding process and atmosphere, oxides can also form in the weld zone, particularly in GTAW or PAW.

5. Specific Welding Processes and Their Effects [186,187,188,189,190,191,192,193,194,320,349,352,353,354,355,356,357]:

- SMAW, GMAW, GTAW—these are the most commonly used arc welding processes. They can induce varying degrees of heat input, affecting the microstructure and phase transformations in Ni-based alloys.
- FCAW—this process, similar to SMAW and GMAW, can be used to weld Ni-based alloys, but its flux composition and welding parameters can influence the weld microstructure.
- EBW, LBW—these welding methods offer high energy density and can result in rapid solidification and fine-grained microstructures.
- FRW, FSW—these solid-state welding processes can produce microstructures with deformation bands and grain refinement in the heat-affected zone.

In summary, the welding process significantly influences the microstructure, primary phase, and secondary phase in Ni-based alloys. Understanding the effects of different welding parameters and processes is crucial for controlling the microstructure and achieving desired mechanical properties in Ni-based alloy welds [194,246,317,318,319,320,323,358].

**Selective laser melting**

Selective Laser Melting (SLM) is an additive manufacturing technique employing powder bed fusion to produce Ni-based alloys. It requires precisely melting metal powder using a high-energy laser to create intricate 3D shapes. SLM is a flexible technique for producing Ni-based superalloys, particularly for parts that need high-temperature durability [359,360].

Process is commonly characterized by [359,360,361,362,363,364,365,366,367,368,369]:

- Powder Bed: A layer of Ni-based alloy particles is distributed over a fabrication platform.
- Laser Scanning: A laser beam precisely melts the powder based on a 3D model, forming the intended layer.
- Layer-by-Layer Construction: The procedure continues, layer by layer, until the finished component is achieved.
- Inert Atmosphere: SLM is frequently conducted in an inert atmosphere (such as nitrogen or argon) to avoid oxidation.
- Essential factors for SLM for Ni-based alloys:
- Microstructure [359]: SLM can create equiaxed or columnar grain structures based on process parameters such as scanning speed, hatch distance, and laser energy.
- Process Specifications [359]: Enhancing these parameters is essential to reduce flaws such as cracks, pores, and construction defects.
- Mechanical Characteristics [359,360]: The SLM process affects the resulting microstructure and mechanical properties.
- Density [364,370]: Attaining high density is essential for the part’s structural integrity.
- Porosity [370,371]: Porosity can arise from trapped gases or incomplete melting, and process parameters can be adjusted to limit it.
- Advantages of SLM for Ni-based alloys include:
- Geometries that are intricate: SLM allows for the fabrication of complex forms and patterns that are challenging to achieve using conventional techniques [372].
- High-Temperature Efficiency: Ni-based alloys are employed in applications that demand high-temperature capabilities, and SLM is an appropriate technique for producing these parts [360,373].
- Repairg and Restoration: SLM can be utilized to fix or rejuvenate Ni-based parts, like those found in gas turbine engines [359,373].
- Exemplary of Ni-based alloys applicable to SLM comprise:
- Inconel 718: A Ni-based superalloy commonly utilized in gas turbines [360,370].
- NiTi: high-quality NiTi alloys can be in situ synthesized with reduced manufacturing defects, the formation of Ni_4_Ti_3_ precipitates, and improved pseudoelasticity and microhardness [374].
- CMSX-4: A different Ni-based superalloy utilized in gas turbines [359,375,376].
- Ni-Nb Alloys: Studies have also concentrated on the SLM of binary Ni-Nb alloys as a substitute for Ni-containing superalloys [377].

### 3.1. The Weldability of CP Ni

Overall, CP Ni alloys exhibit good weldability, superior to that of SSS and PS Ni alloys. Nonetheless, alloys belonging to this category may experience particular weldability problems that can be avoided if their origin is accurately recognized.

***Porosity***

Ni 200 and Ni 201 are the most commonly utilized CP Ni alloys when welding is necessary for fabrication. The primary concern regarding weldability that may arise when joining these alloys is porosity in the weld metal. The formation of porosity occurs due to gases that become trapped within the weld metal. They may result from chemical reactions in the weld metal or be due to H_2_ absorption from moisture, oil, grease, and contaminants on the surfaces of the filler and base metals. Porosity problems can be especially prominent in cases of welding without filler metal.

Actions to reduce porosity entail choosing appropriate filler metal, selecting the right shielding gas along with its flow rate, and ensuring the cleanliness of both filler and base metals.

Common filler wires utilized for welding Ni 200 alloy include deoxidizing agents (up to 1.5 wt.% Al and 2.0–3.5 wt.% Ti, ERNi-1) that diminish porosity by generating oxides and nitrides, [4].

Sufficient gas or flux shielding while welding can also help avoid porosity defects. This essentially indicates a smooth flow of the shielding gas. Larger diameter gas nozzles and gas lenses are advised [158]. Inadequate gas flow rate may lead to increased metal oxidation, while an excessive flow rate can create turbulence, trap air, or result in rapid cooling of the weld pool.

It is frequently necessary to protect the root side of the weld joint to prevent oxidation and porosity from occurring on the backside. Any foreign substance should be deemed harmful when exposed to heat unless demonstrated otherwise. Maintaining cleanliness is crucial for the effective welding of Ni alloys [4,158].

***Craking issues***

CP Ni alloys are only prone to hot cracking in the weld metal throughout the solidification process.

**Cracking during the solidification of weld metal in CP Ni**

This kind of cracking happens during the solidification process and is caused by welding thermal stresses applied to the liquid film found at the grain boundaries in the weld metal that is solidifying. Cracking takes place through the liquid film during the final phase of solidification when accumulated thermal stresses can no longer be absorbed. The tendency of Ni-based alloys to experience cracking during weld metal solidification is influenced by their solidification temperature range, the quantity and distribution of terminal solidification liquid film at grain boundaries, and the extent of local strain at the conclusion of solidification. A broader solidification temperature range raises the likelihood of solidification cracking as the weld metal cools [378].

Elements with low solubility in the Ni matrix, including P, S, B, and Si, tend to encourage cracking during the solidification of weld joints [3,379,380,381]. These components partition to the liquid at the solidification grain boundaries and create a low-melting-point liquid layer. Due to these factors, the concentrations of P, S, B, and Si in Ni alloys are maintained at a very low level.

Due to the limited solidification temperature range, CP Ni alloys are much less prone to cracking than the SSS and PS alloys [379].

Certain CP Ni alloys like Ni 205, 211, and 233 were created for particular electrical uses, whereas Ni 253 and 270 are ultra-pure versions.

The extremely narrow solidification temperature range of ultra-pure Ni alloys effectively removes the mushy zone throughout their solidification. Consequently, the likelihood of cracking during solidification is nearly eradicated. They can become highly vulnerable to hot cracking if the concentration of trace elements (notably S) in their chemical makeup is even slightly raised. The presence of Mn can be advantageous as it easily reacts with S to create MnS globules, thereby decreasing the wetting of grain boundaries [3]. The base and filler metals must remain clean prior to and during welding to prevent contamination and minimize the risk of cracking.

To summarize, various mechanisms of cracking in various Ni-based alloys are presented in Table 35.

### 3.2. Weldability of Ni Alloys

All traditional welding methods are suitable for welding Ni and its alloys, and corresponding welding consumables can be used. Ni and its alloys share many characteristics with austenitic stainless steels; thus, the welding techniques are also comparable. Ni, on the other hand, has a thermal expansion coefficient that is lower than that of stainless steel, so the distortion and measures for controlling distortion are comparable to those of carbon steel. The most critical cracking issue with Ni alloys is hot cracking occurring in the weld metal or near the fusion line in the HAZ, with the latter being more common. The primary cause of this issue is S, though P, Pb, Bi, and B also play a role. Typically, cracking in both weld metal and the heat-affected zone (HAZ) is due to contamination from grease, oil, dirt, and other residues resulting from insufficient cleaning; instances of excess sulfur in the base or filler metals leading to issues are uncommon. Before welding, it is essential to machine or vigorously brush the stainless steel wire and then perform a comprehensive degreasing using an appropriate solvent, aiming to complete the welding within approximately eight hours to minimize the chance of contamination. All heat treatments should be performed with S-free fuel or utilizing electric furnaces. Parts that have been in operation and necessitate weld repair might need to be ground or machined before degreasing to eliminate any contaminants that have been embedded in the surface in or near the weld repair zone. If mechanical wire brushing occurs after the degreasing process or while welding, the compressed air from air-powered tools includes both moisture and oil, potentially leading to re-contamination of the cleaned surfaces [306].

Porosity may pose an issue in Ni alloys, primarily due to nitrogen. Even just 0.025% nitrogen can create pores in the weld metal as it solidifies. Fairly light draughts can disturb the gas shield, leading to atmospheric contamination and causing porosity. It is essential to ensure that the welding area is adequately protected, especially in site welding situations. In gas shielded processes, it is essential for the gas purity and the effectiveness of the gas shield to be optimal. Gas hoses must be inspected for leaks and damage at regular intervals, and when using the TIG process, a ceramic shroud of maximum size should be utilized along with a gas lens. It is obvious that gas purging of the root is crucial when applying a TIG root pass. Adding a small quantity of H_2_ (up to 10%) to the Ar shielding gas has been shown to alleviate the issue. Start and end porosity pose an issue during MMA welding. The weld initiation must be performed by welding over the arc strike location again, remelting any porosity created by inadequate gas shielding at the beginning of the weld. Attention should also be given at the end of the weld, with a shorter arc length and a slight increase in travel speed to minimize the size of the weld pool. Oxygen can also lead to porosity under specific conditions when it reacts with carbon in the weld pool to create carbon monoxide. Producers of consumables typically address this issue by guaranteeing the presence of adequate deoxidants (mainly manganese, aluminum, and Ti) in the filler metal [306].

A characteristic frequently observed in Ni alloys is the development of a thick and sticky scum on the surface of the weld pool. This may be challenging to eliminate and can lead to inclusions and insufficient inter-run fusion if not eliminated before applying the next pass. Wire brushing often proves inadequate for eliminating this layer, making it essential to grind the weld surface instead. The weld pool, along with this surface film, is also slow and does not flow freely as with a carbon or stainless steel. This could lead to a bumpy and extremely convex weld bead and a bad toe blend unless the welder adjusts the weld pool to prevent these flaws. While stringer beads can be utilized, a minor weave can help the weld metal to better wet the side walls of the preparation. Furthermore, weld preparations should be broad enough to allow the welder to manage and guide the weld pool; a recommended included angle for V butt welds is between 70 and 80°. A U preparation with an included angle of 30 to 40° is permissible and, while it may cost more to machine than a V preparation, could be less expensive in total since the quantity of filler wire needed might decrease, depending on the thickness of the material. In TIG welding, incorporating H_2_ into the shielding gas (up to 10% H_2_ in Ar) has also proven advantageous in lowering the surface tension of the weld pool [306].

Another feature of Ni alloys is that the level of penetration is lower than that of carbon or stainless steel. Raising the welding current will not enhance penetration. This suggests that the root face thickness in single-sided full penetration welds ought to be thinner than that of stainless steel. It is advised that the thickness of the root face should not exceed 1.5 mm in a zero-gap TIG butt weld. Detachable backing strips are extremely helpful for managing the shape of root beads. These can be crafted from Cu, stainless steel, or a Ni-based alloy. Backing strips made of carbon or low-alloy steel should be avoided [306].

While the weldability of Ni and its alloys is typically favorable, factors such as composition, metallurgical structure, and heat treatment or service history influence their behavior during welding. Forged, finely textured parts exhibit superior weldability compared to cast materials, which frequently contain considerable levels of segregation. Coarse grains can cause micro-cracking in the HAZ, so it is advisable to avoid high heat input. All alloys are ideally welded in the annealed or solution-treated state, especially the PS alloys like Inconel 718 [306].

Cleanliness is very important from the point of view of welding, especially the elimination of all sulfur-containing compounds. Regarding defect-free welding of Ni and its alloys, this cannot be overstated. In addition to sulfur, there are various other materials that can cause embrittlement in Ni alloys when subjected to elevated temperatures. Included in this group are Pb, P, B, and Bi. These substances can be found in oils, grease, cutting fluids, paints, marker inks, temperature indicating crayons, etc.; it might not be feasible to eliminate their use during manufacturing, so it is crucial that they are cleaned off if the part is to be welded, heat treated or placed in high temperature service. Fuel gases often have sulfur, and it might be essential to utilize radiant gas heaters or electric elements for localized heating or in heat treatment furnaces. Ni alloys can be welded using various conventional arc welding and power beam techniques, with the most frequently used methods being TIG or MIG utilizing pure Ar, Ar/H_2_, or Ar/He mixtures as shielding gases, and MMA where basic flux coatings deliver optimal properties. Nevertheless, the use of Ar/He mixtures only yields notable advantages in penetration and enhanced fusion when He is present in amounts exceeding 40%. Submerged arc welding is limited to welding SSS alloys with basic fluxes. Compatible welding consumables can be found for nearly all Ni alloys [453].

#### 3.2.1. Weldability of SSS Ni Alloys

Although SSS Ni-based alloys are relatively simple to weld, maintaining the strength of the weld joint and its corrosion resistance can be challenging. Welding SSS Ni-based alloys may also lead to significant cracking defects. Numerous cracking defects are related to metallurgy and linked to a reduction in ductility. Ductility reduction can result from the existence of a liquid phase or from embrittling effects at elevated temperatures. According to the fundamental mechanism and site of occurrence, hot cracking can be categorized into weld metal (WM) solidification cracking (or fusion zone cracking), partially melted zone (PMZ), and heat affected zone (HAZ) liquation cracking1, as well as ductility-dip cracking (DDC) [3,454].

Welding alloy B-2 can be accomplished using TIG, MIG, and coated electrode methods. Welding dissimilar metals, specifically alloy B-2 to alloy B-3^®^, can be achieved with AWS ERNiMo-10 for GTAW and GMAW processes, as well as ENiMo-10 for SMAW applications. The workpiece must be completely cleaned with acetone or other appropriate solvents to make sure that all impurities and markings have been eliminated. Grinding before welding can be performed with a clean alumina wheel that has not been used on any Fe-containing materials. Pre- or post-heat treatment processes are unnecessary unless the workpiece has been welded or formed. A complete solution anneal prior to welding is necessary for components that have been shaped [21].

Alloy B-3 can be welded using various conventional techniques; however, oxyacetylene and submerged arc welding methods are recommended for items intended for use in corrosive environments. Extra care must be exercised to prevent excessive heat input [21].

Inconel 600 is suitable for welding and soldering. Any conventional welding technique is applicable [34].

Incontel 601 belongs to weldability group 43 (ISO 15608), and brazing P-number 111 (ASME section IX) [35]. For Inconel 601, it is recommended to use any gas-tungsten arc welding, GTAW, for applications involving temperatures above 1150 °C or at lower temperatures under exposure to H_2_S or SO_2_ [33].

Inconel 625 can be joined to Inconel and stainless steel utilizing standard TIG welding methods for stainless steel. Inconel weldments possess great strength and exhibit excellent resistance to corrosion and oxidation. Welding Inconel may seem “dirty,” with the weld pool possibly appearing beneath a “skin” and lacking clarity. This type of weld pool is relatively “sluggish” (not flowing easily) when compared to steel or stainless steel. These traits may cause flaws such as convex weld beads and generally produce a weldment that appears “coarse” in comparison to stainless steel [455].

The Inconel 718 alloy possesses excellent welding properties, ranking among the most weldable Ni-based superalloys. It is nearly entirely immune to cracking after welding. It is due to its main precipitate, the γ″, which forms at a significantly slower rate than the γ′. This enables alloy 718 to be heated to the solution temperature range without experiencing aging and the associated strain-age cracking. Alloy 718 requires 10,000 min of exposure to approximately 900 °C to exhibit HAZ cracks, whereas Waspaloy does so in just 5 min! The alloy is classified as group 43 under ISO 15608. Welding Inconel 718 alloy using the ERNiCr-3 alloy as the filler material instead of ERNiFeCr-2 reduces the tensile properties by approximately one-third. Brazing at temperatures of 1010 °C or higher leads to grain enlargement and a related decline in stress-rupture characteristics, which cannot be restored through later heat treatment. The vacuum brazing at 10-4 torr in a cold-walled vacuum furnace yielded optimal outcomes for brazing alloy 718 components of air diffusers used in aerospace turbine engines. Before brazing, all joining surfaces underwent Ni plating to a thickness of 0.015 mm, in compliance with AMS 2424. A 0.11 mm BNi-2 braze filler metal tape was initially positioned, and following assembly, an extra braze slurry of BNi-2 filler metal was spread over all joints [37]. Gas-tungsten-arc welding is applicable for Inconel 718 [33].

Sonar et al. [456] investigated how welding methods (GTAW, EBW, and LBW) influence the microstructural features and mechanical properties of Inconel 718 welds. They indicated that the Inconel 718 alloy was prone to metallurgical issues during welding, including hot cracking, HAZ liquation cracking, segregation, and the formation of Laves phase in the fusion zone (FZ). The GTAW Inconel 718 welds displayed lower tensile properties (70% joint efficiency) because of the development of a more coarse networked Laves phase in the fusion zone. The EBW and LBW methods were effective in managing the Nb segregation (10–12%) and the formation of Laves phase (4–6%) in the FZ of Inconel 718 welds. The GTCAW process revealed considerable improvement in the tensile characteristics of Inconel 718 joints, enhancing joint efficiency to 99.20%. The performance of GTCAW joints outperformed other types of GTAW joints and was comparable to costly EBW and LBW joints. Therefore, demonstrating its feasibility in aerospace uses.

Kumar et al. [457] examined the effects of a newly created tri-component oxide flux (Cr_2_O_3_, FeO, and MoO_3_) on weldability, bead shape, temperature fluctuations in the weld pool, and the mechanical strength of Inconel 718 welded connections. They discovered that the flux mixture greatly enhanced the penetration depth and aspect ratio by nearly 200% compared to traditional TIG welding. The arc narrowing due to the new oxide flux increased the heat density and the weld pool temperature of the joints. The presence of trapped oxide particles in the alloy significantly enhanced both the hardness and tensile strength of the joints.

INCONEL alloy G-3 exhibits excellent weldability and requires no heat treatment after welding to regain corrosion resistance. These welded metals show corrosion resistance comparable to that of the base metal [33].

Incoloy 800 is categorized as group 45 per ISO 15608. In accordance with ASME, section IX, the p-number for brazing is 111 [38].

According to [458] the weldability of Incoloys 800H and 800HT is as excellent as that of alloy 800. Both are typically utilized for applications that demand high creep-rupture strength and should be connected using welding materials that possess appropriate strength attributes for the intended service temperatures. For temperatures reaching 790 °C, the INCO-WELD A Electrode is employed for shielded metal arc welding, while INCONEL Filler Metal 82 is utilized for gas shielded welding. Filler Metal 82 is likewise utilized alongside INCOFLUX 4 Submerged Arc Flux for submerged-arc welding of INCOLOY alloys 800H and 800HT. For service temperatures exceeding 790 °C, the ideal welding product selection relies on the particular service temperatures in question and the characteristics required in the welded joint. For uses that demand maximum strength and resistance to corrosion, INCONEL Welding Electrode 117 and INCONEL Filler Metal 617 are advised. To simplify the qualification of welders, the ASME Section IX classifies INCOLOY alloys 800H and 800HT as “P45”. The welding materials Incoloy 800H and 800HT, which were previously suggested for joining, hold an ASME Section IX “F” classification of “F43”.

Incoloy 825 is classified in the 45th weldability group as per ISO 15608. For brazing, the p-number is identified as 111, as per ASME/AWS standards. If forged components are meant to be welded and will function in an environment prone to intergranular corrosion, they should undergo a stabilizing anneal to avoid sensitizing the heat-affected zone [43].

Incoloy 925 has good weldability. The MIG and TIG welding methods are recommended. For optimum results, welded parts should be annealed before welding and solution-treated and aged after welding [44].

Alloy 926 can be welded with:

- Solid solution welding materials: ERNiCrMo-10, ENiCrMo-10;
- Clad Arc Welding Rod: E NiCrMo-10;
- Unclad arc welding electrode: ENiCrMo-10;
- TIG wire: ERNiCrMo-10, ENiCrMo-10;
- MIG/MAG wire: ERNiCrMo-10, ENiCrMo-10.

Such welding consumables are compatible with Incoloy 926 base material and provide high strength of welded joints [459].

Nimonic 86 exhibits good weldability. The best weld is obtained with the TIG process, using matching composition filler wire [46]. According to [460], the closest alloy enriched with the crucial elements (Ni, Co, Cr, Mo) can also be utilized. Every weld bead ought to be somewhat convex. Preheating is not required. Welding surfaces should be clean and devoid of oil, paint, or crayon marks. The cleaned region must reach a minimum of 2″ beyond each side of a welded seam. Gas-Tungsten Arc Welding: It is advised to use DC straight polarity (electrode negative). Maintain the arc length as short as possible and ensure the hot end of the filler metal remains consistently within the protective atmosphere. Shielded Metal-Arc Welding: Electrodes must be stored in a dry environment, and if they have absorbed moisture, they should be heated at 600 F for one hour to ensure they are dry. Present configurations range from 60 amps for thin materials (0.062″ thick) to 140 amps for materials that are 1/2″ thick and above. It is advisable to weave the electrode a bit, as this alloy weld metal tends not to expand. Slag cleaning is performed using a wire brush (manual or electric). Thorough elimination of all slag is crucial before subsequent weld passes and also following the final welding. Gas Metal-Arc Welding: Use reverse-polarity DC for optimal results, with the welding gun positioned at a 90-degree angle to the joint. In Short-Circuiting-Transfer GMAW, a standard voltage ranges from 20 to 23, with a current of 110 to 130 amps and a wire feed rate of 250 to 275 inches per minute. For Spray-Transfer GMAW, a voltage between 26 and 33 volts and a current ranging from 175 to 300 amps, along with a wire feed speed of 200 to 350 inches per minute, are standard. Submerged-Arc Welding: The filler metal used should match that of GMAW. DC can be utilized with either reverse or direct polarity. Convex weld beads are favored.

Nimonic 90 possesses good weldability up to 5 mm thickness using TIG and MIG processes. Cross-sections above 5 mm can be joined using electron beam welding, friction welding, and inertial welding [47].

Hastelloy C-276 weldability is classified in group 43 as per ISO 15608. For brazing, the p-number is 111, as per ASME/AWS. A nitrogen shield is essential for reinforcing the welds [48].

Hstelloy C-4 prevents the development of grain-boundary precipitates in weld heat-affected zones and is suitable for wide chemical process applications in the as-welded condition. Its weldability belongs to group 43 (ISO 15608), its brazing has P-number 111 (ASME section IX) [49].

Hastelloy C-22 has good weldability. It is used as a universal weld filler metal due to its resistance to corrosion in weldments. For example, it has been used successfully to refurbish corroded welds in an alloy C-276 pulp and paper mill bleach-mixing device. It is included in weldability group 43 (ISO 15608) [54]. HASTELLOY^®^ C-22^®^ can be welded using the same type of inert gas welding process as WIG and MIG, as well as the arc fusion welding process [461].

Haynes 59 is appropriate for all technical welding methods. Excellent thermal stability and minimal susceptibility to hot cracking. Weldments possess the same resistance to corrosion as the base metal. Alloy 59 is effectively utilized as a filler metal. Its welding has p-number 43, and its brazing has p-number 111 as per ASME Section IX and ISO/TR 20173. Weld is realized in a solution-annealed state [55].

Hastelloy C2000 exhibits very good weldability, belonging to Group 43 according to ISO 15608 [56].

Inconel 686 is readily weldable without the need for post-weld heat treatment. Alloy 686 Filler metal (AWS A5.14 ERNiCrMo-14, INCO-WELD^®^ 686 CPT) is best for joining Ni alloys (e.g., alloy 686, alloy C-276, alloy 22), as well as duplex, super-duplex, and super-austenitic stainless steels. It is also highly effective for welding dissimilar alloys. Welds made with alloy 686 exhibit superior resistance to pitting and crevice corrosion compared to the base metal. Alloy 686 can also be welded using coated electrodes ENiCrMo-3 and ERNiCrMo filler metals numbered 4 through 10 [57].

Inconel 617 Alloy 617 exhibits good weldability, belonging to weldability group 46 (ISO 15608) and 43 (ASME Section IX). It’s brazing P-number is 111 (ASME Section IX) [58].

Kou et al. [462] established the welding heat-affected zone (HAZ) of the C-HRA-2 Ni-based alloy at varying peak welding temperatures (Tp) ranging from 850 to 1450 °C using welding thermal simulation. The fine and distributed M_23_C_6_ carbides were observed along the grain boundaries in the simulated HAZs at 1050 and 1150 °C. In the simulated HAZs exceeding 1250 °C, the eutectic microstructure of γ matrix associated with M_23_C_6_ carbides was recognized near the grain boundaries caused by constitutional liquation. The liquefaction of the γ matrix and M_23_C_6_ carbides mainly explained the simulated HAZ liquation of the C-HRA-2 alloy during continuous welding heating. The amount of the liquefied phase rose as Tp increased. During the cooling process, the liquefied phase transformed into the eutectic microstructure consisting of γ matrix and M_23_C_6_ carbides. Apart from 1050, 1150, and 1450 °C, the microstructures at other Tp closely resembled the microstructure of the Base Metal (BM). The microhardness distribution of simulated HAZs showed a tendency to be greater in the center and less at both ends as Tp increased. The microhardness of the simulated HAZ matched the BM most closely at 1050 °C.

#### 3.2.2. Cracking

**Cracking during the weld metal solidification in SSS Ni alloys**

**Figure 11:**
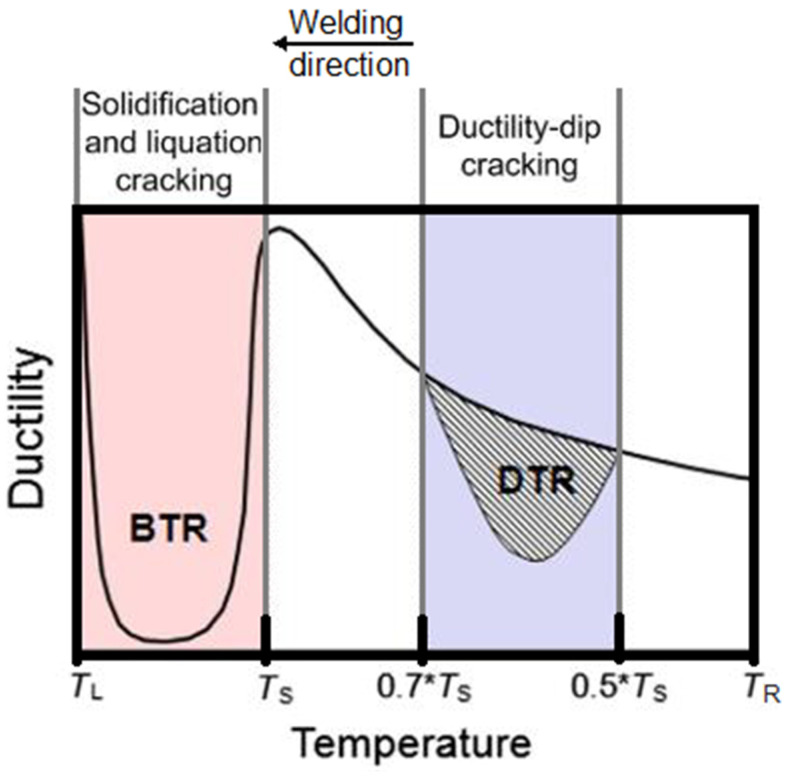
Weld joint ductility as a function of temperature. *T*_R_—room temperature, *T*_S_—solidus temperature; *T*_L_—liquidus temperature; BTR—brittle temperature range; DTR—ductility-dip temperature range [4].

In contrast to pure elements that crystallize at a definite temperature, alloys solidify within a temperature range that lies between liquidus and solidus temperatures, resulting in the mushy zone where both liquid and solid phases coexist simultaneously, as shown in Figure 11. These conditions favor the formation of cracks due to the thermal stresses generated by welding.

A tiny volume of terminal liquid with low surface tension and ongoing wetting of solidification grain boundaries are factors that encourage solidification cracking. Conversely, a substantial quantity of terminal liquid can aid in repairing the fissures by seeping into them. The least chance of cracking during the solidification of these alloys occurs when a minimal quantity of terminal liquid with elevated surface tension creates isolated liquid droplets, [463]. To efficiently reduce the significant cracking tendency of SSS Ni alloys, welding constraints must be kept to a minimum.

**Figure 12:**
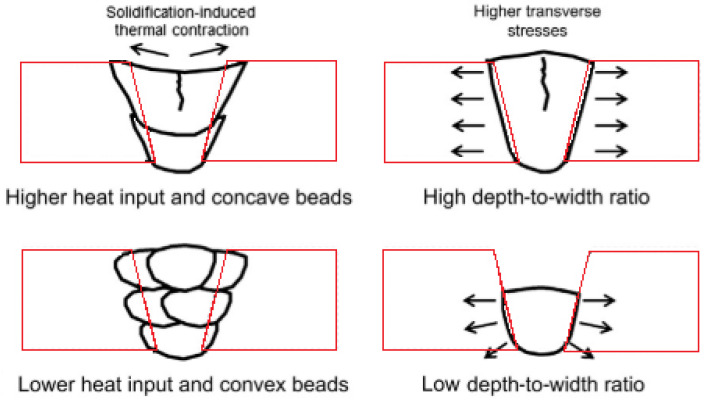
Effect of heat input and depth-to-width ratio on weld metal solidification cracking [4].

There are other options to minimize solidification cracking in weld metal, such as:

- appropriate choice of filler metal that can reduce the solidification temperature range of the weld metal;
- base metal containing minimal amounts of elements (P, S, B) that are capable of creating low-melting eutectics;
- minimal heat input while welding, leading to:
  - reduced solidification duration resulting from a sharper temperature gradient and
  - smaller weld beads that minimize solidification strains,
- appropriate weld shape featuring a low depth-to-width ratio and a convex surface profile due to improved stress distribution and grain structure, as illustrated in Figure 12.

Inconel 800 alloy is prone to hot cracking because of its elevated Fe content. Nonetheless, lowering the Al and Ti content to below 0.06% nearly eradicated hot-cracking. This could also imply that alloy 800H and alloy 800HT are more prone to hot-cracking because of their generally higher Al+Ti content [38].

**Liquation cracking in the semi-molten area and heat-affected region of SSS Ni alloys**

Liquation cracking in Ni-based alloys can take place in the heat-affected zone (HAZ) and partially melted zone (PMZ) of the welded joint. The partially melted area is a narrow boundary layer that separates the weld fusion zone from the heat-affected zone (HAZ). The highest temperature attained in the PMZ during welding fell between the solidus and liquidus of the alloy. The PMZ is typically regarded as a component of the HAZ. Liquation cracks typically form at a grain boundary where impurities with low melting points migrate and concentrate. The second process for grain boundary wetting, referred to as the penetration mechanism, happens when a mobile grain boundary crosses a region that has experienced local melting.

Comparable guidelines for reducing PMZ and HAZ cracking can be utilized for weld metal solidification cracking. Moreover, the quantity of precipitation creating alloying elements (Nb, Ti, and Si) should be reduced if feasible [464,465]. The fine-grained microstructure of the base metal minimizes stress concentrations and evenly spreads the liquid film through the PMZ [466].

**Ductility-dip fractures (DDF) of SSS Ni alloys**

Certain Ni-based alloys experience a reduction in ductility in the solid state within a limited temperature range situated between the alloy solidus temperature (Ts) and 0.5 Ts. This occurrence, known as ductility-dip and seen in all austenitic alloys, may lead to cracking when combined with thermal stresses that arise during welding. Ductility-dip cracking typically occurs in the weld metal, but it may also take place in the HAZ and lead to significant weldability problems in Ni-based alloys. The DDC consistently spreads along migrated grain boundaries. Various theories have been proposed to elucidate the DDC mechanism, yet there remains a lack of agreement on this matter [467]. Nonetheless, DDC arises from a complicated interaction of alloy metallurgy and thermal stresses created by a welding process.

Certain general guidelines might assist in preventing DDC in Ni-based alloys:

- utilize Nickel 82, Nickel 625, and 52MSS filler metals, which possess sufficient niobium to create carbides and a finer grain structure that is resistant to DDC,
- filler metals with a high Cr content (approximately 30 wt.%) should be avoided,
- utilize only Ar or He rather than mixtures that include H_2_ (which is used to enhance the wetting properties of the filler metal),
- reduce constraints and leftover stresses through appropriate joint design and
- favor low thermal input [3,4].

#### 3.2.3. Weldability of PS Ni-Based Alloys

The intricate and delicate microstructure of these alloys can lead to significant weldability challenges. PS Ni alloys have quite similar alloying elements to those in SSS alloys. This is why they experience metallurgy-related cracking issues due to welding—solidification cracking of the weld metal and liquation cracking in the partially melted zone. The ductility-dip cracking seldom occurs in PS Ni-based alloys. Possible reasons for this may be linked to the microstructure of the weld joint. Furthermore, most PS alloy uses involve relatively thin and not heavily constrained workpieces. Nonetheless, PS alloys are often regarded as more challenging to weld than SSS Ni-based alloys, in part due to specific strain-age cracking (SAC) that may occur during reheating or post-weld heat treatment (PWHT) [3,454].

Welding alloy K-500 is best obtained by gas tungsten arc welding (GTAW). Weldments using AWS A5.14 ERNiCu-7 filler metal cannot reach the strength of the base metal because it cannot be age-hardened. For welds needing strength, AWS 5.14 ERNiFeCR-2 filler metal can be used [61].

Alloy 80A exhibits fair weldability. Post-weld heat treatment is needed to achieve the best properties [62]. Nimonic alloy 80A sheet can be easily joined using any of the resistance welding methods. Fusion welding through traditional methods like T.I.G. or M.I.G. (dip or pulsed transfer) works well for section thicknesses of up to approximately 5 mm. Beyond this thickness, micro-fissures can develop in the weld and the heat-affected zone. Electron beam, friction, inertia, and flash-butt welding techniques have all been effectively applied for thicknesses exceeding 5 mm. The standard safety measures for welding Ni alloys must be followed, and welding should be performed on solution-treated material. Heat treatment after welding is essential for attaining optimal properties. Brazing at high temperatures in a vacuum, dry H_2_, or inert environment is effective, and there are several appropriate brazing alloys on the market [468].

Nimonic 90 exhibits good weldability up to 5 mm thickness using TIG and MIG technologies. For cross-sections above 5 mm, it can be joined using electron beam welding, friction welding, or inertial welding [47]. NIMONIC alloy 90 sheet can be easily welded using any resistance welding method. Fusion welding using traditional methods like TIG or MIG (dip or pulsed transfer) is adequate for thicknesses of approximately 5 mm. Above this thickness, microfissuring can take place in both the weld and the heat-affected area. Electron beam, friction, inertia, and flash butt welding have all been effectively utilized for materials thicker than 5 mm. The standard precautions for Ni base alloys must be followed, and welding should be performed on solution-treated materials. Post-weld heat treatment is essential to obtain the best properties. Vacuum, dry H_2_, or inert atmosphere high-temperature brazing is effective for NIMONIC alloy 90, and there are various appropriate brazing alloys obtainable [469].

C-263 Alloy 263 offers better weldability than alloy 80A. It can be joined using any standard technique. It does not exhibit vulnerability to cracking from post-weld heat treatments or from the heat-affected zone. The datasheet for Haynes^®^ 263 alloy indicates that a heat treatment after welding is necessary, consisting of a solution annealing process followed by an aging treatment. The datasheet for Nimonic alloy 263 indicates that “pre-weld heat treatment is unnecessary for age-hardened assemblies; however, a following age-hardening treatment is recommended after completing all salvage welding.” Materials will deteriorate in service if temperatures exceed 750 °C. The datasheet for VDM Alloy C-263 indicates that the material must be in the annealed state prior to welding, and suggests performing diffusion annealing following the welding process [63].

Haynes 282 alloy offers outstanding high-temperature characteristics along with solid weldability and machinability. At elevated temperatures (~900 °C), the alloy exhibits greater creep strength compared to Waspaloy alloy (UNS N07001) and nears the creep strength of R-41 alloy (UNS N07041). Due to its superior thermal stability, fabricability, and weldability compared to Waspaloy and R-41 alloys, this alloy is now being evaluated as a potential universal consumable for welding and repair welding of gamma prime strengthened Ni-based superalloys and is also being seen as an appropriate alternative for uses where R-41, Waspaloy, and 263 (UNS N07263) alloys are presently utilized. The alloy was engineered to exhibit better resistance to strain age cracking, a typical issue associated with gamma prime-strengthened Ni-based superalloys [470].

When welded in the annealed state, the 282 alloy shows outstanding weldability for a γ′-strengthened alloy. Welding tests were performed on 282 alloy following prolonged thermal exposure (LTTE). Welds were effectively finished on the LTTE material, and the transverse weld tensile characteristics were assessed. To assess vulnerability to liquation cracking in the heat-affected zone (HAZ) during welding, Gleeble hot-ductility tests were conducted on the LTTE material. Along with substantial primary Ti(C,N) and (Ti,Mo)-rich MC particles, the microstructure of 282 in the LTTE condition predominantly showed intergranular Cr-rich M_23_C_6_ carbides and both intra- and intergranular Mo-rich M6C carbides. γ′ precipitates with an average diameter of about 100 nm were present. The capability of 282 alloy to be welded in the LTTE condition was shown. Welds were finished successfully without any cracking or other defects. A pre-weld solution anneal is not required before repair-welding 282 material that has been in use. There was liquation in the HAZ closest to the WM, but no HAZ liquation cracking occurred. Concerning the development of the two main secondary phases, intergranular M_23_C_6_ and intragranular γ′, the LTTE microstructure showed signs of rejuvenation after post-weld solution annealing and age hardening. In the as-welded state, failure typically happened in the WM. At 871 °C, the strength remained stable no matter where the failure occurred. Following post-weld heat treatment, tensile failure reliably occurred in the BM/HAZ. In comparison to other samples in a similar age-hardened state, the LTTE welded samples showed reduced strength, which was linked to a comparatively large HAZ grain size. The 282 alloy demonstrates outstanding resistance to HAZ liquation cracking when welded in the LTTE state [471].

Inconel 713 is mainly intended for cast parts, which makes machining and welding challenging. Due to the challenges associated with welding Inconel 713, it is advisable to minimize welding whenever possible. Welded connections might be less strong [72].

Welds are generally made using the gas tungsten arc process with either Haslelloy W Filler Wire or Inconel Filler Metal 92 [472].

Mirak et al. [473] indicated that the welding of Inconel 713 superalloy and high-strength AISI 4140 steel was effectively achieved using a pulsed Nd:YAG laser. A columnar dendritic formation occurred at the edge of the weld metal because of the elevated temperature gradient, whereas a decrease in gradient at the weld metal center resulted in a cellular dendritic structure. The rise in heat input promoted the development of a coarse dendritic structure, leading to a decrease in the hardness of the weld metal. The HAZ of 4140 steel displayed the highest hardness across various heat input levels because of the development of a martensitic microstructure, whereas the hardness in the HAZ of Inconel 713 side did not exhibit a notable variation. The increased heat input resulted in the separation of Nb, Mo, and Ti elements within the interdendritic areas and the creation of a brittle laves phase, leading to a decrease in tensile strength. Nonetheless, the weld joint showed a peak tensile strength of 1068 MPa with reduced heat input (1875 J/mm). The fracture surface of the tensile specimens exhibited a brittle fracture in the weld metal under varying heat inputs.

Waspaloy exhibits moderate weldability. It joins with comparatively few challenges, yet it occasionally experiences cracking during the necessary post-weld heat treatment. To prevent strain-age cracks, the rate of heating to the solution annealing temperature must be maximized. Furthermore, it is advised that the material to be welded should be in a solution-treated state prior to welding [66]. HAYNES^®^ Waspaloy RTW™ filler metal is utilized for gas tungsten arc and gas metal arc welding of the Waspaloy alloy. The weld metal deposited exhibits outstanding strength within the 1000–1800 °F temperature range. The alloy is primarily employed for essential gas turbine engine parts. The RTW™ filler metal coating on the spooled wire facilitates smooth feeding through welding machines and minimizes tip wear in contact tips [474].

René 41 displays adequate weldability with inert gas-arc techniques; however, its Al and Ti content necessitates further heat treatment. An extended solution treatment before welding is necessary (1066–1080 °C, 4 h, water quench). It is advisable to perform an additional aging treatment after welding (900 °C, 1 h, air cool) to reduce the likelihood of cracking [67].

Welding Rene 41 can be especially difficult due to its susceptibility to strain-age cracking.

Suggested Welding Techniques for this type of alloy consist of:

- Resistance Welding: Employs the electrical resistance of the material to produce heat and form welds. Its control overheat input makes it effective for Rene 41.
- Electron Beam Welding (EBW): Provides accurate regulation of the weld area with reduced heat-affected zones. The vacuum setting minimizes contamination, making it perfect for welds with high integrity.
- Gas Tungsten Arc Welding (GTAW): Needs proper joint alignment and cooling methods, like Cu backing bars or water-cooled jigs, to control heat and avoid cracking.

Rene 41 must be completely solution-treated prior to welding, with post-weld heat treatment typically including exposure to 2150 °F (1177 °C) for 4 h, followed by air cooling, and then aging at 1650 °F (899 °C) for an additional 4 h. This procedure aids in reinstating the material’s mechanical characteristics and alleviating residual stresses [475].

Inconel 725 exhibits excellent weldability, ideally utilizing GTAW or GMAW. To achieve optimal results, first anneal alloy 725 before welding, and then perform a solution anneal followed by age hardening post-welding [68].

The weldability of INCONEL alloy 706 is exceptional. The niobium/Ti precipitation-hardening system of the alloy offers a postponed reaction to precipitation-hardening temperatures. The slow aging reaction provides significant resistance to cracking due to post-weld strain aging. The alloy also withstands underbead cracking (microfissuring). Most joints that are not severely constrained can be repaired by welding and directly re-aged without cracking. Pierce-Miller patch tests conducted on four thicknesses of annealed sheets exhibited no cracking following the welding, age-hardening, repair welding, and re-aging of specimens. Repairing welds in heavily constrained joints, however, might necessitate solution treatment prior to re-aging. Welding methods for alloy 706 are identical to those for INCONEL alloy 718. The suggested method is gas-tungsten-arc welding utilizing INCONEL Filler Metal 718. The alloy has also been fused using the flash-butt method; strong welds were created with lower upset pressure than that required for alloy 718. Welds in PS alloy 716 show elevated strength levels [69].

Incoloy alloy 909 can be easily welded using the gas-tungsten arc method [70].

Welding Incoloy 909 can be performed using conventional gas-tungsten arc welding (GTAW) methods. Adequate surface preparation is crucial to prevent contamination and guarantee robust welds. Important factors to take into account for welding consist of:

- Surface Preparation and Filler Material: Make sure surfaces are clean and devoid of contaminants, and choose a suitable filler material to preserve weld strength.
- Welding Settings: Regulate thermal input to avoid grain expansion and maintain the strength of the material [476].

A matching alloy filler metal must be utilized. Ultimately, the closest alloy with higher levels of Ni, Co, Cr, and Mo should be utilized. All weld beads ought to be somewhat rounded. Preheating is not required. Welding surfaces need to be clean and devoid of oil, paint, or crayon marks. The cleaned region must reach a minimum of 2″ past each side of a welded seam. Gas-Tungsten Arc Welding: It is advisable to use DC straight polarity (electrode negative). Maintain the arc length as briefly as you can and ensure the hot end of the filler metal remains consistently within the protective atmosphere. Shielded Metal-Arc Welding: Electrodes need to be stored in a dry location, and if they have absorbed moisture, they should be baked at 600 F for one hour to ensure they are dry. Current configurations range from 60 amps for thin material (0.062″ thick) to 140 amps for material that is 1/2″ and thicker. It is advisable to weave the electrode a bit since this alloy weld metal tends not to disperse. Slag cleaning is performed using a wire brush, either manual or electric. Thorough elimination of all slag is crucial before each subsequent weld pass and also after post-final welding. Gas Metal-Arc Welding: Utilize reverse-polarity DC for optimal outcomes, with the welding gun positioned at a 90-degree angle to the joint. For Short-Circuiting-Transfer GMAW, a common voltage range is 20–23 volts, with a current of 110–130 amps and a wire feed rate of 250–275 inches per minute. For Spray-Transfer GMAW, a voltage between 26 and 33 volts and a current of 175 to 300 amps, along with a wire feed rate of 200 to 350 inches per minute, are commonly observed. Submerged-Arc Welding: A matching filler metal, identical to that used in GMAW, is required. Either reverse or direct polarity of DC current can be utilized. Convex weld beads are favored [477].

Incoloy alloy 945 can be easily welded using gas-tungsten-arc welding (GTAW) and pulsed gas-metal arc welding (P-GMAW) techniques with matching composition filler metal or INCO-WELD filler metal 725NDUR. To achieve optimal outcomes, the current for the P-GMAW process should remain below 185 amperes for regular power sources operating in the “spray arc” metal transfer mode. Submerged-arc welding (SAW) and shielded-metal arc welding (SMAW) are not advised for alloy 945 [71].

**Solidification cracking in weld metal of PS Ni-based alloys**

**Figure 13:**
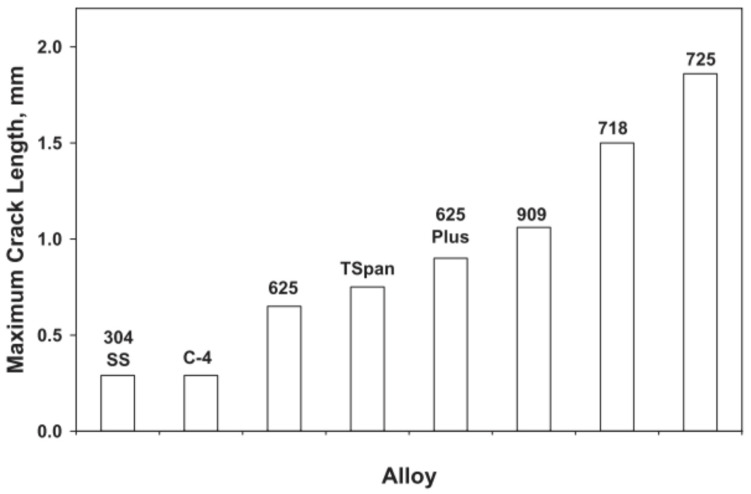
Varestraint test results for some commercial PS Ni-based alloys [3].

The occurrence of elements like P, S, B, C, Nb, and Zr in PS alloys or filler metals negatively impacts solidification cracking, even though their concentrations are quite low [464,478]. Double melting methods can greatly diminish the levels of impurities (S, P), but the levels of hardening elements such as B, C, Nb, and Zr cannot be lowered without compromising their alloying benefits since they contribute to high strength and creep resistance at high temperatures [16]. Figure 13 illustrates the tendency for solidification cracking in certain PS Ni-based alloys. The ranking relies on the longest crack length seen on the specimen post-Varestrain test, where a shorter maximum crack length suggests a reduced tendency for solidification cracking.

**Liquation cracking in the semi-molten area and in the heat-affected zone PS Ni alloys**

The liquation cracking in the PMZ and HAZ continues to be a significant issue in numerous PS Ni-based alloys. The presence of alloying elements and impurities greatly influences the alloy’s vulnerability to this form of cracking. Liquid film segregation of low melting phases from S, P, and B at grain boundaries leads to liquation cracking. Element Nb creates carbides (NbC) and leads to constitutional liquation at grain boundaries, which further adds to the liquation cracking in the PMZ [479]. Conversely, the introduction of Mn and Mg generally decreases PMZ liquation cracking as they interact with sulfur, thereby inhibiting its segregation and wetting at the grain boundaries [381]. A fine-grain size positively influences this type of cracking by decreasing the average thickness of liquid surrounding grain boundaries and allowing for significant liquid continuity, similar to what occurs with larger grains. Moreover, finer grains enlarge the overall boundary area, leading to a decrease in unit strain and local concentration of boundary stress [480].

**Strain-age cracking in PS Ni alloys**

**Figure 14:**
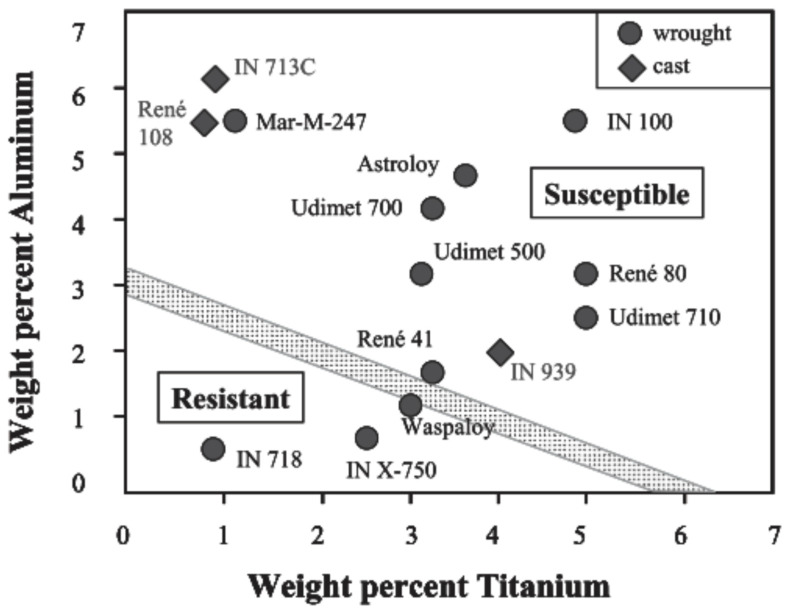
The effect of Ti and Al content on SAC of Ni-based alloys [3].

Strain-age cracking (SAC) in PS Ni-based alloys generally happens in two scenarios. The initial instance occurs during PWHT, which is conducted to reinstate the mechanical properties of the alloy [135]. The second one pertains to the scenario where strain age cracks occur during multi-pass welding, which also leads to reheating. The SAC cracks typically occur in the HAZ or occasionally in the WM zone. The process causing this type of cracking involves simultaneous alloy precipitation hardening (aging) along with the existence of localized strains. The strains arise from the relaxation of weld residual stresses, changes in dimensions caused by precipitation, or uneven heating during PWHT. Alloys enhanced by gamma prime (γ′ or Ni_3_(Ti, Al)) precipitates are particularly prone to SAC, and several of them are deemed unweldable, as shown in Figure 14.

**Figure 15:**
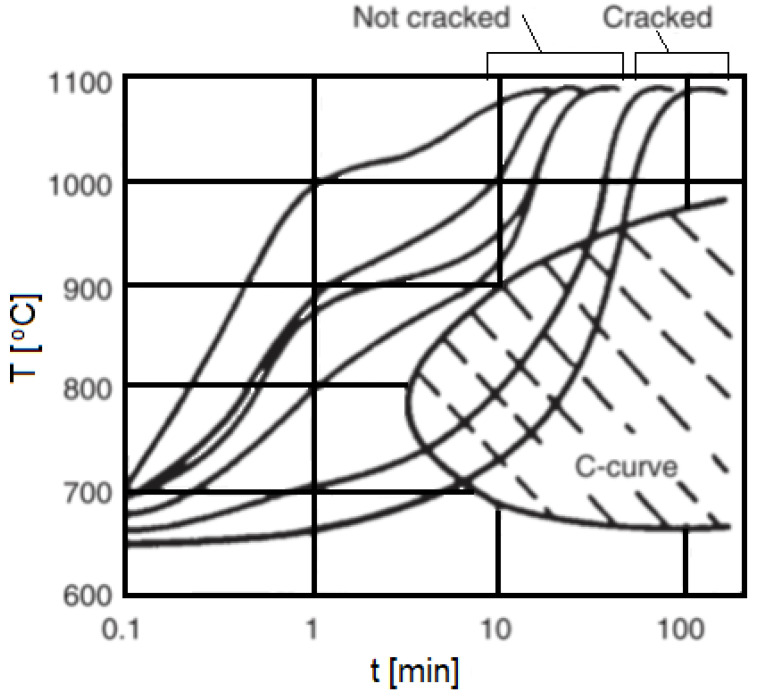
Effect of heating rate and C-curve on SAC susceptibility [3].

The extent of precipitation hardening during PWHT or reheating is regarded as the key factor influencing an alloy’s susceptibility to strain-age cracking. Typically, alloys that are reinforced by γ′ precipitates exhibit a greater hardening rate in comparison to those that are reinforced by γ″ precipitates (Ni_3_Nb, Ni_3_V). This is the primary reason that γ′ precipitations in strengthened alloys are considerably more vulnerable to SAC. The dynamics of alloy precipitation can be illustrated by the C-curve in a time-temperature diagram, as shown in Figure 15. Precipitation hardening takes place when the heating curve intersects the C-curve during PWHT, leading to SAC if local strains at the grain boundaries are also present. The alloys with a C-curve shifted to the right feature reduced Ti and Al levels (for example, Alloy 718) and demonstrate greater resistance to SAC.

A method to lessen the likelihood of SAC is to minimize the restraint level of a welded part, thereby decreasing residual stresses that are alleviated during PWHT. The fine-grained base metal microstructure positively influences SAC—a larger grain boundary area allows for easier stress relaxation, while embrittling phases are spread over a broader area. To further reduce susceptibility to cracking, the base metal must be in a solution-annealed or overaged state. A pliable and malleable base metal allows welding and PWHT stresses to be applied without fracturing.

While PWHT is often necessary for many PS alloys, implementing the correct PWHT process can reduce the likelihood of alloy cracking and also rehabilitate the mechanical properties of the weld joint. Fairly quick heating to the solution temperature can help prevent precipitation reactions within the aging temperature range. For larger welded components, stepped heating is necessary to reduce thermal stresses while heating. It is further shown that performing the PWHT in a protective environment (Ar or vacuum) can marginally enhance SAC resistance.

Typically, the welding technique and related settings ought to be selected to minimize heat input, while a weld joint design that lowers restraint levels is favored. Preheating may also positively influence the removal of SAC cracks in certain PS Ni-based alloys like Alloy 713C.

#### 3.2.4. Weldability of Ni SAs

Both types of alloys, the oxide dispersion-strengthened Ni alloys (ODS) and Ni aluminides, continue to face unresolved challenges related to their fusion weldability. The arc welding methods greatly weaken the weld joint characteristics of ODS alloys when initially well-dispersed oxides cluster or rise to the surface of the weld pool during melting. It appears challenging to sidestep this issue while maintaining the desirable ODS alloy characteristics in welded joints. Slightly improved outcomes can be achieved with laser or electron beam welding, methods that possess considerably greater energy density than arc welding. Options other than fusion welding, like solid-state welding methods and brazing, are feasible, but only minor enhancements can be anticipated [4,481].

Welding Ni aluminides frequently results in crack formation because of their low ductility and toughness [482]. In addition, the intricate cracking process typically happens in HAZ and appears to involve a mix of solidification, liquation, and solid-state cracking. Nonetheless, it has been shown that welds without defects in Ni aluminides can be achieved through electron beam welding within a restricted set of parameters. Moreover, acceptable joints were achieved through brazing [4,483,484,485].

Using infrared brazing at temperatures from 800 to 1200 °C over periods of 2–290 s Yang et al. [486] examined the initial microstructural development of NiAl/Al/NiAl and Ni_3_Al/Al/Ni_3_Al. The Al_3_Ni phase was initially created in both the Al-rich melt and the interface of the Ni aluminide base material with the Al-rich melt for samples brazed at 800 °C. Solid-state interdiffusion between Al_3_Ni and the base material led to the development of the interfacial Al_3_Ni_2_ phase, while the further growth of the Al_3_Ni_2_ phase at 800 °C was hindered by the Al_3_Ni interlayer due to its stoichiometric properties. For samples brazing at 1000 °C, the reaction shifted from hypereutectic to a peritectic one. The Al-rich melt absorbed additional Ni atoms as the brazing temperature rose to 1000 °C. The Al_3_Ni_2_ phase was then first produced in both the Al-rich melt and the joint interface. The development of the Al_3_Ni_2_ interlayer at 1000 °C occurred much more quickly than at 800 °C. The movement of Al atoms in the formation of the Al_3_Ni_2_ phase at 1000 °C was enhanced due to the interaction between Al_3_N_i2_ and the liquid Al-rich melt. The Al_3_Ni in the joint formed during the cooling phase of infrared brazing. The microstructural changes in the Ni_3_Al/Al/Ni_3_Al joint resembled those in the NiAl/Al/NiAl joint, with the exception of the NiAl phase developing between Al_3_Ni_2_ and Ni_3_Al. It can also be ascribed to the solid-state interdiffusion between Ni_3_Al and Al_3_Ni_2_ interlayer. The intermediate phase Ni5Al3, stable at temperatures below 700 °C, was not observed.

Alloys of NiAl-Cr-Mo, particularly those featuring a eutectic structure, are typically regarded as challenging to weld. The difficulties arise from the alloy’s elevated melting point, superior oxidation resistance, and propensity to create brittle intermetallic compounds. Nonetheless, studies are investigating approaches, such as selective electron beam melting, to address these issues and produce high-quality welds. NiAl-Cr-Mo alloys, especially eutectic types, exhibit elevated melting points and outstanding resistance to oxidation at elevated temperatures. These characteristics complicate welding because of the requirement for very high temperatures and the risk of oxidation occurring during the process. The inclusion of Cr and Mo within the NiAl matrix may result in the creation of brittle intermetallic phases, which could adversely impact the mechanical properties of the weld and possibly trigger cracking. Conventional welding techniques might find it challenging to melt and join these alloys without generating too much heat, resulting in distortion, embrittlement, and inferior joint characteristics. Studies are concentrating on different welding methods such as selective electron beam melting (SEBM) to address these issues. SEBM provides elevated cooling rates, resulting in fine microstructures and possibly allowing for the creation of high-quality welds. The welded joint’s microstructure, especially the arrangement of intermetallic phases and the size of the grains, has a considerable effect on the mechanical properties. Enhancing welding parameters and microstructure can result in better strength, hardness, and fracture toughness [487,488].

Oxide dispersion strengthened (ODS) Ni alloys are typically challenging to weld because of their distinct microstructure and the difficulties in preserving the oxide dispersion during the welding process. Although solid-state welding methods, including hot pressing and resistance spot welding, have been investigated, fusion welding techniques like TIG and laser welding are more frequently employed. Porosity, cracking due to heat and warmth, and solidification cracking in weld metal are frequent weldability issues associated with Ni and its alloys, such as Ni ODS [5,489,490,491,492]. The outstanding mechanical properties and high-temperature stability of ODS alloys, resulting from the distribution of nano-sized oxide particles in the metal matrix, pose challenges for welding. The welding procedure may cause these oxide particles to coarsen and clump together, potentially undermining the properties of the material [491]. Solid-state welding techniques, such as hot pressing and resistance spot welding, provide the capability to join ODS alloys with minimal alterations to the microstructure. Nevertheless, these processes might not be ideal for every application, and their relevance must be thoroughly evaluated [489,492]. Fusion welding methods, including TIG and laser welding, entail melting the base metal and joining the parts together. These processes are increasingly utilized for connecting ODS alloys, yet they also present difficulties.

Issues such as porosity, hot and warm cracking, along with weld metal solidification cracking, must be dealt with [5,493]. Cracking due to solidification of weld metal may happen in ODS alloys, especially in those with elevated Cr levels or additional elements that may lead to segregation and cracking at the weld interface [5]. Hot cracking may happen in the weld metal or in the heat-affected zone (HAZ) close to the fusion line. S, P, Pb, Bi, and B may lead to hot cracking in Ni alloys [306]. Ductility-Dip Cracking (DDC) is a form of cracking that may happen during welding, as well as in ODS Ni alloys [5].

TIG and MIG welding are commonly favored for Ni alloys because they can deliver a controlled heat input and minimize the chances of porosity and cracking [196,493].

Other welding techniques, like shielded metal arc welding (SMAW) and submerged arc welding (SAW), can join Ni alloys, but they might not work well for ODS alloys because of difficulties in preserving the oxide dispersion and avoiding cracking [196,493].

O’Donnell [494] discussed on various welding processes involved in joining ODS materials, including gas-tungsten arc welding, gas-metal arc welding, electron-beam and laser-beam welding, resistance welding, furnace brazing, friction welding, and explosion welding.

Onuki et al. [495] explored a novel joining method for Ni-based ODS alloy. This method is executed without brazing and aims to improve the dependability of connections under high-temperature conditions. The key aspect of the process involves eliminating surface oxide layers by maintaining ODS alloys at elevated temperatures (1523 K) in a vacuum (around 10(−4) Pa), followed by a series of solid-state diffusion bonding of the prepared surfaces at 1473 K within the same vacuum chamber. Specimens bonded through this novel method displayed both excellent tensile strength and significant ductility similar to that of the base metal. Nevertheless, the joints’ ductility experienced a minor reduction when subjected to temperatures of 1523 K for 2, 8, and 20 h in a vacuum; however, this extra process significantly enhanced the tensile strength.

Selective laser melting (SLM) offers distinct benefits in producing oxide dispersion-strengthened alloys because of its rapid cooling rate and small molten pool. This research conducted a comparative analysis of the microstructure and mechanical properties of Inconel 625 alloy and Y_2_O_3_ reinforced Inconel 625 alloy produced via SLM. The maximum density for the Y_2_O_3_/Inconel 625 specimen, recorded at 99.6%, was attained when the laser volume energy density was approximately 145.8 J/mm^3^. In contrast to the Inconel 625 specimen, the Y_2_O_3_/Inconel 625 specimen exhibits a larger molten pool, a finer grain structure, and a weaker texture in the deposition direction because of the active flux effect from Y_2_O_3_. In the process of heat treatment, the growth of grains and the formation of annealing twins in the Y_2_O_3_/Inconel 625 specimen are suppressed because of the pinning effect created by Y_2_O_3_ particles. The yield strength of Inconel 625 samples rises by 11.4%, 22.0%, and 12.5% at temperatures of 20, 800, and 1100 °C, respectively, owing to the strengthening from dispersion and grain boundaries caused by the incorporation of 0.48 wt.% Y_2_O_3_ particles. Moreover, the elongation of the Y_2_O_3_/Inconel 625 specimen at elevated temperatures is enhanced due to the role of Y_2_O_3_ in strengthening and providing oxidation resistance to grain boundaries [491].

Oxide-dispersion-strengthened (ODS) alloys display outstanding mechanical characteristics, rendering them suitable for high-temperature uses in fields like aerospace and nuclear reactors. The conventional production of ODS alloys includes mechanical alloying, followed by methods like hot extrusion and hot isostatic pressing. Nonetheless, these techniques face constraints in generating intricate geometries. Recent developments in additive manufacturing (AM) methods, particularly selective laser melting (SLM) and directed-energy deposition (DED), present intriguing new opportunities for producing ODS alloys. Initial studies showed the viability of employing SLM to fabricate intricate components with evenly distributed oxide particles, thus improving the materials’ characteristics. Later research demonstrated that fine-tuning the SLM parameters could enhance the mechanical properties of ODS alloys even further. DED methods have also demonstrated potential, featuring advancements such as in situ oxide creation during deposition and rapid laser cladding. These techniques have succeeded in generating ODS materials that exhibit improved microstructures and superior mechanical properties. Recent studies persist in examining the capabilities of AM for ODS alloys, concentrating on enhancing the distribution of nanoparticles and reducing the likelihood of particle agglomeration [490].

### 3.3. Electrodes and Fillers for Welding and Pre- and Post-Welding Processing

Filler metal is typically chosen to align with the strength and corrosion resistance of the base metal, which is especially crucial in challenging service conditions common for Ni-based alloys. When welding a base metal prone to cracking, it is essential to choose filler metal that modifies the composition of the weld metal and reduces the likelihood of solidification cracking. This occurs when the weld metal solidifies within a fairly limited temperature range and stops the creation of a low melting point eutectic compound. Filler metals frequently contain more major elements to reduce the dilution of weld metal during the welding process—when weld metal is less ‘noble’ than the base metal, galvanic corrosion issues may arise in an electrolyte environment. Additions for grain refinement are occasionally included in filler metal to reduce susceptibility to solidification, while deoxidizing elements like Ti and Al prevent the formation of porosity [3,158].

Suggestions for choosing filler metal for welding various Ni and Ni-based alloys are available in Table 36. In instances of welding rare alloys or dissimilar materials, it is advisable to consult the producer of the alloy [496]. Typically, the filler metal is categorized based on the group of Ni alloys for which it is most appropriate. For instance, filler metals classified as ERNi-1 are utilized for welding CP Ni alloys like Nickel 200 and 201. Consumables classified as ERNiCu-7 are ideally suited for welding Ni-Cu alloys like Alloy 400. When welding Ni-Mo, Ni-Cr, Ni-Cr-Fe, and PS alloys, highly alloyed filler metals are chosen [2].

Also, Table 37 includes some advice relating to the applicability of various fillers for joints between various materials.

According to [454] slag produced from Manual Metal Arc (MMA) welding, especially in submerged arc welding, can be challenging to eliminate from Ni alloys and typically requires grinding between passes for complete removal. It is frequently essential to grind the surface of each pass when welding with gas shielded processes to eliminate oxide scabbing, as wire brushing merely polishes these oxides. Not removing slag or oxide scabs will lead to weld metal inclusions and decrease corrosion resistance if they remain on unprotected surfaces. Thus, the overall welding durations can be significantly greater than those for comparable joints in stainless or carbon steel, and welders must be thoroughly familiar with these variations when switching from welding steels to Ni alloys. While the weld preparations resemble those employed for steel, it is important to contemplate the implementation of double V or U-type preparations at thicknesses thinner than those typical for steels. The extra expense of preparation is balanced out by reductions in consumable costs (with Ni being a costly metal) and welding duration. Most Ni alloys are ideally welded when they are in the annealed or solution-treated state, especially if they have undergone cold working. Preheating is not necessary unless it is to eliminate condensation or if the surrounding temperature is under roughly 5 °C, in which case a light preheat of 40–50 °C is advisable. The interpass temperature must not exceed 250 °C, although some alloy manufacturers suggest a lower interpass of 100 °C for specific alloys like Alloy C276. It is possible that hot crack issues may arise if thermal crayons are employed to gauge this temperature. For the majority of alloys, the heat input needs to be regulated to moderate levels (approximately 2 kJ/mm at most) to restrict grain growth and HAZ size, although for certain alloys like 718, C22, and C276, for instance, A recommended maximum heat input is 1 kJ/mm. On the other hand, using an excessively fast travel speed to achieve a low heat input may lead to a narrow weld bead prone to centerline cracking. Sufficient testing throughout the development of welding procedures should be utilized to enhance the spectrum of permissible welding parameters. Alloy 200 and 625, which are SSS alloys, do not need post-weld heat treatment to ensure corrosion resistance; however, they might undergo PWHT to lower the likelihood of stress corrosion cracking when used in caustic soda applications or with fluoro-silicates, or to achieve dimensional stability. A standard stress relief procedure would involve heating Alloy 200 to 700 °C for 30 min, and heating higher Cr alloys like Alloy 600 or 625 to 790 °C for four hours. The Ni-Mo alloys are designated with the prefix B, for instance, B1, B2, and are utilized in reducing conditions, including HCl gas and H_2_SO_4_, CH_3_COOH, and H_3_PO_4_. Alloy B2 is the most commonly found alloy, and compatible filler metals can be obtained. In contrast to Alloy B1, Alloy B2 fails to develop grain boundary carbide precipitates in the HAZ of the weld, allowing it to be utilized in most applications in its as-welded state. Alloy 400, consisting of 70% Ni and 30% Cu, exhibits excellent resistance to corrosion when in contact with hydrofluoric acid, strong alkaline solutions, and seawater. A compatible filler metal, Alloy 190, exists, but it may become anodic in saline solutions, resulting in galvanic corrosion; therefore, it is advisable to use a Ni-Cr alloy filler like Alloy 600 or 625 in this setting. The age-hardened alloy K-500 lacks a compatible filler metal and is typically welded with Alloy 190 filler, with the strength reduction considered during the design process. PS alloys are most effectively well-minded when in a solution-treated state; welding them in the age-hardened state may lead to cracking in the heat-affected zone. The aging process in the alloys is slow enough that the components can be welded while in the solution-treated state and subsequently aged at approximately 750 °C without losing mechanical properties. A solution treatment of the welded component, accompanied by aging will yield the greatest tensile strength. The susceptibility of the age-hardened alloy to cracking creates challenges during repair efforts, especially for items that have undergone high-temperature service and have experienced further precipitation at the grain boundaries.

There is little that can be done to address this issue besides a complete solution heat treatment, which is frequently unfeasible for a fully manufactured component. If a repair is to be made, it is advised to use small weld beads and welds with controlled low heat input. If the design allows, a low-strength filler metal, such as Alloy 200 or 600, can be utilized to minimize the risk. Applying butter to the surfaces of the repair weld preparation, occasionally together with a peening process, has proven effective. Numerous Ni alloy filler metals have been employed to create dissimilar metal joints with remarkable outcomes; dilution while welding joints of ferritic, stainless, and duplex steels is less critical compared to using a type 309 stainless steel filler. Ni has a thermal expansion coefficient that falls between that of ferritic and austenitic steels, which results in reduced thermal fatigue when high-temperature equipment is subjected to thermal cycling. Alloy 625 is a favored option, with its weld tensile strength either matching or surpassing that of the base metal. This method has its constraints, and care should be taken while choosing an appropriate filler. For instance, Alloy 625 has been widely utilized for welding different joints in austenitic and duplex steels. The application of this filler metal has led to the creation of niobium-rich precipitates near the fusion line and has been stopped. Alloy 59 or C22 filler metals have taken the place of Alloy 625 as the preferred filler.

## 4. Conclusions

Ni and its alloys are often utilized in high-temperature and harsh conditions where exceptional corrosion resistance, creep resistance, and strength are necessary. Given that the weldability of Ni-based alloys differs significantly, maintaining the properties of the alloy in welded joints can occasionally be difficult, if not a tough goal to achieve. Certain alloys, like CP Ni, can be welded with relative ease if low-solubility elements (P, S, B, and Si) are maintained at minimal levels, H_2_ absorption is avoided, and sufficient shielding is provided.

Some Ni-based alloys are classified into different groups by different authors [3,5,33,499]. No uniform standards and criteria for membership in this area have been developed so far.

Nonetheless, when welding Ni-based alloys that are SSS and PS, issues with weldability can arise due to solidification cracking and HAZ liquation cracking. Moreover, SSS alloys are prone to DDC, while PS alloys are also vulnerable to SAC. There is not a one-size-fits-all approach to preventing cracking when welding these alloys, but a few general recommendations can be beneficial: keep weld joint restraint to a minimum; using fine-grain base metal in a soft state (solution-annealed) lowers the risk of weld joint cracking; selecting the right filler metal reduces the alloy’s solidification temperature range and impurity levels; and lowering welding heat input, employing stringer bead techniques that create a convex weld profile, as well as opting for a low weld depth-to-width ratio, also positively influence cracking resistance.

The fusion welding of Ni-based SAs, particularly Ni aluminides, is regarded as very difficult due to the unavoidable degradation of alloy properties in welded joints. Brazing and solid-state welding techniques ought to be regarded as options, while high-energy-density fusion welding methods could be utilized for joining ODS alloys to a certain degree.

Since no standard has been found for specifying samples for testing various Ni-based alloys, samples related to stainless steel can be used.

New Ni-based alloys are under development, and welding methods have not yet been developed for them. This requires further research.

Further research is needed on the applicability and optimization of welding processes for various Ni-based alloys. New laser and FSW methods are being developed that are particularly promising.

## Figures and Tables

**Table 1:** Some of the physical features of CP Ni.

Feature	Unit	Value	Refs.
Density at 20 °C	kg/m^3^	8902	[6]
Melting temperature	°C	1453	[6]
Boiling temperature	°C	2730	[7]
Curie point	°C	354	[6]
Ionization energy	eV	7.64	[8]
Coefficient of thermal expansion at 20 °C	μm/(m·°C)	13	[6]
Electrical resistivity at 20 °C	μΩ·cm	8	[6]
Thermal conductivity at 20 °C	W/(m·K)	69	[6]
Specific heat at 20 °C	J/(kg K)	470	[6]

**Table 2:** Selected mechanical properties of CP Ni, [9].

Feature	Unit	Value	Refs.
Modulus of elasticity in tension	[GPa]	207	[9]
Tensile Strength,	[MPa]	317 *	[9]
Yield strength, 0.2% offset	[MPa]	59 *	[9]
Elongation in 51 mm	[%]	30 *	[9]
Hardness HB	[MPa]	800–3000	[10]
Hardness HV	[HV]	124	[11]
Poisson Number	[-]	0.305–0.315	[10]

* Annealed but with the suitable procedure—hot rolling, annealing, and cold work tensile strengths as high as 1103 MPa are attainable.

**Table 3:** Classification of Ni and Ni-based alloys [3].

Type of Metal and Its Alloys	Ni and Ni-Based Alloys			
Group	CP Metal	SSS	PS	Special alloys
Alloys Compositions	Ni	Ni-Cu	Ni-Al-Ti	Ni-Al Intermetallic
		Ni-Mo	Ni-Cu-Al-Ti	
		Ni-Fe	Ni-Cr-Al-Ti	
		Ni-Cr-Fe	Ni-Cr-Nb	Oxide Dispersion Strengthened
		Ni-Cr-Mo-W		
		Ni-Fe-Cr-Mo	Ni-Fe-Cr-Nb-Al-Ti	
		Ni-Cr-Co-Mo		

**Table 4:** Chemical compositions of some CP Ni alloys [12].

Components	Cu	Al	Fe	Mn	Ti	Ni
Alloy Designation	[%]					
200	<0.25	-	<0.40	<0.35	-	>99.0
270	0.001	-	0.003	0.001	-	>99.9
301	<0.25	4.0–4.75	<0.6	<0.5	0.25–1.0	balance

**Table 5:** Basic mechanical properties of CP Ni alloys.

Property	Alloy Designation	200	270	301	Refs.
	Unit	Value			
Density	[kg/m^3^]	8.89·10^3^	8.89·10^3^	8.26·10^3^	[12]
Modulus of elasticity	[GPa]	207	207	207	[12]
Thermal expansion (20 °C)	[°C^−1^]	13.3·10^−6^	13.3·10^−6^	12.9·10^−6^	[12]
Specific heat capacity	[J/(kg·K)]	456	460	435	[12]
Thermal conductivity	[W/(m·K)]	70.2	86	40.4	[12]
Electric resistivity	[Ohm·m]	9.5·10^−8^	7.5·10^−8^	42.4·10^−8^	[12]
Tensile strength (annealed)	[MPa]	462	345	462	[12]
Tensile strength	[MPa]	485	-	-	[3]
Yield strength (annealed)	[MPa]	148	110	148	[12]
Yield strength	[MPa]	275			[3]
Elongation (annealed)	[%]	45	50	45	[12]
Elongation	[%]	40	-	-	[3]
Hardness	[RB]	70	-	-	[3]
Liquidus temperature	[°C]	1446	1454	-	[12]
Solidus temperature	[°C]	1436	1454	-	[12]
Solution temperature	[°C]	-	-	925	[12]
Aging temperature	[°C]	-	-	588	[12]

**Table 6:** High-temperature strength and physical properties of soft-annealed Nickel 201 (based on [13]).

Property	Temperature	93	204	260	316	371	427	482	538	593	649	Refs.
	Unit	Value										
Yield strength	[MPa]		102	101	105	97	93	89	83	77	70	[12]
Tensile strength	[MPa]		372	372	362	325	284	269	228	186	153	[12]
Elongation	[%]		44	41	42	53	58	58	60	72	74	[12]
Coefficient of thermal expansion, from 20 °C to	[μm/m⋅K]	13.3	13.9		14.4							
Electrical resistivity	[nΩ·m]	126	188		273							
Thermal conductivity	[W/m·K]	67.1	61.3		56.3							

**Table 7:** Basic mechanical properties of selected SSS Ni-based alloys [3].

Property	Tensile Strength	Yield Strength	Elongation	Hardness
Alloy Designation	[MPa]	[MPa]	[%]	[RB]
400	585	345	35	75
600	550	345	40	90
601	620	380	40	95
617	795	450	40	98
C-22	690	380	35	97
625	830	415	30	95
690	620	380	40	95
800	585	310	40	85
800H	585	275	45	80
800HT	585	275	40	80
825	655	345	35	90

**Table 8:** Chemical composition of certain Ni-Cu alloys [12].

Component	Cu	Al	Ti	Fe	Mn	Si	Ni
Alloy Designation	[%]						
Monel 400	28–34	-	-	<2.5	<2.0	-	>63
Monel 405	28–34	-	-	<2.5	<2.0	<0.5	>63
Monel K-500	27–33	2.3–3.15	0.35–0.85	<2.0	<1.5	-	>63

**Table 9:** Basic properties of certain Ni-Cu alloys [3].

Property	Alloy Designation	Monel 400	Monel 405	Monel K-500
	Unit	Value		
Density	[kg/m^3^]	8.80·10^3^	8.80·10^3^	8.44·10^3^
Modulus of elasticity	[GPa]	179	179	179
Thermal expansion (20 °C)	[°C^−1^]	13.9·10^−6^	13.7·10^−6^	13.7·10^−6^
Specific heat capacity	[J/(kg·K)]	427	427	419
Thermal conductivity	[W/(m·K)]	21.8	21.8	17.5
Electric resistivity	[Ohm·m]	54.7·10^−8^	51·10^−8^	61.5·10^−8^
Tensile strength (annealed)	[MPa]	550	550	1100 pc
Yield strength (annealed)	[MPa]	240	240	630 pc
Elongation (annealed)	[%]	48	40	25 pc
Liquidus temperature	[°C]	1350	1350	1350
Solidus temperature	[°C]	1300	1300	1300

pc—precipitation hardened.

**Table 10:** High-temperature strength and physical properties of alloy 400.

Alloy Type	Property	Temperature [°C]	100	200	300	400	500	600	700	800	900	1000	1150	Refs.
		Unit	Value											
400	Yield strength	[MPa]	>150	>135	>130	>130 (425 °C)								[17]
K500			670	640	620	600	570	490						[18]
400	Tensile strength	[MPa]	>420	>390	>380	>370 (425 °C)								[17]
K500			1040	1020	980	890	750	620						[18]
400	Coefficient of thermal expansion, from 20 °C to	[μm/m⋅K]	13.8	14.5	14.9	15.2	15.6	16.0	16.4	16.8	17.3			[17]
K500			13.7	14.6	14.9	15.2	15.5	16.0	16.6	17.0	17.5			[18]
400	Electrical resistivity	[nΩ·m]	540	555	575	585	600	618	635	655	675			[17]
K500			6200	6300	6500	6500	6500	6600	6600	6700	6800			[18]
400	Thermal conductivity	[W/m·K]	25.4	28.7	31.9	34.7	38.4	41.2	43.1	45.1	47.5	50	52.9	[17]
K500			19.4	20.9	25.1	27.8	30.5	33.1	35.7	37.4	41.2			[18]
400	Specific heat capacity	[J/kg·K]	461	473	484	495	523	544	555	566	577	587	603	[17]
K500			545	480	491	500	517	538	567	613	685			[18]
400	Young modulus	[GPa]	180	177	170	165	150							[17]
K500			178	176	173	168	164	162	158					[18]

**Table 31:** Basic mechanical properties of selected PS Ni-based alloys [3].

Property	Tensile Strength	Yield Strength	Elongation	Hardness	Rupture Stress (1000 h Exposure) MPa		
Alloy	MPa	MPa	%	HRC	650 °C	760 °C	870 °C
K500	1100	790	20	-	-	-	-
X-750	1100–1380	690–1035	15–30	35–40	540	275	55
80A	970–1240	585–830	25–36	-	520	220	-
90	1100–1310	760–860	17–30	-	-	240	75
263	970–1100	585–690	35–45	-	400	170	40
282	1165	720	32	32	550	240	70
713	830	-	5	35–45	-	-	-
718	1035–1590	900–1240	14–30	30–40	580	195	-
Waspaloy	1345	900	26	34–45	460	195	50
Rene 41	1345	1070	15	33–40	580	235	60
725	1165–1310	830–970	25–35	30–40	-	-	-
706	1240–1380	970–1100	15–25	30–40	550	170	-
909	1275	1035	15	-	325	-	-
925	970	760	18	26–38	-	-	-
945	1100–1240	900–1035	20–30	40–45	-	-	-

**Table 32:** The chemical composition of certain PS Ni-based alloys.

Components	Ni	Fe	Cr	Mo	W	Al	Co	Cu	Zr	Mn	Ti	Si	Nb	Refs.
Alloy	[%]													
K-500	63–70	2	-	-	-	2.3–3.15	-	Bal.	-	1.5	0.35–0.85	0.5	-	[61]
Nimonic 80A	Bal.	<3.0	18–21	-	-	1–1.8	<2.0	<0.2	-	<1	1.8–2.7	<1	-	[62]
Nimonic 90	Bal.	<1.5	18–21	-	-	1–2	15.0–21.0	<0.2	-	<1	2–3	<1	-	[47]
Nimonic C-263	Bal.	<0.7	19–21	5.6–6.1	-	<0.6	19.0–21.0	<0.2	-	<0.6	1.9–2.4	<0.4	-	[63]
Haynes 282	Bal.	1.5	18.5–20.5	8–9	0.5	1.38–1.65	9–11	0.1	-	0.3	1.9–2.3	0.15	0.2	[64]
Inconel 713	Bal.	<2.5	12–14	3.8–5.2	-	5.5–6.5	1.8–2.8	<0.5	<0.15	0.25	-	-	<2.5	[65]
Inconel 718	50–55	Bal.	17–21	2.8–3.3	-	0.2–0.8	<1.0	<0.3	-	<0.35	0.65–1.15	-	4.75–5.5	[32]
Waspaloy	Bal.	<2	19	4.3	-	1.5	13.5	<0.1	0.05	<0.1	3	<0.15	-	[66]
Rene 41	Bal.	<5	18–20	9–10.5	-	1.4–1.8	10–12	-	-	<0.1	3.0–3.3	<0.5	-	[67]
Inconel 725	55.0–59.0	Bal.	19–22.5	7–9.5	-	<0.35	-	-	-	<0.35	1–1.7	<0.2	2.75–4	[68]
Inconel 706	39–44	Bal.	14.5–17.5	-	-	<0.4	<1	<0.3	-	<0.35	1.5–2	<0.35	2.5–3.3	[69]
Incoloy 909	35–40	Bal.	-	-	-	<0.15	12–16	-	-	-	1.3–1.8	0.25–0.5	4.3–5.2	[70]
Incoloy 945	45–55	Bal.	19.5–23	3–4	-	<0.7	-	1.5–3	-	<1	0.5–2.5	<0.5	2.5–4.5	[71]

**Table 33:** High-temperature strength and physical properties of K500.

Property	Temperature [°C]	100	200	300	400	500	600	700	800	900
	Unit	Values								
Yield Strength	MPa	670	640	620	600	570	490	-	-	-
Tensile Strength	MPa	1040	1020	980	890	750	620	-	-	-
Coefficient of thermal expansion, from 20 °C to	[μm/m⋅K]	13.7	14.6	14.9	15.2	15.5	16	16.6	17	17.5
Electrical resistivity	[nΩ·m]	6200	6300	6500	6500	6500	6600	6600	6700	6800
Thermal conductivity	[W/m·K]	19.4	20.9	25.1	27.8	30.5	33.1	35.7	37.4	41.2
Specific heat capacity	[J/kg·K]	545	480	491	500	517	538	567	613	685
Modulus of elasticity	[GPa]	178	176	173	168	164	162	158	-	-

**Table 11:** Chemical composition of certain Ni-Mo alloys.

Component	Mo	Cr	Ni	Fe	Mn	Co	W	Refs.
Alloy Designation	[%]							
B2	26–30	<1	Rest	<2	<1	<1	-	[20]
B3	27–32	1–3	>65	1–3	<3	<3	<3	[21]

**Table 12:** Physical and mechanical properties of certain Ni-Mo alloys [3].

Property	Alloy Designation	B2	B3
	Unit	Value	
Density ρ	[g/cm^3^]	9.2	9.22
Resistivity at 20 °C	[Ω mm^2^/m]	1.37	1.37
Linear thermal expansion coefficient α	[×10^−6^/K]	10.3	10.6
Thermal conductivity λ at 50 °C	[W/m K]	11.4	
Specific heat capacity at 20 °C	[kJ/kg K]	373–377	
Melting point (approx.)	[°C]	1300–1350	1370–1418
Tensile strength	[MPa]	760	750
Yield point	[MPa]	350	340
Elongation at rupture	[%]	40	40
Young Modulus	[GPa]	217	216
Hardness	[HB]	240	240
**Refs.**		[20,22]	[23]

**Table 13:** High-temperature strength and physical properties of alloys B2 and B3.

Alloy Type	Property	Temperature [°C]	100	200	300	400	500	600	800	Refs.
		Unit	Value							
B2 sheets and plates, 1.3 to 3.0 mm thick, solutioned and quenched	Yield strength	[MPa]				450		425	415	[22]
B2 sheets and plates, 2.5 to 9.0 mm thick, solutioned and quenched						350		325	310	[22]
B2 plates, 9 to 50 mm thick, solutioned and quenched						360		340	315	[22]
B3 3.2 mm thick sheets, bright annealed			380	325	300 (315 °C)	290 (425 °C)	270 (540 °C)	315 (650 °C)		[23]
B3 6.4 mm thick plates, solution treated			375	330	305 (315 °C)	285 (425 °C)	275 (540 °C)	290 (650 °C)		[23]
B2 sheets and plates, 1.3 to 3.0 mm thick, solutioned and quenched	Tensile strength	[MPa]				885		860	860	[22]
B2 sheets and plates, 2.5 to 9.0 mm thick, solutioned and quenched						850		820	805	[22]
B2 plates, 9 to 50 mm thick, solutioned and quenched						870		840	820	[22]
B3 3.2 mm thick sheets, bright annealed			830	760	720 (315 °C)	705 (425 °C)	675 (540 °C)	715 (650 °C)		[23]
B3 6.4 mm thick plates, solution treated			845	795	765 (315 °C)	745 (425 °C)	730 (540 °C)	735 (650 °C)		[23]
B2 sheets and plates, 1.3 to 3.0 mm thick, solutioned and quenched	Elongation	[%]				50		49	51	[22]
B2 sheets and plates, 2.5 to 9.0 mm thick, solutioned and quenched						59		60	60	[22]
B2 plates, 9 to 50 mm thick, solutioned and quenched						60		60	61	[22]
B3 3.2 mm thick sheets, bright annealed			56.9	59.7	63.4 (315 °C)	62.0 (425 °C)	59.0 (540 °C)	55.8 (650 °C)		[23]
B3 6.4 mm thick plates, solution treated			58.2	60.9	61.6 (315 °C)	61.7 (425 °C)	61.7 (540 °C)	64.6 (650 °C)		[23]
B2	Coefficient of thermal expansion, from 20 °C to	[μm/m·K]	10.3 (93 °C)	10.8 (204 °C)	11.2 (316 °C)	11.5 (427 °C)	11.7 (538 °C)			[22]
	Electrical resistivity	[nΩ·m]	1380	1380	1390	1390	1410	1460		[22]
	Thermal conductivity	[W/m·K]	12.2	13.4	14.6	16.0	17.3	18.7		[22]
	Thermal diffusivity	[mm^2^/s]	3.4	3.6	3.8	4.0	4.2	4.5		[22]
	Specific heat capacity	[J/kg·K]		406		431		456		[22]
	Dynamic modulus of elasticity	[GPa]			202 (315 °C)	196 (425 °C)	189 (540 °C)			[22]

**Table 14:** Chemical composition of certain Ni-Fe alloys.

Component	Ni	Fe	Refs.
Alloy Designation	[%]		
Nifethal^®^ 70	72	Bal.	[28]
Nifethal^®^ 52	52	Bal.	[28]
Invar K93600	36	64	[29]

**Table 15:** Physical and mechanical properties of Ni-Fe-based alloys.

Property	Alloy Designation	Nifethal 70	Nifethal 52	Invar K93600
	Unit	Value		
Max operating temperature	[°C]	600	600	260
Density ρ	[g/cm^3^]	8.45	8.20	8.13
Resistivity at 20 °C	[Ω mm^2^/m]	0.20	0.376	0.75
Temperature factor of the resistivity, C_t_				
250 °C	[-]	2.19	1.93	-
500 °C	[-]	3.66	2.77	-
Linear thermal expansion coefficient α				
20–100 °C	[×10^−6^/K]	-	10	1
20–500 °C	[×10^−6^/K]	13	-	-
20–1000 °C	[×10^−6^/K]	15	-	-
Thermal conductivity λ at 50 °C	[W/m K]	17	17	10.5
Specific heat capacity at 20 °C	[kJ/kg K]	0.52	0.52	0.515
Melting point (approx.)	[°C]	1430	1435	-
Tensile strength	[MPa]	640	610	490
Yield strength	[MPa]	-	340	310
Elongation at rupture	[%]	-	30	30
Young Modulus	[GPa]	-	-	140
Magnetic properties (Curie point)	[°C]	610	530	-
Emissivity, fully oxidized condition	[-]	0.88	0.88	-
Hardness	[HB]			140
**Refs.**		[28]	[28]	[20]

**Table 16:** Chemical composition of certain Ni-Mo alloys [29].

Component	Ni	Fe	Cr	Mo	W	Cu
Alloy Designation	[%]					
800	32	45	21	9	3.5	-
800H	32	bal.	20	-	-	-
825	42	25	21	3	-	2
G3	bal.	20	22	7	-	2

**Table 17:** The composition and some basic properties of various types of stainless steel [32].

Component	Ni	Cr	Mn	Si	Corrosion	Melting Range	Young’s Modulus
Type of Steel	%				Mpy (a)	°C	MPa
201	3.5–5.5	16–18	5.5–7.5	<1	20	1398–1454	193,053
301	6.0–8.0	16–18	<2.0	<1	12	1398–1421	193,053
302	8.0–10.0	17–19	<2.0	<1	10–18	1398–1421	193,053
304	8.0–10.5	18–20	<2.0	<1	6–12	1398–1454	193,053
309	19.0–22.0	24–26	<2.0	<1	5–9	1398–1454	199,947

(a) Corrosion rate is in mils per year. The test was performed in 65% Nitric acid at 118 °C.

**Table 18:** Components of Inconel (tm) Alloys.

Component	Ni	Cr	Mn	Fe	Cu	Al	Ti	Co	Nb	Mo	V	Refs.
Type	[%]											
600	>72	14–17	<1	6–10	<0.5	0	0	0	0	-	-	[32]
601	58–63	21–25	<1	Bal.	<1	1.0–1.7	0	-	-	-	-	[32]
625	>58	20–23	<0.5	<5	-	<0.4	<0.4	<1	3.15–4.15	8–10	-	[32]
718	50–55	17–21	<0.35	Bal.	<0.3	0.2–0.8	0.65–1.15	<1	4.75–5.5	2.8–3.3	-	[32]
800	32.5	21	<0.8	46	0.4	0.4	0.4	-	-	-	-	[32]
G3	bal.	21–23.5	<1	18–21	1.5–2.5			<5		6-8	<1.5	[33]

**Table 19:** Properties of Inconel (tm) alloys [32].

Type	Yield Strength at 21 °C	Melting Range	Rupture Strength; 100 h at (°C, MPa)	
	MPa	°C	°C	MPa
600	285	1355–1415	871	37
601	338	1355–1415	871	48
625	490	1260–1335	871	72
718	1186	1260–1335	649	689
800	250	1355–1385	982	145

**Table 20:** Properties of Inconel alloys.

Property	Unit	State	600	601	625	718	800	G3
Density	g/cm^3^	-	8.42	8.1	8.44	8.19	7.94	8.14
Melting Range	°C	-	1354–1413	-	-	1260–1360	1357–1385	1260–1343
Specific Heat	J/kg-K	-	444	450	410	435	502	453
Thermal conductivity	W/m·K	-	-	11.3	9.8	11.4	13	10
Linear expansion coefficient	μm/m·K	-		14.4–17.7	-	13–16	16.8–18	14.6–15.1
Electrical resistivity	μΩ·m	-	1.03	1.19	1.29	1.22	0.989	1.12
Curie temperature	°C	-	−124	-	<126	−112	−115	-
Young Modulus	GPa	-	190	206.5	207	200	193	199
Hardness	HRB	Cold-drawn and annealed	65–85	<220	190	32–40HRC	138	-
		Hot-finished	75–95	-	-	16HRC	-	-
Tensile Strength	MPa	Cold-drawn and annealed	550–690	550–750	855	1034–1240	517–827 (1034)	621–896
		Hot-finished	585–830	741	-	896	552–827	-
Yield Strength	MPa	Cold-drawn and annealed	170–345	>205	490	827–1189	207–448 (862)	241–862
		Hot-finished	240–620	290	-	448	172–448 (621)	-
Elongation	%	Cold-drawn and annealed	55–35	>30	50	12–22	60–25	45–13
		Hot-finished	50–30	47		54	50–25	-
**Refs.**			[34]	[35]	[36]	[37]	[38]	[33]

**Table 21:** High-temperature strength and physical properties of certain Inconel alloys.

Alloy Type	Property	Temperature [°C]	100	200	300	400	500	600	700	800	900	1000	Refs.
		Unit	Value										
601	Yield strength	[MPa]			221		203						[35]
625							405 (538 °C)	420 (649 °C)	420 (760 °C)	375 (871 °C)			[36]
718			1207	1138	1104	1090	1069	1055	897				[37]
601	Tensile strength	[MPa]			674		640						[35]
625							745 (538 °C)	710 (649 °C)	505 (760 °C)	285 (871 °C)			[36]
718			1379	1345	1338	1324	1276	1242	904				[37]
601	Elongation	[%]			46		45						[35]
625							50 (538 °C)	35 (649 °C)	42 (760 °C)	125 (871 °C)			[36]
601	Coefficient of thermal expansion, from 20 °C to	[μm/m·K]	13.75	14.36	14.58	14.83	15.19	15.62	16.11	16.67	17.24	17.82	[35]
625							14.0 (24–540 °C)			15.8 (24–870 °C)			[36]
718			13.2	13.6 (205 °C)	13.9 (315 °C)	14.3 (425 °C)	14.6 (540 °C)	15.1 (650 °C)	16.0 (760 °C)				[37]
800							16.8 (26–500 °C)	17.1 (26–600 °C)	17.5 (26–700 °C)	18.0 (26–800 °C)			[38]
G3			14.3	14.7	15.0	15.3	15.7	16.1	16.5				[39]
601	Electrical resistivity	[nΩ·m]		1207		1229		1247		1249	1259	1262	[35]
800			989	1035	1089	1127	1157	1191	1223	1266	1283	1291	[38]
G3			1170	1190	1210	1230	1250	1260	1270				[39]
601	Thermal conductivity	[W/m·K]		14.3		17.7		21.0		24.4	26.1	27.8	[35]
625							17.5 (540 °C)			22.8 (870 °C)			[36]
800			13.0	14.7	16.3	17.9	19.5	21.1	22.8		21.1	31.9	[38]
G3			12.6	14.3	16.0	17.7	19.3	21.0	22.6				[39]
601	Specific heat capacity	[J/kg·K]		498		548		603		657	686	712	[35]
G3			452	462	471	479	487	495	503				[39]
601	Dynamic modulus of elasticity	[GPa]		196.8		184.8		170.8		150.2	137.9	124.7	[35]
718			193 (150 °C)	190 (205 °C)	184 (315 °C)	178 (425 °C)	171 (540 °C)	163 (650 °C)	159 (705 °C)	147 (815 °C)	130 (925 °C)	110 (1040 °C)	[37]
800			191.3	184.8	178.3	171.6	165	157.7	150.1	141.3			[38]
G3			205	199	192	186	180	173	167				[39]

**Table 22:** Components of Ni-Cr-Mo-W-based alloys.

Components	Ni	Fe	Cr	Co	Mo	Cu	W	V	Ti	Al	Mn	Refs.
Alloy Designation	[%]											
Incoloy 825	38–46	balance	19.5–23.5	-	2.5–3.5	1.5–3	-	-	0.6–1.2	-	<1	[43]
Incoloy 925	42–46	>22	19.5–22.5	-	2.5–3.5	1.5–3	-	-	1.9–2.4	-	<1	[44]
Incoloy 926	46	-	22.5	-	3.5	3	-	-	2.4	-	1	[45]
Incoloy 800, 800H, 800HT	30.0–35.0	>39.5	19–23	-	-	<0.75	-	-	0.15–0.60	0.15–0.60	<1.5	[38]
Nimonic 86	Bal.	-	25	-	10	-	-	-	-	-		[46]
Nimonic 90	Bal.	<1.5	18–21	15–21	-	<0.2	-	-	2–3	1–2	<1	[47]
Hastelloy C-276	Bal.	4–7	14.5–16.5	<2.5	15–17		3–4.5	<0.35	-	-	<1	[48]
Hastelloy C-4	65	<3	16	<2	16	--		-	<0.7	-	<1	[49]

**Table 23:** Physical and mechanical properties of Ni-Cr-Mo-W-based alloys.

Alloy Designation		Incoloy 825	Incoloy 925	Incoloy 926	Incoloy 800	Nimonic 86	Nimonic 90	Hastelloy C-276	Hastelloy C-4
Property	Unit								
Density ρ	[g/cm^3^]	8.14	8.08	8.1	7.94	8.45	8.18	8.9	8.64
Resistivity at 20 °C	[Ω mm^2^/m]	1.127	1.17	-	0.989	-	1.18	1.3	1.25
Linear thermal expansion coefficient α,	[×10^−6^/K]	14	13.2	-	16.8–18	12.7	12.7	11.2	10.8
Thermal conductivity λ at 50 °C	[W/m K]	11.1	12	-	13	-	11.47	9.8	10
Specific heat capacity at 20 °C	[kJ/kg K]	440	435	500	502	-	446	427	416
Melting temperature	[°C]	1370–1400	1311–1366	1320–1390	1357–1385	-	1310–1370	2415–2500	-
Tensile strength	[MPa]	690	>965	600	517–827 (1034)	873	1175–1265	>690	>690
Yield point	[MPa]	324	>724	300	207–448 (862)	438	752–831	>283	>280
Elongation	[%]	45	>18	40	60–25	45	17–30	40	>35
Young Modulus	[GPa]	195	199		193	210	204–220	205	-
Magnetic properties (Curie point)	[°C]	−196	-	−30	−115	-	-	-	-
Hardness	[HB]	90	26–38HRC	86	138	-	-	90	<240 HBW
**Refs.**		[43]	[44]	[45,50]	[38]	[46]	[47]	[48]	[49]

**Table 24:** High-temperature strength and physical properties of certain Ni-Cr-Mo-W-based alloys.

Alloy Type	Property	Temperature [°C]	100	200	300	400	500	600	700	800	900	1000	Refs.
		Unit	Value										
825	Yield strength	[MPa]	279 (93 °C)	245 (204 °C)	232 (316 °C)	228 (427 °C)	229 (538 °C)	213 (649 °C)	183 (760 °C)	117 (871 °C)	47 (982 °C)	23 (1093 °C)	[43]
926			> 230	>190	>170	>160	-	-	-	-	-	-	[51]
86			-	-	251	-	243	-	239	173 (850 °C)	125	44 (1050 °C)	[46]
90			>635	>610	>585	>565	>545	>530	>500	>398	-	-	[47]
825	Tensile strength	[MPa]	655 (93 °C)	637 (204 °C)	632 (316 °C)	610 (427 °C)	592 (538 °C)	465 (649 °C)	274 (760 °C)	135 (871 °C)	75 (982 °C)	42 (1093 °C)	[43]
86			-	-	692	-	661	-	557	319 (850 °C)	237	98 (1050 °C)	[46]
825	Elongation	[%]	-	-	-	-	43 (538 °C)	62 (649 °C)	87 (760 °C)	102 (871 °C)	-	-	[43]
86			-	-	49	-	54	-	56	69 (850 °C)	66	50 (1050 °C)	[46]
825	Coefficient of thermal expansion, from 20 °C to	[μm/m·K]	14 (24–93 °C)	14.9 (24–205 °C)	15.3 (24–315 °C)	15.7 (24–425 °C)	15.8 (24–540 °C)	16.4 (24–650 °C)	17.1 (24–760 °C)	17.5 (24–870 °C)	-	-	[43]
925			13.2	14.2	14.7	15	15.3	15.7	16.3	17.2	-	-	[44]
926			15.8	16.1	16.5	16.9	17.3	-	-	-	-	-	[51]
800H			-	-	-	-	16.8 (26–500 °C)	17.1 (26–600 °C)	17.5 (26–700 °C)	18 (26–800 °C)	-	-	[38]
800HT			-	-	-	-	16.8 (26–500 °C)	17.1 (26–600 °C)	17.5 (26–700 °C)	18 (26–800 °C)	-	-	[38]
86			12.7	12.8	13.1	13.5	13.9	14.1	-	15.5 (850 °C)	-	16.8 (1050 °C)	[46]
90			12.7	13.3	13.7	14	14.3	14.8	15.3	16.2	17.1	18.2	[47]
C276			11.2 (24–93 °C)	12 (24–205 °C)	12.8 (24–315 °C)	13.2 (24–425 °C)	13.4 (24–540 °C)	14.1 (24–650 °C)	14.9 (24–760 °C)	15.9 (24–70 °C)	16 (24–925 °C)	-	[48]
C4			10.8 (93 °C)	11.9 (205 °C)	12.6 (315 °C)	13 (425 °C)	13.3 (540 °C)	13.5 (650 °C)	14.4 (760 °C)	14.9 (870 °C)	15.7 (980 °C)	-	[49]
825	Electrical resistivity	[nΩ·m]	1142 (93 °C)	1180 (205 °C)	1210 (315 °C)	1248 (425 °C)	1265 (540 °C)	1267 (650 °C)	1272 (760 °C)	1288 (870 °C)	1300 (980 °C)	-	[43]
800H			989	1035	1089	1127	1157	1191	1223	1266	1283	1291	[38]
800HT			989	1035	1089	1127	1157	1191	1223	1266	1283	1291	[38]
90			1210	1230	1260	1280	1310	1310	1310	1310	1300	1280	[47]
C4				1260		1280		1320					[49]
825	Thermal conductivity	[W/m·K]	12.3 (93 °C)	14.1 (205 °C)	15.8 (315 °C)	17.3 (425 °C)	18.9 (540 °C)	20.2 (650 °C)	22.3 (760 °C)	24.8 (870 °C)	27.7 (980 °C)		[43]
925			12.9	14.3	15.9	17.4	19.3	22.2	24	28.2	27.7	24.6	[44]
800H			13	14.7	16.3	17.9	19.5	21.1	22.8		21.1	31.9	[38]
800HT			13	14.7	16.3	17.9	19.5	21.1	22.8		21.1	31.9	[38]
90			11.47	12.77	14.44	15.99	17.54	18.97	20.64	22.32	23.99	25.83	[47]
C276			11.1 (93 °C)	13 (205 °C)	15 (315 °C)	16.9 (425 °C)	19 (540 °C)	20.9 (650 °C)	23 (760 °C)	24.9 (870 °C)	26.7 (980 °C)	28.2 (1090 °C)	[48]
C4			11.4	13.2	14.9	16.6	18.4	20.4					[49]
925	Specific heat capacity	[J/kg·K]	456	456	456	456	456	456	456	456	456		[44]
90			467	494	520	547	572	600	626	652	679	706	[47]
C4			426	448	465	477	490	502	-	-	-	-	[49]
925	Modulus of elasticity	[GPa]	195	188	182 (315 °C}	175 (427 °C)	168 (540 °C)	164	155	145 (815 °C)	132 (925 °C)	-	[44]
926			190	182	174	166	158	-	-	-	-	-	[51]
800H			191.3	184.8	178.3	171.6	165	157.7	150.1	141.3	-	-	[38]
800HT			191.3	184.8	178.3	171.6	165	157.7	150.1	141.3	-	-	[38]
86			206	201	195	189	183	176		155 (850 °C)	-	138 (1050 °C)	[46]
90			199	194	188	181	174	168	159	150	137	125	[47]
C276			-	195 (204 °C)	188 (316 °C)	182 (427 °C)	176 (538 °C)	-	-	-	-	-	[48]
C4			10 (93 °C)	11.4 (205 °C)	13.2 (315 °C)	14.9 (425 °C)	16.6 (540 °C)	18.4 (650 °C)	20.4 (760 °C)	18.4 (870 °C)	20.4 (980 °C)	-	[49]

**Table 25:** Components of Ni-Fe-Cr-Mo based alloys.

Components	Ni	Fe	Cr	Mo	W	Al	Co	Cu	V	Mn	Ti	Refs.
Alloy Designation	[%]											
Alloy HX	47	18	22	9	-	-	-	-	-	-	-	[20]
Hastelloy C-22	56.0	3	22	13	3	-	<3	-	<0.35	<0.5	-	[54]
Haynes 59	Bal.	<1.5	22–24	15–16.5	-	0.1–0.4	<0.3	<0.5	-	<0.5	-	[55]
Hastelloy C2000	Bal.	<3	22–24	15–17	-	-	<2	1.3–1.9	-	<0.5	-	[56]
Inconel 686	Bal.	<5	19–23	15–17	3–4.4	-	-	-	-	<0.75	0.02–0.25	[57]

**Table 26:** Physical and mechanical properties of Ni-Cr-Fe-Mo alloys.

Alloy Designation	Unit	HX	Hastelloy C-22	Haynes 59	Hastelloy C2000	Inconel 686	Inconel 617
Property							
Density ρ	[g/cm^3^]	8.22	8.7	8.6	8.5	8.73	8.36
Resistivity at 20 °C	[Ω mm^2^/m]	1.15	1.14	1.26	1.28	1.237	1.222
Linear thermal expansion coefficient α	[10^−6^/K]		12.4	11.9	12.4	11.97	12.6
Thermal conductivity λ at 50 °C	[W/m·K]	9.1	10.1	10.4	9.1	9.8	13.4
Specific heat capacity at 20 °C	[kJ/kg K]	485	420	414	428	373	419
Melting point (approx.)	[°C]		1357–1400	1310–1360	1328–1358	1338–1380	1332–1380
Tensile strength	[MPa]	690	765–800	690–900	752–758	740–848	734–769
Yield point	[MPa]	270	359–407	>340	345–372	359–396	318–383
Elongation at rupture	[%]	30	57–70	>40	61–68	45–71	56–62
Young Modulus	[GPa]	205	206	210	207	207	211
Hardness	[HB]	180	240 HBW		<240 HBW	<240 HBW	172–193 BHN
**Refs.**		[20]	[54]	[55]	[56]	[57]	[58]

**Table 27:** High-temperature strength and physical properties of certain Fi-Cr-Fe-Mo-based alloys.

Alloy Type	Property	Temperature [°C]	100	200	300	400	500	600	700	800	900	1000	Refs.
		Unit	Value										
C22	Yield strength	[MPa]			248 (316 °C)	241 (427 °C)	234 (538 °C)	221 (649 °C)	214 (760 °C)				[54]
59			>290	>250	>220	>190							[55]
C2000			317 (93 °C)	241 (204 °C)	214 (316 °C)	193 (427 °C)	193 (538 °C)	193 (649 °C)					[56]
686			323 (93 °C)	290 (204 °C)	288 (316 °C)	224 (427 °C)	261 (540 °C)						[57]
C22	Tensile strength	[MPa]			655 (316 °C)	634 (427 °C)	607 (538 °C)	572 (649 °C)	534 (760 °C)				[54]
59			>650	>615	>580	>545							[55]
C2000			724 (93 °C)	669 (204 °C)	634 (316 °C)	607 (427 °C)	572 (538 °C)	524 (649 °C)					[56]
686			691 (93 °C)	635 (204 °C)	602 (316 °C)	570 (427 °C)	545 (540 °C)						[57]
C22	Elongation	[%]			68 (316 °C)	68 (427 °C)	67 (538 °C)	69 (649 °C)	68 (760 °C)				[54]
59			>40	>40	>40	>40							[55]
C2000			68 (93 °C)	72 (204 °C)	70 (316 °C)	72 (427 °C)	69 (538 °C)	78 (649 °C)					[56]
686			69 (93 °C)	67 (204 °C)	60 (316 °C)	61 (427 °C)	69 (540 °C)						[57]
C22	Coefficient of thermal expansion, from 20 °C to	[μm/m·K]	12.4		12.6	13.1	13.7	14.3	14.9	15.5	15.9		[54]
59			11.9	12.2	12.5	12.7	12.9	13.1					[55]
C2000			12.4	12.4	12.6	12.9	13.2	13.3					[56]
686			11.97	12.22	12.56	12.87	13.01	13.18					[57]
C22	Electrical resistivity	[nΩ·m]		1240		1260		1280					[54]
59			1270	1290	1310	1330	1340	1330					[55]
C2000			1290	1300	1310	1320	1340	1350					[56]
686			1246	1257	1263	1272	1289	1295	1279				[57]
C22	Thermal diffusivity	mm^2^/s	3	3.5	3.9	4.2	4.6	4.8					[54]
C2000			2.9	3.3	3.6	4	4.3	4.7					[56]
C22	Thermal conductivity	[W/m·K]	11.1	13.4	15.5	17.5	19.5	21.3					[54]
59			12.1	13.7	15.4	17	18.6	20.4					[55]
C2000			10.8	12.6	14.1	16.1	18						[56]
686			11	12.8	14.8	16.6	18.6	21.4	23.5	25.3	26.4	29.6	[57]
C22	Specific heat capacity	[J/kg·K]	423	444	460	476	485	514					[54]
59			425	434	443	451	459	464					[55]
C2000			434	443	455	468	486						[56]
686			389	410	431	456	477	498	519				[57]
C22	Modulus of elasticity	[GPa]		197	191	185	179	174	168	160	152	144	[54]
59			207	200	196	190	185	178					[55]
C2000					191	180	173	166					[56]
686			205	197	193	185	183	173	165				[57]

**Table 28:** Components of Ni--Cr-Co-Mo based alloys.

Components	Ni	Fe	Cr	Mo	Al	Co	Cu	Mn	Ti	Si	Refs.
Alloy Designation	[%]										
Inconel 617	>44.5	<3	20–24	8–10	0.8–1.5	10–15	<0.5	<1.0	<0.6	<1.0	[58]

**Table 29:** Physical and mechanical properties of Ni-Cr-Co-Mo-based alloys.

Alloy Designation		Inconel 617
Property	Unit	Value
Density ρ	[g/cm^3^]	8.36
Resistivity at 20 °C	[Ω mm^2^/m]	1.222
Linear thermal expansion coefficient α	[×10^−6^/K]	12.6
Thermal conductivity λ at 50 °C	[W/m K]	13.4
Specific heat capacity at 20 °C	[kJ/kg K]	419
Melting point (approx.)	[°C]	1332–1380
Tensile strength	[MPa]	734–769
Yield strength	[MPa]	318–383
Elongation at rupture	[%]	56–62
Young Modulus	[GPa]	211
Hardness	[HB]	172–193 BHN
**Refs.**		[58]

**Table 30:** High-temperature strength and physical properties of Inconel 617.

Property	Temperature [°C]	200	300	400	500	600	700	800	900	1000
	Unit	Values								
Coefficient of thermal expansion, from 20 °C to	[μm/m⋅K]	12.6	13.1	13.6	13.9	14	14.8	15.4	15.8	16.3
Electrical resistivity	[nΩ·m]		1268	1278	1290	1308	1332	1342	1338	1378
Thermal conductivity	[W/m·K]		17.7	19.3	20.9	22.5	23.9	25.5	27.1	28.7
Specific heat capacity	[J/kg·K]		490	515	536	561	586	611	636	662
Modulus of elasticity	[GPa]		194	188	181	173	166	157	149	139

**Table 34:** Application of Ni-based alloys.

Alloy Group	Alloy Type	Application	Refs.
Ni pure	CP Ni	Industrial uses of CP Ni mainly focus on extremely corrosive settings (chemical sector) due to its exceptional resistance to corrosion and tarnishing. A variety of CP Ni alloys were specifically formulated for use in high-temperature applications and unique electrical requirements. CP Ni is utilized in the production of equipment for food processing, containers for chemicals, equipment for handling caustics, components for electrical and electronic devices, anodes for electroplating, heat exchangers, fluorescent lighting, and both decorative and protective coatings.	[5,12]
SSS Ni alloys		Industrial uses of are typically designed for corrosive environments and very high operating temperatures (up to 1200 °C). They are frequently utilized in the chemical, thermal processing, maritime, petrochemical, and aerospace sectors. For instance, Alloy 600 shows significant resistance to reducing environments, whereas Alloy 601 demonstrates outstanding oxidation resistance.	[3]
Ni-Cu Alloys	Monel	Ni-Cu Alloys (Monel) are utilized for producing equipment for chemical processing, valve stems, springs, pumps, shafts, fittings, heat exchangers, screw machine products, and marine apparatus.	[12]
Ni-Mo	B2, B3	Alloy B2 is commonly utilized in harsh reducing environments. Alloy B2 can be applied in the welded state, and it exhibits reduced vulnerability to stress corrosion cracking in various conditions. Alloy B2 must be avoided at temperatures ranging from 1000 °F to 1600 °F because it develops secondary phases that may reduce the material’s ductility. Alloy B-2 is appropriate for application in the chemical processing sector, particularly in regions where HCl acid, phosphoric acid, and sulfuric acid are utilized or handled. Alloy B-2 has additionally been utilized in manufacturing pharmaceuticals, acetic acid, ethylene alkylation, and herbicides. End-use applications encompass pumps, valves, mechanical seals, and rupture disks, flanges, fittings, tanks, and vessels. Alloy B2 has outstanding resistance to hydrochloric acid across a broad spectrum of concentrations and temperatures. Alloy B2 exhibits strong resistance to HCl, sulfuric acid, and phosphoric acids, and demonstrates outstanding resilience to pitting and stress corrosion cracking in the heat-affected zone. The uniform corrosion rates in different environments are comparable to those of other Ni-Mo alloys like alloy B3. The existence of any oxidizing substances, even in minimal quantities, will substantially enhance corrosion. Alloy B2 must not be utilized in oxidizing environments, as these alloys exhibit minimal to no resistance in those conditions. Alloy B3 exhibits outstanding resistance to hydrochloric acid across all concentrations and temperatures. It can also resist sulfuric, acetic, formic, and phosphoric acids, along with other non-oxidizing substances. The B3 alloy can reach a level of thermal stability significantly better than that of its predecessors, such as the B2 alloy. It shows remarkable resistance to pitting corrosion, stress-corrosion cracking, and attacks from knife-line and heat-affected zones. The enhanced thermal stability of alloy B3 leads to a lower likelihood of forming harmful intermetallic phases in alloy B3, thus granting it superior ductility compared to B-2 alloy under different thermal cycling conditions and afterward. B3 exhibits excellent overall characteristics for forming and welding. Similarly to B2 alloy, B3 should not be employed in environments containing ferric or cupric salts, as these salts can lead to quick corrosion failure. The top benefit of alloy B3 compared to B2 alloy comes from its capacity to retain outstanding ductility when subjected to short-term exposures at intermediate temperatures. The NiMo master alloy is utilized in manufacturing stainless steels, special steels, and superalloys for solid solution strengthening, precipitation hardening, deoxidation, desulfurization, and more. NiMo is employed for precipitation hardening and solid solution strengthening. NiMo is also utilized in creating intricate carbides in Ni-containing super alloys.	[21,24]
Ni-Fe	Nifethal 70, Nifethal 52, Alloy 36 Invar (UNS K93600),	Ni-Fe (NiFe) alloys for temperatures reaching 600 °C comprise Nifethal^®^ 70 and Nifethal^®^ 52 low-resistivity alloys possessing a high temperature coefficient of resistance. The positive temperature coefficient enables heating elements to decrease power as the temperature rises. Common uses include low-temperature tubular components with self-regulating characteristics Ni-Fe alloys are utilized as soft magnetic materials, for glass-to-metal bonding, and as materials with specified thermal expansion characteristics. Invar (UNS K93600) with an almost negligible coefficient of thermal expansion near room temperature can be used where high dimensional stability is necessary, like in precision measuring devices and thermostat rods. It is additionally utilized at cryogenic temperatures due to its extremely low thermal expansion rates. Alloys with 72–83% Ni possess optimal soft magnetic characteristics and are utilized in transformers, inductors, magnetic amplifiers, magnetic shielding, and memory storage devices Alloy 36 (Invar K93600) exhibits good strength and toughness at very low temperatures. Such an alloy can be used for tanks for liquid natural gas, measuring and thermostatic instruments.	[20,28,29]
Ni-Cr-Fe	800, 800H, 800HT, Inconel™ alloys 600, 601, 625, 718, 800, G3	Ni-Cr-Fe alloys are used in bearings, castings, ballast, step soldering, radiation protection, household appliances, and general heating devices. The physical and mechanical properties do not vary significantly between alloy 800, alloy 800H and alloy 800HT, especially at temperatures below 650 °C. Inconel (tm) alloys have excellent resistance to numerous corrosive substances. Except for a few cases, high-Ni alloys generally perform much better than martensitic, ferritic, and austenitic stainless steels in corrosive conditions.	[29,38]
Ni-Cr-Mo-W	Incoloy 825, Incoloy 925, Incoloy 926, Nimonic 86, Nimonic 90, Alloy C276, Hasteloy C4	Incoloy 825 has outstanding resistance to phosphoric and sulfuric acids. The alloy is resistant to a broad spectrum of corrosive conditions and intergranular sensitization. It is resistant to oxidizing and reducing acids, stress corrosion cracking, pitting, and intergranular corrosion, making it suitable for chemical and petrochemical processing, oil and gas extraction, pollution management, waste treatment, and pickling uses. Alloy 825 is utilized in phosphoric acid evaporators, pickling machinery, vessels and piping for chemical processing, equipment for recovering spent nuclear fuel, propeller shafts, and tank trucks. Incoloy 925 is a corrosion-resistant, high-strength Ni-Cr-Fe alloy that is PS, featuring additions of Ti, Al, Mo, and Cu. It can be considered an enhanced variant of alloy 825 that has similar corrosion resistance but offers superior strength characteristics due to precipitation hardening. Alloy 925 is mainly utilized in the oil and gas sector for drilling and surface gas well parts, such as piping, valves, and fasteners. It is also utilized in the maritime sector. Incoloy 926 exhibits enhanced corrosion resistance against various aggressive environments. The resistance to pitting and crevice corrosion in halide media was significantly enhanced. It exhibits the stability of metallographic structures and the reduced tendency for intergranular separation during thermal or welding processes. It has enhanced tolerance to sulfuric acid, high yield and tensile strength. It is relevant in multiple systems such as fire protection, water treatment, marine engineering, and hydraulic piping flow, as well as in the elements utilized in acidic gas and phosphate manufacturing. It can also be utilized in power generation facilities for cooling sewage water in condensation and piping systems, as well as for producing acidic organic catalyst chlorinated derivatives, cellulose pulp, polished bars in corrosive oil wells, hose systems in ocean engineering, components of flue gas desulfurization systems, sulfuric acid condensation and separation systems, transportation of corrosive chemical containers, and reverse osmosis desalination plants. Nimonic 86 exhibits outstanding resistance to cyclic oxidation at 1050 °C, is ductile, suitable for welding, and resistant to creep. Nimonic 86 is utilized in aerospace (gas turbine combustion chambers, afterburner components) and thermal processing (heat-treatment furnace machinery). Nimonic 90 can withstand heat and creep up to 920 °C. It is utilized for gas turbine parts (blades, disks, forgings, rings), within the automotive sector, for hot-working tool parts, and for springs that operate at high temperatures. Alloy C276 (Hastelloy C276) possesses remarkable resistance to heat and corrosion. It is utilized in chemical processing, pollution management, pulp and paper manufacturing, industrial and municipal waste treatment, as well as the retrieval of sour natural gas. Its uses encompass stack liners, ducts, dampers, scrubbers, stack-gas reheaters, fans, fan enclosures, heat exchangers, reaction vessels, evaporators, and transfer pipes. Hasteloy C4 exhibits remarkably high-temperature stability, exceptional resistance to harsh aqueous conditions, and satisfactory weldability. It can be viewed as a more stable and superior weldable version of Alloy C276. Alloy C4 exhibits favorable ductility and corrosion resistance following extensive aging at temperatures ranging from 650 to 1040 °C. It shows great resistance to stress-corrosion cracking and oxidizing environments up to 1040 °C.	[43,44,45,46,47,48,49]
Ni-Fe-Cr-Mo	Alloy HX, Alloy 59, Alloy 2000, Alloy 686	Alloy HX/2.4665 exhibits an excellent combination of oxidation resistance, fabricability and high-temperatures strength. This alloy can be used for components for gas turbines and industrial furnaces. Alloy C22 (N06022) has improved thermal stability compared to Alloy C276 and enhanced resistance to chloride-induced localized corrosion and stress-corrosion cracking over Alloy C4. Alloy C22 possesses versatility across various corrosive conditions. Alloy C22 is utilized in the production of acetic acid, manufacturing of cellophane, systems for chlorination, intricate acid mixtures, electro-galvanizing rollers, expansion bellows, flue gas scrubbing systems, systems for HF scrubbing, geothermal wells, incineration scrubber systems, reprocessing of nuclear fuel, production of pesticides, creation of phosphoric acid, systems for pickling, plate heat exchangers, selective leaching systems, cooling towers for SO_2_, sulfonation systems, tubular heat exchangers, and valve weld overlays. Alloy 59 (UNS N08031) is a corrosion-resistant alloy. It is a modification of alloys C-2000 and C-4, created explicitly for harsh environments in flue gas desulfurization systems. It exhibits superior thermal stability compared to C2000 and C4, and it achieved NACE MR 0175/ISO 15156 Level VII resistance, making this alloy quite appealing for the Oil andGas sector. It is approved for the transportation of dangerous materials. It is primarily utilized in chemical processing and pollution management. Common uses include scrubbers, heat exchangers, ventilators, and agitators for flue gas desulfurization in fossil fuel power stations and waste combustion facilities, SO_2_-washers for marine diesel engines, reactors for acetic acid, acetic anhydrides, hydrofluoric acid, coolers for sulfuric acid, and pipes in geothermal energy plants. Alloy 2000 (N06200, Hastelloy C2000) can be regarded as an enhanced variant of C-22, exhibiting overall superior resistance to both reducing and oxidizing conditions. Ductile, simple to weld, resistant to pitting, crevice attack, and stress corrosion cracking, it stands out as one of the most adaptable alloys utilized in chemical processing. The blend of approximately 16% Mo and around 1.6% Cu offers protection against reducing agents (such as dilute hydrochloric or sulfuric acids), while about 23% Cr content ensures strong resistance to oxidizing acids. It is used in the components of chemical processing plants—such as reactors, heat exchangers, valves, and pumps—for environments that are both oxidizing and reducing. Alloy 686 (UNS N06686) withstands very aggressive oxidizing (due to high Cr) and reducing (Ni and Mo) conditions. Due to its minimal Fe and C levels, along with the combination of Mo and W, it provides excellent resistance to localized corrosion like pitting and crevice. Low C preserves corrosion resistance in the heat-affected areas of welded joints. It is utilized in chemical processes, pulp production, paper production, pollution management, and waste disposal applications.	[20,54,55,56,57,58]
Ni-Cr-Co-Mo	Alloy 617	Alloy 617 (UNS N06617/W.Nr. 2.4663a) is known for its strength at elevated temperatures, excellent creep resistance, and oxidation durability. It also possesses strong resistance to environments with high-temperature carburizing and nitriding. It is utilized for gas turbines, petrochemical and thermal processing, as well as for the production of nitric acid.	[58]
Ni-Cu-Al-Ti	Alloy K-500	Alloy K-500 is commonly used in the marine, chemical processing, oil and gas, pulp and paper, pharmaceutical, food processing, and electronics sectors. Applications for the end use of alloy K-500 encompass fasteners, springs, chains, components for pumps and valves, drill collars, doctor blades, scrapers, mixing shafts, impellers, sensors, electrical parts, and various other highly corrosive uses where both strength and hardness matter. The corrosion resistance of alloy K-500 is comparable to that of alloy 400. Nonetheless, in its age-hardened state, alloy K-500 may undergo stress corrosion cracking in specific environments. The resistance of alloy K-500 to H_2_S renders it valuable in sour gas settings, making it a perfect option for use in the oil industry. The minimal corrosion rates in seawater render alloy K-500 ideal for applications in the marine sector. Pitting can happen in stagnant or slow-moving seawaters, but the pitting rate eventually diminishes after the initial onset.	[61]
Ni-Cr-Al-Ti	Alloy 80A, Alloy 90, Alloy C263,	Alloy 80A (UNS N07080) is age-hardened, heat-resistant, creep-resistant, and provides resistance to S in combustion gases. It operates at temperatures up to 815 °C. It finds application in the automotive industry for exhaust valves, in aerospace for fasteners, gas turbine blades, rings, and disks, and in nuclear power facilities for boiler tube supports. Alloy 90 (UNS N07090) is wrought and age-hardened, with additions of Ti and Al as alloying elements. It is resistant to heat and creep up to 920 °C. It is utilized for components in gas turbines (blades, disks, forgings, rings), in the automotive sector, for hot-working tool parts, and for high-temperature springs. Alloy C263 (UNS N07263) is resistant to creep, PS, and is designed for high temperatures. It is provided in the annealed state. It is suggested for service temperatures reaching 850 °C, though it can withstand oxidation up to 1000 °C. It welds easily (showing no tendency for cracking after weld heat treatment), possesses favorable fabrication properties, offers excellent wear resistance, and does not demonstrate tensile ductility at intermediate temperatures. It is utilized in aerospace and industrial gas turbines for combustion chambers at low temperatures, transition liners, and exhaust cones and rings.	[47,62,63]
Ni-Fe-Cr-Nb-Al-Ti	Haynes 282, Inconel 713, Waspaloy, René 41, Inconel 725, Inconel 706, Incoloy 909, Incoloy 945	Haynes 282 is designed for structural uses at elevated temperatures, demonstrating superior creep strength within the range of 650–930 °C, outperforming both Waspalloy and Rene 41. Haynes 282 features a distinctive blend of creep strength, thermal stability, weldability, and fabricability that is absent in presently available commercial alloys. Its characteristics render it appropriate for essential gas turbine uses, including combustors, turbine and exhaust parts, and nozzle elements. Inconel 713 excels in high-temperature environments and under severe mechanical stress. A key benefit is its capacity to sustain stability at high temperatures while providing outstanding creep resistance. It retains mechanical strength up to 980 °C. Inconel 713 displays excellent resistance to distortion at elevated temperatures. It demonstrates outstanding resistance to oxidation. It is perfect for gas turbines and engine parts that function at elevated temperatures. It is not as appropriate for marine settings as Inconel 625, but provides better thermal stability. It is commonly favored for parts subjected to elevated temperatures, like in aerospace, gas turbine, and energy industries. Its appropriateness for casting applications provides a major benefit in manufacturing intricate engine components. Waspaloy hardens with age, designed for use in gas turbine applications. Resistant to creep and heat, it is utilized in combustion environments up to 870 °C. Nevertheless, in crucial and demanding applications, the highest operating temperature must not surpass 650 °C. This is used for gas turbine parts in the aerospace sector, encompassing compressor disks, rotor disks, shafts, spacers, seals, rings, casings, fasteners, and more. René 41 (UNS N07041) has high age-hardening and creep resistance. It is suitable for components in jet engines and high-speed airframes, including fasteners, wheels, turbine casings, and afterburner parts. Inconel 725 Alloy 725 (N07725) is an age-hardenable version of alloy 625, offering similar corrosion resistance but greater strength. It withstands ordinary corrosion, pitting, sulfide stress cracking, hydrogen embrittlement, and stress-corrosion cracking in sour gas wells. It can be utilized for hangers, landing nipples, side pocket mandrels, polished bore receptacles in sour gas operations, high-strength fasteners in marine use, polymer extrusion dies. Inconel 706 (UNS 09706) has excellent mechanical strength along with good workability. The properties of the alloy resemble those of Inconel alloy 718, except that alloy 706 is easier to fabricate, especially through machining. It has excellent protection against oxidation and corrosion. Inconel 706 has outstanding resilience against post-weld strain-age cracking. Inconel alloy 706 is utilized in numerous applications that demand high strength along with easy fabrication. In the aerospace sector, the alloy is utilized for turbine disks, shafts, and housings; diffuser housings; compressor disks and shafts; engine supports; and connectors. Besides aerospace uses, the alloy is utilized for turbine disks in major industrial gas turbines. Incoloy 909 (UNS N19909) has a consistently low coefficient of thermal expansion, a stable modulus of elasticity, and impressive strength. The alloy is reinforced through a precipitation-hardening heat treatment enabled by the inclusion of niobium and Ti. The merging of low expansion with high strength renders Incoloy alloy 909 particularly advantageous for gas turbines. The reduced expansion allows for tighter regulation of clearances and tolerances, resulting in improved power output and fuel efficiency. The increased strength enhances strength-to-weight ratios, resulting in lighter aircraft engines. Due to these factors, Incoloy alloy 909 is utilized for vanes, casings, shafts, and shrouds in gas turbines. The characteristics of Incoloy alloy 909 are appealing for use in rocket-engine thrust chambers, ordnance components, springs, steam-turbine bolts, gauge blocks, instrumentation, and glass-sealing applications. Incoloy 945 (UNS N09945) is a robust, corrosion-resistant material intended for rigorous applications in the oil and gas sector. The alloy 945 has high resistance against stress corrosion cracking induced by chlorides. The alloy 945 possesses exceptional resistance to general corrosion in reducing environments. It also has a high resistance to localized damage (such as pitting and crevice corrosion). The alloy has protection in oxidizing conditions and enhanced strength. Incoloy alloy 945 is ideal for downhole oil and gas applications that demand high strength and resistance to corrosion in harsh sour wells with elevated levels of H_2_S and HCl. Due to its resistance to stress cracking in H_2_S-rich environments, the alloy is appropriate for gas-well components both downhole and on the surface, such as tubular products, hangers, valves, landing nipples, tool joints, and packers. The alloy can additionally be utilized for fasteners, pump shafts, and robust piping systems.	[64,66,67,68,69,70,71,72]

**Table 35:** Mechanisms of cracking in various Ni-based alloys.

Alloy Type	Mechanism of Cracking	Refs.
**Pure Ni**	It is not very prone to stress corrosion cracking, except in conditions of intense cold work at elevated temperatures (>250 °C) with concentrated caustic solutions and liquid metal. It could be vulnerable to hydrogen embrittlement. In neutral 1.5 m LiAlC14/SOCl2 solutions with cathodic applied potential and slow strain rate, zero valence Li leads to intergranular and transgranular cracking. In a battery electrolyte containing 1.5 m LiAlC14/SOCl2 during cathodic polarization, intergranular SCC is observed in the powder metallurgy (PM) alloy, while the wrought C+W alloy shows no cracking. During cathodic hydrogen charging in a 1 N H_2_SO_4_ solution for 24 h followed by bending in air, intergranular cracking takes place in both types of materials. Hydrogen influences the cracking vulnerability of Ni-200 and Ni-270 samples under slow-strain-rate conditions. In air, the fracture mode of Ni-200 exhibits dimples, while in the H_2_SO_4_ solution, it reveals a combination of quasi-cleavage and intergranular cracking, indicating the influence of hydrogen-induced cracking. The presence of sulfur at grain boundaries may worsen hydrogen-induced cracking in Ni. Under continuous stress in a battery electrolyte melt comprising NaAlCl4 + 2% S at 300 °C during standard battery cycling voltages of 2–3 V, Ni-200 wires show no signs of cracking even after over 1400 h.	[382]
**Ni-Cu alloys**	Alloy 400 has low susceptibility to stress corrosion cracking (SCC) because of its reduced mechanical strength and increased ductility. Alloy 400 is vulnerable to SCC in acidic environments with mercury salts, in liquid mercury, in hydrofluoric acid, and in fluosilicic acid. The age-hardenable alloy K-500 might be vulnerable to HE. Multiple oil wells in the K-500 field experienced cracking, primarily characterized by an intergranular cracking pattern. The failures could be linked to a hydrogen embrittlement process likely caused by cathodic protection or because the alloy was paired with a carbon steel that has lower corrosion resistance.	[382]
**Ni-Mo alloys**	Their resistance to SCC in hot concentrated chloride solutions is attributed to the presence of 70% Ni. Samples of 20%, 33%, and 50% cold-worked alloys B and B-2 showed no cracking after a week of testing in 25% NaCl and comparable chloride concentrations of CaCl2 and MgCl2 at 121 °C, 149 °C, 177 °C, 204 °C, and 232 °C. In other words, cracking did not happen, even though the hardness for the 50% cold-worked alloy B-2 could reach up to HRC 49. Alloy B-2 and, to a lesser extent, alloy B-3 lose ductility when subjected to temperatures between 550 and 850 °C, caused by a solid phase transformation that results in the creation of ordered intermetallic phases like Ni_4_Mo. The formation of these ordered phases alters the deformation mechanisms of the alloys, rendering them vulnerable to EAC, including hydrogen embrittlement. The precipitation kinetics of harmful phases like Ni_4_Mo in alloy B-2 are very responsive to minor changes in chemistry and past thermal treatments, and certain compositions can develop these harmful ordered phases within minutes when exposed to the critical temperature range of 650–750 °C, such as in the heat affected zone (HAZ) during welding. The B-3 alloy was created to enhance the development of intermetallic and short ordering phases during welding, leading to greater resistance to SCC. B-2 alloy experienced intergranular stress corrosion cracking in the HAZ when subjected to organic solvents with sulfuric acid traces at 120 °C. B-2 alloy is susceptible to transgranular stress corrosion cracking when exposed to hydroiodic acid (HI) at temperatures above 177 °C. In the investigation of stress corrosion cracking of B, B-2, and B-3 alloys in acidic solutions, transgranular fissures were observed in all three alloys at anodic potentials (200 mV above the free corrosion potential) for both mill-annealed and aged materials. At cathodic potentials (100 mV and 400 mV below the free corrosion potential), intergranular cracking was observed solely in the aged (sensitized) alloys. The rise in intergranular brittle cracking at the reduced applied cathodic potential is linked to hydrogen embrittlement, which causes this environmentally induced cracking.	[382]
**Ni-Cr-Mo alloys**	Alloys like C-276, made of Ni-Cr-Mo, are the most resistant nickel-based alloys to conventional localized corrosion caused by chlorides, which affects and restricts the application of austenitic stainless steels. SCC was noted in high-strength Ni-Cr-Mo materials in certain instances; however, cracking occurred exclusively under highly aggressive conditions, such as temperatures exceeding 200 °C, pH levels below 4, and in the presence of hydrogen sulfide. Samples of C-2000, C-22, and C-276 alloys showed no cracking in boiling (154 °C) 45% MgCl2 solution following 1008 h of testing. C-276 and C-4 alloy did not exhibit cracking in a 25% NaCl solution at 232 °C; however, these alloys were prone to cracking in a MgCl2 solution with the same chloride concentration at the same temperature. C-22 alloy showed resistance to SCC in a 20.4% MgCl2 solution at temperatures up to 232 °C, even when subjected to a 50% cold-worked state and a condition of 50% cold-worked plus aging at 500 °C for 100 h. In tests conducted at the yearly average relative humidity of 72% and temperature of 18.5 °C, while also exposing the air to sulfur dioxide, nitrous oxides, and hydrochloric acid, the Ni alloys C-4 and 625 showed no signs of cracking. Aging Ni-Cr-Mo alloys at temperatures exceeding 600 °C for extended periods (for instance, 1000 h at 650 °C) can lead to long-range ordering reactions and the precipitation of tetrahedrally close-packed (TCP) phases (m, P, s). The existence of TCP phases caused by thermal aging can significantly decrease the ductility of Ni-Cr-Mo alloys. After aging at 649 °C for nearly 2 years, alloys C-276 and 625 experienced a 50% reduction in ductility following 4000 h of aging. The alloy with the highest resistance to aging was C-4, which maintained over 40% ductility even after being aged at 649 °C for 16,000 h. Alloy C-4 exhibits greater resistance to thermal aging effects because of its reduced quantity of alloying elements. The C-276 alloy that had been thermally aged was prone to hydrogen-induced cracking in H_2_S environments. C-276 alloy can be utilized at temperatures up to 218 °C in environments with a partial pressure of hydrogen sulfide reaching 700 kPa and any level of chloride concentration, along with the appropriate in situ pH, as long as the hardness does not exceed 40HRC and the yield strength remains below 1034 MPa. C-276 is applicable at any chloride concentration and at any partial pressure of H_2_S and CO_2_ up to 260 °C. Elemental sulfur’s presence can lead to severe cracking in this alloy. Ni-Cr-Mo alloys are also prone to environmentally induced cracking under conditions related to supercritical water oxidation (SCWO). C-276 and Alloy 625 experienced intergranular cracking when subjected to different aqueous solutions near the critical point of water (374 °C). An Alloy C-276 preheater tube experienced intergranular cracking while introducing a methylene chloride waste into a SCWO system; nonetheless, the cracking took place under subcritical conditions. Cracking appeared in an Alloy 625 tube that was intermittently exposed for 300 h at 425 °C to an aqueous solution with HCl at pH = 2. Cracking occurred in Alloy C-22 tubing after being exposed for 53 h at 350 °C to a HCl feed with a pH of 0.48, whereas no cracking occurred at a pH of 4.4 even after 70 h.	[382]
**Ni-Cr-Fe-(Mo) alloys**	Alloy 600 experiences stress corrosion cracking in high-temperature pure water (>300 °C) in both laboratory settings and during service. The cracking susceptibility of Alloys 600 and 690 is highly influenced by environmental factors including temperature, tensile stress levels, deformation rate, hydrogen gas presence, solution pH, and electrochemical potential, as well as metallurgical factors like the presence of minor alloying elements (impurities), the extent of cold work, and heat treatment (intragranular or intergranular carbides). In nuclear service, Alloy 600 primarily experiences intergranular cracking (IGSCC). In certain instances (e.g., lead contamination from the secondary side), cracking may be transgranular (TGSCC). Proper water chemistry practices reduce the occurrence of cracking in Alloy600 tubing but do not completely prevent it. Key variables influencing SCC from the electrolyte perspective include the pH level of the solution, the presence of impurities like lead, and the amounts of dissolved hydrogen and oxygen, which also affect the system’s electrochemical potential. The cracking of Alloy 600 tubing on the secondary side is generally more harmful in areas with occluded zones or crevices near the tubes, where the electrolyte can turn alkaline (increased pH levels). The existence of lead can alter the cracking pattern of Alloy 600 and 690 in high-temperature aqueous solutions from intergranular to transgranular. The impact of Pb on the SCC of Ni alloys could also be influenced by additional factors in the system, including the alloys’ metallurgical condition and the pH level of the electrolyte. Temperature plays a crucial role in governing both the initiation and propagation of SCC in nickel alloys like Alloy 600. As the temperature increases, Alloy 600 becomes more susceptible to SCC. The partial pressure of hydrogen is a crucial factor influencing the SCC susceptibility of Ni alloys. The hydrogen effect influences the electrochemical potential within the system. The greatest susceptibility of Ni alloys (such as X-750 and 600) to SCC is observed at a moderate partial pressure of hydrogen, leading to a limited potential range (~100 mV) associated with the transformation of Ni metal into Ni oxide (NiO). When a higher hydrogen partial pressure prevents the formation of NiO, susceptibility to SCC decreases, and with a lower hydrogen partial pressure, when a protective oxide forms, susceptibility to SCC is also diminished. The partial pressure of hydrogen required to achieve the peak of maximum susceptibility depends on other factors, including alloy composition, solution pH, and temperature. Hydrogen gas, lowering the electrochemical potential in the system, reduces the likelihood of SCC. The likelihood of environmental cracking in structural materials rises with the system’s potential. In commercial power plants, hydrogen gas is introduced to the water to keep a low potential and consequently reduce the likelihood of cracking. When platinum nanoparticles are used on the alloy components’ wet surfaces, reduced quantities of hydrogen gas are required in the high-temperature water to achieve a comparable low protective potential. Alloy 690, containing twice the chromium of Alloy 600, exhibits greater resistance to high-temperature cracking in pure water and caustic solutions compared to Alloy 600. In steam generator applications, Alloy 800 is typically more crack-resistant than Alloy 600, likely due to the intermediate nickel composition found in Alloy 800. Precipitation-hardened high-strength AlloyX-750, like Alloy 600, is prone to SCC in elevated temperature water typical of nuclear reactors. Alloy X-750 may also be vulnerable to hydrogen embrittlement at temperatures under 150 °C. Alloy 718 is also susceptible to hydrogen-induced cracking at low temperatures. Alloy 825 exhibits greater resistance to stress corrosion cracking in chloride solutions compared to 316 stainless steel (S31600) because of its higher nickel content. Alloy825 is prone to transgranular stress corrosion cracking in 45% MgCl2 solutions when temperatures exceed 146 °C. Factors in the environment that could influence the stress cracking behavior of Alloy 825 (as well as other alloys) in oil and gas wells encompass temperature, chloride concentration, and the presence of hydrogen sulfide gas. G-30 parts utilized in the industrial manufacturing of hydrofluoric acid experience cracking. G-30 alloy specimens remained intact after 500 h in a 45% MgCl2 solution at 154 °C. Like other nickel alloys, G-30 experiences cracking under the harsh conditions present during super critical water oxidation (SCWO) processes.	[382]
**Ni-Cr-Al-Ti**	Alloy 80A (Nimonic 80A) is susceptible to cracking because of several reasons, mainly during welding, high-temperature applications, and under mechanical strain. The microsegregation of alloying elements such as chromium during the welding process may result in the creation of carbides (M_23_C_6_), which can cause hot cracking. Oxidation at grain boundaries under high temperatures can result in both cracking and recrystallization. Hot cracking in welding is affected by: Microsegregation—while welding, microsegregation of alloying elements such as chromium may take place in the fusion zone. This results in the creation of carbides (M_23_C_6_) at grain boundaries, potentially diminishing ductility and raising the risk of cracking. Welding methods—various welding methods (such as Gas Tungsten Arc Welding—GTAW and Pulsed Current Gas Tungsten Arc Welding—PCGTAW) influence microsegregation and minimize cracking. Filler Wires—the kind of filler wire utilized can also affect cracking. For instance, using ERNiCrMo-3 filler wire in PCGTAW can lessen microsegregation and the formation of intermetallic phases when compared to GTAW. Cracking induced by oxidation is affected by: Grain boundary oxidation—at high temperatures (such as 550 °C), oxidation may happen at grain boundaries in Alloy 80A, producing oxides such as NiCr2O4 and NiCrO3. Recrystallization—the oxidation process may result in a recrystallized nickel layer forming at the grain boundaries, which can be prone to cracking because of its softness. Stress concentration—the oxidation process may additionally result in stress concentrations at the grain boundaries, which further promote cracking. Fracturing under mechanical pressure include: Creep—Alloy 80A may show creep crack propagation when stressed at elevated temperatures. Fracture resilience—the fracture toughness of Alloy 80A can be influenced by elements such as boriding, which may enhance hardness and vulnerability to cracking. High-Angle Grain Boundaries (HAGB)—cracks in Alloy 80A have been noted to spread intergranularly, frequently associated with high-angle grain boundaries (HAGB). Cracking during solidification is affected by: Melt pool instability—in techniques such as laser beam melting (LBM), fast cooling and solidification may cause instability in the melt pool and fractures, particularly in high-strength alloys. Residual stresses—elevated internal residual stresses can also play a role in crack formation and growth during the solidification process. Fracture repair—methods such as remelting may be utilized to repair cracks and decrease Alloy 80A’s vulnerability to cracking	[383,384,385,386,387]
	Alloy 90, a copper-nickel alloy, is prone to different cracking mechanisms, such as stress corrosion cracking (SCC) and sulfide stress cracking (SSC). SCC may take place in environments rich in sulfides, resulting in the dealloying (selective dissolution of copper), whereas SSC is affected by hydrogen presence and typically occurs at temperatures under 90 °C. Stress Corrosion Cracking (SCC): In concentrated sulfide solutions, SCC of 90/10 Cu-Ni alloy can occur through a dealloying process that selectively dissolves copper, resulting in a nickel-rich structure. This form of cracking is more probable to happen under conditions of low strain rate. Sulfide Stress Cracking (SSC)—is a cracking mechanism induced by hydrogen that may happen in settings with sulfide and elevated hydrogen levels. Elements such as weld metal hardness, tensile strength, and the existence of residual stresses can affect SSC susceptibility. Post-weld heat treatment (PWHT) can assist in alleviating SSC by lowering hardness and relieving stress. Additional elements influencing cracking include: Microstructure—the microstructure of Alloy 90, especially the nature of grain boundaries, can affect the initiation and spread of cracks. Processing parameters—elements such as heat input, scanning methods, and residual stress presence in additive manufacturing techniques (such as laser powder bed fusion) can influence the tendency to crack. Solute distribution—during solidification, solute separation can generate regions susceptible to cracking, particularly in the mushy zone where the alloy shifts from solid to liquid. Thermal evolution history—the alloy’s thermal history, encompassing cooling rates and preheating temperatures, can influence cracking mechanisms.	[388,389,390,391,392,393,394,395,396,397,398,399,400,401,402]
	In C263, a nickel-based superalloy, the main cracking process is creep cracking, which is greatly affected by carbide precipitation and the behavior of grain boundaries that follows. This cracking can be reduced through precise management of alloy composition and heat treatment methods. Creep cracking—C263 is prone to creep cracking, particularly at elevated temperatures and when subjected to constant stress. Creep refers to the slow alteration and breakdown of a material when subjected to stress, even if the stress remains beneath the yield strength. Carbide precipitation—the existence and arrangement of carbides, especially at grain boundaries, are vital in the initiation and progression of creep cracks. Carbides are tough, fragile compounds created during the alloy’s thermal processing. Grain boundary characteristics—grain boundaries are the surfaces that separate varying crystalline orientations within the material. Carbides located at grain boundaries can obstruct their capacity to slip and deform when under stress, resulting in stress concentration and the possible onset of cracks. Effects on crack formation and development—the density and dimensions of carbides at grain boundaries can influence the critical flaw size, representing the largest defect a material can endure without failing under creep conditions. Increased carbide densities can result in reduced critical flaw sizes. Effect of thermal treatment—the heat treatment method employed to produce C263 can markedly affect carbide formation and the behavior of grain boundaries. Effective management of heat treatment parameters can enhance the alloy’s ability to resist creep cracking. Notch sensitivity—the existence of notches (minor flaws or dips) can additionally worsen creep cracking. In certain instances, notched samples might break sooner than smooth samples, suggesting notch sensitivity	[403,404,405,406]
**Ni-Fe-Cr-Nb-Al-Ti**	Haynes 282 alloy may experience cracking from multiple mechanisms, such as hot cracking in welding, HAZ cracking, and the propagation of fatigue cracks. These fractures can be affected by elements such as heat input while welding, thermal cycling, and the microstructure of the material. Hot Cracking—a form of cracking that takes place during solidification or welding while the material remains hot, can pose a considerable problem in Haynes 282. In certain instances, hot cracking can result in a liquid phase appearing at grain boundaries, which may subsequently cause cracking. Elements such as the heat applied during welding and the duration the material remains at maximum temperatures can affect the likelihood of hot cracking. Cracking in the Heat-Affected Zone (HAZ)—found in the region of the material that is heated but not melted during welding—is also an issue with Haynes 282. HAZ cracking is frequently linked to a loss of hot ductility caused by subsolidus grain boundary liquation. Reducing the heat input while welding may elevate the chances of cracking in the HAZ. Fatigue crack growth—Haynes 282 may show fatigue crack progression when subjected to cyclic loading. The fracture path usually follows a transgranular route at ambient temperature. Rising temperatures can elevate the rates of fatigue crack growth, and this rise is unrelated to creep or oxidation mechanisms. Elements such as temperature, loading frequency, and stress intensity can affect the speed of fatigue crack growth. Additional factors include: Thermal aging—aging processes can result in the formation of grain boundary carbides and other phases, which may influence mechanical properties and crack propagation. Microstructure—the microstructure of Haynes 282, featuring precipitates and carbides, can affect the propagation of cracks. Thermal processing—heat treatments can influence the microstructure and mechanical characteristics of Haynes 282, subsequently impacting its cracking behavior.	[407,408,409,410]
	Inconel 713, a nickel-chromium-based superalloy, is susceptible to cracking due to a few key mechanisms, primarily related to solidification and thermal stresses. Solidification cracking (Hot cracking)—Inconel 713C, specifically, is susceptible to cracking during solidification because of the development of low-melting-point eutectic mixtures at grain boundaries. This happens when carbides (such as NbC) partially liquefy, resulting in the creation of a thin liquid layer that breaks during deformation because of thermal stresses and solidification shrinkage. Cracks generally appear at angles to the welding direction, especially within the fusion zone. Ductility Dip Cracking (DDC)—Inconel 713C and comparable alloys may encounter cracking within the ductility dip temperature range (DTR) during the solidification process, as ductility is reduced. This commonly results from the development of microvoids at grain boundaries or the partial melting of carbides, combined with thermal stresses and reduced ductility in the DTR. In this instance, cracks can occur along the grain boundaries or dendritic boundaries. Segregation-triggered liquation fracturing—as solidification occurs, elements may separate at grain boundaries, resulting in liquation (partial melting) and eventual cracking when under stress. This is particularly significant in processes like electron beam welding (EBW) and laser powder bed fusion (LPBF), where quick cooling can worsen segregation. Contraction stresses—contraction from solidification during welding or other processes can produce tensile stresses that, when paired with mechanisms such as liquation or DDC, may result in cracking. Thermal strains—irregular heating and cooling during welding or similar processes can lead to thermal stresses that promote cracking, especially alongside other factors such as DDC or segregation.	[65,411,412]
	Waspaloy, a nickel-containing superalloy, may experience cracking due to multiple reasons, such as stress during cooling, fatigue, and environmental deterioration. Particularly, crack initiation may happen within groups of grains that have advantageous slip transmission, resulting in planar slip being the primary deformation mechanism. Cracks mostly spread through the grains due to stress buildup at grain boundaries and weak bonding between interfaces. Thermal Cycling—cycles of repeated heating and cooling can result in stress accumulation, particularly during quick cooling, potentially leading to cracks. Impact of Grain Boundaries—cracks frequently develop along grain boundaries because of stress concentration and weakened bonding strength between the matrix and intergranular carbides. Slip transmission—beneficial crystallographic connections among grains can promote slip transfer, resulting in crack formation at those sites. Fatigue crack propagation is characterized by: Irreversible Plastic Flow at the Crack Tip—crack propagation is fueled by plastic flow at the crack tip, generating the necessary force for crack growth. Effects dependent on time—at lower frequencies, the rates of crack growth are affected by temperature, frequency, duration of holding, and stress ratio, possibly including creep or environmental deterioration. Environmental decline—the presence of air can greatly influence high-temperature fatigue crack growth, as lower crack growth rates are seen in a vacuum. Brief exhaustion fractures—short fatigue cracks may show varying initiation and propagation characteristics in contrast to long cracks. Alternative Factors include: Hydrogen embrittlement—hydrogen may gather at voids and inclusions, raising pressure and possibly causing cracks. Stress Corrosion Cracking (SCC)—the interplay of tensile stress and a corrosive setting can lead to cracking. Creep damage—creep may result in the initiation and development of cavities at grain boundaries, hastening the progress of fatigue cracks. Solidification cracking—during solidification, crack formation can occur due to shrinkage constraints in the mushy zone. Hot short tears—quick deformation during hot working can cause significant temperature increase, leading to hot short fractures.	[413,414]
	René 41, a superalloy based on nickel, is prone to strain-age cracking, particularly during the welding process and subsequent heat treatment. This cracking process mainly pertains to the microstructure of the alloy, especially the formation and shape of gamma prime (γ′) precipitates and carbides. Residual stresses caused by shrinkage during solidification and the development of γ′ during thermal processing further enhance this effect. Strain-age fractures—this kind of cracking happens when residual stresses, frequently caused by welding or heat treatment, interact with the aging process, resulting in the development of brittle phases at the boundaries of grains. Microstructural elements—the existence and structure of γ′ precipitates, along with carbides such as MC, M6C, and M_23_C_6_, are crucial factors. The dimensions, arrangement, and makeup of these precipitates can influence the alloy’s vulnerability to cracking. Effects of heat treatment—heat treatments before and after welding can affect the microstructure by dissolving or precipitating various phases, changing the alloy’s tendency to experience strain-age cracking. Residual stresses—elevated residual stresses, particularly those generated during welding, can worsen cracking by facilitating the development of cracks at grain boundaries. Conditions for processing—the selection of welding techniques (e.g., electron beam welding) and the parameters for post-weld heat treatment (e.g., rates of heating and cooling) can greatly influence the development of strain-age cracks. Mitigation strategies—improving heat treatment parameters, keeping carbon content low, and applying welding methods that lessen residual stresses can decrease René 41’s tendency for strain-age cracking.	[415,416]
	Inconel 725 failure mainly occurs due to intergranular cracking, especially in the presence of hydrogen. This cracking is frequently linked to the existence of precipitates at grain boundaries, particularly the F phase, which can weaken the cohesion of the grain boundaries and promote crack growth. Hydrogen, in effect, can additionally compromise these interfaces, resulting in expedited cracking in vulnerable conditions. Intergranular cracking—Inconel 725, similar to other nickel-based alloys, may undergo cracking at the grain boundaries (intergranular cracking). This kind of cracking is different from transgranular cracking, which spreads through the crystal structure. Grain Boundary Precipitates (F Phase)—the existence of particular intergranular phases, such as the F phase, can greatly influence the cracking resistance of Inconel 725. These precipitates may serve as stress concentrators or weaken the grain boundary cohesion, facilitating the initiation and propagation of cracks. The function of hydrogen—hydrogen, a prevalent element in various settings, can also enhance intergranular cracking in Inconel 725. Hydrogen atoms may gather at grain boundaries, especially at interfaces with the F phase, weakening the adhesion between the matrix and precipitates. This diminishing effect may result in early failure. Fracture onset and progression—although cracks usually begin at free surfaces, the existence of precipitates at grain boundaries and hydrogen can greatly affect the speed of crack growth. The F phase may serve as a location where a crack is most prone to initiate and expand. Mitigation strategies—grasping the function of the F phase and hydrogen in the cracking process enables the creation of approaches to enhance the crack resistance of Inconel 725. For instance, incorporating boron may aid in inhibiting the precipitation of the F phase, likely lessening hydrogen embrittlement. Additional Factors—although the emphasis is on hydrogen and grain boundary cracking, additional aspects such as stress levels, temperature, and the particular environment may also affect the overall cracking behavior of Inconel 725.	[417,418]
	In Inconel 706, cracks commonly start and extend along grain boundaries, particularly in regions with carbides and/or acicular η platelets. Fatigue crack growth can occur through both intergranular and transgranular modes, though their magnitudes differ. Elements such as heat treatment, formation of the η phase, and mechanisms of crack closure can affect the growth of cracks. Grain boundary fracturing—Inconel 706 is prone to cracking along grain boundaries, where stress concentrations may intensify. Clusters of carbides or η platelets at these interfaces can act as stress concentrators, aiding in crack formation and growth. Transgranular fracture—although intergranular cracking predominates, transgranular (through-grain) cracking may also happen, especially under specific heat treatment conditions. Effect of η Phase—the η phase, a secondary precipitate in Inconel 706, may exhibit a multifaceted influence. Although some research implies it may inhibit crack propagation, other findings show it can create straightforward routes for crack advancement. The η phase’s quantity and shape are influenced by the heat treatment, and its existence can change the fatigue crack growth resistance of the material. Effects of heat treatment—various heat treatments (e.g., solution treatment, stabilization annealing) can affect the microstructure and, as a result, the cracking tendency of Inconel 706. Stabilization heat treatments can occasionally lower fatigue crack propagation resistance by enhancing the amount of η phase. Crack closure—crack closure, occurring when the crack tip is compressed due to residual stresses or friction, can influence the effective stress intensity factor and, consequently, crack growth. Crack closure induced by roughness is considered a crucial element in Inconel 706. Impact of temperature—temperature can affect the growth rates of fatigue cracks in Inconel 706. Colder temperatures can decrease crack growth rates, and the influence of the R-ratio (the ratio of minimum to maximum stress) on crack propagation may also change with temperature.	[419,420]
	In Incoloy 909, cracks usually develop via a mechanism named liquation cracking, occurring in the heat-affected zone (HAZ) during welding, especially with elevated heat input. This cracking occurs due to low-melting eutectics and compositional segregation at the boundaries of grains. The process includes intergranular liquation, the beginning of cracks, and the expansion of cracks Intergranular liquation—while welding, the temperature variation in the HAZ results in the separation of elements, resulting in the creation of low-melting eutectics at the boundaries of grains. Fracture onset—these low-melting eutectics diminish the strength of the grain boundaries, rendering them prone to cracking when subjected to stress. Fracture expansion—cracks extend along the grain boundaries, which are weakened due to the presence of the liquid layer and segregation. Elements that affect liquation cracking in Incoloy 909 consist of: Heat introduction—increased heat input results in more noticeable segregation and creates broader liquid films, raising the likelihood of cracking. Microstructure—the dimensions and arrangement of grains can influence the chances of cracking. Welding settings—modifying the welding speed and laser power can assist in managing heat input and minimizing cracking. Pre-Welding thermal treatment—implementing a solid solution treatment in place of aging may decrease mechanical properties and can also aid in laser welding by reducing liquation cracking. Composition separation—the existence of low-melting phases such as Laves phases (Ni2Ti) at grain boundaries can worsen the cracking process. To reduce cracking, strategies consist of: Minimizing heat contribution—reducing laser power and welding speed can aid in decreasing segregation and preventing liquid film formation. Optimizing welding settings—meticulously managing welding variables is essential to avoid the development of significant tensile stresses and coarse microstructures. Pre-Welding preparation—solid solution treatment can reduce the material’s vulnerability to HAZ cracking. Surface readiness—eliminating oxides and guaranteeing adequate surface cleanliness prior to welding can avert porosity and enhance weld quality.	[421,422]
	The cracking mechanisms of Incoloy 945 are mainly linked to its vulnerability to hydrogen embrittlement and stress corrosion cracking (SCC) in certain conditions. The alloy’s elevated nickel levels offer protection against chloride-induced SCC, though it may still be influenced by different mechanisms Hydrogen embrittlement—Incoloy 945 may be prone to hydrogen embrittlement, especially in sour gas settings. Hydrogen atoms may penetrate the material, resulting in decreased ductility and heightened brittleness. This may lead to fractures, particularly when under pressure. Stress Corrosion Cracking (SCC) is a type of failure in materials that occurs due to the combined effects of tensile stress and a corrosive environment. This phenomenon can lead to sudden and catastrophic failures in structures, particularly in metals. Conditions such as temperature, humidity, and the presence of specific chemicals can significantly influence the susceptibility of a material to SCC. Identifying and mitigating SCC is essential for ensuring the durability and safety of engineering systems. Regular inspections and maintenance practices can help to minimize the risks associated with this type of cracking. Incoloy 945 typically shows strong resistance to chloride-induced SCC owing to its elevated nickel levels. Nonetheless, it remains vulnerable to other forms of SCC, including alkaline SCC or SCC in certain acidic conditions. Alkaline SCC is a process in which the protective layer on the material breaks, resulting in corrosion and the development of cracks when under stress. The precise mechanism of SCC may differ based on the particular environment and stress conditions. Elements affecting cracking include: Concentration of hydrogen—elevated hydrogen levels in the environment raise the likelihood of hydrogen embrittlement. Levels of stress—increased stress levels, particularly tensile stresses, can hasten the occurrence of cracking. Ecological circumstances—the existence of certain chemicals, including chloride ions, hydrogen sulfide, or various corrosive substances, can enhance SCC. Microstructure—the alloy’s microstructure, which comprises precipitates and grain boundaries, can affect the tendency to crack.	[423,424]
**Ni-aluminides**	Cracking in nickel aluminides (Ni-Al) can happen because of different mechanisms, mainly associated with their microstructure and processing conditions. These mechanisms consist of solidification cracking, ductility-dip cracking, liquation cracking, and oxidation-facilitated cracking. Grasping these mechanisms is essential for creating and producing Ni-Al parts with enhanced reliability and performance. Solidification fracturing is characterized by: Mechanism—takes place during the solidification phase, frequently in traditional casting or additive manufacturing (AM) techniques. It results from the unequal contraction of the material as it cools and the development of cracks at the boundaries of grains. Elements—high-angle grain boundaries (HAGBs) have a notable tendency to crack, especially when they contain higher concentrations of elements such as Mo, Ta, or Re. The rate of dendrite growth, local strain rate, and rate of liquid feed also affect the likelihood of cracking. Control—reducing the temperature gradient during solidification and fine-tuning the composition to limit the development of brittle phases can aid in preventing solidification cracking. Ductility-dip fracturing is characterized by: Mechanism—a high-temperature solid-state event that takes place especially in multi-pass welding or additive manufacturing processes. It pertains to the decrease in ductility at specific temperatures, resulting in fractures at grain boundaries. Internal aspects—the existence of low-melting-point phases and the level of residual stresses greatly affect ductility-dip cracking. Control—regulating the heat input during welding or additive manufacturing and enhancing the microstructure can minimize the vulnerability to this kind of cracking. Liquation Cracking is characterized by: Mechanism—results from the liquation of eutectic phases with low melting points or secondary precipitates at grain boundaries under residual stress. It is frequently seen in Ni-Al alloys that are laser-welded. Elements—the existence of low-melting-point phases, the intensity of heat cycling during welding, and residual stresses all play a role in liquation cracking. Control—adjusting the alloy mixture to prevent low-melting-point phase formation and managing heat input in welding can minimize liquation cracking. Oxidation-enhanced fracture is characterized by: Mechanism—oxidation occurring on the surface or inside the material may result in the development of brittle oxide layers or the weakening of grain boundaries, promoting the initiation and growth of cracks. Elements—elevated temperatures and the availability of oxygen in the surroundings can hasten oxidation-induced cracking. Control—enhancing the oxidation resistance of Ni-Al through the addition of elements such as chromium or the application of protective coatings can reduce this kind of cracking. Extra considerations relate to: Residual stresses—elevated residual stresses, especially in additive manufacturing processes, can greatly influence the initiation and growth of cracks. Microstructure—the dimensions, form, and arrangement of grains and precipitates may affect crack behavior. Processing settings—selecting the laser power, scan speed, and various parameters in additive manufacturing may influence the microstructure and the likelihood of cracking.	[387,425,426,427,428,429,430,431,432,433,434,435,436,437,438,439,440,441,442,443,444,445]
**Oxygen Dispersion Strengthened (ODS) Ni alloys**	Cracking in Oxygen Dispersion Strengthened (ODS) Ni alloys may arise from various mechanisms, such as solidification cracks, liquation cracking, and ductility-dip cracking. Solidification cracks, referred to as hot cracking, may occur at steep grain boundaries during solidification because of the liquid film. Liquation cracking, commonly observed in laser welding and additive manufacturing, entails the creation of small fractures in the heat-affected area resulting from repeated thermal cycling and the emergence of low-melting-point phases. Ductility-dip cracking, a solid-state occurrence, takes place at elevated temperatures and is linked to a decrease in material plasticity in the heat-affected region. Solidification cracking—this type of cracking occurs during the last phase of solidification when liquid films are confined at high-angle grain boundaries. These liquid layers may cause stress concentrations and initiate cracks at the edges. Liquation cracking—this fracture process is frequently seen in laser welding and additive manufacturing techniques. It results from repeated heat cycling, which may cause the precipitation of phases with low melting points or the occurrence of constitutional liquation. Ductility-dip cracking—this fracturing takes place at elevated temperatures and is a solid-state occurrence. It is associated with a decrease in material ductility in the heat-affected zone, which can be caused by elements such as residual stresses or alterations in microstructure. Oxide-associated fracturing—in certain instances, the existence of oxides, whether within grain interiors or at grain boundaries, may aid in crack development. These oxides may serve as stress concentrators and enhance crack formation, especially during reheating procedures. Crack prevention—different methods can be utilized to reduce cracking in ODS Ni alloys, such as grain refinement, the incorporation of specific high-angle grain boundaries, and the application of passivation techniques. For instance, incorporating La_2_O_3_ in the alloy can enhance grain refinement and diminish susceptibility to cracking. ODS improvement—the existence of oxide dispersion in ODS alloys may also contribute to crack development. Although oxides can enhance strength and elevate high-temperature characteristics, an overabundance or uneven distribution of oxides may lead to stress points that encourage cracking.	[427,446,447,448,449,450,451,452]
**Ni alloys used in Petroleum Industry**	Specimens of Alloy 945, pre-stressed to 100% of the yield stress and exposed to the NACE TM0177 method C solution (20% NaCl + 508 psi CO_2_ + 508 psia H_2_S) at 175 °C, exhibited no cracking after 90 days of testing. Likewise, Alloy 945 tensile samples subjected to 90% of the actual yield stress and galvanically connected to steel that underwent TM0177 method A acidified solution at 24 °C showed no signs of cracking after 30 days of testing. Alloy C-22HS, when age-hardened, exhibits outstanding resistance to environmentally induced cracking under simulated oil well conditions. The age-hardened Cu-Ni K-500 is utilized in the oil and gas sector, but its applications are restricted to less severe conditions compared to the superior alloys like C-276 and 718. The environmentally assisted cracking of materials during the production of petroleum products can be divided into three types: 1. The widely recognized elevated temperature stress corrosion cracking caused by chlorides, which restricts the use of austenitic stainless steels, 2. Sulfide stress corrosion primarily impacts martensitic materials, 3. Hydrogen embrittlement is commonly linked to cathodic charging when Ni alloys are coupled with carbon steel parts. Most, if not all, environmental cracks in Ni alloys are probably linked to hydrogen entering the alloys during their use.	[382]
**Ni alloys used in** **nuclear application**	The primary mechanism of degradation or failure of Ni alloys in nuclear power plants is stress corrosion cracking. The reduction in cracking in steam generator tubing can be achieved through modifications in water chemistry, design, manufacturing, and tubing alloy materials (e.g., utilizing Alloy 690 rather than Alloy 600). Failures related to SCC in other components were also documented, such as the cracking of Alloy 600 reactor vessel head (RVH) penetrations, Alloy X-750 bolts and springs, as well as Alloys 82 and 182 welds. The susceptibility of Alloy 600 to SCC in the nuclear power sector is influenced by the alloy’s composition and microstructure, along with external factors including the system’s redox potential, tensile stress levels, temperature, electrolyte pH, presence of harmful dissolved species like lead, and hydrogen partial pressure. Seven main factors influence the extent of SCC penetration: the impact of alloy composition and structure, the influence of pH, the role of environmental species like lead, sulfate, etc., the effect of stress, the influence of electrochemical potential, the impact of temperature, and the effect of time. These variables are interconnected, and if any one of them changes, the influence of all the other variables on SCC also changes. The susceptibility of Alloy 600 to SCC in high-temperature water is influenced by its thermomechanical history, as well as the quantity, shape, and distribution of carbon within the matrix. Alloy 600 mill annealed (600MA) is the condition most vulnerable to SCC in the service conditions of PWR steam generator tubing. Alloy 600MA, typically annealed at temperatures under 950 °C, contains the majority of carbide located in intragranular (or transgranular) positions. Alloy600 thermally treated (600TT), containing a minimum of 0.02% carbon and subjected to annealing temperatures over 1000 °C, then heated at 700 °C to precipitate the majority of carbides in an intergranular state, exhibits strong resistance to SCC in high-temperature water. Some plants address Alloy 600 SCC by utilizing Alloy 690TT instead of the more SCC-resistant Alloy 600TT. Alloy 690 is more resistant to SCC than Alloy 600 because of its higher Cr content. Certain plants favor Alloy800 over 600TT or 690TT. Alloys with a moderate Ni content (such as Alloy 800 with 33% Ni) exhibit greater resistance to cracking in typical high-temperature pure water conditions found in nuclear power compared to alloys with high Ni content. Alloy 800 is resistant to both cracking in solutions containing chloride and in water at high temperatures. The occurrence of cold work significantly enhances Alloy600’s vulnerability to SCC regarding both crack initiation and growth. The crack growth rate of SCC in Alloy 82 weld metal rose when the material was subjected to 12% cold working. Alloy 690 (29% Cr) exhibits greater resistance to SCC in elevated temperature water compared to Alloy 600 (16% Cr). Alloy 690 and its weld metals (Alloy 52/152) exhibit a SCC crack propagation rate that is about 100 to 400 times less than that of Alloys 600 and 182 tested under comparable conditions in simulated primary water at 340–360 °C. Increased Cr content in alloy 690 results in reduced resistance to hydrogen embrittlement compared to Alloy 600. C-22 showed remarkable resistance to EAC across various solutions. In tests performed with cyclic loading, constant load, constant deformation, and slow strain rate conditions in solutions of 14 molal MgCl2, as well as simulated concentrated groundwaters with pH values from 3 to 13, samples of C-22, C-4, G-3, 825, and 625 alloys were analyzed. Gas tungsten arc welded (GTAW) and non-welded specimens were subjected for over 5 years to the corrosion potential of the vapor and liquid phases of three distinct solutions (pH 2.8–10), replicating up to 1000 times the groundwater concentration at both 60 °C and 90 °C. No signs of environmentally induced cracking were observed in any of these alloys. Alloy C-22 exhibited susceptibility to EAC during SSRT on mill-annealed samples in hot simulated concentrated water (SCW) under anodic applied potentials. SCW is an alkaline solution with multiple ions, roughly 1000 times more concentrated than groundwater. It was first thought that the minor concentration of fluoride ions in this solution (1400 ppm) was responsible for the cracking of C-22. The primary cause of the SCC of Alloy 22 is the bicarbonate found in the SCW solution. The vulnerability to cracking of C-22 was significantly influenced by the applied potential and the solution’s temperature. The greatest vulnerability to EAC was observed at approximately 90 °C at +400 mV in the saturated silver chloride (SSC) electrode layer. At the corrosion potential, C-22 showed no signs of EAC even at 90 °C. Likewise, under anodic applied potentials, C-22 remained free from EAC at room temperatures, but as the temperature rose, the duration until failure in the tests shortened. The presence of an anodic peak in the polarization curve of the alloy in SCW environments was associated with the occurrence of EAC. At room temperature, the peak is absent, and EAC does not occur. The most aggressive species for EAC in SCW was bicarbonate, yet the presence of chloride in the bicarbonate solution increases the environment’s aggressiveness. Alloy C-22 can experience embrittlement when it is subjected to slow strain with cathodic applied potentials (or currents). The highest vulnerability to cracking under cathodic conditions appeared to happen at room temperatures, indicating a failure mechanism associated with hydrogen.	[382]

**Table 36:** Fillers for welding.

Ni Alloy Group	Alloy Designation	Pair Component	Electrode	Facilitated Filler	Flux	Refs.
CP Ni	200	-	-	ERNi-1	-	[2]
	201	-	-	ERNi-1	-	[2]
	Dissimilar Ni/Steel	-	-	ERNi-1	-	[2]
SSS Ni alloys	400	-	-	ERNiCu-7	-	[2]
	405	-	-	ERNiCu-7	-	[2]
	K-500	-	-	ERNiCu-7	-	[2]
	B2	-	-	ERNiMo-7	-	[22]
	B3	-	-	ERNiMo-7	-	[23]
	Invar K93600	-	-	2.4648 (Inconel 600)	-	[20]
	Inconel 600	Inconel 600	Inconel Welding Electrode 182	Inconel Filler Metal 82;	Incoflux 4 Submerged Arc Flux—for submerged welding	[34]
	Inconel 601	Inconel 601	ENiCrFe-3, ENiCrMo-3	ERNiCr-3, ERNiCrMo-3	-	[35]
		stainless steel	ENiCrFe-2	ERNiCr-3	-	[35]
		low-alloy steel	ENiCrFe-2	ERNiCr-3	-	[35]
		5-9% Ni steel:	ENiCrFe-2	ERNiCr-3	-	[35]
		Cu	ENi-1	ERNi-1	-	[35]
		Ni-Cu	ENi-1	ERNi-1	-	[35]
	Inconel 625	Inconel 625	ENiCrMo-10	ERNiCrMo-3	Incoflux NT100 Submerged Arc Flux	[36,72]
		Stainless steel	ENiCrMo3	ERNiCrMo-3	Incoflux NT100 Submerged Arc Flux	[33]
	Inconel 718	-	ERNiFeCr-2	ERNiFeCr-2, ERNiCr-3	-	[37]
	Inconel 800	-		ERNiCr-3	-	[38]
	Incoloy 800H, Incoloy 800HT	-	ENiCrCoMo-1	ERNiCr-3, ERNiCrCoMo-1	-	[38]
	G3	-	Inconel G-3 for shielded metal arc welding ENiCrMo-3	Inconel G-3 for gas-shielded arc welding ERNiCrMo-3	-	[33]
	Incoloy 825	-	ENiCrMo3	ERNiCrMo-3	-	[43]
	Incoloy 925	-	725NDUR	ERNiCrMo-17, 725NDUR	-	[44]
	Incoloy 926	-	ENiCrMo-10	ERNiCrMo-10	-	[459]
	Hastelloy C-276	-	ENiCrMo3	ERNiCrMo-4 to -10	-	[48]
	Hatelloy C-4	-	ENiCrMo-7	ERNiCrMo-7	-	[49]
	Inconel HX	-		2.4665 (INCONEL HX)	-	[20]
	Hastelloy C-22	-	ENiCrMo-10	ER NiCrMo-10	-	[461]
	Haynes 59	-		ERNiCrMo-13	-	[55]
	Hastelloy C2000	-	ENiCrMo-17	ERNiCrMo-17	-	[56]
	Inconel 686	-	ENiCrMo-3	ERNiCrMo-14, ERNiCrMo-4 through -10	-	[57]
	Inconel 617	-	ENiCrCoMo-1	ERNiCrCoMo-1	-	[58]
PS Ni alloys	K500	-	-	ERNiCu-7, ERNiFeCR-2	-	[61]
	80A	-	-	ERNiCr-3	-	[497]
	90	-	-	AMS5829	-	[469]
	C-263	-	-	NiCo20Cr20MoTi	-	[63]
	Haynes 282	-	-	AWS A 5.14: ERNiCrCoMo-2 mod	-	[498]
	Waspaloy	-	-	AMS 5828, UNS N07001	-	[474]
	Rene 41	-	-	Rene 41 (Filler alloy)	-	[475]
	Inconel 725	-	-	ERNiCrMo-17/SG-NiCr23Mo16Cu (Filler alloy)	-	[68]
	Inconel 706	-	-	Inconel Filler Metal 718	-	[69]
	Incoloy 945	-	-	725NDUR^®^	-	[71]

**Table 37:** Filler metals for GMA/GTA welding of Ni-based alloys [5].

Filler Metal	Primary Applications
ERNi-1	Nickel 200 and Nickel 201; dissimilar combinations of Ni alloys and steels; surfacing of steels
ERNiCu-7	Alloys 400, R-405, and K-500; surfacing of steel
ERCuNi	Alloy 450; weldable grades of 70/30, 80/20, and 90/10 Cu-Ni alloys
ERNiCr-3, ERNiCrFe-7	Alloy 600, alloy 690; dissimilar combination to steels; surfacing of steels
ERNiCr-3	Alloys 600 and 601; alloys 800 and 800HT; alloy 330; dissimilar combinations of steels and Ni alloys; surfacing of steels
ERNiCr-3, ERNiCrMo-3	Dissimilar combinations of steel and Ni alloys
ERNiCrCoMo-1	Alloy 617; alloy 800HT; dissimilar combinations of high-temperature alloys
ERNiCrMo-3	Alloys 625 and 601; pit-resistant alloys; dissimilar combinations of steels and Ni alloys; surfacing of steels
ERNiFeCr-2	Alloys 718 and X-750
ERNiCrMo-3	Alloy 825
ERNiCrMo-4 through 10	Alloy 686, alloy 622, alloy C-276; other pit-resistant alloys; surfacing of steels

## Data Availability

No new data were created or analyzed in this study.

## Abbreviations

The following abbreviations are used in this manuscript:

CP	Commercial Pure
FSW	Friction Stir Welding
PS	Precipitation Strengthened
SA	Specialty Alloy
SSS	Solid-Solution Strengthened

## References

[1] Mudd G.M., Jowitt S.M. (2014). A Detailed Assessment of Global Nickel Resource Trends and Endowments. Econ. Geol..

[2] Davis J.R., ASM International (2000). ASM Specialty Handbook: Nickel, Cobalt, and Their Alloys.

[3] Lippold J.C. (2009). Welding Metallurgy and Weldability of Nickel-Base Alloys.

[4] Caron J.L., Sowards J.W. (2014). Weldability of Nickel-Base Alloys. Comprehensive Materials Processing.

[5] Kojundžić D., Krnić N., Samardžić I. Weldability of Nickel and Nickel-Based Alloys. Proceedings of the International Conference on Materials Corrosion, Heat Treatment, Testing and Tribology, MTECH 2021.

[6] Isabellenhütte Heusler GmbH & Co. KG. (2020). Pure Nickel—Data Sheet 2020.

[7] Nickel Institute Properties of Nickel 2025. https://nickelinstitute.org/en/nickel-applications/properties-of-nickel/.

[8] (2025). Lenntech Chemical Elements Listed by Ionization Energy. https://www.lenntech.com/periodic-chart-elements/ionization-energy.htm.

[9] (2010). Standard Welding Terms and Definitions.

[10] AZOMaterials (2025). Nickel-Properties, Fabrication and Applications of Commercially Pure Nickel.

[11] Joseph Sahaya Anand T. (2012). Nickel as an Alternative Automotive Body Materials. J. Mech. Eng. Sci..

[12] Kopeliovich D. (2023). Commercially Pure Nickel Alloys. https://www.substech.com/dokuwiki/doku.php?id=commercially_pure_nickel_alloys.

[13] (2025). Virgamet Alloy 200, Alloy 201, 2.4066, 2.4068, Nickel 200, Nickel 201—Commercially Pure Nickel. https://virgamet.com/offer/alloy-200-201-2-4066-2-4068-nickel-200-201-n02200-n02201.

[14] Yonezawa T., Konings R.J.M. (2012). 2.08—Nickel Alloys: Properties and Characteristics. Comprehensive Nuclear Materials.

[15] Geddes B., Leon H., Huang X. (2010). Superalloys: Alloying and Performance.

[16] Reed R.C. (2006). The Superalloys: Fundamentals and Applications.

[17] (2025). Virgamet Alloy 400, 2.4360, UNS N04400, Monel^®^ Alloy 400—Nickel Alloy. https://virgamet.com/offer/alloy-monel-400-2-4360-n04400-bs-na13.

[18] (2025). Virgamet Monel K500, Alloy K500, 2.4375, UNS N05500—Nickel Alloy. https://virgamet.com/offer/alloy-monel-k500-2-4375-n05500-na18.

[19] Haußmann L., Ur Rehman H.U., Matschkal D., Göken M., Neumeier S. (2021). Solid Solution Strengthening of Mo, Re, Ta and W in Ni during High-Temperature Creep. Metals.

[20] (2025). MetalCor Ni Alloys. https://www.metalcor.de/en/datenblatt/.

[21] (2025). CorrosionMaterials Corrosion Resistant Alloys. https://corrosionmaterials.com/alloys/.

[22] (2025). Virgamet Alloy B2, 2.4617, UNS N10665, Hastelloy^®^ B2-Ni-Mo Alloy. https://virgamet.com/offer/alloy-hastelloy-b2-2-4617-uns-n10665-nimo28-nimo28fe5.

[23] (2025). Virgamet Alloy B3, 2.4600, UNS N10675, Hastelloy^®^ B3—Nickel Alloy. https://virgamet.com/offer/hastelloy-alloy-b3-2-4600-uns-n10675-nimo30cr-ni1067.

[24] (2014). AZOMaterials Nickel-Molybdenum (NiMo) Master Alloy. https://www.azom.com/article.aspx?ArticleID=10865.

[25] Mehta K.K., Mukhopadhyay P., Mandal R.K., Singh A.K. (2015). Microstructure, Texture, and Orientation-Dependent Flow Behavior of Binary Ni-16Cr and Ni-16Mo Solid Solution Alloys. Met. Mater. Trans. A.

[26] Tawancy H.M. (2017). On the Precipitation of Intermetallic Compounds in Selected Solid-Solution-Strengthened Ni-Base Alloys and Their Effects on Mechanical Properties. Metallogr. Microstruct. Anal..

[27] Yang C., Muránsky O., Zhu H., Thorogood G.J., Huang H., Zhou X. (2017). On the Origin of Strengthening Mechanisms in Ni-Mo Alloys Prepared via Powder Metallurgy. Mater. Des..

[28] (2025). Kanthal Nickel-Iron (NiFe) Alloys. https://www.kanthal.com/en/knowledge-hub/heating-material-knowledge/resistance-alloys-for-lower-temperature-applications2/nickel-iron-alloys/.

[29] (2025). Nickel Institute Nickel Alloys. https://nickelinstitute.org/en/nickel-applications/nickel-alloys/.

[30] Sun F. (2022). Achieving High Tensile Strength of Heat-Resistant Ni-Fe-Based Alloy by Controlling Microstructure Stability for Power Plant Application. Crystals.

[31] (2022). VDM Metals International GmbH VDM Alloy 36 Pernifer 36. https://www.vdm-metals.com/fileadmin/user_upload/Downloads/Data_Sheets/Data_Sheet_VDM_Alloy_36.pdf.

[32] Johnson A. (1996). Ni-Cr-Fe. https://sv.rkriz.net/classes/MSE2094_NoteBook/96ClassProj/examples/nicrfe.html.

[33] SpecialMetals (2025). INCONEL^®^ Welding Products. https://www.specialmetals.com/divisions/welding-products/tradenames/inconel.

[34] Virgamet Inconel (2025). 600, Alloy 600, Material 2.4816, UNS N06600. https://virgamet.com/offer/inconel-600-alloy-600-material-2-4816-uns-n06600.

[35] (2025). Virgamet Alloy 601, 2.4851, UNS N06601, Inconel^®^ 601—Nickel Alloy According to ASTM B166, DIN 17754. https://virgamet.com/offer/inconel-alloy-601-24851-uns-n06601-nicr23fe-na49-ncf601.

[36] Virgamet Alloy (2025). 625, UNS N06625, 2.4856—Nickel Alloy. https://virgamet.com/offer/nickel-alloy-inconel-625-n06625-2-4856-bs3075-na21.

[37] Virgamet Alloy (2025). 718, 2.4668, UNS N07718, Inconel^®^ 718—Nickel Alloy. https://virgamet.com/offer/alloy-inconel-718-2-4668-n07718-bs2901-na51-ncf718-chn55mbju.

[38] Virgamet Alloy (2025). 800, Alloy 800H, Alloy 800HT—Nickel Alloy. https://virgamet.com/offer/alloy-incoloy-800-800h-800ht-n08800-n08810-n08811-1-4876-1-4958-1-4959.

[39] Woite M. (2025). GmbH Material No.: Alloy G3. https://woite-edelstahl.com/alloyg3en.html.

[40] Ashby M.F. (2013). Material Profiles. Materials and the Environment.

[41] Symons D.M. (1997). Hydrogen Embrittlement of Ni-Cr-Fe Alloys. Met. Mater. Trans. A.

[42] AZOMAterials (2012). Super Alloy HASTELLOY(r) G-3 Alloy (UNS N06985). https://www.azom.com/article.aspx?ArticleID=7733#:~:text=Super%20alloys%20or%20high%20performance%20alloys%20have,with%20deformation%20resistance%20and%20high%20surface%20stability.&text=Annealing%20of%20HASTELLOY(r)%20G%2D3%20alloy%20can%20be,rapid%20cooling%20of%20air%20and%20water%20quenching.

[43] Virgamet Alloy (2025). 825, 2.4858, UNS N08825, Incoloy 825—Nickel Alloy. https://virgamet.com/offer/alloy-incoloy-825-2-4858-uns-n08825-nicr21mo-na16.

[44] Virgamet ALLOY (2025). 925, UNS N09925, INCOLOY^®^ 925—STOP NIKLU. https://virgamet.pl/oferta/alloy-incoloy-925-n09925-h09925-ns2401.

[45] NineSteel (2025). Incoloy 926. https://www.ninesteel-ss.com/products/incoloy-926.

[46] Virgamet Alloy (2025). N86, Nimonic^®^ Alloy 86—Nickel Alloy. https://virgamet.com/offer/nickel-alloy-n86-nimonic-86.

[47] Virgamet Alloy (2025). 90, UNS N07090, 2.4632, NiCr20Co18Ti—Nickel Alloy. https://virgamet.com/offer/nimonic-alloy-90-n07090-2-4632-nicr20co18ti-nickel-alloy.

[48] Virgamet Alloy (2025). C-276, UNS N10276, 2.4819—Otherwise Hastelloy C-276, CrNiMo16-60-16, NiMo16Cr15Fe6W4 According to ASTM B462 and ISO 9722. https://virgamet.com/offer/hastelloy-alloy-c276-2-4819-n10276-crnimo156016-nimo16cr15fe6w422.

[49] (2025). Virgamet Alloy C4, UNS N06455, 2.4610, Hastelloy^®^ C-4—Nickel Alloy According to DIN 17744:2019 i ASME SB366-21. https://virgamet.com/offer/alloy-hastelloy-c4-n06455-2-4610-nimo16cr16ti-nimo16cr17ti.

[50] (2025). Aeether Incoloy 926 Alloy. https://www.aeether.com/AEETHER/grades/926.html.

[51] Virgamet Stainless Steel (2025). 1.4529, X1NiCrMoCuN25-20-7, Alloy 926, UNS N08926. https://virgamet.com/offer/x1nicrmocun25207-1-4529-alloy-926-6mo-uns-n08926-stainless-steel.

[52] VDM (2020). Metals International GmbH VDM Alloy 825 Nicofer 4221. https://www.vdm-metals.com/fileadmin/user_upload/Downloads/Data_Sheets/Data_Sheet_VDM_Alloy_825.pdf.

[53] AZOMaterials (2012). Incoloy 825–Properties, Applications, Fabrication, Machinability and Weldability of Incoloy 825. https://www.azom.com/article.aspx?ArticleID=4245.

[54] Virgamet Alloy (2025). C22, UNS N06022, 2.4602, Hastelloy^®^ C-22—Nickel Alloy According to ASTM B 574-18 and DIN 17750:2021. https://virgamet.com/offer/alloy-hastelloy-c22-uns-n06022-nicr21mo14w-2-4602-nw6022.

[55] Virgamet Alloy (2025). 59, 2.4605, UNS N06059, Haynes^®^ 59, NiCr23Mo16Al—Nickel Alloy According to ASTM B 366 and Other Standards. https://virgamet.com/offer/alloy-59-uns-n06059-2-4605-nicr23mo16al-haynes-59.

[56] Virgamet Alloy (2025). 2000, 2.4675, UNS N06200, Hastelloy^®^ C-2000—Nickel Alloy. https://virgamet.com/offer/alloy-2000-2-4675-n06200-hastelloy-c2000-nicr23mo16cu.

[57] Virgamet Alloy (2025). 686, 2.4606, UNS N06686—NICKEL ALLOY. https://virgamet.com/offer/inconel-nickel-alloy-686-n06686-2-4606.

[58] Virgamet Alloy (2025). 617, 2.4663, UNS N06617, Inconel^®^ 617—Alloy. https://virgamet.com/offer/alloy-inconel-haynes-617-na50-24663-nicr23co12mo-n06617.

[59] Dong C., Chen Z., Zhao Y., Zhou Y., Wu Y., Wang Z. (2023). Microstructure Characterization and Strengthening Behavior of a Non-γ’ Phase Nickel-Based Alloy C-HRA-2 after Creep at 650 °C. Mater. Today Commun..

[60] Chen K., He X., Liu Z., Li G., Bao H. (2024). Solidification Path and Precipitation Mechanism of a Ni-Cr-Co-Mo Based Heat-Resistant Alloy. Mater. Charact..

[61] (2025). CorrosionMaterials Alloy K-500. https://corrosionmaterials.com/alloys/alloy-k-500/.

[62] (2025). Virgamet Alloy 80A, 2.4952, UNS N07080, Nimonic^®^ 80A—Nickel Alloy. https://virgamet.com/offer/alloy-nimonic-80a-uns-n07080-na20-2-4952-nicr20tial.

[63] (2025). Virgamet Alloy C-263, 2.4650, Nimonic^®^ Alloy 263, Haynes^®^ 263—Nickel Alloy. https://virgamet.com/offer/alloy-c-263-2-4650-nicr20co18ti-n07263-haynes-nimonic-263.

[64] (2025). AlloyWire Haynes 282. https://www.alloywire.com/alloys/haynes-282/.

[65] Łyczkowska K., Adamiec J., Jachym R., Kwieciński K. (2017). Properties of the Inconel 713 Alloy Within the High Temperature Brittleness Range. Arch. Foundry Eng..

[66] (2025). Virgamet Waspalloy, 2.4654, Haynes^®^ Waspaloy—Nickel Alloy. https://virgamet.com/offer/waspalloy-haynes-waspaloy-n07001-2-4654-nicr20co13mo4ti3al.

[67] (2025). Virgamet René 41, Alloy 41, 2.4973, UNS N07041—Nickel Alloy According to AMS 5399, 5545, 5800, 7469D. https://virgamet.com/offer/rene-alloy-41-n07041-2-4973-nicr19como.

[68] (2025). Virgamet Alloy 725, UNS N07725, Inconel^®^ 725—Nickel Alloy. https://virgamet.com/offer/alloy-725-n07725-inconel-725.

[69] (2004). Special Metals Inconel Alloy 706. http://specialmetals.ir/images/technical_info/nickel-base-alloy/inconel-alloy-706.pdf.

[70] (2004). Special Metals Incoloy Alloy 909. https://www.specialmetals.com/documents/technical-bulletins/incoloy/incoloy-alloy-909.pdf.

[71] (2025). Special Metals Incoloy Alloy 945. https://www.specialmetals.com/documents/technical-bulletins/incoloy/incoloy-alloy-945.pdf.

[72] (2025). Hursan What Is Inconel 713? Properties, Applications, and Advantages. https://hursan-com.translate.goog/en/what-is-inconel-713-properties-applications-and-advantages/?_x_tr_sl=en&_x_tr_tl=pl&_x_tr_hl=pl&_x_tr_pto=rq.

[73] Zhang F., He J., Wu Y., Mao H., Wang H., Liu X., Jiang S., Nieh T.G., Lu Z. (2022). Effects of Ni and Al on Precipitation Behavior and Mechanical Properties of Precipitation-Hardened CoCrFeNi High-Entropy Alloys. Mater. Sci. Eng. A.

[74] Zhao Y., Cao S., Zeng L., Xia M., Jakse N., Li J. (2023). Intermetallics in Ni–Al Binary Alloys: Liquid Structural Origin. Met. Mater. Trans. A.

[75] Jozwik P., Polkowski W., Bojar Z. (2015). Applications of Ni_3_Al Based Intermetallic Alloys—Current Stage and Potential Perceptivities. Materials.

[76] Meng Y., Li J., Zhang S., Gao M., Gong M., Chen H. (2023). Wire Arc Additive Manufacturing of Ni-Al Intermetallic Compounds through Synchronous Wire-Powder Feeding. J. Alloys Compd..

[77] Asirvatham M.C., Masters I., West G., Harris C. (2025). Influence of Nickel-Plating on Laser Weldability of Aluminium Busbars for Lithium-Ion Battery Interconnects. J. Mater. Res. Technol..

[78] Li J., Wang Y., Huang X., Zhang C., Ren J., Lu X., Tang F., Xue H. (2021). Tensile Mechanical Performance of Al/Ni Dissimilar Metals Bonded by Self-Propagating Exothermic Reaction Based on Molecular Dynamics Simulation. Mater. Today Commun..

[79] Liu C.T., White C.L., Horton J.A. (1985). Effect of Boron on Grain-Boundaries in Ni_3_Al. Acta Metall..

[80] Crawford G. (2003). Nickel Magazine. https://www.academia.edu/107987847/Surface_Engineering_of_Corrosion_Environmental_Fracture_Cavitation_and_Impingement_Resistant_Materials.

[81] Hadi M., Kamali A.R. (2009). Investigation on Hot Workability and Mechanical Properties of Modified IC-221M Alloy. J. Alloys Compd..

[82] Walter J.L., Cline H.E. (1970). The Effect of Solidification Rate on Structure and High-Temperature Strength of the Eutectic NiAl-Cr. Met. Trans..

[83] Cline H.E., Walter J.L. (1970). The Effect of Alloy Additions on the Rod-Plate Transition in the Eutectic NiAl−Cr. Met. Trans..

[84] Cline H.E., Walter J.L., Lifshin E., Russell R.R. (1971). Structures, Faults, and the Rod-Plate Transition in Eutectics. Met. Trans..

[85] Johnson D.R., Chen X.F., Oliver B.F., Noebe R.D., Whittenberger J.D. (1995). Processing and Mechanical Properties of In-Situ Composites from the NiAlCr and the NiAl(Cr,Mo) Eutectic Systems. Intermetallics.

[86] Rahaei M.B., Jia D. (2014). Processing Behavior of Nanocrystalline NiAl during Milling, Sintering and Mechanical Loading and Interpretation of Its Intergranular Fracture. Eng. Fract. Mech..

[87] Yang J.-M., Jeng S.M., Bain K., Amato R.A. (1997). Microstructure and Mechanical Behavior of In-Situ Directional Solidified NiAl/Cr(Mo) Eutectic Composite. Acta Mater..

[88] Whittenberger J.D., Raj S.V., Locci I.E., Salem J.A. (2002). Elevated Temperature Strength and Room-Temperature Toughness of Directionally Solidified Ni-33Al-33Cr-1Mo. Met. Mater. Trans. A.

[89] Cui C.Y., Chen Y.X., Guo J.T., Li D.X., Ye H.Q. (2000). Preliminary Investigation of Directionally Solidified NiAl–28Cr–5.5Mo–0.5Hf Composite. Mater. Lett..

[90] Cui C.Y., Guo J.T., Ye H.Q. (2008). Effects of Hf Additions on High-Temperature Mechanical Properties of a Directionally Solidified NiAl/Cr(Mo) Eutectic Alloy. J. Alloys Compd..

[91] Cui C.Y., Guo J.T., Qi Y.H., Ye H.Q. (2002). Deformation Behavior and Microstructure of DS NiAl/Cr(Mo)Alloy Containing Hf. Intermetallics.

[92] Guo J.T., Cui C.Y., Chen Y.X., Li D.X., Ye H.Q. (2001). Microstructure, Interface and Mechanical Property of the DS NiAl/Cr(Mo,Hf) Composite. Intermetallics.

[93] Guo J.T., Cui C.Y., Qi Y.H., Ye H.Q. (2002). Microstructure and Elevated Temperature Mechanical Behavior of Cast NiAl–Cr(Mo) Alloyed with Hf. J. Alloys Compd..

[94] Sheng L.Y., Guo J.T., Zhou L.Z., Ye H.Q. (2009). The Effect of Strong Magnetic Field Treatment on Microstructure and Room Temperature Compressive Properties of NiAl–Cr(Mo)–Hf Eutectic Alloy. Mater. Sci. Eng. A.

[95] Guo J.T., Sheng L.Y., Tian Y.X., Zhou L.Z., Ye H.Q. (2008). Effect of Ho on the Microstructure and Compressive Properties of NiAl-Based Eutectic Alloy. Mater. Lett..

[96] Guo J.T., Huai K.W., Gao Q., Ren W.L., Li G.S. (2007). Effects of Rare Earth Elements on the Microstructure and Mechanical Properties of NiAl-Based Eutectic Alloy. Intermetallics.

[97] Liang Y., Guo J., Xie Y., Zhou L., Hu Z. (2009). High Temperature Compressive Properties and Room Temperature Fracture Toughness of Directionally Solidified NiAl-Based Eutectic Alloy. Mater. Des..

[98] Shang Z., Shen J., Zhang J., Wang L., Fu H. (2012). Effect of Withdrawal Rate on the Microstructure of Directionally Solidified NiAl–Cr(Mo) Hypereutectic Alloy. Intermetallics.

[99] Shang Z., Shen J., Zhang J., Wang L., Wang L., Fu H. (2014). Effect of Microstructures on the Room Temperature Fracture Toughness of NiAl–32Cr–6Mo Hypereutectic Alloy Directionally Solidified at Different Withdrawal Rates. Mater. Sci. Eng. A.

[100] Milenkovic S., Schneider A., Frommeyer G. (2011). Constitutional and Microstructural Investigation of the Pseudobinary NiAl–W System. Intermetallics.

[101] Barclay R.S., Kerr H.W., Niessen P. (1971). Off-Eutectic Composite Solidification and Properties in Al-Ni and Al-Co Alloys. J. Mater. Sci..

[102] Shang Z., Shen J., Wang L., Du Y., Xiong Y., Fu H. (2015). Investigations on the Microstructure and Room Temperature Fracture Toughness of Directionally Solidified NiAl–Cr(Mo) Eutectic Alloy. Intermetallics.

[103] Azarmi F. (2011). Creep Properties of Nickel Aluminide Composite Materials Reinforced with SiC Particulates. Compos. Part. B Eng..

[104] Jha S.C., Ray R., Gaydosh D.J. (1989). Dispersoids in Rapidly Solidified B2 Nickel Aluminides. Scr. Metall..

[105] Jha S.C., Ray R., Whittenberger J.D. (1989). Carbide-Dispersion-Strengthened B2 NiAl. Mater. Sci. Eng. A.

[106] Zhou L.Z., Guo J.T., Fan G.J. (1998). Synthesis of NiAl–TiC Nanocomposite by Mechanical Alloying Elemental Powders. Mater. Sci. Eng. A.

[107] Krivoroutchko K., Kulik T., Matyja H., Portnoy V.K., Fadeeva V.I. (2000). Solid State Reactions in Ni–Al–Ti–C System by Mechanical Alloying. J. Alloys Compd..

[108] Whittenberger J.D., Grahle P., Behr R., Arzt E., Hebsur M.G. (2000). Elevated Temperature Compressive Strength Properties of Oxide Dispersion Strengthened NiAl after Cryomilling and Roasting in Nitrogen. Mater. Sci. Eng. A.

[109] Albiter A., Salazar M., Bedolla E., Drew R.A.L., Perez R. (2003). Improvement of the Mechanical Properties in a Nanocrystalline NiAl Intermetallic Alloy with Fe, Ga and Mo Additions. Mater. Sci. Eng. A.

[110] Sheng L., Zhang W., Guo J., Yang F., Liang Y., Ye H. (2010). Effect of Au Addition on the Microstructure and Mechanical Properties of NiAl Intermetallic Compound. Intermetallics.

[111] Liu E., Gao Y., Jia J., Bai Y., Wang W. (2014). Microstructure and Mechanical Properties of in Situ NiAl–Mo2C Nanocomposites Prepared by Hot-Pressing Sintering. Mater. Sci. Eng. A.

[112] Darolia R., Dobbs J.R., Field R.D., Goldman E.H., Lahrman D.F., Waltson W.S. (1996). NiAl Intermetallic Alloy and Article with Improved High Temperature Strength. U.S. Patent.

[113] Chakravorty S., Wayman C.M. (1976). The Thermoelastic Martensitic Transformation Inβ′ Ni-Al Alloys: I. Crystallography and Morphology. Met. Trans. A.

[114] Chandrasekaran M., Beyer J., Delaey L. (1992). Some Questions on the Structure of Martensite and Precursor in Ni(<63at%)-Al Alloys. Scr. Metall. Et Mater..

[115] Hangen U.D., Sauthoff G. (1999). The Effect of Martensite Formation on the Mechanical Behaviour of NiAl. Intermetallics.

[116] Kainuma R., Ohtani H., Ishida K. (1996). Effect of Alloying Elements on Martensitic Transformation in the Binary NiAl(β) Phase Alloys. Met. Mater. Trans. A.

[117] Thompson R.J., Zhao J.-C., Hemker K.J. (2010). Effect of Ternary Elements on a Martensitic Transformation in β-NiAl. Intermetallics.

[118] Ozgen S., Adiguzel O. (2003). Molecular Dynamics Simulation of Diffusionless Phase Transformation in a Quenched NiAl Alloy Model. J. Phys. Chem. Solids.

[119] Guo Y.-F., Wang Y.-S., Wu W.-P., Zhao D.-L. (2007). Atomistic Simulation of Martensitic Phase Transformation at the Crack Tip in B2 NiAl. Acta Mater..

[120] Lazarev N., Abromeit C., Schäublin R., Gotthardt R. (2008). Atomic-Scale Simulation of Martensitic Phase Transformations in NiAl. Mater. Sci. Eng. A.

[121] Cui C.Y., Guo J.T., Qi Y.H., Ye H.Q. (2001). High Temperature Embrittlement of NiAl Alloy Induced by Hot Isostatic Pressing (HIPing) and Aging. Scr. Mater..

[122] Wang L., Shen J., Shang Z., Fu H. (2014). Microstructure Evolution and Enhancement of Fracture Toughness of NiAl–Cr(Mo)–(Hf,Dy) Alloy with a Small Addition of Fe during Heat Treatment. Scr. Mater..

[123] Bewlay B.P., Jackson M.R., Subramanian P.R., Zhao J.-C. (2003). A Review of Very-High-Temperature Nb-Silicide-Based Composites. Met. Mater. Trans. A.

[124] Bewlay B.P., Briant C.L., Jackson M.R., Subramanian P.R., Kmeringen G., Rodhammer P., Wildner H. (2001). Recent Advances in Nb-Silicide in-Situ Composites. Proceedings of the 15th International Plansee Seminar.

[125] Frommeyer G., Rablbauer R. (2008). High Temperature Materials Based on the Intermetallic Compound NiAl Reinforced by Refractory Metals for Advanced Energy Conversion Technologies. Steel Res. Int..

[126] Lin Lü B., Qing Chen G., Qu S., Su H., Long Zhou W. (2013). Effect of Alloying Elements on Dislocation in NiAl: A First-Principles Study. Phys. B Condens. Matter.

[127] Kawagishi K., Yeh A., Yokokawa T., Kobayashi T., Koizumi Y., Harada H., Huron E.S., Reed R.C., Hardy M.C., Mills M.J., Montero R.E., Portella P.D., Telesman J. (2012). Development of an Oxidation-Resistant High-Strength Sixth-Generation Single-Crystal Superalloy TMS-238. Superalloys 2012.

[128] Yuan Y., Kawagishi K., Koizumi Y., Kobayashi T., Yokokawa T., Harada H. (2014). Creep Deformation of a Sixth Generation Ni-Base Single Crystal Superalloy at 800 °C. Mater. Sci. Eng. A.

[129] Carter T.J. (2005). Common Failures in Gas Turbine Blades. Eng. Fail. Anal..

[130] Xu R., Geng Z., Wu Y., Chen C., Ni M., Li D., Zhang T., Huang H., Liu F., Li R. (2022). Microstructure and Mechanical Properties of In-Situ Oxide-Dispersion-Strengthened NiCrFeY Alloy Produced by Laser Powder Bed Fusion. Adv. Powder Mater..

[131] TWI Global (2025). What Is an Oxide Dispersion Strengthened (ODS) Alloy?.

[132] TWI Global (2025). What Are the Common Properties of Oxide Dispersion Strengthened (ODS) Alloys?. https://www.twi-global.com/technical-knowledge/faqs/faq-what-are-the-common-properties-of-oxide-dispersion-strengthened-ods-alloys.

[133] Pasebani S., Dutt A.K., Burns J., Charit I., Mishra R.S. (2015). Oxide Dispersion Strengthened Nickel Based Alloys via Spark Plasma Sintering. Mater. Sci. Eng. A.

[134] Yalcin M.Y., Derin B., Aydogan E. (2022). Development and Additive Manufacturing of Oxide Dispersion Strengthened Inconel 718: Thermochemical and Experimental Studies. J. Alloys Compd..

[135] Avery R.E., Tuthill A.H. (1994). Guidelines for the Welded Fabrication of Nickel Alloys for Corrosion Resistant Service.

[136] Xia C., Kou S. (2020). Evaluating Susceptibility of Ni-Base Alloys to Solidification Cracking by Transverse-Motion Weldability Test. Sci. Technol. Weld. Join..

[137] Choudhury B., Chandrasekaran M. (2017). Investigation on Welding Characteristics of Aerospace Materials—A Review. Mater. Today: Proc..

[138] INFITALAB (2025). 5 Types Of Welding Tests for Quality Assurance. https://infinitalab.com/welding/5-types-of-welding-tests-for-quality-assurance/.

[139] Applied Technical Services Lab (2025). Welding Testing. Destructive Weld Testing. https://atslab.com/testing-and-analysis/welding-testing/destructive-weld-testing/.

[140] Body & Paint Center Inc. (2025). Destructive Weld Testing Explained. https://bodyandpaintcenter.com/destructive-weld-testing-explained/.

[141] Tulsa Welding School (2025). Tensile Strength Testing in Welding.

[142] Inspection for Industry LLC (2025). What Is Weld Destructive Testing?. https://www.inspection-for-industry.com/weld-destructive-testing.html.

[143] NextGen Material (2024). Testing Top 10 Most Common ASTM Standards for Metal Testing. https://www.nextgentest.com/blog/top-10-most-common-astm-standards-for-metal-testing/.

[144] (2017). Underwater Welding Code.

[145] Red River (2025). What Are the Grades of Pressure Vessel Material?.

[146] MechTest (2025). Mechanical Testing: Everything You Need to Know. https://mechtest.com.au/information/mechanical-testing-everything-you-need-to-know.

[147] Tijs B.H.A.H., Turon A., Bisagni C. (2024). Characterization and Analysis of Conduction Welded Thermoplastic Composite Joints Considering the Influence of Manufacturing. Compos. Struct..

[148] Biodpi Bend (2025). Testing: Evaluation of Strength and Ductility of Materials. https://biopdi.com/bend-testing/.

[149] Dascau H. (2025). Destructive Testing of Welded Joints—Overview of Standard Requirements and Most Common Errors. https://www.google.com/url?sa=i&url=https%3A%2F%2Fproject-trust.eu%2Fimg%2Fdocuments%2Fpublic-articles%2FTRUST%2520-%2520Public%2520Article_ISIM.pdf&psig=AOvVaw1Qd0JO4QnJIXt44IssCJY1&ust=1749284187609000&source=images&cd=vfe&opi=89978449&ved=0CAQQn5wMahcKEwjAsv7FrdyNAxUAAAAAHQAAAAAQBA.

[150] Kubik K. (2025). Destructive Testing of Welded Joints—Overview of Standard Requirements and Most Common Errors. https://www.google.com/url?sa=t&source=web&rct=j&opi=89978449&url=https://project-trust.eu/img/documents/public-articles/TRUST%2520-%2520Public%2520Article_Lukasiewicz_Instytut.pdf&ved=2ahUKEwiG35_AsNyNAxVjA9sEHYBfJu4QFnoECBgQAQ&usg=AOvVaw3BMJPHGKFg3eGw7u8D9Ics.

[151] WERMAC (2025). ASTM—Nondestructive Testing Standards. https://www.wermac.org/societies/astm_ndt_std_part1.html.

[152] ASTM (2025). ASTM Nondestructive Testing Standards. https://www.scribd.com/document/381202794/ASTM-Nondestructive-Testing-Standards-pdf.

[153] One Stop NTD (2021). Standards and Specifications for Welding. https://www.onestopndt.com/ndt-articles/standards-and-specifications-for-welding.

[154] Rzeszow University of Technology (2025). Non-Destructive Testing (NDT) Laboratory.

[155] ANSI (2025). Non-Destructive Weld Testing Standards. https://webstore.ansi.org/industry/safety-standards/welding-safety/non-destructive-weld-testing.

[156] (2025). One Stop NDT. What Are NDT Standards for Welding?. https://www.onestopndt.com/ndt-faq/what-are-ndt-standards-for-welding.

[157] Hinshaw E.B., Olson D.L., Siewert T.A., Liu S., Edwards G.R. (1993). Welding of Nickel Alloys. Welding, Brazing, and Soldering.

[158] O’Brien A., American Welding Society Welding Handbook (2015). Volume 5 Part 2: Materials and Applications.

[159] Janaki Ram G.D., Venugopal Reddy A., Prasad Rao K., Madhusudhan Reddy G. (2004). Control of Laves Phase in Inconel 718 GTA Welds with Current Pulsing. Sci. Technol. Weld. Join..

[160] Kumar S.A., Sathiya P. (2015). Experimental Investigation of the A-TIG Welding Process of Incoloy 800H. Mater. Manuf. Process..

[161] Sivakumar J., Vasudevan M., Korra N.N. (2021). Effect of Activated Flux Tungsten Inert Gas (A-TIG) Welding on the Mechanical Properties and the Metallurgical and Corrosion Assessment of Inconel 625. Weld. World.

[162] Sonar T., Balasubramanian V., Malarvizhi S., Venkateswaran T., Sivakumar D. (2020). Effect of Heat Input on Evolution of Microstructure and Tensile Properties of Gas Tungsten Constricted Arc (GTCA) Welded Inconel 718 Alloy Sheets. Metallogr. Microstruct. Anal..

[163] Ramkumar K.D., Kumar B.M., Krishnan M.G., Dev S., Bhalodi A.J., Arivazhagan N., Narayanan S. (2015). Studies on the Weldability, Microstructure and Mechanical Properties of Activated Flux TIG Weldments of Inconel 718. Mater. Sci. Eng. A.

[164] Hanif M., Shah A.H., Shah I., Mumtaz J. (2023). Optimization of Bead Geometry during Tungsten Inert Gas Welding Using Grey Relational and Finite Element Analysis. Materials.

[165] Davila-Iniesta E.M., López-Islas J.A., Villuendas-Rey Y., Camacho-Nieto O. (2025). Automatic Segmentation of Gas Metal Arc Welding for Cleaner Productions. Appl. Sci..

[166] Ferro P., Zambon A., Bonollo F. (2005). Investigation of Electron-Beam Welding in Wrought Inconel 706—Experimental and Numerical Analysis. Mater. Sci. Eng. A.

[167] Janaki Ram G.D., Venugopal Reddy A., Prasad Rao K., Reddy G.M., Sarin Sundar J.K. (2005). Microstructure and Tensile Properties of Inconel 718 Pulsed Nd-YAG Laser Welds. J. Mater. Process. Technol..

[168] Sidharth D., KV P.P. (2019). Microstructure and Properties of Inconel 718 and AISI 416 Laser Welded Joints. J. Mater. Process. Technol..

[169] Sun J., Ren W., Nie P., Huang J., Zhang K., Li Z. (2019). Study on the Weldability, Microstructure and Mechanical Properties of Thick Inconel 617 Plate Using Narrow Gap Laser Welding Method. Mater. Des..

[170] Brien A.O., Guzman C. (2014). Welding Handbook, Welding Processes, Part 2.

[171] Damodaram R., Raman S.G.S., Rao K.P. (2013). Microstructure and Mechanical Properties of Friction Welded Alloy 718. Mater. Sci. Eng. A.

[172] Lemos G.V.B., Hanke S., Dos Santos J.F., Bergmann L., Reguly A., Strohaecker T.R. (2017). Progress in Friction Stir Welding of Ni Alloys. Sci. Technol. Weld. Join..

[173] Lemos G.V.B., Meinhardt C.P., Dias A.R.D.P., Reguly A. (2021). Friction Stir Welding in Corrosion Resistant Alloys. Sci. Technol. Weld. Join..

[174] Cabibbo M., Forcellese A., Santecchia E., Paoletti C., Spigarelli S., Simoncini M. (2020). New Approaches to Friction Stir Welding of Aluminum Light-Alloys. Metals.

[175] Smith C., Grant G. (2023). Integrated Process Improvement Using Laser Processing and Friction Stir Processing for Nickel Alloys Used in Fossil Energy Power Plant Applications. https://netl.doe.gov/sites/default/files/netl-file/23FECM_19_Smith.pdf.

[176] Sharma A., Miura T., Morisada Y., Ushioda K., Singh S., Fujii H. (2024). Friction Stir Welding of Haynes 282 Ni Superalloy by Using a Novel Hemispherical Tool. Sci. Rep..

[177] Lippold J.C., Rodelas J.M., Rule J.R. (2013). Friction Stir Processing of Ni-Base Alloys. Proceedings of the 1st International Joint Symposium on Joining and Welding.

[178] Sonar T., Balasubramanian V., Malarvizhi S., Venkateswaran T., Sivakumar D. (2020). Multi-Response Mathematical Modelling, Optimization and Prediction of Weld Bead Geometry in Gas Tungsten Constricted Arc Welding (GTCAW) of Inconel 718 Alloy Sheets for Aero-Engine Components. Multiscale Multidiscip. Model. Exp. Des..

[179] Sabhadiya J. (2025). 10 Types of Welding Explained: MIG, TIG, SMAW & More. https://www.mechdaily.com/types-of-welding.

[180] Chinoy N. (2019). Increasing Weld Speeds in GTAW. https://b2bpurchase.com/increasing-weld-speeds-in-gtaw/#:~:text=G%20as%20Tungsten%20Arc%20Welding%20.

[181] Mohamed A.M., Moatasem M.K. (2020). Effects of Welding Parameters on Characterization and Mechanical Properties of Steel 37 Weldments. J. Eng. Sci. Assiut Univ. Fac. Eng..

[182] MITUSA Inc. (2025). 5 Types of Welding Methods & Their Usage.

[183] Universal Technical Institute (2025). Welding Types: Arc Welding Guide. https://www.uti.edu/programs/welding/types-welding.

[184] Tulsa Welding School (2025). Your Ultimate Guide to Every Different Type of Welding.

[185] Minnick W.H. (1996). Gas Tungsten Arc Welding Handbook.

[186] Antonini J.M. (2014). Health Effects Associated with Welding. Comprehensive Materials Processing.

[187] Singh R. (2012). Welding Corrosion Resistant Alloys—Stainless Steel. Applied Welding Engineering.

[188] Houldcroft P. Principles and Basic Characteristics of Welding and Related Processes. In Which Process? Elsevier: Amsterdam, The Netherlands, 1990; pp. 37–93, ISBN 978-1-85573-008-3.

[189] Weman K. (2012). Arc Welding: An Overview. Welding Processes Handbook.

[190] Pfeifer M. (2009). Manufacturing Process Considerations. Materials Enabled Designs.

[191] Alves Do Carmo D., Rocha De Faria A. (2015). A 2D Finite Element with through the Thickness Parabolic Temperature Distribution for Heat Transfer Simulations Including Welding. Finite Elem. Anal. Des..

[192] Chen S., Yan Z., Jiang F., Lu Z. (2016). The Pressure Distribution of Hollow Cathode Centered Negative Pressure Arc. J. Manuf. Process..

[193] Zhou J., Tsai H.L. (2005). Welding Heat Transfer. Processes and Mechanisms of Welding Residual Stress and Distortion.

[194] Singh R. (2012). Welding and Joining Processes. Applied Welding Engineering.

[195] Sashank S.S., Rajakumar S., Karthikeyan R., Nagaraju D.S. (2020). Weldability, Mechanical Properties and Microstructure of Nickel Based Super Alloys: A Review. E3S Web Conf..

[196] TWI Global (2025). Weldability of Materials—Nickel and Nickel Alloys.

[197] Goldiez R. 15 Types of Welding Processes with Their Advantages and Limitations. Hirebitics 2024. https://blog.hirebotics.com/types-of-welding-processes.

[198] New England Institute of Technology (2020). 4 Different Types of Welding Procedures and When to Use Them.

[199] Inspectioneering (2025). Common Welding Processes.

[200] (2024). SENLiSWeld—Marc Types of Welding Process. https://www.senlisweld.com/types-of-welding-process/.

[201] SchuetteMetals (2025). How Well Do You Know the 5 Most Popular Welding Methods?. Fab Times Schuette Metals Blog..

[202] (2015). WeldingEngineer Flux Cored Welding. http://www.weldingengineer.com/1flux.htm.

[203] Miller Electric Mfg Co. (2006). Solid Wire versus Flux Cored Wire-When to Use Them and Why.

[204] Steel Fabrication (2020). Service Important Welding Terminology. https://steelfabservices.com.au/important-welding-terminology/#:~:text=SMAW%20(%20Shielded%20Metal%20Arc%20Welding%20),Arc%20Welding%20)%20it’s%20at%20the%20centre.

[205] Siefert J.A., Shingledecker J.P., DuPont J.N., David S.A. (2016). Weldability and Weld Performance of Candidate Nickel Based Superalloys for Advanced Ultrasupercritical Fossil Power Plants Part II: Weldability and Cross-Weld Creep Performance. Sci. Technol. Weld. Join..

[206] Ramalingam D., Veerappagounder B., Rangaswamy S. (2023). Thermomechanical Simulation of Heat-Affected Zones in Nickel-Free High Nitrogen Stainless Steel: Microstructural Evolution and Mechanical Property Studies. J. Mater. Res..

[207] Baghel P.K. (2022). Effect of SMAW Process Parameters on Similar and Dissimilar Metal Welds: An Overview. Heliyon.

[208] El-Shennawy M., Abdel-Aleem H.A., Ghanem M.M., Sehsah A.M. (2024). Effect of Welding Parameters on Microstructure and Mechanical Properties of Dissimilar AISI 304/Ductile Cast Iron Fusion Welded Joints. Sci. Rep..

[209] Nickel Institute (2018). Guidelines for the Welded Fabrication of Nickel Alloys for Corrosion-Resistant Service.

[210] Eid A., Abd Allatif M., Elsabbagh A., Halfa H. (2023). Effect of Heat Input on Weldability of Low Nickel High Manganese Stainless Steel. Int. J. Mater. Technol. Innov..

[211] Mosallaee M., Semiromi M.T. (2021). Effect of Nickel Content on the Microstructural, Mechanical and Corrosion Behavior of E7018-G Electrode Weld Metal. J. Mater. Eng. Perform..

[212] Pouranvari M. (2010). On the Weldability of Grey Cast Iron Using Nickel Based Filler Metal. Mater. Des..

[213] (2020). Sodel Welding Nickel Alloys. https://www.sodel.com/en/blog/nickel/welding-nickel-alloys#:~:text=Weld%20penetration%20for%20nickel%20and,gasses%20from%20the%20weld%20bead.

[214] (2002). Total Materia AG Welding of Nickel Alloys. https://www.totalmateria.com/en-us/articles/welding-of-nickel-alloys/.

[215] Kumar N., Kumar P., Pandey C. (2025). Comparative Study on the SMAW Dissimilar Welding of IN718 and ASS304L Using ENiCrCoMo-1 and ENiCrFe-3 Electrodes. Mater. Chem. Phys..

[216] Bansod A.V., Patil A.P. (2017). Effect of Welding Processes on Microstructure, Mechanical Properties, and Corrosion Behavior of Low-Nickel Austenitic Stainless Steels. Metallogr. Microstruct. Anal..

[217] Yu C., Kawabata T., Kyouno S., Li X., Uranaka S., Maeda D. (2024). Influence of Welding Methods on the Microstructure of Nickel-Based Weld Metal for Liquid Hydrogen Tanks. J. Mater. Sci..

[218] El-Sayed Seleman M., Ahmed M., Abd E., Mahmoud E., El Soeudy R. (2023). Effect Of Multi-Pass Shielded Metal Arc Welding on Microstructure Characterization and Mechanical Properties of 2507 Super Duplex Stainless Steel. J. Egypt. Soc. Tribol..

[219] Addai B., Gyimah K.O., Asumadu T.K., Anto M., Klenam D.E.P., Soboyejo W.O. (2024). Strain Gradient Plasticity in AISI A36 Plain Carbon Steel Weldment: Comparison of Butt and Lap Joint Configurations. Results Eng..

[220] SchuetteMetals (2025). What Does It Take to Master the Art of Shielded Metal Arc Welding?. Fab Times Schuette Metals Blog..

[221] Rodríguez M. (2024). Coated Electrodes: Guide to Selection and Proper Storage.

[222] EwacAlloys (2024). Choosing the Right Maintenance Welding Electrodes: A Comprehensive Guide.

[223] Sisodia R., Weglowski M., Sliwinski P. (2024). In Situ Localised Post-Weld Heat Treatment with Electron Beam Welding of S690QL Steel. J. Adv. Join. Process..

[224] Hameed F.H., Mohamed M.T., Nawi S.A., Gattmah J. (2021). Dissimilar Arc Stud Welding AISI 304/AISI 1008: Mechanical Properties. IOP Conf. Ser. Mater. Sci. Eng..

[225] Govindaraju K., Madeshwaren V. (2024). Exploration of TIG Welding’s Impact on the Structural Integrity and Metallographic Characteristics of AISI 316/AISI 410 Stainless Steel Joints. Matéria.

[226] Ghosh S. (2019). A Review Study of Welding Distortion of Thin Plate under Welding. IJRASET.

[227] Medvecká D., Nový F., Mičian M., Bokůvka O., Preisler D. (2022). Microstructural Changes in HAZ of Weld Joints of S960 MC Steel. Mater. Today Proc..

[228] Silveroak (2018). Engineering Collage Welding Defects. https://www.slideshare.net/slideshow/welding-defects-94873471/94873471#1.

[229] ParFabFieldServices (2024). Exotic Material Welding: Techniques and Technologies.

[230] Quality Aircraft Accessories (2025). 7 Most Common Welding Methods to Join Aeronautical Components. https://www.qaa.com/blog/7-most-common-welding-methods-to-join-aeronautical-components#:~:text=Friction%20stir%20welding%20joins%20dissimilar%20materials%2C%20such,carbon%20steels%20without%20post%2Dweld%20heat%20treatment%20(PWHT).

[231] Tillack D.J. Nickel Alloys and Stainless Steel for Elevated Temparature Service: Weldability Considerations. Proceedings of the Materials Solutions ′97 on Joining and Repair of Gas Turbine Components.

[232] Rathi Welding (2024). Welding in Extreme Environments: Challenges and Solutions for Underwater, Space, and Cryogenic Welding. https://www.linkedin.com/pulse/welding-extreme-environments-challenges-solu-tions-underwater-dddtc#:~:text=Shielded%20Metal%20Arc%20Welding%20(SMAW)%20and%20Gas,making%20it%20ideal%20for%20welding%20high%2Dnickel%20alloys.

[233] Puviyarasan M., Senthil T.S., Rathinasuriyan C., Shanmugasundar G. (2023). Influence of GTAW Parameters on the Mechanical Properties and Microstructure of Wire Arc Additive Manufactured Inconel 625. Mater. Today Proc..

[234] Kumar S., Madugula N.S., Kumar R., Kumar N., Giri J., Kanan M. (2024). An Extensive Analysis of GTAW Process and Its Influence on the Microstructure and Mechanical Properties of SDSS 2507. J. Mater. Res. Technol..

[235] Suthar F.V., Shah H.N., Parmar R.N., Chaudhari M.D., Bhatt P.M., Chaudhury S.K. (2023). Optimization of GTAW Process Parameters for Deposition of Nickel-Based Hardfacing Alloy Using Taguchi Method. Weld. World.

[236] Barrick E.J., DuPont J.N. (2019). Mechanical Properties and Microstructural Characterization of Simulated Heat-Affected Zones in 10 Wt Pct Ni Steel. Mater. Sci. Eng. A.

[237] Horton H.L., Jones F.D., Oberg E., Ryffel H.H., Oberg E., McCauley C.J. (2000). Machinery’s Handbook: A Reference Book for the Mechanical Engineer, Designer, Manufacturing Engineer, Draftsman, Toolmaker, and Machinist.

[238] Yaeger C. (2023). Welding Difficult and Dissimilar Metals.

[239] Chai T.X., Zhang L.L., Wang X.J., Xu H.T. (2021). The Role of ERNi-1 Wire on Microstructure and Properties of Pure Nickel N6 Plasma Arc Welding Joint. J. Mater. Res..

[240] Norrish J. (2006). Gases for Advanced Welding Processes. Advanced Welding Processes.

[241] Caruso S., Borda F., Sanguedolce M., Filice L. (2024). Finite Element and Experimental Analysis of Microstructural and Hardness Variations in Plasma Arc Welding of AISI 304 Stainless Steel. J. Manuf. Mater. Process..

[242] Chenrayan V., Shahapurkar K., Manivannan C., Rajeshkumar L., Sivakumar N., Rajesh Sharma R., Venkatesan R. (2024). Effect of Powder Composition, PTAW Parameters on Dilution, Microstructure and Hardness of Ni–Cr–Si–B Alloy Deposition: Experimental Investigation and Prediction Using Machine Learning Technique. Heliyon.

[243] Wu C.S., Wang L., Ren W.J., Zhang X.Y. (2014). Plasma Arc Welding: Process, Sensing, Control and Modeling. J. Manuf. Process..

[244] Airao J., Khanna N., Roy A., Hegab H. (2020). Comprehensive Experimental Analysis and Sustainability Assessment of Machining Nimonic 90 Using Ultrasonic-Assisted Turning Facility. Int. J. Adv. Manuf. Technol..

[245] Donnini R., Varone A., Palombi A., Spiller S., Ferro P., Angella G. (2025). High Energy Density Welding of Ni-Based Superalloys: An Overview. Metals.

[246] Manickam S.K., Palanivel I. (2024). Practical Implications of FSW Parameter Optimization for AA5754-AA6061 Alloys. Matéria (Rio J.).

[247] (2025). Huaxialmetal Incoloy Metals and Alloys: A Comprehensive Guide. https://www.huaxiaometal.com/blogs/incoloy-metals-and-alloys-a-comprehensive-guide.html.

[248] Khrais S., Al Hmoud H., Abdel Al A., Darabseh T. (2024). Experimental Investigation of the Impact of GMAW Welding Parameters on the Mechanical Properties of AISI 316L/ER 316L Using Quaternary Shielding Gas. Mater. Res. Express.

[249] Tukahirwa G., Wandera C., Kumar S. (2023). Influence of Process Parameters in Gas-Metal Arc Welding (GMAW) of Carbon Steels. Welding—Materials, Fabrication Processes, and Industry 5.0.

[250] Shafeek M., Suranjan S., Doreswamy D., Sachidananda H.K. (2024). Effect of Welding Parameters on Microstructure and Mechanical Properties of GMAW Welded S275 Steel Welded Zone. Discov. Mater..

[251] Zhang L., Okudan G., Basantes-Defaz A.-D.-C., Gneiting R.M., Subramaniam S., Ozevin D., Indacochea E. (2020). Characterization of GMAW (Gas Metal Arc Welding) Penetration Using Ultrasonics. Materials.

[252] Votruba V., Diviš I., Pilsová L., Zeman P., Beránek L., Horváth J., Smolík J. (2022). Experimental Investigation of CMT Discontinuous Wire Arc Additive Manufacturing of Inconel 625. Int. J. Adv. Manuf. Technol..

[253] Prakash J., Tewari S.P., Srivastava B.K. (2012). Shielding Gas for Welding of Aluminium Alloys by TIG/MIG Welding-A Review. Int. J. Mod. Eng. Res..

[254] Amiruddin M.H., Ismail M.E., Sumarwati S., Salleh M.R.M., Noor N.A.A. (2021). Parameters Optimization Using Factorial Analysis Method for Gas Metal Arc Welding (GMAW) Process. AIP Conf. Proc..

[255] Guy B. (2014). Low-Alloy Filler Metals: From A to W.

[256] Seabery (2025). A Comprehensive Guide to the Main Types of Welding Processes. https://seaberyat.com/en/guide-main-types-welding-processes/.

[257] Shook J. (2022). 4 Different Welding Methods and Where They Are Best Used. Southern Careers Institute. https://scitexas.edu/blog/different-welding-methods-used/.

[258] (2025). Hunter Structureal Steel. Types of Welding. The Four Main Techniques. https://huntersteel.com.au/types-of-welding/#:~:text=Flux%20Cored%20Arc%20Welding%20(FCAW)%20FCAW%20is,that%20when%20heated%20creates%20the%20gas%20shield.

[259] Arc Machines, Inc (2025). The Complete Guide to Orbital Welding. https://resources.arcmachines.com/the-complete-guide-to-orbital-welding-slp/.

[260] Reddy K.K., Jayadeva C.T., Ramesh Kumar S.C., Kaviti R.V.P., Bhowmik A., Prakash C. (2024). A Comparative Assessment of Chromium–Boron Hardfacing Using SMAW and FCAW Techniques. Sci. World J..

[261] Hwang J., Choi K., Lee S.M., Jung H.Y. (2022). Microstructural Evolution and Mechanical Properties of FCAW Joints in 9% Ni Steel Prepared with Two Types of Ni-Based Weld Metals. Int. J. Adv. Manuf. Technol..

[262] Mohamat S.A., Ibrahim I.A., Amir A., Ghalib A. (2012). The Effect of Flux Core Arc Welding (FCAW) Processes On Different Parameters. Procedia Eng..

[263] Weldas (2025). Comparison of Welding Processes: TIG and MIG Welding. https://www.weldaseurope.com/knowledge-base/comparison-of-welding-processes-tig-and-mig-welding/.

[264] (2025). G.E. Mathis Company Understanding Different Types of Welding. https://www.gemathis.com/different-welding-types#:~:text=Submerged%20Arc%20Welding%20(SAW)%20Requiring%20a%20continuous,protect%20the%20weld%20zone%20from%20atmospheric%20contamination.

[265] Andersson J., Ott E., Banik A., Andersson J., Dempster I., Gabb T., Groh J., Heck K., Helmink R., Liu X., Wusatowska-Sarnek A. (2014). Weldability of Ni-Based Superalloys. 8th International Symposium on Superalloy 718 and Derivatives.

[266] Huang Y., Huang J., Zhang J., Yu X., Li Q., Wang Z., Fan D. (2021). Microstructure and Corrosion Characterization of Weld Metal in Stainless Steel and Low Carbon Steel Joint under Different Heat Input. Mater. Today Commun..

[267] Chu Y., Chen Y., Chen Y., Liu P., Li X. (2020). Microstructure and Corrosion Behavior of Ni-Cr-Mo Nickel-Based Alloy Weld. J. Mater. Res..

[268] Oteplace (2025). Different Types of Welding and Their Ppe Requirements.

[269] Metal Exponents (2025). 5 Types of Welding Processes.

[270] LinkedIn (2025). Plasma Arc Welding. MET’s IIW-INDIA. https://www.linkedin.com/posts/met-s-iiw-chapter_plasma-welding-is-very-similar-to-tig-as-activity-7225486783554191360-U6vZ.

[271] Saju T., Velu M. (2021). Review on Welding and Fracture of Nickel Based Superalloys. Mater. Today: Proc..

[272] Joseph B., Katherasan D., Sathiya P., Murthy C.V.S. (2018). Weld Metal Characterization of 316L(N) Austenitic Stainless Steel by Electron Beam Welding Process. Int. J. Eng. Sci. Tech..

[273] Khodir S., Shibayanagi T., Takahashi M., Abdel-Aleem H., Ikeuchi K. (2014). Microstructural Evolution and Mechanical Properties of High Strength 3–9% Ni-Steel Alloys Weld Metals Produced by Electron Beam Welding. Mater. Des..

[274] Emerson J.N., Marrero-Jackson E.H., Nemets G.A., Okuniewski M.A., Wharry J.P. (2024). Nuclear Reactor Pressure Vessel Welds: A Critical and Historical Review of Microstructures, Mechanical Properties, Irradiation Effects, and Future Opportunities. Mater. Des..

[275] Tikweld (2025). The Evolution of Welding Technology: Past, Present, and Future.

[276] BaisonLaser (2025). SMAW vs. Laser Welding: Which One to Choose?.

[277] Chaurasiya M., Singh P.K., Mishra A. (2022). The Optimization of Various Parameters of Shielded Metal Arc Welding (SMAW) for a Cast Iron. J. Emerg. Technol. Innov. Res. (JETIR).

[278] Xu Y., Zhang S., Liu H., Cao X., Wang W., Yu W., Li J., Lai M. (2025). Microstructural Evolution and Impact Properties of Vacuum Laser Welded Near-Alpha Ti-6Al-3Nb-2Zr-1Mo Titanium Alloy: Effect of Base Metal Microstructure. Mater. Sci. Eng. A.

[279] CAEAssistant (2025). Abaqus Welding Simulation Complete Guide: Essential Methods and Theories Explained. https://caeassistant.com/blog/abaqus-welding-simulation-methods/#:~:text=It%20(%20Laser%20Beam%20Welding%20(LBW)%20),)%20suitable%20for%20aerospace%20and%20electronics%20industries.

[280] Han C., Jiang P., Geng S., Mi G., Wang C., Li Y. (2021). Nucleation Mechanisms of Equiaxed Grains in the Fusion Zone of Aluminum-Lithium Alloys by Laser Welding. J. Mater. Res. Technol..

[281] American Friction Welding, Inc. (2025). Automotive Welding.

[282] Pathak D., Singh R.P., Gaur S., Balu V. (2020). Experimental Investigation of Effects of Welding Current and Electrode Angle on Tensile Strength of Shielded Metal Arc Welded Low Carbon Steel Plates. Mater. Today Proc..

[283] Geng P., Qin G., Ma H., Zhou J., Ma N. (2021). Linear Friction Welding of Dissimilar Ni-Based Superalloys: Microstructure Evolution and Thermo-Mechanical Interaction. J. Mater. Res. Technol..

[284] Farbakhti M., Elmi Hosseini S.R., Mousavi Mohammadi S.A., Sadatabhari S., Yuan-ming H., Li R. (2025). Similar and Dissimilar Rotary Friction Welding of Steels: A Review of Microstructural Evolution and Mechanical Properties. J. Mater. Res. Technol..

[285] Winiczenko R. (2016). Effect of Friction Welding Parameters on the Tensile Strength and Microstructural Properties of Dissimilar AISI 1020-ASTM A536 Joints. Int. J. Adv. Manuf. Technol..

[286] Shanjeevi C., Arputhabalan J.J., Dutta R. (2017). Pradeep Investigation on the Effect of Friction Welding Parameters on Impact Strength in Dissimilar Joints. IOP Conf. Ser. Mater. Sci. Eng..

[287] Chamanfar A., Jahazi M., Cormier J. (2015). A Review on Inertia and Linear Friction Welding of Ni-Based Superalloys. Metall. Mater. Trans. A.

[288] Lang R. (2024). Innovate and Excel: Modern Thick Metal Welding Techniques.

[289] (2025). Stirweld Mounting Bracket and FSW: Friction Stir Welding Performance in Response to the Welder Shortage. https://stirweld.com/en/mounting-bracket-fsw/#:~:text=FSW%20(%20Friction%20Stir%20Welding%20)%20welded,of%20the%20FSW%20tool%20in%20the%20workpiece.

[290] Samadi M.R., Ayaz M., Afshari M., Afkar A. (2023). An Investigation on the Friction Stir Welding of PP/TiO2 Nanocomposites for Improving the Tensile Strength and Hardness of the Weld Joint. Colloid. Polym. Sci..

[291] TWI Global (2025). Materials Weldable by Friction Stir.

[292] Manufacturing Technology, Inc. (2025). FAQs—Friction Stir Welding. https://www.mtiwelding.com/blog/faqs-friction-stir-welding/#:~:text=Because%20Friction%20Stir%20Welding%20creates%20extremely%20high%2Dquality%2C,aluminum%20sheets%2C%20extrusions%2C%20panels%2C%20and%20other%20products.

[293] El-Zathry N.E., Akinlabi S., Woo W.L., Patel V., Mahamood R.M. (2024). Taguchi-Based Optimisation of FSW Parameters for Advancement in Aerospace Materials: Al-Li 2060 Alloy. Heliyon.

[294] Abboud M., Dubourg L., Racineux G., Kerbrat O. (2024). Experimental Methodology to Identify Optimal Friction Stir Welding Parameters Based on Temperature Measurement. J. Manuf. Mater. Process..

[295] Khalafe W.H., Sheng E.L., Bin Isa M.R., Omran A.B., Shamsudin S.B. (2022). The Effect of Friction Stir Welding Parameters on the Weldability of Aluminum Alloys with Similar and Dissimilar Metals: Review. Metals.

[296] Sorensen C., Nelson T.W. (2007). Chapter 6—Friction Stir Welding of Ferrous and Nickel Alloys. Friction Stir Welding and Processing.

[297] Codinter, Inc. (2025). Orbital Welding: A Complete Guide.

[298] McClements D. (2025). SMAW vs. GMAW: What Are the Key Differences?.

[299] Kossel J. (2024). How to Choose the Right Welding Machine for Your Project.

[300] Milfit Boru (2025). Seamless Steel Pipe Welding Processes: Technical Information and Details.

[301] (2025). Badassweld Types of Welding. https://badassweldingproducts.com/blogs/blog-our-blog/types-of-welding#:~:text=If%20you’re%20working%20with%20thick%20materials%2C%20processes,types%20of%20welds%20you%20need%20to%20make.

[302] (2024). Arccaptain Applications and Advantages of GTAW (Gas Tungsten Arc Welding). https://www.arccaptain.com/blogs/article/advantages-of-gtaw#:~:text=When%20compared%20to%20GMAW%2C%20GTAW%20offers%20a,can%20result%20in%20a%20less%20precise%20weld.

[303] Lyttle K., Stapon W.F.G., Simplifying Shielding Gas Selection The Welder 2005. https://www.thefabricator.com/thewelder/article/consumables/simplifying-shielding-gas-selection#:~:text=As%20a%20rule%20of%20thumb%2C%20use%20GTAW,material%20or%20when%20welding%20out%20of%20position.

[304] Jacobsson J., Andersson J., Brederholm A., Hänninen H. (2016). Weldability of Ni-Based Superalloys Waspaloy^®^ and Haynes^®^ 282^®^—A Study Performed with Varestraint Testing. Res. Rev. J. Mater. Sci..

[305] Hope A. (2025). Four Common Metallurgical Challenges When Welding Ni-Based Alloys and How to Mitigate Them.

[306] TWI Global (2025). Welding of Nickel Alloys—Part 1.

[307] Ardakani H.A., Naffakh-Moosavy H. (2019). The Effect of Pulsed Nd:YAG Laser Welding Parameters on Defects of Kovar to AISI 304L Dissimilar Joint. Opt. Laser Technol..

[308] Ikpe A.E., Idiong K.E., Bassey M. (2023). A Comprehensive Overview of Welding Defects and Associated Failure Mechanisms in Metal Joining Process.

[309] Liu Y., Yu D., Zhang Y., Zhou J., Sun D., Li H. (2023). Research Advances on Weldability of Mg Alloy and Other Metals Worldwide in Recent 20 Years. J. Mater. Res. Technol..

[310] (2024). Megmeet WeldingTechnology Mastering MIG Welding Tips for High Carbon Steel Welding. https://www.megmeet-welding.com/en/news/mig-welding-high-carbon-steel#:~:text=3)%20Control%20of%20Heat%20Input:%20Excessive%20heat,undesirable%20microstructures%20and%20cracking.%20Control%20heat%20by:.

[311] Winsor F.J., Olson D.L., Siewert T.A., Liu S., Edwards G.R. (1993). Welding of Low-Alloy Steels. Welding, Brazing, and Soldering.

[312] Bhatia A. (2025). Introduction to Welding and Non-Destructive Testing (NDT).

[313] SECIndustrial (2025). Is It Difficult to Weld Different Kinds of Metal Together?. https://www.secindustrial.com/blog/is-it-difficult-to-weld-different-kinds-of-metal-together/#:~:text=Welding%20different%20kinds%20of%20metals%20together%20can%20be%20challenging%2C%20but,to%20successfully%20weld%20dissimilar%20metals.

[314] Hodúlová E., Šimeková B., Kovaříková I., Sahul M. (2020). Experimental Study of Nickel Electron Beam Welding. Mater. Sci. Forum.

[315] Ma M., Wei W., Zhang H., Yao Z., Sun Y., Chen M., Wu F. (2025). Dissimilar Gas Tungsten Arc Welding of AlCoCrFeNi2.1 Eutectic High Entropy Alloy to 316L Stainless Steel. J. Mater. Res. Technol..

[316] TWI Global Weldability of Materials—Carbon Manganese and Low Alloy Steels 25. https://www.twi-global.com/technical-knowledge/job-knowledge/weldability-of-materials-carbon-manganese-and-low-alloy-steels-019#:~:text=Solidification%20cracking%20is%20best%20avoided%20by%20careful,high%20manganese%20and%20silicon%20contents%20are%20preferred.

[317] Satheeshkumar V., Narayanan R.G., Gunasekera J.S. (2023). Sustainable Manufacturing. Sustainable Manufacturing Processes.

[318] Popović O., Prokić-Cvetković R., Burzić M., Lukić U., Beljić B. (2014). Fume and Gas Emission during Arc Welding: Hazards and Recommendation. Renew. Sustain. Energy Rev..

[319] Yusof F., Jamaluddin M.F. (2014). Welding Defects and Implications on Welded Assemblies. Comprehensive Materials Processing.

[320] Scott Process Systems, Inc. (2023). Navigating the Welding Maze: GTAW, GMAW, SMAW, and FCAW in Industrial Piping.

[321] Welding and Welder (2023). Understanding TIG Filler Rods: A Comprehensive Guide for Welders.

[322] Di Bella G., Favaloro F., Borsellino C. (2023). Effect of Process Parameters on Friction Stir Welded Joints between Dissimilar Aluminum Alloys: A Review. Metals.

[323] Andersson J., Hosseini V., Neikter M., Pederson R. (2023). Welding of Special Alloys. Welding of Metallic Materials.

[324] Cai J., Zhang H., Wu X., Liu Y., Wu Y., Wang J., Zhang C., Sun B., Wu F. (2024). Straw Return Decreases Polycyclic Aromatic Hydrocarbon (PAH) Accumulation in Winter Wheat and Human Health Risk by Enhancing PAH Dissipation in Rhizosphere Soil. Pedosphere.

[325] Li L., Du Z., Sheng X., Zhao M., Song L., Han B., Li X. (2022). Comparative Analysis of GTAW+SMAW and GTAW Welded Joints of Duplex Stainless Steel 2205 Pipe. Int. J. Press. Vessel. Pip..

[326] Verma J., Taiwade R.V. (2017). Effect of Welding Processes and Conditions on the Microstructure, Mechanical Properties and Corrosion Resistance of Duplex Stainless Steel Weldments—A Review. J. Manuf. Process..

[327] Nath R.K., Maji P., Sinha A.K., Karmakar R., Paul P. (2022). Effect of Different Electrode Angles as Well as Weld Direction on the Bead Geometry of Submerge Arc Welding Process. Mater. Today Proc..

[328] Karamimoghadam M., Rezayat M., Contuzzi N., Denora V., Mateo A., Casalino G. (2025). Effect of Wire Feed Rate on ER70S-6 Microstructure of Wire Arc Additive Manufacturing Process. Int. J. Adv. Manuf. Technol..

[329] Popović O., Prokić C., Burzić R.M., Milutinović Z. The Effect of Heat Input on the Weld Metal Toughness of Surface Welded Joint. Proceedings of the 14th International Research/Expert Conference.

[330] Veeman D., Siva Shanmugam N., Sathish T., Petle V., Sriram G. (2020). Experimental Investigation of Plasma Arc Welded Ti–6Al–4V Sheets. Trans. Can. Soc. Mech. Eng..

[331] Khan A.U., Madhukar Y.K. (2021). Effects of Pillar-Based Substrate on the Wire Arc Additive Manufacturing Process. Int. J. Precis. Eng. Manuf..

[332] Iqbal H., Ascari A., Fortunato A., Liverani E. (2025). Elucidating the Effects of Metal Transfer Modes and Investigating the Material Properties in Wire-Arc Additive Manufacturing (WAAM). Prog. Addit. Manuf..

[333] Ajithkumar S., Arulmurugan B. (2024). Microstructure and Mechanical Properties of Inconel 686 Fabricated by Gas Metal Arc Welding-Based Wire Arc Directed Energy Deposition: Impact of Cryogenic Treatments. J. Mater. Sci..

[334] SchuetteMetals (2025). SMAW vs. FCAW: Sparking a Debate About These Popular Welding Methods.

[335] Singh M. (2024). Arc Welding, Smaw, Saw, Mig, Tig Advantage. https://www.slideshare.net/slideshow/arc-welding-smaw-saw-mig-tig-advantage/265377149.

[336] Gupta R.K., Anil Kumar V., Sukumaran A., Kumar V. (2018). High-Temperature Tensile Behaviors of Base Metal and Electron Beam-Welded Joints of Ni-20Cr-9Mo-4Nb Superalloy. Met. Mater. Trans. A.

[337] Barua A., Pradhan S., Priyadarshini M., Patra A., Kumari K. (2025). Recent Advancement in Tungsten Heavy Alloy Processing for Different Industrial Applications. J. Mater. Eng. Perform..

[338] Araujo J.V.D.S., Milagre M.X., Calderón-Hernández J.W., Morais N.W., Costa I. (2025). Friction Stir Welding Effects on the Corrosion Resistance of the 2098-T351 Alloy. Materialia.

[339] Handa A., Chawla V. (2016). Experimental Evaluation of Mechanical Properties of Friction Welded Dissimilar Steels under Varying Axial Pressures. Stroj. Cas.—J. Mech. Eng..

[340] Xi B., Liu B., Li S., Wang D., Zhang Y., Szakálos P., Ejenstam J., Wallenius J., He G., Zhang W. (2022). Influence of TIG and Laser Welding Processes of Fe-10Cr-4Al-RE Alloy Cracks Overlayed on 316L Steel Plate. Materials.

[341] Zhang Y., Jiang X., Fang Y., Fang Y., Liu B., Sun H., Shao Z., Song T. (2021). Research and Development of Welding Methods and Welding Mechanism of High-Entropy Alloys: A Review. Mater. Today Commun..

[342] Sri Ram Vikas K., Rao K.S., Rahul, Reddy G.M., Venkata Ramana V.S.N. (2022). Influence of Heat Treatments on Microstructural and Mechanical Properties of Grade 5 Titanium Friction Welds. Eng. Res. Express.

[343] Zhang J., Zhang Y., Chen X., Li Z., Huang G., Pan F. (2023). Improving Joint Performance of Friction Stir Welded AZ31/AM60 Dissimilar Mg Alloys by Double-Sided Welding. Mater. Sci. Eng. A.

[344] Sirohi S., Kumar S., Kumar A., Kumar A., Pandey C. (2023). To Investigate the Effect of Process Parameters on the Dissimilar Welded Joint of AA7075 and Cu. Mater. Today Proc..

[345] Sindhuja M., Neelakrishnan S. (2021). Friction Stir Welding Parameters and Their Influence on Mechanical Properties of Welded AA6061 and AA5052 Aluminium Plates. Mater. Res. Express.

[346] Tong Y., Zhang L., Li C., Ma Y., Li P., Dong H. (2024). Microstructure and Mechanical Properties of IN690 Ni-Based Alloy/316LN Stainless-Steel Dissimilar Ring Joint Welded by Inertia Friction Welding. Materials.

[347] Tathgir S., Rathod D.W., Batish A. (2020). Process Enhancement Using Hydrogen-Induced Shielding: H_2_-Induced A-TIG Welding Process. Mater. Manuf. Process..

[348] Tamilkolundhu S., Josephraj F.X. (2025). Influence of CMT Welding Parameters on Microstructural and Properties Investigation of Dissimilar Weld Joint for Aluminum Bronze and Carbon Steel. Mater. Sci. Technol..

[349] Zhang Z., Qu S., Zhang Y., Zhang H., Lu X., Li B., Li H., Zhang T., Wu D., Chu P. (2025). Microstructure, Mechanical Properties, and Corrosion Resistance of DSS Laser-Welded Joints. Weld. World.

[350] Betini E.G., Gomes M.P., Mucsi C.S., Orlando M.T.D., Luz T.D.S., Avettand-Fènoël M.-N., Rossi J.L. (2019). Effect of Nitrogen Addition to Shielding Gas on Cooling Rates and in the Microstructure of Thin Sheets of Duplex Stainless Steel Welded by Pulsed Gas Tungsten Arc Welding Process. J. Mater. Res..

[351] Uzun H., Dalle Donne C., Argagnotto A., Ghidini T., Gambaro C. (2005). Friction Stir Welding of Dissimilar Al 6013-T4 To X5CrNi18-10 Stainless Steel. Mater. Des..

[352] Gupta D., Bansal A., Jindal S. (2024). Influence of SAW Flux Ingredients on Chemical Compositions of Weldments of Duplex Steel-2205. Trans. Indian. Inst. Met..

[353] Köse C., Topal C. (2019). Effect of Post Weld Heat Treatment and Heat Input on the Microstructure and Mechanical Properties of Plasma Arc Welded AISI 410S Ferritic Stainless Steel. Mater. Res. Express.

[354] Singh I.J., Murtaza Q., Kumar P. (2025). Effect of CMT Welding Process Parameters on the Microstructural and Mechanical Properties of Dissimilar Aluminum Alloys of AA8011 and AA6061. Trans. Indian. Inst. Met..

[355] Kumar S., Ramakrishna A., Madugula N.S., Kumar N., Kapoor M., Bhatia H.S. (2025). Evolution of Surface Morphology and Mechanical Characterization of GTAW Welded SDSS Thin Sheets. Results Surf. Interfaces.

[356] Nandan Banjare P., Kumar Dewangan S., Bhowmik A., Kumar Manoj M. (2023). Effect of Assisted Heating on Microstructure, Material Flow and Intermetallic Formation during Friction Stir Welding of Copper Alloy and AA 6063. Mater. Today Proc..

[357] (2025). Laser Welder Friction Stir Welding (FSW): Definition, Process, and Application. https://laser-welder.net/laser-welding/types/friction-stir/#:~:text=FSW%20(%20Friction%20Stir%20Welding%20)%20causes,stir%20zone%20is%20where%20intense%20deformation%20occurs.

[358] Fan J., Zhang J., Zhang D. (2024). Thermodynamic Insights into the Influence of Welding Current on Oxygen Levels in the Submerged Arc Welding Process. Processes.

[359] Lopez-Galilea I., Ruttert B., He J., Hammerschmidt T., Drautz R., Gault B., Theisen W. (2019). Additive Manufacturing of CMSX-4 Ni-Base Superalloy by Selective Laser Melting: Influence of Processing Parameters and Heat Treatment. Addit. Manuf..

[360] Siddiqui S.F., Fasoro A.A., Gordon A.P., Badiru A.B., Valencia V.V., Liu D. (2017). Selective Laser Melting (SLM) of Ni-Based Superalloys. A Mechanics of Materials Review. Additive Manufacturing Handbook: Product Development for the Defense Industry.

[361] Yap C.Y., Tan H.K., Du Z., Chua C.K., Dong Z. (2017). Selective Laser Melting of Nickel Powder. Rapid Prototyp. J..

[362] Konovalov S., Osintsev K., Golubeva A., Smelov V., Ivanov Y., Chen X., Komissarova I. (2020). Surface Modification of Ti-Based Alloy by Selective Laser Melting of Ni-Based Superalloy Powder. J. Mater. Res. Technol..

[363] Li R.D., Shi Y.S., Wang Z.G., Liu J.H. (2011). Selective Laser Melting of Multi-Component Ni-Based Powder Mixture for Building Metallic Parts. Mater. Sci. Forum.

[364] Duchna M., Cieślik I., Kloshek A., Adamczyk-Cieślak B., Zieniuk M., Moszczyńska D., Mizera J. (2022). Ni-Based Alloy 713C Manufactured by a Selective Laser Melting Method: Characteristics of the Microstructure. Rapid Prototyp. J..

[365] Petkov V.I. (2018). Alloy 718 Manufactured by AM Selective Laser Melting. Master’s Thesis.

[366] Jinoop A.N., Paul C.P., Mishra S.K., Bindra K.S. (2019). Laser Additive Manufacturing Using Directed Energy Deposition of Inconel-718 Wall Structures with Tailored Characteristics. Vacuum.

[367] Zhang J., Yan Y., Li B. (2022). Selective Laser Melting (SLM) Additively Manufactured CoCrFeNiMn High-Entropy Alloy: Process Optimization, Microscale Mechanical Mechanism, and High-Cycle Fatigue Behavior. Materials.

[368] Yap C.Y., Chua C.K., Dong Z.L., Liu Z.H., Zhang D.Q., Loh L.E., Sing S.L. (2015). Review of Selective Laser Melting: Materials and Applications. Appl. Phys. Rev..

[369] Yang K., Huang Q., Wang Q., Chen Q. (2020). Competing Crack Initiation Behaviors of a Laser Additively Manufactured Nickel-Based Superalloy in High and Very High Cycle Fatigue Regimes. Int. J. Fatigue.

[370] Xia M., Gu D., Yu G., Dai D., Chen H., Shi Q. (2016). Selective Laser Melting 3D Printing of Ni-Based Superalloy: Understanding Thermodynamic Mechanisms. Sci. Bull..

[371] Chekotu J.C., Groarke R., O’Toole K., Brabazon D. (2019). Advances in Selective Laser Melting of Nitinol Shape Memory Alloy Part Production. Materials.

[372] (2023). JLC3DP What Is SLM 3D Printing. https://jlc3dp.com/help/article/What-is-SLM-3D-Printing.

[373] Starikov K., Polozov I., Borisov E., Kim A., Voevodenko D., Gracheva A., Shamshurin A., Popovich A. (2023). Selective Laser Melting of Non-Weldable Nickel Superalloy: Microstructure, Cracks and Texture. Metals.

[374] Zhang D., Li Y., Cong W. (2022). In-Situ Synthesis of High-Quality Pseudoelastic NiTi Alloys with Intrinsic Ni4Ti3 Phase Precipitation Using Laser DED. J. Manuf. Process..

[375] Zhang J., Lu F., Huang T., Li R., Zhang G., Liu L. (2023). An Advanced Approach to Improve the High-Temperature Property for Ni-Based Superalloys: Interface Segregation Manipulation. Mater. Sci. Eng. A.

[376] Chen H., Lu Y., Luo D., Lai J., Liu D. (2020). Epitaxial Laser Deposition of Single Crystal Ni-Based Superalloys: Repair of Complex Geometry. J. Mater. Process. Technol..

[377] Atli K.C., Boon H.M., Seede R., Zhang B., Elwany A., Arroyave R., Karaman I. (2021). Laser-Based Additive Manufacturing of a Binary Ni-5 Wt.%Nb Alloy. J. Manuf. Process..

[378] Cieslak M.J., Headley T.J., Romig A.D. (1986). The Welding Metallurgy of HASTELLOY Alloys C-4, C-22, and C-276. Met. Trans. A.

[379] Lingenfelter A.C. (1972). Varestraint Testing of Nickel Alloys. Weld. J..

[380] Cieslak M.J., Stephens J.J., Carr M.J. (1988). A Study of the Weldability and Weld Related Microstructure of Cabot Alloy 214. Met. Trans. A.

[381] Savage W.F., Nippes E.F., Goodwin G.M. (1977). Effect of Minor Elements on Hot-Cracking Tendencies of Inconel 600. Weld. J..

[382] Rebak R.B. (2011). Stress Corrosion Cracking (SCC) of Nickel-Based Alloys. Stress Corrosion Cracking.

[383] Chao W., Yuming L., Xu W., Yichao P., Yijun H., Ting B., Jixue L., Narayan R.L. (2022). Oxidation Assisted Recrystallization and Cracking at Grain Boundaries in Nimonic 80 A during Elevated Temperature Service. Corros. Sci..

[384] Subramani P., Manikandan M. (2018). Development of Welding Technique to Suppress the Microsegregation in the Aerospace Grade Alloy 80A by Conventional Current Pulsing Technique. J. Manuf. Process..

[385] Farhangi H., Samimi P. Fractographic and microstructural investigation of the failure of high temperature nimonic 80a insert bolts hassan farhangi and peyman samimi. Proceedings of the 8th International Fracture Conference.

[386] Riedel H., Wagner W. (1984). Creep Crack Growth in Nimonic 80a and in a 1cr-1/2mo Steel. Fracture 84.

[387] Chauvet E., Kontis P., Jägle E.A., Gault B., Raabe D., Tassin C., Blandin J.-J., Dendievel R., Vayre B., Abed S. (2018). Hot Cracking Mechanism Affecting a Non-Weldable Ni-Based Superalloy Produced by Selective Electron Beam Melting. Acta Mater..

[388] Islam M., Riad W.T., Al-Kharraz S., Abo-Namous S. (1991). Stress Corrosion Cracking Behavior of 90/10 Cu–Ni Alloy in Sodium Sulfide Solutions. Corrosion.

[389] Kohl A.L., Nielsen R.B. (1997). Mechanical Design and Operation of Alkanolamine Plants. Gas Purification.

[390] Wolski K. (2012). Environmentally Assisted Cracking (EAC) in Nuclear Reactor Systems and Components. Nuclear Corrosion Science and Engineering.

[391] Naeem M. (2013). Developments in Laser Microwelding Technology. Handbook of Laser Welding Technologies.

[392] Shoji T., Lu Z., Peng Q. (2011). Factors Affecting Stress Corrosion Cracking (SCC) and Fundamental Mechanistic Understanding of Stainless Steels. Stress Corrosion Cracking.

[393] Moradi Z., Lanjan A., Tyagi R., Srinivasan S. (2023). Review on Current State, Challenges, and Potential Solutions in Solid-State Batteries Research. J. Energy Storage.

[394] Petit J., Hénaff G., Sarrazin-Baudoux C. (2003). Environmentally Assisted Fatigue in the Gaseous Atmosphere. Comprehensive Structural Integrity.

[395] Pourazizi R., Mohtadi-Bonab M.A., Szpunar J.A. (2020). Investigation of Different Failure Modes in Oil and Natural Gas Pipeline Steels. Eng. Fail. Anal..

[396] Alloy J.R. (2025). Wheel Repair Can Cracked Alloys Be Repaired?. https://jralloywheelrepair.co.uk/can-cracked-alloys-be-repaired/#:~:text=Can%20Cracked%20Alloys%20Be%20Repaired?%20Cracked%20alloys,is%20essential%20for%20effective%20repair%20and%20prevention.

[397] SOS Gases, Inc. (2025). How Does Weld Cracking Occur and What Can Be Done about It?.

[398] Sonawane A., Roux G., Blandin J.-J., Despres A., Martin G. (2021). Cracking Mechanism and Its Sensitivity to Processing Conditions during Laser Powder Bed Fusion of a Structural Aluminum Alloy. Materialia.

[399] (2024). AWS Understanding Weld Cracking. https://www.aws.org/magazines-and-media/inspection-trends/it-may-24-feature-02-weld-cracks/#:~:text=The%20metallurgical%20characteristics%20of%20the,restraint%20can%20appreciably%20increase%20cracking.

[400] Khalifeh A., Huang Z.-M., Hemeda S. (2019). Stress Corrosion Cracking Damages. Failure Analysis.

[401] Xu M., Zhang H., Yuan T., Yan Z., Chen S. (2023). Microstructural Characteristics and Cracking Mechanism of Al-Cu Alloys in Wire Arc Additive Manufacturing. Mater. Charact..

[402] Pu Y., Chen S., Man C., Hou Y., Feng H., Wang W., Li W., Cheng Y.F., Tang D. (2023). Investigation on the Stress Corrosion Cracking Behavior and Mechanism of 90/10 Copper-Nickel Alloy under the Cooperative Effect of Tensile Stress and Desulfovibrio Vulgaris. Corros. Sci..

[403] Davies S.J., Jeffs S.P., Coleman M.P., Lancaster R.J. (2018). Effects of Heat Treatment on Microstructure and Creep Properties of a Laser Powder Bed Fused Nickel Superalloy. Mater. Des..

[404] Di Gianfrancesco A. (2017). Alloy 263. Materials for Ultra-Supercritical and Advanced Ultra-Supercritical Power Plants.

[405] Mueller F., Oechsner M., Speicher M., Klenk A., Shingledecker J., Takeyama M. (2019). Creep and Creep Crack Behavior of Alloy C-263 Used for Thick-Walled Components—An Update. Advances in Materials Technology for Power Plants.

[406] Simões B.D., Fernandes É.M.D., Marques E.A.S., Carbas R.J.C., Maul S., Stihler P., Weißgraeber P., Da Silva L.F.M. (2024). Development of a Cyclic Creep Testing Station Tailored to Pressure-Sensitive Adhesives. Machines.

[407] Maj P., Bochenek K., Sitek R., Koralnik M., Jonak K., Wieczorek M., Pakieła Z., Mizera J. (2023). Comparison of Mechanical Properties and Structure of Haynes 282 Consolidated via Two Different Powder Metallurgy Methods: Laser Powder Bed Fusion and Hot Pressing. Archiv. Civ. Mech. Eng..

[408] Yang Y. (2024). Optimization of Large Cast Haynes 282 Based on Thermal Induced Cracks: Formation and Elimination. Mech. Ind..

[409] Osoba L.O., Ojo O.A. (2012). Influence of Laser Welding Heat Input on HAZ Cracking in Newly Developed Haynes 282 Superalloy. Mater. Sci. Technol..

[410] Rozman K.A., Kruzic J.J., Sears J.S., Hawk J.A. (2015). Fatigue Crack Growth Mechanisms for Nickel-Based Superalloy Haynes 282 at 550-750 °C. J. Mater. Eng. Perform..

[411] Łyczkowska K., Adamiec J. (2017). Repair of Precision Castings Made of the Inconel 713C Alloy. Arch. Foundry Eng..

[412] Mohsin Raza M., Lo Y.-L. (2021). Experimental Investigation into Microstructure, Mechanical Properties, and Cracking Mechanism of IN713LC Processed by Laser Powder Bed Fusion. Mater. Sci. Eng. A.

[413] Wang G., Liu H., Tao X., Zhou S., Li J., Zou H., Hu D., Zhang B., Zheng L. (2024). Unraveling the Plastic Deformation, Recrystallization, and Oxidation Behavior of Waspaloy during Thermal Fatigue Crack Propagation. J. Alloys Compd..

[414] (2015). HighTempMetals Waspaloy Technical Data. https://www.hightempmetals.com/techdata/hitempWaspaloydata.php.

[415] Atabay S.E., Sanchez-Mata O., Muñiz-Lerma J.A., Gauvin R., Brochu M. (2020). Microstructure and Mechanical Properties of Rene 41 Alloy Manufactured by Laser Powder Bed Fusion. Mater. Sci. Eng. A.

[416] Kayacan R., Varol R., Kimilli O. (2004). The Effects of Pre- and Post-Weld Heat Treatment Variables on the Strain-Age Cracking in Welded Rene 41 Components. Mater. Res. Bull..

[417] Lu X., Ma Y., Ma Y., Wang D., Gao L., Song W., Qiao L., Johnsen R. (2023). Unravelling the Effect of F Phase on Hydrogen-Assisted Intergranular Cracking in Nickel-Based Alloy 725: Experimental and DFT Study. Corros. Sci..

[418] Wang M.S., Hanson J.P., Gradečak S., Demkowicz M.J. (2013). Cutting Apart of Γ″ Precipitates by Dislocations Emitted from Nanoscale Surface Notches in Ni-Base Alloy 725. Mater. Res. Lett..

[419] Kim Y., Bae J., Lee J., Kang H., Kim J.G., Kim S. (2024). Effect of Stabilization Annealing on Fatigue Crack Propagation Behavior of Inconel 706 Alloy at 25 and 650 °C. J. Mater. Res. Technol..

[420] Oh H., Kim S., Kim J.G., Taleghani F., Kim S. (2022). Low Cycle Fatigue Behavior of Inconel 706 at 650 °C. J. Mater. Res. Technol..

[421] Yan F., Li R., Li J., Wang Y., Wang C., Hu X. (2014). The Effect of Aging Heat Treatment on Microstructure and Mechanical Properties of Laser Welded Joints of Alloy GH909. Mater. Sci. Eng. A.

[422] Guo X., Kusabiraki K., Saji S. (2001). Intragranular Precipitates in Incoloy Alloy 909. Scr. Mater..

[423] FushunSpecialSteel (2025). INCOLOY^®^ Alloy 945—High-Strength Corrosion-Resistant Superalloy for Demanding Oil & Gas Applications. https://www.fushunspecialsteel.com/incoloy-alloy-945-n09945-superalloy/#:~:text=Resistance%20to%20Sulfide%20Stress%20Cracking%20FUSHUN%20METAL’s,the%20alloy’s%20suitability%20for%20sour%20service%20applications.

[424] AZOMaterials (2013). INCOLOY Alloy 945X (UNS N09945). https://www.azom.com/article.aspx?ArticleID=9512.

[425] Hippsley C.A., Strangwood M., DeVan J.H. (1990). Effects of Chromium on Crack Growth and Oxidation in Nickel Aluminide. Acta Metall. Et. Mater..

[426] Tan Q., Liu K., Li J., Geng S., Sun L., Skuratov V. (2024). A Review on Cracking Mechanism and Suppression Strategy of Nickel-Based Superalloys during Laser Cladding. J. Alloys Compd..

[427] Guo C., Li G., Li S., Hu X., Lu H., Li X., Xu Z., Chen Y., Li Q., Lu J. (2023). Additive Manufacturing of Ni-Based Superalloys: Residual Stress, Mechanisms of Crack Formation and Strategies for Crack Inhibition. Nano Mater. Sci..

[428] Hippsley C.A., DeVan J.H. (1990). Mechanisms of High Temperature Crack Growth in Nickel Aluminide. Mater. Sci. Technol..

[429] Qin X., Yan W., Liang Y., Li F. (2024). Effects of Crack–γ/Γ′ Interface Relative Distributions on the Deformation and Crack Growth Behaviors of a Nickel-Based Superalloy. RSC Adv..

[430] Ryou K., Ji Im H., Park J., Choi P.-P. (2023). Microstructural Evolution and Hot Cracking Prevention in Direct-Laser-Deposited Ni-Based Superalloy through Hf Addition. Mater. Des..

[431] Chai H., Wang L., Lin X., Zhang S., Yang H., Huang W. (2023). Microstructure and Cracking Behavior of Ni_3_Al-Based IC21 Alloy Fabricated by Selective Laser Melting. Mater. Charact..

[432] Wang Y., Yong Z., Sun K., Wen Q., Niu S., Yang Z. (2025). Microstructure and Mechanical Properties of SiC Ceramics and Kovar Alloy Joints Brazed with AgCuInTi and AgCuInTi + B Composite Fillers. Mater. Charact..

[433] Liu Z., Ma C., Chang Z., Yan P., Li F. (2023). Advances in Crack Formation Mechanism and Inhibition Strategy for Ceramic Additive Manufacturing. J. Eur. Ceram. Soc..

[434] Zhang X., Chen H., Xu L., Xu J., Ren X., Chen X. (2019). Cracking Mechanism and Susceptibility of Laser Melting Deposited Inconel 738 Superalloy. Mater. Des..

[435] Chandra S., Tan X., Narayan R.L., Wang C., Tor S.B., Seet G. (2021). A Generalised Hot Cracking Criterion for Nickel-Based Superalloys Additively Manufactured by Electron Beam Melting. Addit. Manuf..

[436] Li T., Wang Z., Hu S., Yang Z., Wang Y. (2022). Hot Cracking during the Fabrication of Inconel 625/Stainless Steel 308 L Functionally Graded Material by Dual-Wire Arc Additive Manufacturing. J. Manuf. Process..

[437] Sharahi H.J., Pouranvari M., Movahedi M. (2020). Enhanced Resistance to Liquation Cracking during Fusion Welding of Cast Magnesium Alloys: Microstructure Tailoring via Friction Stir Processing Pre-Weld Treatment. Mater. Sci. Eng. A.

[438] Chen S., Yu H., Lu N., Liang J., Zhang X., Mu Y., Chen L., Xu W., Li J. (2024). The Effect of Carbon Content on the Printability and Mechanical Properties of Ni-Based Superalloy in Laser Additive Manufacturing. Mater. Sci. Eng. A.

[439] Wang P., Zhao X., Yue Q., Xia W., Ding Q., Bei H., Gu Y., Zhang Z. (2023). Crack Initiation in Ni-Based Single Crystal Superalloy under Low-Cycle Fatigue-Oxidation Conditions. Metals.

[440] Rosalind J. (2024). Stress Relaxation Cracking in Industrial Systems: Key Insights for Reliability. https://becht.com/becht-blog/entry/stress-relaxation-cracking-in-industrial-systems-key-insights-for-reliability/#:~:text=Welding%20Residual%20Stresses:%20The%20welding%20method%20used,with%20high%20operating%20temperatures%2C%20exacerbated%20the%20cracking.

[441] Cai Q., Rey Rodriguez P., Carracelas Santos S., Castro G., Mendis C.L., Chang I.T.H., Assadi H. (2024). Crack Healing via Electropulsing Treatment Applied to Additive-Manufactured TiC/316L Stainless Steel Composites. Mater. Lett..

[442] Fang Y., Fossier T., White K.W. (2004). Crack Path Simulation and Identification in Polycrystalline Alumina. Scr. Mater..

[443] Li K., Ma R., Zhan J., Wu J., Fang J., Wang S., Yang Q., Gong N., Murr L.E., Cao H. (2024). Insight into the Cracking Mechanism of Super-Elastic NiTi Alloy Fabricated by Laser Powder Bed Fusion. Virtual Phys. Prototyp..

[444] Hassanipour M., Watanabe S., Li H., Hirayama K., Toda H., Takeuchi A., Uesugi K. (2020). A Surrogate Approach to Reveal Microstructural Mechanisms Controlling the 3D Short Crack Growth in a Ti-6Al-4V Alloy. MATEC Web Conf..

[445] Zhu G., Kang B., Zhu M.-L., Xuan F.-Z. (2024). Quantitative Assessment of Microstructural Damage for Interior Crack Initiation and High Cycle Fatigue Life Modeling. Mater. Sci. Eng. A.

[446] Zhang Z., Han Q., Liu Z., Wang L., Zhang H., Zhao P., Zhu G., Huang C., Setchi R. (2023). Cracking Behaviour and Its Suppression Mechanisms with TiB2 Additions in the Laser Additive Manufacturing of Solid-Solution-Strengthened Ni-Based Alloys. Compos. Part B Eng..

[447] Ryou K., Yoo B., Choi P.-P. (2021). On the Oxygen-Induced Hot Cracking in a Direct Laser Deposited Ni-Based Superalloy. Scr. Mater..

[448] Guo Q., Chen S., Wei M., Liang J., Liu C., Wang M. (2020). Formation and Elimination Mechanism of Lack of Fusion and Cracks in Direct Laser Deposition 24CrNiMoY Alloy Steel. J. Mater. Eng. Perform..

[449] Agyapong J., Mateos D., Czekanski A., Boakye-Yiadom S. (2024). Investigation of Effects of Process Parameters on Microstructure and Fracture Toughness of SLM CoCrFeMnNi. J. Alloys Compd..

[450] Xi X., Lin D., He Z., Ma R., Wei H., Shi Z., Zhao W., Chen B., Tan C., Dong Z. (2025). Additively Manufactured Crack-Free Nickel-Based Superalloy with Synergistic Strength and Plasticity via Grain Refinement and Striped Oxides Inhibition. Compos. Part B Eng..

[451] Ryou K., Park Y., Im H.J., Choi P.-P. (2024). Prevention of Hot Cracking in Ni-Based Superalloy via Passivation Layer Formation during Additive Manufacturing. J. Mater. Res. Technol..

[452] Park C.W., Byun J.M., Choi W.J., Lee S.Y., Kim Y.D. (2019). Improvement of High Temperature Mechanical Properties of Ni-Based Oxide Dispersion Strengthened Alloys by Preferential Formation of Y-Ti-O Complex Oxide. Mater. Sci. Eng. A.

[453] TWI Global (2025). Welding of Nickel Alloys—Part 2.

[454] Donachie M.J., Donachie S.J. (2002). Superalloys: A Technical Guide.

[455] (2025). Burns Stainless Inconel. https://burnsstainless.com/blogs/articles-1/inconel#:~:text=Inconel%20625%20can%20be%20welded,dissimilar%20metals%20including%20stainless%20steel.

[456] Sonar T., Balasubramanian V., Malarvizhi S., Venkateswaran T., Sivakumar D. (2021). An Overview on Welding of Inconel 718 Alloy—Effect of Welding Processes on Microstructural Evolution and Mechanical Properties of Joints. Mater. Charact..

[457] Kumar H., Ahmad G.N., Singh N.K. (2019). Activated Flux TIG Welding of Inconel 718 Super Alloy in Presence of Tri-Component Flux. Mater. Manuf. Process..

[458] (2025). SpecialMetals INCOLOY^®^ Technical Bulletins for INCONEL^®^ Alloys. https://www.specialmetals.com/documents/technical-bulletins/incoloy/.

[459] (2023). AntonMetal What Is Incoloy 926 Properties, and Applications. https://www.antonmetal.com/news/what-is-incoloy-926-properties-and-applications/.

[460] (2025). Metal Suppliers Online Super Alloy Nimonic 86(Tm). https://www.suppliersonline.com/propertypages/Nimonic86.asp.

[461] (2025). ZAPP AG Hastelloy C-22 Data Sheet. https://www.zapp.com/fileadmin/user_upload/HASTELLOY_C-22-alloy-Datasheet.pdf.

[462] Kou D., Chen Z., Chen Z., Li Y., Ma Y., Li Y. (2023). Evolution of Microstructure in Nickel-Based C-HRA-2 Alloy during Welding Thermal Simulation. Mater. Res. Express.

[463] DuPont J.N., Notis M.R., Marder A.R., Robino C.V., Michael J.R. (1998). Solidification of Nb-Bearing Superalloys: Part I. Reaction Sequences. Met. Mater. Trans. A.

[464] Richards N.L., Chaturvedi M.C. (2000). Effect of Minor Elements on Weldability of Nickel Base Superalloys. Int. Mater. Rev..

[465] Lippold J.C. (1983). Investigation of Heat-Affected Zone Hot Cracking in Alloy 800. Weld. J..

[466] Thompson R.G., Cassimus J.J., Mayo D.E., Dobbs J.R. (1985). Relationship Between Grain Size and Microfissuring in Alloy 718. Weld. J..

[467] Young G.A., Capobianco M.A., Penik M.A., Morris B.W., McGee J.J. (2008). The Mechanism of Ductility Dip Cracking in Nickel-Chromium Alloys. Weld. J..

[468] Special Metals (2025). NIMONIC^®^ Alloy 80A. https://www.specialmetals.com/documents/technical-bulletins/nimonic-alloy-80a.pdf.

[469] Special Metals (2025). NIMONIC^®^ Alloy 90. https://www.specialmetals.com/documents/technical-bulletins/nimonic-alloy-90.pdf.

[470] White H.J. (2010). Weldability of Haynes^®^ 282^®^ Alloy.

[471] Caron J., Pike L. (2014). Weldability of HAYNES 282 Superalloy after Long-Term Thermal Exposure. MATEC Web Conf..

[472] NikelInstitute (2025). Engineering Properties of ALLOY 713C. https://www.nickelinstitute.org/media/2487/alloys-713c_337.pdf.

[473] Mirak A., Shams B., Bakhshi S. (2022). Dissimilar Welding of Inconel 713 Superalloy and AISI 4140 Steel Using Nd:YAG Pulse Laser: An Investigation on the Microstructure and Mechanical Properties. Opt. Laser Technol..

[474] (2025). Haynes International HAYNES^®^ Waspaloy RTWTM Filler Metal. https://haynesintl.com/en/alloys/product-forms/wire-and-welding/tig-gtaw-and-mig-gmaw-filler-metal-products/haynes-waspaloy-rtw-filler-metal/.

[475] (2025). Machine MFG Rene 41 vs. Inconel: What’s the Difference?. https://shop.machinemfg.com/rene-41-vs-inconel-whats-the-difference/.

[476] (2024). MachineMFG Incoloy 909 (UNS N19909): Composition, Properties, and Uses. https://shop.machinemfg.com/incoloy-909-uns-n19909-composition-properties-and-uses/.

[477] (2025). Metal Suppliers Online Super Alloy Incoloy 909(Tm). https://www.metalsuppliersonline.com/propertypages/Incoloy909.asp.

[478] Gozlan E., Bamberger M., Dirnfeld S.F., Prinz B. (1992). Role of Zirconium in the Phase Formation at the Interdendritic Zone in Nickel-Based Superalloys. J. Mater. Sci..

[479] Thompson R.G., Genculu S. (1983). Microstructural Evolution in the Haz of Inconel 718 and Correlation With the Hot Ductility Test. Weld. J..

[480] Radhakrishnan B., Thompson R.G. (1991). A Phase Diagram Approach to Study Liquation Cracking in Alloy 718. Met. Trans. A.

[481] Molian P.A., Yang Y.M., Patnaik P.C. (1992). Laser Welding of Oxide Dispersion-Strengthened Alloy MA754. J. Mater. Sci..

[482] Çam G., Koçak M. (1998). Progress in Joining of Advanced Materials. Int. Mater. Rev..

[483] Maksymova S.V., Khorunov V.F., Myasoed V.V., Voronov V.V., Kovalchuk P.V. (2014). Microstructure of Brazed Joints of Nickel Aluminide. Paton Weld. J..

[484] Peng H. (2013). Brazing of Nickel, Ferrite and Titanium–Aluminum Intermetallics. Advances in Brazing.

[485] Trandafir N.-M., Arghir G. (2017). Research of Nickel-Aluminum Brazing. Acta Tech. Napoc..

[486] Yang T.Y., Wu S.K., Shiue R.K. (2001). Interfacial Reaction of Infrared Brazed NiAl/Al/NiAl and Ni_3_Al/Al/Ni_3_Al Joints. Intermetallics.

[487] Förner A., Giese S., Arnold C., Felfer P., Körner C., Neumeier S., Göken M. (2020). Nanoscaled Eutectic NiAl-(Cr,Mo) Composites with Exceptional Mechanical Properties Processed by Electron Beam Melting. Sci. Rep..

[488] Ning H., Wang D., Wang B., Liu G. (2021). Investigations on the NiAl–Cr(Mo) Eutectic Alloy with Optimized Microstructure and Improved Room-Temperature Compressive Properties. Mater. Sci. Eng. A.

[489] NTRS NASA (2013). Solid State Welding Processes for an Oxide Dispersion Strengthened Nickel-Chromium-Aluminum Alloy. https://ntrs.nasa.gov/api/citations/19750012449/downloads/19750012449.pdf.

[490] McGuiness P., Paulin I., Donik Č., Dobkowska A., Kubásek J., Pokorny J. (2025). Recent progress in oxide-dispersion-strengthened (ods) alloys produced by additive manufacturing. Mater. Technol..

[491] Li M., Wang L., Yang H., Zhang S., Lin X., Huang W. (2022). Microstructure and Mechanical Properties of Y_2_O_3_ Strengthened Inconel 625 Alloy Fabricated by Selective Laser Melting. Mater. Sci. Eng. A.

[492] TWI Global (2025). Joining ODS Alloys.

[493] YESWELDER (2025). How To Weld Nickel Alloys.

[494] O’Donnell D., Olson D.L., Siewert T.A., Liu S., Edwards G.R. (1993). Joining of Oxide-Dispersion-Strengthened Materials. Welding, Brazing, and Soldering.

[495] Onuki J., Nihei M., Funamoto T., Doi H., Fukui Y. (2001). Joining of Oxide Dispersion Strengthened Ni Based Super Alloys. Mater. Trans..

[496] Hejripour F., Aidun D.K. (2017). Consumable Selection for Arc Welding between Stainless Steel 410 and Inconel 718. J. Mater. Process. Technol..

[497] (2025). MetalCor Alloy 80A. https://www.metalcor.de/en/datenblatt/117/.

[498] (2025). Certilas CEWELD NiCrCo 282. https://certilas.com/en/product/nicrco-282.

[499] (2025). Virgamet Nickel and Nickel Alloys. https://virgamet.com/offer/c/nickel-cobalt-special-alloys.

